# Nanopore RNA Sequencing Revealed Long Non-Coding and LTR Retrotransposon-Related RNAs Expressed at Early Stages of Triticale SEED Development

**DOI:** 10.3390/plants9121794

**Published:** 2020-12-17

**Authors:** Ilya Kirov, Maxim Dudnikov, Pavel Merkulov, Andrey Shingaliev, Murad Omarov, Elizaveta Kolganova, Alexandra Sigaeva, Gennady Karlov, Alexander Soloviev

**Affiliations:** 1 Laboratory of Marker-Assisted and Genomic Selection of Plants, All-Russia Research Institute of Agricultural Biotechnology, Timiryazevskaya str. 42, 127550 Moscow, Russia; max.dudnikov.07@gmail.com (M.D.); paulmerkulov97@gmail.com (P.M.); kronstein491@yandex.ru (A.S.); muradok98@gmail.com (M.O.); liza.colg@gmail.com (E.K.); alexandra9954@gmail.com (A.S.); karlovg@gmail.com (G.K.); A.Soloviev70@gmail.com (A.S.); 2 Kurchatov Genomics Center of ARRIAB, All-Russia Research Institute of Agricultural Biotechnology, Timiryazevskaya Street, 42, 127550 Moscow, Russia; 3 Faculty of Computer Science, National Research University Higher School of Economics, Pokrovsky Boulvar, 11, 109028 Moscow, Russia

**Keywords:** long non-coding RNAs, seed development, Nanopore sequencing, retrotransposons, triticale

## Abstract

The intergenic space of plant genomes encodes many functionally important yet unexplored RNAs. The genomic loci encoding these RNAs are often considered “junk”, DNA as they are frequently associated with repeat-rich regions of the genome. The latter makes the annotations of these loci and the assembly of the corresponding transcripts using short RNAseq reads particularly challenging. Here, using long-read Nanopore direct RNA sequencing, we aimed to identify these “junk” RNA molecules, including long non-coding RNAs (lncRNAs) and transposon-derived transcripts expressed during early stages (10 days post anthesis) of seed development of triticale (AABBRR, 2*n* = 6*x* = 42), an interspecific hybrid between wheat and rye. Altogether, we found 796 lncRNAs and 20 LTR retrotransposon-related transcripts (RTE-RNAs) expressed at this stage, with most of them being previously unannotated and located in the intergenic as well as intronic regions. Sequence analysis of the lncRNAs provide evidence for the frequent exonization of Class I (retrotransposons) and class II (DNA transposons) transposon sequences and suggest direct influence of “junk” DNA on the structure and origin of lncRNAs. We show that the expression patterns of lncRNAs and RTE-related transcripts have high stage specificity. In turn, almost half of the lncRNAs located in Genomes A and B have the highest expression levels at 10–30 days post anthesis in wheat. Detailed analysis of the protein-coding potential of the RTE-RNAs showed that 75% of them carry open reading frames (ORFs) for a diverse set of GAG proteins, the main component of virus-like particles of LTR retrotransposons. We further experimentally demonstrated that some RTE-RNAs originate from autonomous LTR retrotransposons with ongoing transposition activity during early stages of triticale seed development. Overall, our results provide a framework for further exploration of the newly discovered lncRNAs and RTE-RNAs in functional and genome-wide association studies in triticale and wheat. Our study also demonstrates that Nanopore direct RNA sequencing is an indispensable tool for the elucidation of lncRNA and retrotransposon transcripts.

## 1. Introduction

Long non-coding RNAs (lncRNAs) are a diverse set of RNAs longer than 200 bp with no or very little coding potential. Traditionally, lncRNAs are considered to be protein non-coding, although many of them carry small open reading frames and encode functional peptides in different plants [1]. A broad range of functionality has been described for lncRNAs in plants, including the miRNA sponge, protein scaffolding, and post-transcriptional regulation of target genes via antisense pairing followed by mRNA decoy [2]. Based on their genomic localization regarding other genes, lncRNAs are broadly grouped into different classes: (1) lincRNAs or long non-coding intergenic RNAs; (2) NAT lncRNAs or natural anti-sense lncRNAs; (3) intronic lncRNAs, located in the introns; and (4) sense lncRNAs [3]. LncRNAs have been identified in many plant species and their expression in response to various abiotic and biotic stresses has been investigated, although our knowledge about lncRNA functions is still very limited [4,5,6,7,8,9]. However, the catalogue of lncRNAs for some plants with complex polyploidy and repeat-rich genomes, including wheat (*Triticum aestivum* L.), remains mainly underexplored [10].

Intergenic space and introns are frequently sources of lncRNA origin, although these genomic regions are enriched by insertions or remnants of transposable elements (TEs) [11]. Corroborating this, lncRNA sequences are more often associated with TEs than protein-coding genes and the bulk of them possess TE-related sequences [11,12,13,14,15,16]. For example, up to 75% of human lncRNAs have at least one exon with sequences originating from TEs [12,17]. A similar trend was demonstrated in some plant species, including maize, where 65% of lncRNAs had similarities to TEs [18]. More intriguingly, the TE-derived sequences may trigger the origin of new lncRNAs, providing a positive feedback loop with the evolution of lncRNAs [11,19]. TE-derived lncRNAs can have important and conserved biological functions [20]. For example, the rice lncRNA MIKKI is derived from LTR retrotransposons and has been shown to sequestrate miR171 and prevent degradation of its targets, mRNAs of SCARECROW-Like (SCL) transcription factors, in roots [20]. It is important to note that TEs can become transcriptionally and transpositionally active under certain circumstances, including stressful conditions, and in some developmental stages (e.g., microsporogenesis) and tissues (e.g., developing endosperm) [21,22,23]. However, individual TE transcripts and their coding potential have only been studied episodically in plants. The association of lncRNAs with repeat sequences like TEs makes the annotation of many lncRNAs challenging because of the ambiguity in the mapping of repeat-derived short RNAseq reads. RNAseq reads with multiple mapping positions in the genome sequence are frequently discarded from further analysis. Although some tools have been developed so far to overcome these obstacles, most of the lncRNA identification pipelines still ignore ambiguously mapped reads [24,25]. Thus, long-read sequencing technologies provide a great opportunity for transcriptome exploration, including the identification of transcribed repeats (e.g., transposable elements) or repeat-related transcripts [26]. Moreover, Panda and Slotkin [26] showed that by using Nanopore long cDNA reads, it is even possible to trace the expression of individual TEs from multicopy families in *Arabidopsis* and maize. The application of long-read technologies to the exploration of the lncRNA repertoire in plants has been demonstrated for several plant species, including *Oryza sativa* L. ssp. *japonica* [27], *Populus simonii* [28], *P. qiongdaoensis* [29], poplar “Nanlin 895” [30], *Trifolium pratense* [31], *Cardamine violifolia* [32], and *Vigna angularis* [33]. These studies demonstrated that long-read sequencing can be used to obtain a comprehensive catalogue of lncRNAs.

Grain development is one of the most important and practically relevant biological processes. It involves massive biochemical, physiological, and transcriptomic changes [34,35,36]. Wheat grain development is divided into five stages: (i) undifferentiated embryo and cellularization of the endosperm (0–7 days post anthesis (dpa)); (ii) embryo differentiation the embryo with formation of the main cell types (transfer cells, aleurone, starchy endosperm and the surrounding cells) (7–14 dpa); (iii) root and leaf primordia differentiation, full kernel development and the milk-ripe stage (14–21 dpa); (iv) further growth and differentiation of primary and lateral roots, and the dough stage of endosperm (21–31 dpa); (v) fully differentiated embryo (31–50 dpa) [34]. Studies of the transcriptome during wheat seed development have been extensively elucidated using RNAseq sequencing [35,37,38,39,40,41,42,43]. Global transcriptome analysis of developing seeds has shown that the expression of protein-coding genes is highly dynamic. Recently, Madhavan et al. (2020) used publicly available Illumina RNAseq reads and identified 443 lncRNAs expressed during the grain filling stage (14 and 30 dpa) [44]. It is currently unknown which types of lncRNA are expressed during other stages of wheat seed development.

Here, we used Nanopore long-read sequencing to discover lncRNAs, TE transcripts, and TE-related lncRNAs that are specifically expressed during the cell proliferation stage of seed development (10 dpa) in triticale (× *Triticosecale* Wittmack, AABBRR genome, 2*n* = 6*x* = 42) an interspecific hybrid between wheat and rye (*Secale cereale* L.). We identified 796 lncRNAs and 20 LTR retrotransposon-derived transcripts, with most of them being previously unannotated. The majority of the detected retrotransposon RNAs had a single intron, carried open reading frames (ORFs) encoding for a diverged set of GAG proteins, and were encoded by potentially autonomous and non-autonomous retrotransposons. The lncRNAs were also expressed during wheat seed development and had high stage specificity. Moreover, we found that lncRNA loci were biased toward frequent TE sequence exonization and were mainly located in the intergenic regions of A and B genomes of triticale. Our work explored the lncRNA landscape during the early stage of wheat and triticale seed development and provides a unique dataset for further functional studies of lncRNA and TEs, and their implications for seed development. Finally, the identified lncRNAs can be further incorporated into genome-wide association studies for marker-assisted improvement of the bread quality of modern triticale genotypes.

## 2. Material and Methods

### 2.1. Plant Material and DNA Isolation

For this study, the spring triticale line “L8665” obtained from the Department of Genetics, Russian State Agrarian University, was used. For DNA isolation, seeds of this line were germinated in the dark (room temperature) during 5–7 days and genomic DNA was isolated by the cetyltrimethylammonium bromide (CTAB) protocol [45].

### 2.2. Sample Collection and RNA Isolation

Plants of the spring triticale line “L8665” were grown in a greenhouse under natural light conditions. Developing seeds at 10 days post anthesis and flag leaves were dissected and placed into liquid nitrogen. RNA was isolated by the ExtractRNA kit (Evrogen, Moscow, Russia) following the manufacturer’s instructions. The RNA concentration and integrity were estimated by Nanodrop (Nanodrop Technologies, Wilmington, CA, USA) and gel electrophoresis using an 1.2% agarose gel with ethidium bromide staining.

### 2.3. RT-PCR

For RT-PCR, RNA was treated by DNAse I (Qiagen, Hilden, Germany, Q-79254) following the manufacturer’s instruction and used for cDNA synthesis. cDNA was synthesized using the MMLV RT kit (Evrogen, Moscow, Russia). Primers used for RT-PCR and the expected fragment lengths are listed in Table 1. The CDC (Cell division control protein, AAA-superfamily of ATPases; Ta54227) gene was used as a reference because of its high expression stability as shown by a previous study [46].

The PCR conditions were 94 °C for 1 min; 35 cycles of 94 °C for 1 min, 58 °C for 1 min, and 72 °C for 1 min; and a final elongation of 72 °C for 3 min.

### 2.4. Nanopore Direct RNA Sequencing and Transcript Assembly

Poly-A+ mRNA was purified from 100 µg of total RNA by the Dynabeads mRNA DIRECT Kit (ThermoFisher Scientific, Waltham, MA, USA) following the manufacturer’s instructions. Final poly-A+ RNA concentration was measured using a Quantus Fluorometer (Promega Corporation, Madison, WI, USA) and checked by gel electrophoresis. For Nanopore sequencing, a library was prepared from 1 µg Poly-A+ using the nanopore Direct RNA Sequencing Kit SQK-RNA002 (Oxford Nanopore Technologies, Oxford, UK). Direct RNA Sequencing (DRS) was carried out using MinION and flow cell FLO-MIN106. Basecalling was performed by Guppy (Version 4.0.11).

For transcript assembly, sequences of the A and B genomes and unanchored scaffolds (Un) of *Triticum aestivum* ([48]) downloaded from the EnsemblPlants server (https://plants.ensembl.org/Triticum_aestivum/Info/Index) were combined with *Secale cereale* genome sequences [49] into a single fasta file. Reads were mapped to the obtained fasta file by minimap2 software [50] with the ‘-ax splice’ argument. The obtained sam file was converted to a bam file by samtools [51] (samtools view -Sb) followed by sorting of the bam file by bamtools [52]. Transcript assembly was performed by StringTie2 [53] with the following arguments: –L –j 2 –f 0.05. The obtained gtf file was converted to gff format by the gffread tool [54]. The sorted bam and gff files were used for read mapping visualization by the locally installed JBrowse [55].

To obtain a high-confidence set of transcripts, we extracted transcript sequences using gtf from StringTie2 assembly by the gffread tool [54]. The reads were then mapped back to the transcripts using minimap2 software (settings: -ax map-ont), and the bam file with only primary alignments and mapping quality > 30 was obtained using samtools with –F 256 –q 30 –b parameters. This bam file was used to count the number of reads per transcripts and transcripts with > 5 DRS reads were selected.

### 2.5. Long Non-Coding RNA Prediction

To identify lncRNAs, all high-confidence transcripts with a length of >200 bp were selected using biopython [56]. Transcripts with open reading frame (ORF) lengths of >300 bp predicted by getorf were filtered out. Protein coding potential was calculated by three tools: LncFinder [57], PLEK [58], and CNCI [59]. LncFinder [57] was run in Rstudio Version 1.2.1335 (http://www.rstudio.com/) with R version 3.6.0. The following parameters were utilized: parallel.cores = 20, SS.features = TRUE, format = “DNA”, frequencies.file = “wheat”, svm.model = “wheat”. PLEK [58] and CNCI [59] were run with the default settings. Only transcripts identified by all three tools as non-coding and without similarity to Pfam domains were classified as lncRNAs.

To identify lncRNAs with exons overlapping the annotated TEs, we intersected CLARITE TE annotation (https://urgi.versailles.inra.fr/download/iwgsc/IWGSC_RefSeq_Annotations/v1.0/) with the genomic coordinates of the assembled exons.

### 2.6. Retrotransposon-Related Transcript Annotation

LTR retrotransposons (RTEs) were predicted in the genome using LTRharvest 1.5.10 with the default parameters [60] and LTRdigest 1.5.10 [61] with the following parameters: -aaout yes -pptlen 10 30 -pbsoffset 0 3 -pdomevalcutoff 0.001. Hidden markov model (HMM) profiles of RTE domains were downloaded from the GyDB database [62]. The gff3 file from the LTRdigest analysis was treated by a custom Python script (https://github.com/Kirovez/LTR-RTE-analysis/blob/master/LtrDiParser_v2.2.py) to extract sequences of LTR retrotransposons possessing similarity to any RTE domains including GAG, reverse transcriptase, RNAse H, aspartic protease, and integrase. To identify retrotransposon-related transcripts, the TEsorter tool (parameters: -eval 0.00001 -db gydb) was run with the set of all high-confidence transcripts. The transcripts with a similarity to RTE domains were manually checked in the locally installed JBrowse [55]. TEsorter data were also used for RTE classification. We also ran TEsorter with confident transcripts and the RExDB [63] database (-db rexdb) to identify the transcripts of Class II transposons but no transcripts were detected.

### 2.7. GAG Protein Analysis

To find GAG proteins, ORFs were predicted for RTE transcripts and Blastp with corresponding proteins was run followed. GAG proteins were aligned by MAFFT [64] with the standard parameters and a phylogenetic tree was constructed using iTOL [65]. The multiple alignment visualization was carried out in Jalview version 2.11.1.3 [66]. RNA-binding motifs (CX2CX4HX4C, where X is any amino acid) were identified by a custom Python script (https://github.com/Kirovez/LTR-RTE-analysis/blob/master/RBM_GAG_screen.py).

### 2.8. Gene Ontology Enrichment

Gene ontology (GO) enrichment analyses was performed using ShinyGO v0.61 [67] (http://bioinformatics.sdstate.edu/go/) with an false discovery rate (FDR) <0.01.

### 2.9. Expression Analysis

For RNA-Seq analysis of lncRNA and RTE transcript expression in different organs and development stages, publicly available data were used (Table 2). Reads were mapped on the de novo assembled transcriptome by Hisat2 [68] with the default options. The obtained files with alignments were used to calculate RPM (read per million reads) values for every transcript. For this purpose, we used Salmon v0.8.1 [69] and the quant command with the default parameters.

### 2.10. Extrachromosomal Circular DNA Isolation

Extrachromosomal circular DNA (eccDNA) was isolated and amplified according to the protocol of Lanciano et al. [72] with several modifications. Briefly, 5 µg of genomic DNA was treated by Plasmid-Safe ATP-Dependent DNAse (Epicenter, Madison, WI, USA) for 48 h according to the manufacturer’s instructions. DNA precipitation was carried out by 0.1 volume 3 M sodium acetate and 2.5 volume absolute ethanol, followed by overnight incubation at −20 °C. After centrifugation, the eccDNA pellet was obtained and exposed to the rolling circle amplification (RCA) reaction by the Illustra TempliPhi 100 Amplification Kit (GE Healthcare, Chicago, IL, USA) for 65 h at 28 °C. Detection of the eccDNA of LTR retrotransposon TaeST2.45518.1 (named ‘*MIG*’, location in wheat genome: 7B: 312,336,869…312,341,902 (5 kb)) was performed by inverse PCR with specific primers: Forward: CACACCACTAGCAACCTCCA ; Reverse: TGCTTGTGACAAGATGGGCA. The PCR conditions were 94 °C for 1 min; 35 cycles of 94 °C for 1 min, 58 °C for 1 min, 72 °C for 1 min; and final elongation at 72 °C for 3 min.

### 2.11. Statistics and Visualization

Statistical analysis was done in Rstudio Version 1.2.1335 (http://www.rstudio.com/) with R version 3.6.0. Bar plots, density plots, and box plots were drawn by ggplot2 [73]. Heatmaps were constructed by the ComplexHeatmap [74] R package.

## 3. Results

### 3.1. Direct Oxford Nanopore RNA Sequencing

**Figure 1:**
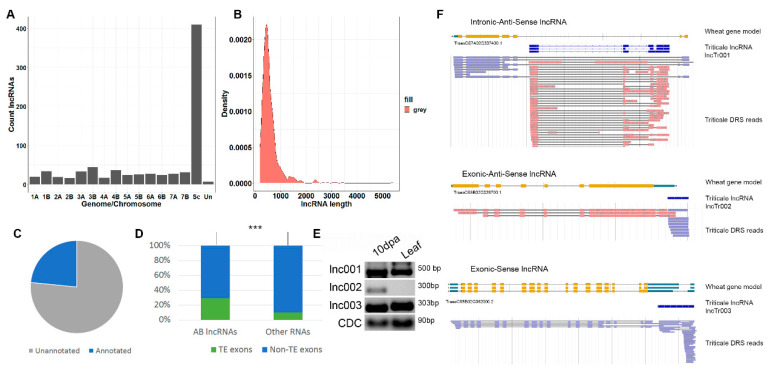
Identification and classification of triticale long non-coding RNAs (lncRNAs). (**A**) Bar plot showing the number of triticale lncRNA loci located on different chromosomes of Genomes A, B, and R and unanchored wheat contigs (Un). Sc indicates loci mapped to *Secale cereale* contigs. (**B**) Density plot of the lncRNA length distribution. (**C**) Pie graph showing the portion of the AB lncRNAs located in the unannotated genomic regions. (**D**) The portion of genes encoding AB lncRNAs and other RNAs (not classified as lncRNAs) that have exons with transposable elements. Three stars (***) indicate significant differences based on Fisher’s Exact Test for Count Data, p-value < 0.001. (**E**) RT-PCR with specific primers on three genic lncRNAs of distinct types (**F**), and RNA isolated from developing grain (10 days post anthesis (dpa)) and flag leaves. CDC: reference gene (the cell division control protein). (**F**) Types of genic lncRNAs and the corresponding wheat genes.

Total RNA was isolated from whole spikes of hexaploid triticale (AABBRR) cv. L8665 at 10 days post anthesis (dpa). This RNA was used for direct RNA sequencing by MinION (Oxford Nanopore). In total, 1,100,000 direct RNA sequencing (DRS) reads with N50~1.1 kb were obtained (Figure 1). To assemble the transcripts, reads were mapped to the genome sequence created artificially by combining the A and B genome sequences of the wheat chromosome-level assembly and rye draft genome contigs. Overall, 82,785 transcripts from 74,904 loci were assembled with 47,378 and 26,169 loci located in genomes A/B (AB lncRNAs) and R. A total of 36,490 transcripts had >5 mapped DRS reads, representing a set of transcripts with high confidence that was used for further analysis.

To estimate the triticale seed development phase used for Nanopore sequencing, we determined the expression values (reads per million mapped reads (RPM)) of the key genes involved in starch biosynthesis (expression started at Phase 1), the genes of storage proteins (high molecular weight glutenins and gliadins), and those of the *wbm* protein (Table 3), which are expressed during the grain filling stage. We identified the expression of starch biosynthesis genes, while no genes of storage proteins or the *wbm* protein were expressed. This suggests that the seeds used for Nanopore sequencing were in the early stages of seed development (before 14 dpa).

Thus, using direct Nanopore RNA sequencing, we assembled a high-confidence transcriptome of triticale seed at the early development stage and detected the expression of key genes known to be involved in the biological process (starch biosynthesis) occurring at this stage.

### 3.2. Long Non-Coding RNA Prediction

The assembled high-confidence set of transcripts was used for long non-coding RNA (lncRNA) prediction. The following criteria were applied to distinguish lncRNAs from protein-coding transcripts: (1) transcript length of >200 bp; (2) transcripts with an ORF length of <300 bp; (3) transcripts classified as non-coding by three tools including LncFinder [57], PLEK [58], and CNCI [59]; and (4) transcripts with no similarity to any Pfam domains. Using these criteria, we identified 796 triticale lncRNAs (Supplementary Files S1 and S2) encoded by 780 loci in Genomes A (167 lncRNAs), B (212 lncRNAs), and R (410 lncRNAs) and in unanchored wheat scaffolds (seven lncRNAs) (Figure 1A). Most of the lncRNAs had lengths of <1000 bp (Figure 1B). LncRNAs (386 transcripts) encoded by the loci of Genomes A and B or unanchored wheat scaffolds (AB lncRNAs) were used for further analysis because of the significantly better annotation of these genomes compared with the R genome. Intersection of the genome position of the AB lncRNA loci with lncRNA and mRNA loci previously annotated in the A or B wheat genomes showed that 281 (73%) of the triticale AB lncRNAs are located in the previously unannotated genomic regions (Figure 1C). This number is significantly higher (Fisher’s Exact Test, *p*-value < 2.2 × 10^−16^) than that for non-lncRNA AB transcripts (10%, 2523), pointing to the underexplored nature of lncRNA loci. Moreover, only 13% (106) of the AB lncRNAs in our dataset were previously known.

LncRNAs are frequently associated with transposons. We found that 111 (29%) AB lncRNAs had exons that had transposon sequences with a length of >50 bp (Figure 1D). This was significantly more than that expected by chance (10%, Fisher’s exact test, *p*-value < 2.2 × 10^−16^). Of the TE-related lncRNAs, 61, 47, and 3 lncRNAs had exons with similarity to Class I, Class II, and unclassified TEs, respectively.

The AB lncRNAs were classified regarding the position of the annotated wheat protein-coding genes, resulting in 17 genic lncRNAs. Gene ontology analysis revealed that the genes overlapping with genic lncRNAs were significantly enriched (FDR < 0.01) in several Gene Ontology categories including “vesicle-mediated transport”, “lipid modification”, “ATPase activity”, and “hydrolase activity”. The genic lncRNAs were of different types, with Intronic-antisense (two lncRNAs, Figure 1F (top)), exonic-anti-sense (five lncRNAs, Figure 1F (middle)), and exonic-sense (nine lncRNAs, Figure 1F (bottom)) transcripts being the most common types. In addition, exonic (sense and anti-sense) lncRNAs were found. The expression levels of three types of genic lncRNA (depicted in Figure 1F) belonged to the distinct types estimated in developing grain (10 dpa) and flag leaves (Figure 1E). The RT-PCR results suggested that two lncRNAs (lnc001 and lnc003) were expressed in both samples, while the expression of one type of lncRNA (lnc002) only occurred in developing grain (10 dpa).

Altogether, we identified hundreds of previously unknown genic and intergenic lncRNAs of triticale and showed that they frequently possess the remnants of Class I and Class II transposable elements.

### 3.3. AB lncRNAs Are Prone to Tissue-Specific Expression

To find lncRNAs with possible specific roles during seed development, we used wheat RNAseq data to estimate lncRNA expression in several seed developmental stages, leaf tissues, and pistils of wheat.

**Figure 2:**
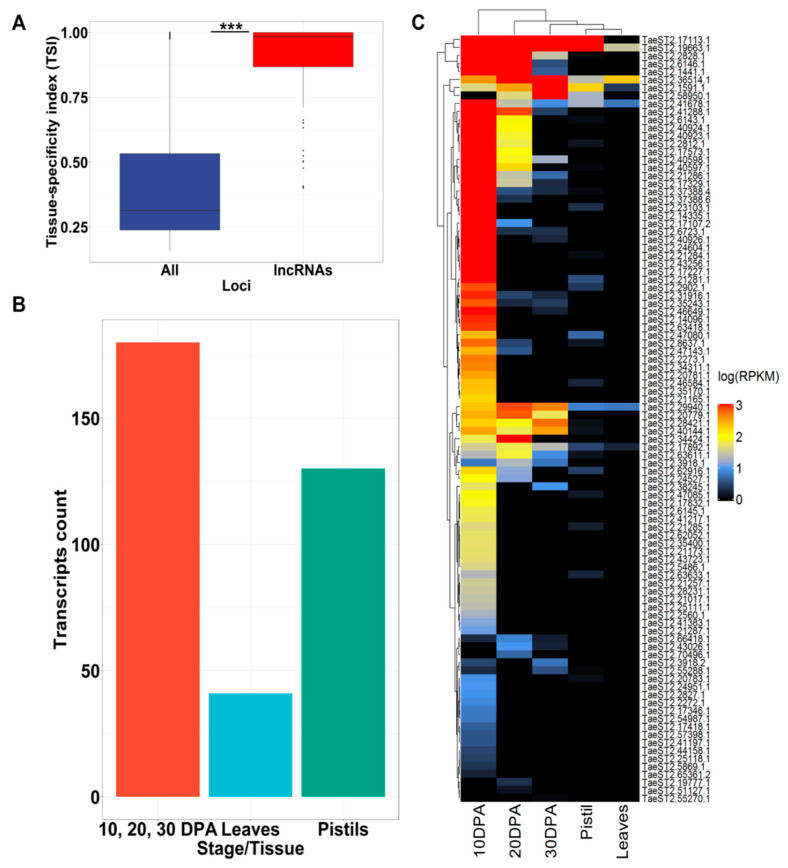
Expression patterns of triticale AB lncRNAs based on wheat RNAseq data. (**A**) Boxplot of the tissue-specificity index (TSI) for lncRNAs and all AB high-confidence transcripts assembled from direct RNA sequencing (DRS) reads (TSI values close to 1 represent high tissue specificity). Stars indicate significant differences estimated by the Wilcoxon rank sum test with continuity correction (*p*-value < 2.2 × 10^−16^). (**B**) Bar plot showing the number of AB lncRNAs with the maximum expression (reads per kilobases per million reads, RPKM) level at a specific stage of wheat development (10, 20, and 30 days post anthesis (dpa)) or tissue (leaves and pistils). (**C**) Heatmap of expression values (log(RPKM)) of AB lncRNAs with >90% of RPKM values based on wheat RNAseq data (10, 20, and 30 dpa; pistils and leaves).

A non-zero expression value in at least one condition was obtained for 351 AB lncRNAs. To estimate any biases in AB lncRNA expression compared with all high-confidence transcripts assembled from DRS reads, we calculated the tissue-specificity index (TSI). The results showed that the TSI was significantly (according to the Wilcoxon rank sum test with continuity correction, *p*-value < 2.2 × 10^−16^) higher for lncRNAs, suggesting high tissue specificity of lncRNA expression (Figure 2A). We further found that 46% of the lncRNAs had their highest level of expression at 10, 20 or 30 dpa, with 107 lncRNAs having their maximum expression level at 10 dpa; this is in accordance with the type of our triticale sample (10 dpa) used for RNA isolation (Figure 2B). Moreover, we identified 95 AB lncRNAs for which >90% of the sum of RPKM (reads per kilobases per million reads) values across all the samples accounted for the 10, 20, and 30 DPA stages (Figure 2C).

Thus, the expression pattern of the identified lncRNAs was found to be tissue-specific, with almost half of the lncRNAs demonstrating the maximum expression level during seed development.

### 3.4. Retrotransposon-Related Transcripts Encoding GAG Proteins Are Expressed during Early Seed Development in Triticale

**Figure 3:**
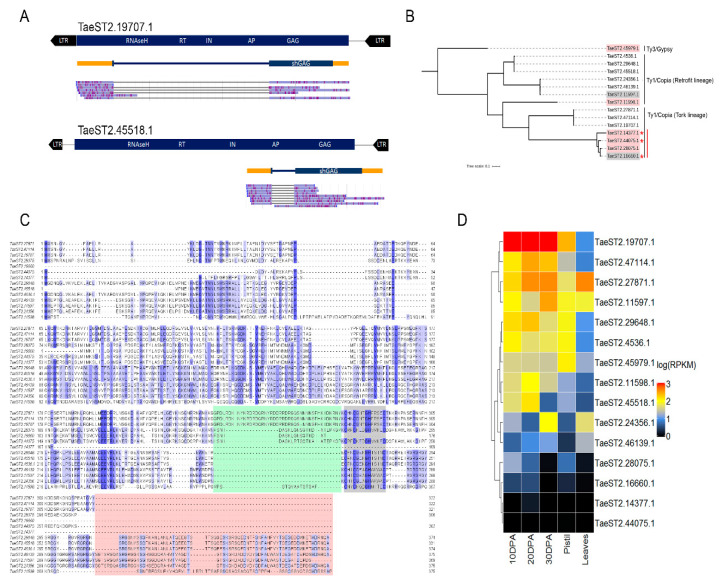
Identification, phylogenetic analysis, and expression pattern of RTE-related transcripts. (**A**) Full-length LTR retrotransposons of Tork (TaeST2.19707.1) and Retrofit (TaeST2.45518.1) lineages expressing a short isoform encoding the GAG protein (shGAG). The dark blue rectangles show ORFs encoding all retrotransposon proteins (genome scheme) and the GAG protein (isoform scheme). Orange color indicates untranslated regions (UTRs). The Nanopore direct RNA read alignment on the LTR retrotransposon genome sequence is also shown. (**B**) Neighbor-joining phylogenetic tree built from 15 GAG proteins. Red and gray highlight the GAG proteins expressed from RTEs without one or more encoded proteins and from loci without predicted RTEs, respectively. Red stars indicate GAG proteins with <300 aa. The vertical red line indicates a group of GAGs encoded by truncated retrotransposons possessing only GAG ORFs. (**C**) Multiple alignment of Ty1/Copia GAG proteins. Green and red highlight variable regions between the Tork and Retrofit regions. Gray shows the RNA-binding motif (CX_2_CX_4_HX_4_C, where X is any amino acid). (**D**) Heatmap of the log(RPKM) expression of isoforms encoding GAG proteins in wheat leaves and pistils, and during seed development.

Early embryonic and endosperm development may be accompanied by the activation of transposable element (TE) activity [22]. Therefore, we analyzed the assembled transcripts for the presence of open reading frames encoding TE-related proteins. We focused on the transcripts of Genomes A/B because of the high quality of wheat genome assembly and annotation compared with the rye genome. No transcripts corresponding to DNA transposons were detected. However, we found 20 transcripts (RTE-RNAs) carrying a single ORF with similarities to distinct proteins of LTR retrotransposons. Surprisingly, we found no transcripts encoding for the full set of RTE proteins (GAG and POL). To check whether any RTE-RNAs were encoded by LTR retrotransposons with detectable LTR sequences (RTEs), we predicted RTEs in Genomes A and B. The results showed that 5 and 10 RTE-RNAs (15) were transcribed from full-length (potentially autonomous) or non-complete (one or more RTE domains were not detected while both LTRs were present) RTEs, respectively. Thus, almost 25% of the RTE-RNAs were found to be transcribed from potentially autonomous RTEs. For five RTE-RNAs, no associated RTEs were predicted, but 75% (15) of the RTE-RNAs were found to carry an ORF encoding a single GAG protein (GAG-RNAs). Of those, eight (53%) and five (33%) GAG-RNAs were encoded by full-length (Figure 3A) and non-complete copies of LTR retrotransposons, respectively. For two (14%) GAG-RNAs, no corresponding RTEs were identified (Table 4). It should be noted that four GAG-RNA genes were found to be located in the introns of three annotated protein-coding genes in the sense or anti-sense orientation, including TraesCS2B02G261900 (sense and anti-sense), TraesCS5A02G298800 (anti-sense), and TraesCS1B02G222500 (sense). Two GAG-RNA genes (TaeST2.11597.1 and TaeST2.11598.1) were found to be located in the introns of the same gene (TraesCS2B02G261900).

We further focused on GAG-encoding RTE-RNAs (GAG-RNAs) as the most represented group. Oxford Nanopore RNA sequencing provides a unique opportunity to analyze the exon–intron structure of GAG-RNA-encoded loci and predict the deduced full GAG protein sequences. Classification of the deduced GAG proteins showed that 85% (14) and 15% (1) of them belong to Ty1/Copia and Ty3/Gypsy elements, respectively. Based on the information from the transcript assembly, we grouped all GAG-RNAs into three categories based on the number of introns they possessed: (1) a single intron, (2) two introns, and (3) no introns. The vast majority (86%, 13) of GAG-RNAs were found to carry a single intron (Figure 3A), while one GAG-RNA from Ty3/Gypsy had no introns, showing that the exon–intron structure may differ between the two LTR retrotransposon superfamilies. To understand the functional role of splicing in the generation of GAG-RNA transcripts, we also predicted ORFs for unspliced RNA variants corresponding to the regions between two LTRs. We observed that unspliced transcripts expressed from the whole RTEs had significantly longer ORFs, resulting in GAG proteins being fused with other RTE proteins. Additionally, for two GAG-RNA encoding RTEs (TaeST2.19707.1 and TaeST2.45518.1, Figure 3A), only one very long ORF (>4000 bp) was predicted, while other RTEs were found to have two or three ORFs encoding distinct proteins. Thus, we showed that the splicing of the GAG-RNA isoform is critical to ensure the production of the entire GAG protein.

We next performed a comparison and phylogenetic analysis of the amino acid sequences of the 15 GAG proteins. The multiple alignment revealed significant differences between one Ty3/Gypsy GAG (TaeST2.45979, GAG length 514 aa) and the Ty1/Copia GAG proteins. We compared the Ty1/Copia GAG sequences and found that Ty1/Copia GAGs originated from two RTE lineages, Tork (seven sequences) and Retrofit (seven sequences). Phylogenetic tree analysis revealed three and two groups of highly similar (up to 99%) GAG proteins in the Retrofit and Tork lineages, respectively (Figure 3B). In addition, we detected pronounced sequence divergence between the GAG proteins of Tork and Retrofit lineages. The GAG protein of the Retrofit lineage has a ~50 aa-specific C-end which is not found in the Tork GAG sequences (Figure 3C). In turn, Tork GAGs have specific amino acid sequences before the RNA-binding motif (RBM) site. These differences are also reflected in the phylogenetic tree’s topology, where the branches corresponding to the Tork and Retrofit Ty1/Copia lineages are readily distinguishable (Figure 3B).

In addition, we noticed that the divergence of groups in a single lineage correlated well with the completeness of the RTEs expressing corresponding GAG-RNAs; the most diverged group of GAG was the one with the Tork lineage and encoded by truncated retrotransposons (Figure 3B) that had a single ORF-encoding GAG.

We then analyzed the GAG protein sequences in more detail. In particular, we estimated the presence of the RNA-binding motif (RBM)(CX_2_CX_4_HX_4_C, where X is any amino acids), a specific part of GAG proteins, which is responsible for GAG–RNA interactions. We found that RBM could be identified in all except three GAG proteins, including one Ty3/Gypsy (TaeST2.45979.1) and two Ty1/Copia elements (TaeST2.14377.1, TaeST2.16660.1) of the Tork lineage. The Ty1/Copia GAG-RNAs without the CX2CX3GHX4X motif (TaeST2_14377 and TAeST_16660) are truncated GAG proteins with smaller protein lengths (175 aa and 182 aa vs >300 aa for the full-length Ty1/GAG protein) and probably very limited functionality. Altogether, the results of the phylogenetic analysis suggest that a divergent set of GAG proteins, including truncated GAGs with no RNA-binding capacity, is expressed during triticale seed development.

We then estimated the expression patterns of the GAG-RNAs in several seed developmental stages and in leaf tissues and pistils of wheat. Nine of the 15 GAG-RNA loci had a specific expression pattern with maximum expression levels at early developmental stages or in pistils. For two GAG-RNA loci (TaeST2.14377.1 and TaeST2.44075.1), the expression level was too low, indicating a possible triticale-specific expression pattern (Figure 3D). Thus, the expression data showed that most of the identified triticale GAG-RNAs were also expressed during wheat seed development, and some RTEs expressed genomic RNA (gRNA) as well as a short isoform (shGAG) carrying ORF for GAG protein.

Overall, our results suggested that tens of transcripts encoded by full-length and truncated LTR retrotransposon copies are expressed at early stages of triticale seed development. Three-fourths of these RNAs carry ORFs for encoding a set of GAG proteins of variable length and phylogenetic diversity.

### 3.5. A Full-Length Ty1/Copia LTR Retrotransposon Is Active in Triticale Seeds

**Figure 4:**
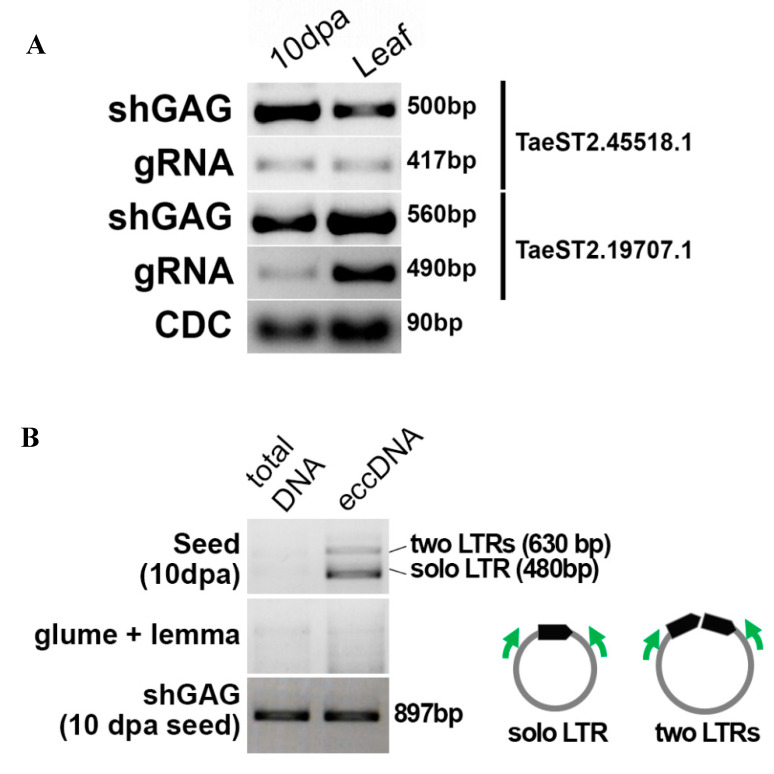
Expression and extrachromosomal circular DNA (eccDNA) formation. (**A**) RT-PCR detection of the shGAG (a short isoform carrying ORFs for GAG protein) and gRNA isoforms of RTE3B and *MIG* full-length RTEs. CDC: reference gene (the cell division control protein). (**B**) Inverted PCR with genomic DNA and eccDNA-enriched fractions obtained from developing seeds (10 dpa) and glume and lemma triticale tissues. The positions of the primers on eccDNA are shown in the small representation in the right. PCR with shGAG primers with total and eccDNA-enriched DNA was used as a control.

To transpose in the genome, RTEs need to express the full-length genomic RNA (gRNA). Although we did not detect gRNA for the RTEs expressing TaeST2.19707.1 (RTE3B, location in wheat genome: 3B:555,156,557…555,163,131 (6.58 kb)) and TaeST2.45518.1 (named ‘*MIG*’, location in wheat genome: 7B:312,336,869…312,341,902 (5 kb)) shGAG RNAs by Nanopore sequencing (Figure 3A), we performed RT-PCR with primer pairs designed to detect (a) shGAG isoforms and (b) gRNA isoforms. The RT-PCR analysis resulted in detection of the expression levels of shGAG and gRNAs in developing triticale seeds (10 dpa) and flag leaves, although the gRNA expression level was lower (Figure 4A). Next, we assessed whether the RTEs were capable of transposing. To answer this question, we determined the generation of the extrachromosomal circular DNA (eccDNA) by these RTEs using inverted PCR. EccDNAs are byproducts of RTE activity in plants [71]. We first determined that inverted PCR with the designed primers did not produce PCR products with genomic DNA. Unfortunately, the primers on RTE3B produced PCR fragments with genomic DNA; therefore, the activity of RTE3B could not be assessed by inverted PCR. We continued eccDNA detection only for *MIG* (Supplementary File S3). For this, we enriched the eccDNA fraction by exonuclease treatment of total genomic DNA using an enzyme that specifically cut linear DNA while leaving circular DNA molecules intact. The product was then amplified by rolling circle amplification, and inverted PCR was carried out. We enriched eccDNA in genomic DNA isolated from developing grain at 10 dpa, as well as glume and lemma tissue. The specific products were detected only for eccDNA isolated from developing grain, and no products were obtained with eccDNA of glume and lemma tissues. Thus, our results showed that RTE *MIG* expresses both shGAG RNA and gRNA isoforms and has transposition activity.

Here, we provide experimental evidence suggesting that some detected RTE-RNAs originate from autonomous LTR retrotransposons with ongoing transposition activity in triticale at early stages of seed development.

## 4. Discussion

### 4.1. A Set of Intergenic lncRNAs Detected by Nanopore Sequencing Is Expressed during the Early Stage of Triticale Seed Development

Wheat seed development is a dynamic multistage process that involves significant changes in the transcriptome landscape. Here, we uncovered the lncRNA transcriptome of triticale seed at 10 days post anthesis (dpa), corresponding to the second stage of grain development, known as embryo differentiation (7–14 dpa) [34]. This stage is characterized by the formation of Type A starch granules and the expression of genes involved in starch biogenesis [36]. In turn, the genes encoding for the main storage proteins (the high molecular weight (HMW) glutenins and gliadin [36]) and the genes involved in storage protein biogenesis (e.g., wbm [75]) are expressed during the mid-development stage (14–21 dpa). In agreement with this, we detected the expression levels of the starch metabolism genes (Table 3), while expression of the storage protein genes (HMW glutenins and gliadins) and the wbm gene (found recently in the analyzed line (L8665) [76]) was not detected by Nanopore read analysis. These results prove that the analyzed triticale transcriptome corresponds to the early stage of grain development.

Our analysis showed that hundreds (798) of lncRNAs were expressed during this stage. Surprisingly, we found that 87% of the A and B lncRNAs were expressed from as yet unannotated regions of Genomes A and B. LncRNAs are often underrepresented in plant genome annotation. For example, 8009 lncRNAs were previously identified in the intergenic regions of barley [77], and 1760 unannotated lncRNAs were identified in foxtail millet [78]. The annotation of the lncRNAs expressed from the intergenic space can be challenging because of the biological properties of lncRNAs, including their high tissue- and stage-specificity. Indeed, we identified a high tissue-specificity index for triticale lncRNAs, suggesting that a large number of lncRNAs are expressed during a narrow time window. Additionally, lncRNAs often possess exons containing transposon-similar multicopy sequences that make the transcript assembly difficult because of unambiguity in short RNAseq read mapping. Here, we found that almost 30% of the triticale lncRNAs possessed exons with similarity to TEs. This is in accordance with previous reports on rice [79], where 73% lncRNAs were found to overlap with different TEs. Furthermore, 9.18% of sunflower lncRNAs have TE-related exons [80]. In these terms, RNAseq-based lncRNA identification can underestimate the number of lncRNAs in a cell or lead to transcript misassembly, because short reads from the repeat regions are often mismapped or discarded from the analysis [25]. However, notably, the study of plant lncRNAs and transposon-derived transcripts has mostly been limited by short read RNAseq data. Here, to escape lncRNA identification biases because of the short length of the RNAseq reads, we applied Nanopore direct RNA sequencing. This approach allowed us to precisely determine the transcribed regions in a complex wheat genome. In addition, Nanopore direct RNA sequencing is strand-specific and can be used to identify natural anti-sense lncRNAs. The application of third-generation sequencing could help to illuminate “the dark side” of developing seed transcriptomes involving lncRNAs and transposon-derived loci, thereby overcoming the obstacles in lncRNA discovery by short RNAseq reads. It will be especially useful for crop plants where lncRNA loci can be included into genome-wide association studies (GWAS) to determine which of them can influence key agronomical traits. Notably, lncRNA loci have not been well used for GWAS analysis so far [81]. We believe that the triticale lncRNAs identified in this study could act as valuable new targets for marker-assisted selection.

### 4.2. Transcripts Encoding Diverse GAG Proteins Are Expressed during the Early Stage of Seed Development

Seed development is accompanied by epigenetic relaxation, which may trigger retrotransposon (RTE) activity [21,22,23]. Here, we identified 20 transcripts with similarities to RTE proteins. Interestingly, we found that 75% of the RTE-related transcripts carry ORFs encoding GAG proteins, the main component of the RTE virus-like particle. It is known that during their lifecycle, RTEs have to produce significantly more GAG proteins that other RTE proteins. To ensure that there are excess GAG proteins compared with other RTE proteins, an *Arabidopsis* RTE called *EVD* encodes a special isoform (shGAG) encoding the GAG protein [82]. Because almost no systematic studies of RTE transcripts have been carried out at the single isoform level in crop plants, it was not previously clear whether shGAG transcript production is a common pattern for plant species. In this study, we detected isoform production for several full-length triticale RTEs and showed their transcription in wheat, implying that shGAG is a very common pattern for plants. Moreover, we showed that one of the full-length RTEs, TaeST2.45518.1, is capable of producing extrachromosomal circular DNAs (eccDNA), which are a byproduct of RTE activity and have been used to isolate transpositionally active RTEs [74]. Whether this RTE is active in the triticale embryo and can produce copies that are transmitted to the next generation or whether it is active in other tissues of developing grains (e.g., endosperm) warrants further investigation.

Based on the knowledge of splicing patterns of GAG-encoding isoforms, we were able to predict the GAG protein sequence and analyze it in more detail. Our results point to the existence of a divergent group of GAG proteins expressing during triticale and wheat seed development. These GAG proteins and the RTEs encoding them have several distinct features: (1) the RTEs encoding these GAG proteins are non-autonomous elements and possess no similarity to POL proteins; (2) the lengths of most of these GAG proteins (169–270 aa) are less than that of the conventional GAG protein (>300 aa); and (3) half of these GAG proteins lack the RNA-binding motif and cannot interact with RTE RNAs. The elements encoding GAG-RNA consist of two LTRs and the internal part is similar to the GAG protein. This structure makes these elements very similar to the previously described TR-GAG elements (terminal repeat with GAG domain) ([83]) found in many angiosperm species. However, the TR-GAG elements described in the current paper are classified as Ty1/Copia, while GAG proteins of previously identified TR-GAG elements are similar to both Ty1/Copia and the Ty3/Gypsy superfamily. While further functional and evolutionary studies are required, we suggest that these GAG loci are intermediate products of RTE diversification or “domestication”. The “domestication” of GAG proteins has been documented in animals and insects [84,85,86,87]. Because of the short lengths of these GAG proteins, it can be also suggested that they may be involved in control of the activity of functional RTEs via incorporation into their virus-like particles. The mechanism of copy number control of RTEs via virus-like particles (VLP) misassembly, which is caused by a truncated GAG form, known as dominant-negative factor, has been well described for yeast [88,89]. No such examples have been described in plants. In the future, it will be intriguing to check whether TR-GAG proteins are capable of interacting with normal GAG proteins of full-length RTEs, which, as we showed here, are expressed in the same developmental stage.

Previously, Nanopore long-read sequencing of transcriptomes was used to annotate expressed transposons in *Arabidopsis* mutants lacking key systems of TE suppression, resulting in elevated expression of transposons [26]. The authors detected the expression of almost 1300 TEs. However, elucidation of transposon expression a “wild-type” genetic background would provide a unique opportunity to trace natural evolutionary forces shaping plant retrotranscriptome and to more deeply understand the features of host–transposon interactions. Recent studies on maize and sunflower [26,90] and our current results point to the great advantage of Nanopore RNA sequencing to decipher RTE expression in crop plants, even those with complex genomes such as triticale. Together with the growing number of publicly available long-read RNA sequencing datasets, this opens a broad avenue for studies of transposon expression in plants on the isoform-based level.

## 5. Conclusions

Here, using Nanopore direct RNA sequencing, we identified hundreds of previously unknown lncRNAs and LTR retrotransposon-derived transcripts that are expressed in the early stages of triticale and wheat seed development. We showed that triticale lncRNAs often possess similar sequences to transposons and their expression has high stage and tissue specificity, with half of the lncRNAs having the highest expression level at 10–30 days post anthesis in wheat. In addition, we found that most of the detected retrotransposon-related RNAs have a single intron, carry ORFs encoding for a divergent set of GAG proteins, and are encoded by potentially autonomous and non-autonomous retrotransposons. Of these, we identified one Ty1/Copia LTR retrotransposon that produces extrachromosomal circular DNA, and we suggest that it has transposition activity in developing triticale seeds. Finally, this study identified a unique set of lncRNAs and LTR retrotransposons expressed in the early stages of seed development, which we believe will be useful for further exploration of their functional potential and the association with phenotypic variation in triticale and wheat.

## Figures and Tables

**Table 1:** Primers used for RT-PCR.

Target	Primer Sequences	Amplicon Size
Lnc001	lncTR001/F: AGGTTGCAAGTCTCTTGCTCTTGA lncTR001/R: TCATGCCCGCTAAGAATTACAGTGT	RNA/DNA = 500 bp/~1100 bp
Lnc002	lncTR002/F: TGGGTTGTGACTTGTGATACGCA lncTR002/R: CGGTTAGGGCTGGGCTGAATG	RNA/DNA = 300 bp/300 bp
Lnc003	lncTR003/F: ACAGTATGAAGCTAGCCGGCTTG lncTR003/R: TATCCTGTCGTCCTCTCGTCTCG	RNA/DNA = 303 bp/303 bp
CDC (the cell division control protein), Ta54227 [47]	CDC/F: GCCTGGTAGTCGCAGGAGAT CDC/R: ATGTCTGGCCTGTTGGTAGC	RNA/DNA = 76 bp/76 bp
gRNA TaeST2.19707.1	gRNATae_19/F: ATTACACCCCCAAACCGCCAAAT gRNATae_19/R:TGGGGAATTTTCCACACCCACTT	RNA/DNA = 490 bp/490 bp
shGAG TaeST2.19707.1	shGAGTae_19/F: TTGATTGCCGCCTGGTTATCACA shGAGTae_19/R: AGTGGGAATCGGAGGAACTGGAA	RNA/DNA = 560 bp/3200 bp
gRNA TaeST2.45518.1	gRNATae_19/F: ATTACACCCCCAAACCGCCAAAT gRNATae_19/R: TGGGGAATTTTCCACACCCACTT	RNA/DNA = 417 bp/417 bp
shGAG TaeST2.45518.1	shGAGTae_45/F: GCTTACTCTTGTCTACTCCACGCA shGAGTae_45/R: GGACTGGAGAAGCGAATGCATCT	RNA/DNA = 500 bp/897 bp

**Table 2:** Publicly available RNAseq data used in this study.

SRA Accession	Number of Reads	Development Stage/Organs	Reference
ERR392055	26,791,465	10 dpa/seed	[39]
ERR392076	29,714,230	20 dpa/seed	[39]
ERR392069	31,433,795	30 dpa/seed	[39]
SRR10522394	39,611,224	Leaves	[70]
SRR1175868	64,825,850	Pistils	[71]

**Table 3:** Expression of the key genes of the starch biosynthesis pathway and wheat storage proteins.

Wheat Gene ID	Gene Expression, +/−	Reads per Million (RPM)	Gene Annotation	Genomic Coordinates
TraesCS4A02G418200	+	76	GBSS/Starch synthase, chloroplastic/amyloplastic	4A:688,097,145–688,100,962
TraesCS4B02G029700	+	7	(BGC1) Flo6/5′-AMP-activated protein kinase subunit beta-2 (PTST)	4B:21,937,120–21,944,075
TraesCS4A02G284000	+	3		4A:590,660,989–590,667,561
TraesCS7B02G139700	+	6	ISA	7B:175,999,323–176,007,332
TraesCS7A02G251400	+	15		7A:235,460,629–235,468,417
TraesCS6A02G048900	−	0	α/β-gliadins	6A:24,921,651–24,922,607
TraesCS6A02G049200	−	0		6A:25,203,493–25,204,413
TraesCS6A02G049100	−	0		6A:25,107,550–25,108,401
TraesCS6A02G049600	−	0		6A:25,472,841–25,473,704
TraesCS1A02G007400	−	0	γ-gliadin-A3	1A:4,033,339–4,034,196
TraesCS7A02G531903	−	0	wbm	7A:710,471,331–710,471,679
TraesCS1A02G317311	−	0	HMW Glu-1Ax	1A:508,723,999–508,726,319
TraesCS1B02G329711	−	0	HMW Glu-1Bx	1B:555,765,127–555,766,152

**Table 4:** Number of retrotransposon-related transcripts (RTE-RNAs) in different groups based on completeness of associated LTR retrotransposons (RTEs) and the type of predicted RTE proteins encoded by open reading frames (ORFs) (reverse transcriptase, RT; aspartic protease, AP; RNAse H).

	RTE Group			
Encoded proteins	Full-length RTEs (all domains are detectable)	Non-complete RTEs (one or more canonical protein domains are not detectable)	No associated RTEs identified	**Total number of RTE-RNAs**
GAG	8	5	2	**15**
Other RTE proteins (AP, RT, RNAse H)	1	1	3	**5**
**Total number of RTE-RNAs**	**9**	**6**	**5**	**20**

## Data Availability

Nanopore data produced for this study are available in Sequence Read Archive (SRA) NCBI under Bioproject Accession PRJNA683988.

## Supplementary Materials

The following are available online at https://www.mdpi.com/2223-7747/9/12/1794/s1, Supplementary File S1: Genomic location and overlapped genes for 976 Triticale IncRNAs, Supplementary file S2. Fasta sequences of 796 lncRNAs, Supplementary file S3. Sequence of RTE7B LTR retrotransposon that is expressed and produce eccDNA in triticale developing seed at 10 dpa.
