# Supplementary material for: Nanopore RNA Sequencing Revealed Long Non-Coding and LTR Retrotransposon-Related RNAs Expressed at Early Stages of Triticale SEED Development
Source: Plants (Basel). 2020 Dec 17;9(12):1794. doi: 10.3390/plants9121794 (PMC7765848; doi:10.3390/plants9121794)
Supplement: Supplementary file 1 [file plants-09-01794-s001.zip › Supplementary file S2.docx]

**Supplementary file S2.** Fasta sequences of 796 lncRNAs (no introns included).

>TaeST2.333.1

ttaaatctcattgaaatatattgcaaccgactcccgcagcaacgcgcggggtatcatctagtttattcaatttaccaacagaagtaggcgcccctgtcactacaacaaaaaattataaccacaaaaacaaaccaaaaacagggtagctcaagatatatcaccagaagtgctagtttcctgtcactacaataaaatatagcatagattttactaccgaaaacagggtacaacataaactcttctgctgacataagaaagttagccaagtccagcaagacaggtagggaagctatggatcagctgctgcagaaggaccatcgccaccttgatgagcttcagcttgtgcccttttttgtttgtcgccgttcagcgcatgacaaggatcaagcatacaagctttcatctcactaacttctgaagataagactgagaagagaacttccattgtgtaaagtcgtttctcaattgcctcgaatcctttcacaactttctcgtgcaacccctctatcagttttgaaagttgagcattgctggcaactgttgaatcaggattaactggttcaggattaactggttgcggtagctgatgagaataggacatgggctgctggtgctgcacctctataacttcatgctcagctgaggcagcacgttgttcacgattcaagcttgcaaggtgcagggagtacttcttgaggcagcatttggtatcaatgtttgtaaggtcacacttcagatggcgaagaagacaaaatataagaggtgcatatggcagtgcatgatttacagtgtcaatggcctcacacatctcactgaggaggaaatgagcaatgtcaaactgctttccgtggtaaatggcataggcaagcttgagaagcatggtggtgcatccacctcgatttcctgatttaggaaaaattgtatgtcgatttatcctttggatgagatcaacttcagttttaagatccctaactagaccaatgtcgggattaggtactcgaaagagagaataaacatcaaaatcatcccatgtgtgcaaaacaaggctgtcagctctaggctcaattagcaaggccttttcaagatcttccttggtagcaaaacaatgctgtgagcctatcatgaaatgcatttccctgcgatcaggatcaacataaagggtgccataaaactgtctcacgacctcttcattccaaacccaagatgaattgttgctggttagacctttgagaccaagatcctcatacttagtggacaaatcaaaagggcatgtttcagcatcagctatatctgaccactccacaaatctatgcttaaacactttcttctcatgacagaactgatcatatagctctttctgtctgttgtcccagaaattgcagtcaggagaggtagatctagcagcaacataagggttacgttgcttctgcctcttaagttcaggatacctcaaatggcccgatgtcctcctcttcctcagctccttcctttccctatccatggctttatgcct

>TaeST2.363.1

tttacagccttaaaaaaagtcatgtttttacagactccctttccttgtaagcacgtgcaattggtcatggcaaaatgtaaagagattgaaccaaaatctgcaaacacaatcccctcaaaagaaaactgcaagcacaaagtgaacaaaatcatcaacacttttagttacaggctgaagagcacatgtacagcagcaacatgtaaaaggagaggaggaagcaaagccatgtgaattgacttcggagatgtttggcatcatggaagtatcctgaaataccatgaggctcatgtccccaagacaacagatgacatgctcaggccctcagcaaagagacacatgaatattggcacctacaaacaaagttgccctctccagcgattcaaccatatcggaatggggagtacacagcgtgatagcaaaagccagaaacactattttgatttgcactggtgcccctatgatatactactagttcctatatatctttcctgggtcggtgaacttgagctgctgactgctggtgcgctgatcttcatgttcctcgggacttttgagatgtgagcgaagggcaggcctcatcggagaagaaaaacaacacgcatcatgcaaaacaacatgtgaagatttattaagtatggtgtgtcaaaaaccaatctctgtagtcagataatcgtaataacacgcaaattagtgtctacaaataatgtcactcttcctatgatgacataattaatagaactgaacatatttaaaataagactctccaataattctctatatacgaatattttagagcgagaacatatgtccgcatgaaaaatggagtaagtagcgagagctattttgaaaaatctggaggtgtaccattagccatcattactatagctagtatttgggttagtaataatcaaatagagcaaacaatgattggttgtgcttgctcaatgctatgggttgtggggttacaaaaggtgctagtttgaaagaaaaaaaaattattgctcacctattatgatctgccttctcatctgaaggcttgtttattatctaaacatatttcccaatagtccaatggctcatgaagtggaagaattattatagttgtatggtgcggtggggtgcaacacgttcagtcgtccgctgcggctgctggactgctctgccggtcgagcagccctgtcaacatgtggcgatcgcttgtttatgatctctctcaaacttacagaaatatgcttatatgtaataaactagtcttcagatgagaagcaaatcataacaattggagcaacaatatcccttttattttcttcaaaatagcatcttctgtaacctcatgaggcataaaattgagcaaacaaaaccaataatttttttgctgcatttggtattattactaaccaaaatagtagctatagtaataatggctatgatacactgccacatttcttcgaaatagctctcgatattttctccaattcacgtgaacatatattctcatcgtgatatatcttctatagaaaattatttgagagtcttatttgaaagaggtttctcatgaactatgtatcacaaaaaaagtgatatgcttcaaagatactaattttgcatct

>TaeST2.751.1

agtttccaggcgctacagaaccctcgaggcccccctccaggatttgcccgactcacgccgccgcctgacccgaccgccgactcctccctaagacgccgcgccgcctcgacgccggcgatctcctccggcatactggacttctccccggtgattggagcaagaagactgatgtgaaaagggagacatcgcctggcatgatcaaaaaggagattttgcgtgtagaagtagagctgatgaagtgaagtgtaccagctaattcctatgttggttctcagtcaccaaaagaagcaacaataaacaacctggatggataaatttctctcgggttctcaaagctagatgatcatgacaccacaagatggatacatgtgtctgcacgttgttgaaatgaactattgttgtcgtaatgaattgttgttgtcgaagatcacgttgtttgataaattatgattgctgagatttgagattcgaagtcacgctgttatgataaattatgttcttgctgtgatgagatattgatttcaaatcgtgcaaaattatgttgcttctaaa

>TaeST2.1007.1

cactccactcttctctgcctgattagcgattattttagcccacgcaccggcaccaacaccacttgccccatcgccgccaagctccactgctccgctcctcccctccgcctccctcccaagcctctcgtctccgacggatcaaggaaggaggccaggagcagcagcaagatcagcagctcgattgggcaggcaaggggcggatggagttcgtgctcatcatatccctgccgctcctcatcctgatcctcatcatcgccgtcgtcctctgcatcgtctgccgctagccacatgccgacgccaaagcgctcgtctctgctcggtctgctgtgactgtggtcgcaccatatgttgtgttaatcaatccaatattacatcatgactcggcatagaaaagaaaagaaaaaaggctcctctccaccaccatttctatttttctgttctgtccctgtggtgtgattagctggctatagctttactggctttcctgatggcaacaaaagcatcgatcccgtgcgtcgatgcgcaatgcatatgatatacttatgtggctgtaaaaaaagagaacaaattccgttcctgctctgattgattcggggaggttgaatacctggaagtgcatcttctt

>TaeST2.1434.1

tgagtagtacgcactcccatctcatctgacgatcttgaacaacctgcgcgcgcaggaatggcgaactactacatgaagccggccttccagttcctggtgcttttccttgttcttctcggatgcttcgcgtctcgcgctcagagtcaagggcatggaccggacatattctcatgtgagaggcagctgatgcggccatgcgacttgtgttactgctgcttgtatggcaacaataagggagtgtgctatgcaactgatgtggactgcaggctggcttgccctccgcctaaattcaccctaccatgaacctgaacattttcttgtaatgtctctcatgtagtacgtctgtacttcttaccccatactctgaagaataactggcagaatcaactgatgccatatgtgtgtgtgcttatctgtttgcactactttttctgtcgaaaaacaaa

>TaeST2.1441.1

cacctatataaacaccgtcaaagtgaactttgaaacacaccgcatattagcacaacaattgagcatagaagtcacttgttggcctcaccaaatggcaaccttcaagatgaatcttggtcccttctttttggtggctctcatggtactggctactattgtgtccccatgccatgcgaacaatgagatacatgcagatgcaataccactacctgaaagttgctatccaatgaagtttcctaaatgcactaacaaggcgtgctacaagtattgcataaaccctggaggctactgcaaagatgttgattattgttgttgccctatggaataaacaccactcaacccccgagatgaaacttttgggttgtcgtttttaacctatgaataaagaaagcttg

>TaeST2.1567.1

aacaaagccgccgcgttctcctctgcgacacgcaaccttttcccctcccccctatagactagaccagatccttgatatagagaagaacacgccccttcctcccaaccacaactacctccatctcggcaaggtgagcccgtgagtctctgtctaccatgattttttcctcctcgaaacccccgtgcgctctctggttcgcaggttcctcgtcgatcggcgggctgggcttgcggcgatcttggagcgagacgcgaggcggctgcatcttccgatctccccgtccggcgactcagatctgttacctatctgatggcccgcggcggatctgggggttcccggcggtctagagttgactctgcggcgatgaaggcttcttctccgacgtgcacgtgtggatcccggcttccccgcttcgcgcgacgacggctcttgtattgataattatccgatgatatctcgataattattagatctaagagatcagaggtaagagcatgtaacaagcgacgtgatcgtgccgagaggggaaaaggagtgaaaaaagagagatgtgacacctagaggcctttgccatgttgggtgcgctcgcatggcaggccgccaccgtaataaagatggttcttgtgtcccggggggagatcgcacgggggttattctgttttagccccggggtctccccccaccgtgtcctggtgtgctcctttgtctgcacctctcgtccgatccgatcttcctggcacaacgttctaccgtcgcgaatcgatcaaggtgctacaaaccaaaggtacaatcgataatctacagctgaagcatacataa

>TaeST2.1591.1

tatcactgaacttttctgttatttaattgcatcctggctgcgctaacagataaccaagcatctatgcttttctgttacgatcatatcaaaaatctgaaagaaaaacaaaaaataaacagaggagagaaagagggaagggaacaggacaagtgaaaacaacataacaagtctgatgatatctattttccccagatgtatcagttcatgctacagatggagatggacagcctaataatataccgataaaacatggcttacagtttcgacgagccctacaattcagtagtagaaattattttctgaaccgaagaaaaccagggaccattgcctggcctgctctctcccatgaacaaccgaataaccttcctcccagcaaccagagatccttggagactcctcagcctcaacagcacgggtagatggtgacggagcgccgcttcggcggggtgcagaggagcatgaacaccagggcgatgacggccgcgacgaggagccccaggaagctgaaccccgcctcctcctccgactcgctgctgcagcacaccttccccatgctagcctgagcagagaggttgcttcccggtgctgcctacgctggaggtcagtggtggtcagacgaagaagtgaggctcgcttcctagcagtggtacgtttcgttctttcttataccaccacggccatgatcgccgtgggtgtggcca

>TaeST2.1604.1

caagaatcggccatgcctctatataaacagcagaaggaaccagcaaagtagtacaagtctaaaccaaacggacataggagaagagaacactaaacaatttcattatggctctaatccggatcaacactggtgttctgtgcttgatggcacttttgatggtttcaaccactttattgtcgagtgaagcatcagggaggaagataggcaaggaagctaacattggcgtcgaggggcaccgtgaaccctacactccatgtacggatccttcattttgtaatgatatctgcaacagagatggttacggaggtgggacctgccgtgatggtcactgcgaatgcatcagataattgtagcaagaaaatgaccaagaataatgtttggaaataaagattcacttgggagcattatgaattgtttttctagcttgaataatttaattgaacattttta

>TaeST2.1622.1

aaaaggacataggagaagagaacactaaacaatttcgttatggctctaatccggatcaacactggtgttctgtgcttgatggcacttttgacggtttcaaccactttattgtcgagtgaagcgttaggaaggaagataggaaaggaagctaacattggtgtcgaggggcaccgtgaaccctacactccatgtacggatccttcattttgtaatgatatctgcaacagagatggttatggaggtgggacttgccgtggtggtcactgcgaatgcatcatataattgtagcaagaaaatgaccaagaataatgtttggaaataaatatccacttgggagcattatgaattgtttttctagctggaataatttaaatcccacttttttattttcctactctttgtacttgaacccgtatcatggccttt

>TaeST2.2272.1

ggttgtgcctctatataaacaacagaatgagaaactaaattgaccggaaatacagcgataggagaaaaagaatagtgtaccccctccccatggcgcttctcaagaacaccccaggggttttgtgcttggcgtcccttcttgtgatatctgccaccttcttatcatgtgatgcatcctacggggcgacagatggagaaggtttaaacaagttctgtgtgggttgggaccaaggctgcaaggatccagtgaatggaatccctagtcagtgccagatttactgcagagataacggttttgtcttagagaaaggcaaatgtgcaccggacaatggaaagcccaaccgcatgtgttgctgctataagtagacacttccgcgcgaatttgtggaagagtgcaaaacattttctgtatgagagatcttgaaaaataaattaagaaagcattgtttgatctatgtc

>TaeST2.2273.1

aacgataggaaaaaaagaatagtgtaccccctcctcatggcgcttctcaagaacatcccaggggttttgtgcttggcagcccttcttgtgatatccgccaccttcttatcatgtgatgcatcctacggggtcaaagatggagaaggttttaacaagttctgtgtgggttgggaccaaggctgcaaggacccggtgaatggaatccctagtcagtgtcagatttactgcagagacaacggttttgtcttagagaaaggcaaatgtgcaccggacaatggaaagcccaaccgcatgtgctgctgctataagtagacacttctgtgtgaattcgtgaaagagtgcaaaactttttctatatgagagatctcgaaaaataaattaataaagcattgtttgatctatgtctatcatcagaatgactactcagtgatcccgggcagtgctacgcagtacaacattcagcaatcaacggccgtggagtagttgcgatcccta

>TaeST2.2543.1

tagggctatacggattcgaaccgtagaccttcttggtaaaacagatcaaacggattattatcgaaatgatttgaactgtttcaaagacccaacatgcattttttgcattgggctctttcataaactgatttaaagatcagttagtccaccatactttttctttacggaaagataatgagatggctccctgtgctctgattgatttttttttattatgatctatctaggagcaataccaaagtgtttcaaaggaggattaccttgacttaggtctgcctccggtctaaattaaatcaacctaagtgaaatggagtctctatcgttccactgcaagagttaactatgagacttcatacaccttaaagttcatagaacgaaaagaagttttttggaggcccttatcctcattacgcctagcatttagtgggctggatatttaccttatcaactagcaaatccataagggttctatttgattaggcacctgaattggcacctgaatcggactgaaccgactgtttgtcaagctactgttctcctattctctcgaatctatgaagtaagacattgattttgcaataagatcgattatgttcattgcataataagctcctttgaaa

>TaeST2.2560.1

ttaagcaataatatcatgatcaaagcatcatgataaaatttcccctaaaaagtaacaacaacaaattaatttgcctttaggaatatactaattcagttctccaatcttttcaccattttattcatgccaaagatgactcacctgattagaataataaccaagagaaccaattaacatcacaacaacaagccacaatatcaattgattgatagatctacttttcctcgaaggctggaaccatcaccgacatcggcggcgtggctattgtatccttctcttcatgcacctctttctcctcggtgttggtggcagcctgtagttcttttgcgattgcgaggtggatgtgggtgatctgttcgaggccagagaacatgtcatcggggcagtcgcaagactccatgaaggcatcgagcgagtgctcgaagtcttcctgtgtcttcgcacagtcgggcttgcgagtggccgcgacggtagcgatgacaagcgcggcataggcaacagcgttgacacccacaaacttggccattggagatggaagaggttactctagatcttaccgca

>TaeST2.2812.1

gataacaatctcctcccacttttattagcaaaatagcttacacataatatttctggaaactagtcgcacaaattgcatataagaaacaaaacgaccaatttgaagcacattttattggccctagcttatagctcggaggagagtgagacaacatatcaaggagtggtggaggatgcagttccattggaaatcactccgccgaacccacaacaatagatgtccctagggcagcaaacgggattgtaccccatgagcctgccggctgcctacgctaccggtgctgccatcatcatcagtaagcaaacttgcgcgatgagaaaaacaacgaatacctggaggatgctattattttccatcgcctaagttcacacagagcaatagaaagcactatgcaatactcttgtca

>TaeST2.2827.1

taaattcacaattagtcaatgtatcttgtagatataggatacaacatctaagggatgccaacttttggatcttgtcaagacagagagatatgcattgagacaggtaatatataaaaatctcctcccacttttattagaaaaatagcttgtacataatatttcctgaaaatagtaacacaattacatatatgaaacaacacaaccacgtttaagcatattttataggcctagcttatagctcggaggagaatgaaaccacatatcaaggcttggtggaggatgcagttccattggccatcacactgccaaacccacaacaatagatgtctctagggcagcaaactggattgtatcccataagcctgcctgctgcctgcgctatcggtgttgccatcatcatgagtaagcaaacttgcgcaatgataaaaacaatgaagacttggaggatgctagtatttgccattgcctaagttcacagagagcggtagagaacactatgcaatactcctttcact

>TaeST2.2828.1

tttgaatctctttgcggcacattgcaagcaagatagatagcaatctcctcccacttttattagcaacatagtttatacacaacatttcttgaaactagcaacacaaattacataaaagaaaacacaaccaccgttgaagcacattttgtaggacatagcttatagcttggaggagaattaaaccacatatcaaggcttggtggaggattcagttccattggccatcacgcggccgaacccacaacaccaggggtccctagggcaacagggtggatcatgtcccataagcctgccggctgcctgcactattggcgtggccatcatcatgactaagcaggcttgcgcgatgagaaaaacaatgaagacttggaggatgctattatttgccattgtctaagctcacaaagagcgatagagaacagtatgcaattctcctttcaccggtatctccgtgtttggtccaagaaaatgctcgatatatatagatacaatggcatgaagcttgtgcttctcctaataa

>TaeST2.2829.1

gcaatacattggaatcaacaacaaatagtgcaattatgacaataacatggatttaaccataaaaatataggatgatatggtcctctaaaaacattgatttcgacataattgtaatctctttgtggcacattgcaaacaacataagtaataatctcctcccactttattagaaaaatagcttatataataacatttctggaaactagcaacacaaaatatatatgtggaacaacaaaccacacatgggagcatattttatatgtcctagcttatttatcggaggagaaaaaaccacatatcaaggcttcatggaggatgcggttccattggccatcacgccaccgagcccacaacaatagatgtccctagggcagcaaactgggttgtatcccataagcctgccggctgcctgtgctaccggtgctgcaatcatcatgagcaagcagacttgcgcaatgagaaaaacaacaaagacttggagaatgctattatttgccatcgcctaaattcacaaagagcagtagagagcactatgcaatactgcgttcagt

>TaeST2.2825.1

aaccgggacaactatcagcagcatcttgtgtctactaagccaagatgagggtaacccttatgctactcctgaccctcacgtttctcgtgctaccatcagatgctgaaacaacttgtagttccagggattggcccccggggggtacagcgtgtgatagaagcgagtgtgaggacaaatgtaagaactattataatgactattaccagcattctacttgtgtaggtctctgtagttggacttccatctgtacagacaatgggtgccattgctcaatttgtgatgatcacagccatccattcgggcactgaataaactgatcctaaatatgtatgctgaaatggtttcctcattgaaataagagagatattaattgcaa

>TaeST2.2902.1

agacgcagcactcctagcagacaacgatggccagatctatgaaccccttgcacacaacgatcgtgctctcggtggtgcttcttctcgtcttcttggctaatcatgggcaaagcagaaacttggatccagacatgggaaacggtagtaaggtcattttgccaggcgatctgtgccgagctggatcctgcgcagatatgccggggtgttactgctgtatgagcgacgacatctgccgcccttcactcgtcgcgtgctcgaaaacctgtggaaggtcttcttctacccgtgttcttcgccgccctcccgtctagtcctagttgcgtgcattttatggtttaatttaagtctggaataatgtgtaattaaaagagttgatgattaagttttctttgaaaacttctgatctaattcatcttcaatcatggtaa

>TaeST2.3091.1

gcagttttgcttacaacacatactctccaatcattctcgattctgtttctttaggggaaatctcgatcctgttaataagcacaaataagtaccactgttgcatcagattatcttgatggccaagtacattactgcgccagtcctactttctcaacttcttatcgtatgcctggctactacaacacaatgtcgactcatcgacggcatggataatgataagatcaatttaccaggcgggttgtgcatctacgacaaagacgttggtggatattgttgccttgtgacgaatttgtgctataggacacaagatatttgccttggaatatgcaaaaaatcagcccctcttctccctctgctcccaacaaattgacctttcgttagaagaaatcatatgtgatgtggatgtaatattttttaataaaaagtagcatggcaaatgaacta

>TaeST2.3106.1

cagatccaatgaaaatgaatgtatatttcttttacaaaaaaacggatttacaagctagccattcttcacatgtatacatacgagcaagaatatcctacgagtagcaacttctaagtgttctccacatcagattcgcacggacaagtgccagaatacatcaaattcagatccacttaaaatgcatcaagaaagcaacacacacgttgctagcatgggcagcctagatccaccgggaagttgcagcgctgcaaccaaaccacatgtgtacagggcccctgtcacagccagcaagtatactagaggcagcaacagtgcgacgaggacgggcatgccaacgcgccggtgacggggtccgaaagcggaggccggcttgggcaggagcagtgacgcggctgggccgagagtcattgcggaaccagctgctggggcggcacacaccggcggatggatctagcatgcgggcggctcaagcggccacgcgcacgaatcccttggggcggcgatgcggttgacgtctggtggcgcgaagctccgcggccgtccatggcgtcgggtgcctgagagggagagggcaagggataaggcataggggatagatagagaacgaggaatataggcaggaggaaggagacggtggtaggggcagtggagcttgagatggtgctcgtcggcggcttggggaacggcgggaggtagaagtggatctcgactgggagggagcagaggcgtgggggacgggaga

>TaeST2.3349.1

cagtatgatgaacacacgcgacagaaaaactgatagttaaggatacgtctcgtaccctaaaaattcctctttttattttcttcttttcttgccacggtgttacaactttgcacaacgtgctgtgtatctttacctaataataaagcacgtagggtttctgtcgtacgtcatcggcatttttacagaaacccccctgctattttggctattcaacccgtaatactattataagtcaacctgaagcgatactgacaagagagaaaaagaaataacccacccacccacccgcacgtccctgcctcccaatcccgtaaaaaaacaatcccatctcaacgtcctgccgccggtcacaacacgcgccgccggcccacccccacgccgctgtgtgtcgccggcccacccccacgccgatacgcgccgccggtgcatcagttggacgacgtgcctggcctccttgcgagttgcgagccaaaaagaagcaacgaggttcactcaaaag

>TaeST2.3531.1

tacatccaaccgttgttttacacaaccacatacacatacatatacacaaaatcaacactagccagttcaagaaaaataagtggaatatgggcgttgcatactgtaccacgccgcacacttctttgaccatggtgccaatctgcatatcatcgtgcaccaccctggatcactaactgcacagcatatgcccattccaaatctacaacaatttagtacttctctgaaactgaacatggcaagtgcaaaatttttaaaaaatggaagtgaaccaacgcacagtacaagcgaaccacatccaaaaagaaagtgtactcctctgtgtttttcataaacagaattgaaccaccaaaaaattgtcacaaacgagagcaaatagtggacttcatggtacacgcgagcaatgtgattacagcgcggctcgctgtgtacgcaaggtcacggcatcgactccccctgatgagagcaccttctctttccctctagcgaattgcaaatggcagcatggccacccttaaagagctttttttgtttttccaaaaagaagtgaatagcggacgccgatgaagcgaactgcacgtagatgaaaatgggtcaatacaaaaaatgtgagatcagggaaatatacttgcaaataacagctgccgaaactgcacccaggcgggccacgagctgcccccgctggagatgtgggctgcgccaaactccggctacacaatggatgatgggtgcaacgagtggatgactttgcagcatggcaaggtgtgcactgcacatgtcagaggagggcaggagggcattgacc

>TaeST2.3598.1

gttggtcaggaaactgaagctgcagctacgaagagccaaagaacattcttgctgaacaggctaaagccaagaggggtgctgctactggtgtaaaaaccattttatcaaaagaactgaagtagtagctaatgcaagcattgaagaattgacgcagaggttacgctatattggcatgaagtcagagcagtgcctgaatactacataagatagtttctggtgaccttgagtctatatgtcctaaccaacagcagccaccttttatttcgtatcaatcgatccagtatctttccccttttggctttgctctgagcaagatatgtatagcctttacatttccactcgtgtacactttagacctcattactgtctctgtggggaagtttgtgatcacttgttctatataagtaatcaactgcccttctactaatattttcagaagactatttatgtatgtttatactggttagagaaaatcatgacattgccagcaacaatttttatttccacatgtttcaa

>TaeST2.3883.1

tccgccgatagcagatccattcgataaattgagaagaaatcagcgcagaaggaaaaacaagatcacagaaacataagaccaggttgatcccaagtgcgagatgcagatagaacaattcatccggcattagcaagtcgataactgaaaatagatcatatatacatgccaaagaggccacaactcgatcgtattatttaagaactgacacgagaaagctaacgaaagttgacaccgttcaggcttgcgatcgatcggacaggaagaagacgggcgggcagggccggatgacgtagcgcctactcgtcggaggggtcgccatctccctaggacttcttgttgtcggaggcgccggtacccctgcccttgttgccgatgaggtcaagatcctcgtcggtgtagccgtcggcgccctcccaggtgaacttgccgccacggccgcccttcttcgcgtcgccgctcatcccggagcctgacttgcggtccctgcgctcctgcggcagcttgtacgggtcccggctcttcttcccgccgtccatcgccggccctttttcctcccctgcgagaagatccagcaacgagaagagacggaaagtgcttttttaagggagatgaaaagtgctgtgcgtctctgtttcgctggtgcttgtttccaggggcttatt

>TaeST2.3918.1

gtgctccacatctttcttctcttccccaagccgaagggaagaatccctttctcctctcttctccagccagcctactccctcctcccttccttccccactcctccccaggtgcctccttgctgcaccacgcccttcaccacctccatggctgccctttgatttagggctttgaggaagaaggaaaggagacgagggagaacaagaaagcttccccaacttttcccttaaccaaatctttgtgatttgaccatagaggttgcagcacaaatccaccggagatagcctcgcgccacctccctcgcaacggttgaggcgagtacatcaccgtgaggctttgcaagtattaggaagggcgtagctcccatgggtgttcactcacacatttcacaccccttccatccacctagccgatcatgataactctcatatttagttcatgattcttggtagctaggatctacttgtggtgtataaactttgccttggttggtttccatgatcactatgtttacgtgtcaaggtgtgttggaccgtatgttttctttgctatgtgtagcataacagttggtggcttcaccactttgctctacatgggttgtaggagcttgttggatgcttctgcttgtgatattatgaatatatgctttgtttatgggaaacgtttggcaacggtgcgccggccgagtttgcgccggtcgcacgctgcttcgcca

>TaeST2.3918.2

gtgctccacatctttcttctcttccccaagccgaagggaagaatccctttctcctctcttctccagccagcctactccctcctcccttccttccccactcctccccaggtgcctccttgctgcaccacgcccttcaccacctccatggctgccctttgatttagggctttgaggaagaaggaaaggagacgagggagaacaagaaagcttccccaacttttcccttaaccaaatctttgtgatttgaccatagaggtacatcaccgtgaggctttgcaagtattaggaagggcgtagctcccatgggtgttcactcacacatttcacaccccttccatccacctagccgatcatgataactctcatatttagttcatgattcttggtagctaggatctacttgtggtgtataaactttgccttggttggtttccatgatcactatgtttacgtgtcaaggtgtgttggaccgtatgttttctttgctatgtgtagcataacagttggtggcttcaccactttgctctacatgggttgtaggagcttgttggatgcttctgcttgtgatattatgaatatatgctttgtttatgggaaacgtttggcaacggtgcgccggccgagtttgcgccggtcgcacgctgcttcgcca

>TaeST2.4026.1

aaaatgtgcagaacatatattcagaaacagatttcagcaaatggtttctctgatccttcttcaggaactgtatgttatatgatctgtcaatcatccgggttgtagaaattaatggatacaggagtatgatgatctctttgtaaagatcgcttatttactggacttcaccaaataagtaaagcagaaaccaccaccagagtacaatattacactcaaacttgtatcttcagtagtagtagatcagtgaacaacaagctcatcactgtcactcacgggaagaattttaactatatactcttgaatacttgagctttccatgaaagtgagatcagtggcgtccatttccgaagccaccgccggagccataaccgtatccagagccagacccggagccatagccagagcctccaccttgaccacctccgccgccaccgccgccgccgccgcctccatacgctccaccatatccactgccagacccggaaccgtagcctgagcacccgccagacccaccaccttgtccgccaccgccaccacctccgtggccaccaccaccactagcaccaccgccatagccagaaccctcaccggacccatgcccatagcctgaaccaccgcc

>TaeST2.4144.1

cccacgcaccggcaccaacaccacttgccccatcgccgccaagctccactgctccgctcctcccctccgtctccctcccaagcctctcgtctccgacagatcaaggaggccaggagcagcaacaagatcagcagctcgattgggcaggcaagcggcggatggagttcgtgctcatcatatccctgccgctcctcatcctgatcctcatcatcgccgtcgtcctctgcatcgtctgccgctagccacatgccgactcgtctctgctcggtccgctgtgactgtggtcgcaccatatgttgtgttaatcaatccaatattacatcatgactcggcatagaaaagtgaagaaaaaaagctcgtctccaccaccatttctatttttctgttctgtcactgtggtgtgattagctggctatagctttattggctttcctgatggcaacaaaagcatcgatcccatgtgtcgatgcgcaatgcatatgatatactatgtggctgtaaaaaaaagaacaaattccgttcctgctctgattgattcggggaggttgaatacctgaaagtgccatcttctttttactgtacatttttcttctcgttcttccgagttgttcgagatcggaatgttgcaggcttgcaggttcaggagaccgatttgcgaatctcctatgtgccgtgctcc

>TaeST2.4161.1

taaccactcaaatatatatttatattcacacacctcaaacagtacagttgtacaagtgtattacaaatctttggacaaaagtatagggagaccatactatgctaatctatacagcaatgccacaccaaacttagaccaacacaaaatatctttgagtcaactaggacaacaggacacagttgagacacactaaccaccactctcacgtgcacaatatattaatagtcaattggtcaatagtatagtctatgtatgggcttcaaccttcagccaattagaagctagcacctgtgagaacaatggagaagggaatgggctgccataccagccaggggagatggatttctgatattaagaggggagagagcagggcaagccattgccaagacctcactcatctccattcttccaaatagccaaaccaacagcttgtcttccccaccgtcttcatctctctacctacaaccaacaagaatccaagagcaataagtgaagatggtgacctgagcaatattggagatcgaaggggacaggctagagcaagaagctagcctactggactggggtctggaggtaggagcaacactagtaggaccaacagagggagcaagagaagaaaggaagcgagtatagtcaggagaaggaggcgctacaaaagcaaactgagttcggggctgaatacgataactcagagcaaagcttgtatgagaattctactggaccaagtgaggagagcaatggtgatggctacaactacacaggggacctagatgtggatccatctagtgctatgtacgatctggatgccacatatgggggttgggatgaataagtaactatcatctagctatgtaatccaggaactatttttatcaccacctatttggtactgctagttggaactccagtgttaggagatgtaataatttggtgaacaagtgagaagcctacctgagacttttagccctgcgtggcatggtattgtaatataaattaccatctctagcta

>TaeST2.4205.1

aggacaataggagaaggaaccaaagaaacagtaaagaacttttgctatggctccaatcaactcaaggtttctgtgcttgatggttcttttgatgatctcatccacttgcttgtcgagtcaagtatcagagatcaacataggtctggaagccaacgaatgccggggccgtgaaccctaccagtacaattgtacgcctgatggttgtgttactgcctgcaaaaaactgggctgtgttactggggtttgccacaaagatggtagttgctcttgcagctagatgaggctgctcagtcctcagtggttgctctaatttgtcgcaagaaaaagacccagaataataagcttaggacataatgttgagctgtcttcaattcctttgattagttaaatggagattctttattttccctgtggtctcccgtatcatattttttttaatgaaagaaatggtggtcttttggcttagtctaccacttgtgttaaaaaactttgatgttgtggaa

>TaeST2.4323.1

ccccttcctcatgattcccctctcaccccgagctccatgaccatgacgctgcccgtcaccaccaatttctgctaggtgaggcgctttcccacgacgtcactcacaagcccggctgtcgtgcagtcctctcctctgatgcttcactgccgtgacttcctcctcgctgccacacatgcccctgcagcaaccctagccgccacgggcctccggtgcgactcccgcgtaggccctctaccccgcatctccgtatgcgccggtgccagctcgggctcaacggtgttgcccgcctctggagacctcgccttcgatattgacgatttagttctgaaattgacattgcttcagccaacatgtgtgtgcttgtttgattgcattctcatcctagtagcagttcaatctgttctgttgatgaacatgcag

>TaeST2.4324.1

cccctaggatttaagatggtgaagttacttgatgtaggacctagggtatactataactgtatcaccttttgttgtttatcggcttgcttcaataagaacttcagtgcttgagtccaaccctttaaggtttcactttgctccaattactcaatcagaactcgtggttctatatgttgtaggctctgaacttccttcaatcagaatctattttttcacaagattatgaaatatttatgtcccccaggttcttattggccaatttctcagattaggagttgatataaacatcaatatgctcaagattacaaagagattgcccaatctagaaaagaaaaggtgtatgccaaactgaagagtcgtgtgacgtaagcagctgagatgctaaaaagtttgtattgtcaagataaaatggataatgcatggcttggatgctgattgcaaggatgcgaatggtgctacaactggataattagaatgtcattttcaggatgatgtttgaaacattatagttgattgcaaggtctagaaattggctactacttgatagtttcacatcttgtaatctttggatcctgttctttgtaatgcaacatagttgatttaatatatgagaccttccagtttatta

>TaeST2.4546.1

tgggtagtatttacttattttcttaacggattcattttagacaaggtttacaaaatggtgcatgttcatggtgcattcatgtatcttgtatttcatatgttgaatttagggatgttatccatccaattcatgagcaaggctatccatttcaatgatggaaagaaaatagcaagctttctttagtcatagattaaacatgacaacccaaaagttccatctccggagttgagtggtgtttagtcgatagggcaacaacaataatcaacatctttgcagtggcctccagggtttatgcaatacttgtagcatgccttttcagtgcatttaggaaacttcataggatagcaacttgcaggtactcgtactgcatctgcatgtatctcattgttcgcatggcatggggacacaatagtagccaggaccatgagagccgccaaaaacaagggaccaagattcatcttgaaggttgccattttgtgaggccagcaagtgatttctattatcaattgttg

>TaeST2.4689.1

tgaactaaaactaaccacggactaactgcagaagaatcaaatagtacataatttactaatgaagagtacatcttaaccagcttcggactaactgcagaagaatcaaatatcacataatttactgatgaagagcacatcttaaccagtttctgaaagtctgctgggcctacaatgaccggaattgaggtccacgtcccaggttagattcttctgctgagcacatattccaaaatcaaggcaggcttcttcatagtggttcagccaatggcgattagaacaaatatgatgtccaggtccagggaagataactgtagatttcatttgtgtactatacaatccgatcatacagctgttcaggctacattaggtaagagtttacaaattgaaataggagaccaagatcaaattgtgatcatcaaatatattaacataaatttagacacaataacaacctgacacatataccatattctgaggaaaaaataaaatatattgaatctgataccaaagcgatttggtcgtttggaccgccgattgttattgaagtgcaaatttgagctgtgatgtggtactatatgcaacagcgaatcacacgaatgcaacagcgaatcacacgaataattttaccacaactatgagagtaacttaaataggttccagtgattacgaattcctgtgtaaacgatgtcagaacagaatacaatttacgagagcagtaatttgtcttaagtttatgtatgcttcagctgtagattatcgattgtacctttggtctttggtcggtagctccatgatcgattcgcgacggtagaacgttgtgccaggaagatcggatcggacgagaggtgcagacaaaggagcacaccaggacacgatggggggagaccccggggctaaaacagaataacccccgtgcgatctccccccgggacacaagaaccatctttattacggtggcggcctgccatgcgagcgcacccaacatggcaaaggcctctagatgtcacatctctcttttttcactcctttttccctcttggcacgatcacctcgcttgttacatgctcttacctccgatctcttagatctactccctccgttccaaaatagatgactcaactttatactaactttagtacaaagttgggtcatctattttggaacggagggagtaataattatcgagatatcatcagataattatcaatacaagagccgtcgtcgcgcgaggcggagaagccgggatccacacgtgcacgtcggagaagacgccttcctcaccgcagagtcaacccgagaccgcctggaaccc

>TaeST2.4923.1

ccccgtcctctctctccattatctcatctctctctctgtctccattatctcaagatccagccagaccttcctcctccatggccggccaccctcccgatctggaggtctccgaccagattattcccctccccaccacggcatgcggcatggagtcccgggatcgctcgccgtcaaccaatgctatgcggatctggcgagtcctaactcaccaagatcccatgacggacaaggtttgttccgcctcctcctgaagctgtcgaggaccatgaacgccacttcctctcaccggttcagcaccaacacatccactacccgcaggtaatccccctgtcccccgctccaaatccaggacctggtcgacttcctctggtcaagtccgcctcaacgaattgggattgcttcttcctaattgcccatggatttgtaatacacttcgataaggatttgtttgtaggtgtgcaaaattgcttgttccctgatggatatagtagcacatggatccatcacctttggtcttgtctggtgttctccggtccgtgatgaatttcgaaggatgaaacagaggctgggcgacagggagatttagctggaagagtgttgcctggtgccagatccgccatgaagtgagctggccctctccctctcatgccgacatcggcatattgcctgcttcttcccccggcttgatgaacctaccttcgccaaattgatggattttaatacagagaaaactagttcaattgagttttatcttaaaaaaggctagtcatcgcctgatggccattgtatacataaaataggctgcagtttatttttcctgtactgctgcaataaggcaacgtttcatcactattcagttctatgcaattccctttctgttttttgctctgggtcatgagcactaaattttaaagtttccaacatagattgcaatattatgggcagaattgtacaagaactttttgga

>TaeST2.4971.1

catatcaaaactaactagagaaagaacagaaaagagagagtatacttgatacttcgcccaacaccatcgattatgggtgcgagaaatgctaccatctgctttgtgctgatccttgtgttattcgcgaatcatggttctgcagaaaaaaagtgttggggaaatacctctgggagtccactctgtgtaggcttcctgtgcaaggcaacatgcttgattggaggcaaaatgtttaacgccgacgtaaaggagcacaagtgtatagggccagcatggagcagcgaatgctactgctatttctgcgacaaatagtttgttagcgcggagctgaagagtgcctcattattacaaatggtcaaagaaaacagagacaatagttcgtgatttcttggtttagcatataaaggggatgaataatttattctcgttgtcaacctcattgttcgatgatgttatgatgatgcgaattttataaacaagtgcagtattatatcctggcaaaagatgtccattgatttctaatcgtaaaagtatatgatgtgctaa

>TaeST2.4969.1

tttaaatcgcagatcggatactcataatacagatcagttaactgcacccacaacttcataagaaaacatgcatattttgaagaagacactgtgaacaacaaactgacccataacaatctagcacagattcatcaccaagagcaactcacatgatagaaaccatgccacctttgtccaaagatggaccggatgaagagcattcaaagagtaccacagctaccgccgacccaaacgaaacaaaccacacatagttacggccatacgaccagcacatcgcgaggaagcagagaaaacatacaaaaatgaagcaagcagctaagcttaggcacgcttgcacttgaggttcctcatgcaggagagatgcatctcggcgaccttcacgaccttggcaatgccacggctcgggcagctccgcccgggcacgcaccggatctcggcctcccgctccacctggcagaagcacctggggttctccaccatggcgcagcacctctcgccgaacccgcgccgaaggcagttgatgaagtagctgccctgcgtcgcgcagctctcgggcgggaacgccgtggccggcgtcgccgcgagcgcggcgcacacggcgacaagagcgagcgcggcggcgagcttcacggacgccattgatgtttctgtggactgggatcgatctggatggagtcacaggccgagggaatgatggattcgcacagcatgtgatggtttttgatctgtcttctacaaagcgcttggcggtagcggcagcagtcacg

>TaeST2.5016.1

cccactctggacgctctatttcgataagctcgagggcgagctcgagcggcgcagcatagcaggctattctccccgtccatggcgattctcgccgctcgattttgatttcttttccctttctctttgatctccagtgtcgcgtgtaatttgttggtgtaatttgttcatcaaggaattcatcgtctccatcttgtccaaattcatgttatagaacaggtttgtgtcccagggattggattctcctcccccatcttcgcttgctggtatttcctgatgcaattctgcctccagtgtctactaagaaactccttcagttttttgttcttgcaagaggtgaagagagatgatggatgagggagttggtgtttgactgtttttgctgtgtggtccctaaatcaggagggggtttgtttgtccaggacgaaccgggtgggatgtttatccgacctatttagtgattttactgctaataaatgtgtactgtgttgattggtcaggaaatttgactgttttcggattgaaaaacagtcaaatgtcaaatagatagtccctaaatttatacttgagcca

>TaeST2.5016.2

cccactctggacgctctatttcgataagctcgagggcgagctcgagcggcgcagcatagcaggctattctccccgtccatggcgattctcgccgctcgattttgatttcttttccctttctctttgatctccagtgtcgcggttggttgctcctctctcccgctctcttccgctctctgcccgtgtgctttttgcttgcgtctggttccaaatctagtgtaatttgttggtgtaatttgttcatcaaggaattcatcgtctccatcttgtccaaattcatgttatagaacaggtttgtgtcccagggattggattctcctcccccatcttcgcttgctggtatttcctgatgcaattctgcctccagtgtctactaagaaactccttcagttttttgttcttgcaagaggtgaagagagatgatggatgagggagttggtgtttgactgtttttgctgtgtggtccctaaatcaggagggggtttgtttgtccaggacgaaccgggtgggatgtttatccgacctatttagtgattttactgctaataaatgtgtactgtgttgattggtcaggaaatttgactgttttcggattgaaaaacagtcaaatgtcaaatagatagtccctaaatttatacttgagcca

>TaeST2.5220.1

cacacacacacacattcacggtgcttcaagaagaaaaatgaagagccgattttgccaccttgttttcattttccttgtggtctctctatgttccttacctgcatttggtatacgccacggaagctttcaatcagtgaaccatagggacctggctcccagtccaagttcgactgagctcccttctctggcacaaagccgtgcgtttgaatcattgatctcggtggactataccggtgtagaaggacacgcaccccagatccacgcacctaagttggtagaggcgcctccgtctgactaaagccttcgagtatggcacaccttgtacaggacgaggaagtgccccaataaataccc

>TaeST2.5305.1

taccaaatccttacacagcgagggcacaggatcgaaaattcctcggcggcgggggcggccgctcaaatcctgagccaagccgggcgggctcgcgcagcaaagatgttgatccatctcctctccaatggctcctaaccgtttcgtcgccagtggcgcagtttgaggcagattagatcggattccttacctcctccccactctcaggctcccagatcagctcgcgaccgtcctcctccaccccccacccccactccctctcgtgattcagcactagctctcggaggtcgtggatcctcgccttcttcctggccggccgcttctagttctccgaacccaccacggttcttcgttcttcagaaagggaatatgtactataattccaaacgctccatggaaggaattgaattcctacacaatagtttgtgtagcattgagttgggtttagcccaactagtttcggctgtaccatgcaatacacaagaaaatgaggagtagtgtatcgggagcatcattccaaagcatgttaccatcctcacacctaaggatatcaatgcaaggggcacatgctctagaataaaggggcatagggatggtgtgaggagtggatctggtgagaagagaaggcctggaattcatcaaaaagtcgcgcgcaaatgcaacatttgtaaggaaatggtgtttcatgatgcaagaacatgttctaagaaacaaacgacaaaacaatagaggtagattgagctgatcaagtgtataagagcccatggaggttgtcatctctttcccagcatgaagctcgattgctaggaagcactaacacgaggcaaaacgacagatattttcagtcttgtaccagcaagatgcttgttgggcgatgataatttcagcctttaccacttggtcttgccggccatgtactaaaaatgatctgtatggctcatgtacccaagtactcaaatgcttgctgagcactgataatttcagtccttaatttgctctacttccagtttgacatgtatgcgtggttctacttgcctcttcgtgatgtgttctcatgtactgaaatgcttgccgagcagctacaatttcaatcattaggttgctatatttccagtttgacatgt

>TaeST2.5379.1

ttatactaaaaagcatcctttatcaaataaacaaatgtgtgtacccaacaatttttctatttgaaatagaaaagaacatgtaatttacaatatgtactcatatcatgtatgaatacgagacgaaccaaagaaaaatcccaaataactgcgcgcacaaggacatggcctcagaagaagtagccgaagacgagcgcggtcagggacaccatggcaacagctggggcggcagctgacgcgccagaggcaggcgcgggagctggtccatcggccgcggagaccgtgccaaccatgacagcggcggctgccgccacggcgaggatcctgagcttcagagtaaccgccatttgcttcctcgacggatgttgctgcaatgaactagcaaagatgactctggtgctgggagagagccggagaagttgtcaggcttgtg

>TaeST2.5486.1

cgataggagaaaaagaatagtgtaccccctccccatggcgcttctcaagagcaacccagaggttttgtgcttggtggcccttcttgtgatatctgcgaccttcttatcatgtgatgcatcctacggggttagagatggagaaggttttaacaagttctgtgtgggttgggaccaaggctgcaaggacccggtgaatggaatccctagtcagtgtcagatttactgcagagatatcggttttgtcttagaaaaaggcaaatgtgcaccggacaatggaaagcccaaccgcatgtgctgctgctacaagtagacacttgcgtgtgaattcgtgaaagaaaatctttttctgtatgagagatttcggaaaatatattaataaagcattgtttgatctgtgtctatca

>TaeST2.5568.1

gcattgaaattcatcagcatagatcactaaactagtgagaaatctcaatacatcatctctattacaaaatggaagtacatttgatggacggttttacagacacaaatgcatgacaacatagcaatggacctcactgaaaatgagcagtatactggtatttggaatgattctccagttgtgctgcctaggtcgtgagtacacagaactgagcagggcttcagtttctaaataatctgctagcagacttgctaatggaaaagcatctcctggtcaaaattaaaaaactgcattttaaacaaaaataattaaacagcaatgcggtattggtgctcagattctatgggagtcattcacaaagatatcactgctgcccaatcacatctaactagattgttccataaaaatcataacgcaactctcagtgaaaacaaagaagaaataacataataatacgcattgaaacaagaaatagtgtccattgatgctcagattctatgggtgtatagatgtcgttcacagggatttcccgtcagtacgaggaatgttttataatcactgcaccaaagaaggatcaattgactcatcaacaacaacaaaacagagtttaaactgcaatttgggggcgagcgtaacccaagatccatctggacggatttgtgctttaaaagccacctgaactacatctcaggagcgttgttaactcagatgctacaacagcagccgaacattcaaacagtgctccccacattacatgatgaaacatcgagcagaagacaacaaagtatgaggatattcatatggctcagacatccaaggaaggaattagcagcattagctctcaagcctggctgggcgaaagatcttaggcggcatctgcgaatgttttgagaaacaagccttaaatttatcatcatcgccagaacatatcctcaaaagtgatcaaatcgacaaataacttgtgagccaatacaaaaatattttaaccacaatagctataccaaccaaaataaaactaaggttcaacacacaaacaacagagcaaacaatatgagagaatccctcagagacccatttaaaatcatgcacacagctgaaccaaaccaggtgcaaatggagattccagccctgaccaaaccacaaaggtaatagccatggaggacatgtgcagtggtgagaaacagctgctgtttacagtgtcatacggcacttcctggtctaagaatcatcaccgggaaaatttaagttaacaaaattaactatataattaaaaaagaaaatcatacgctactggttttgtgcctcagaaagccgcaaaaatatcgtcattcaaaatcagacggtaattccaacataaagctaatcatcataggcttcatttactttgcaggtctctagtgtaatcataatcgctgtccagctgacaaaaaactaaaacaatgactaagataacatggtgtatcatcagaatataaaagccttctcatccacaggaaaataggcactgtgtcagaaggcgtcctaagattgaataaggtttcatcacttgatacagacgaaatctagaagaaaataaaaactagaaactgatctatataatggattcagttgcaaagatgacatcacataggcagctaatgcaacccctcggatcaagtgttggattatgcatcaatacctcctcacaatccaaacagatcatcgacaaaaataacagcaaaaatgattgaaaatatagctgctcagtggtggcaatgtttaattggttcgattctgccagcaatgtcgtcaatgctttcagaagagggagcttgatatcctcattgcaactacactctccaaacgaaacacacgcatcaaacaaaaaataggaataagaacaatgaacggcagattcaaaataaagggtatgagatggctcggttcagcgggataatcggcatacaaatcaactcctgagagaagacctgtaatttggtctgttgagcttgaatatttgcaaatcgtcactgccatcacccaagaatattgagtagaaaggaaaatcaaaccaactatctaaattttaatttgctgagtcagatttaatagtatgtcaatcagcacagatccacctaagcggaacctaagactggggcgacaaatttgaaatcatcatcctcataactacaaagatctgctgactatgttccttctgtaccgtacaagattatttcagtctaaatgaatttcaattgataatatgtaaaatgaagactcagaatatgggtggtgatatagcaggatggtcggcaaggcaaataaatgccagttacgacgaacgagaccaaataccaatcttcaccgtcctctacaacaaattcagacatgacgttgaaaaataattaacaagcaaactaacacacgaaatgttgattctcaagaaaaagcttaagggttctaaggtcatcagataggagcgaaagactaattttgttcaagtaatcacgatttgatcatatgaactatgcacttgtgatcatcatccgaccttagcaaaccaataattcaagagaaaaaacaaaacgccaagaaaaggacagaaggattaaagggatcgtgatgcctcagaaggaaggcagtaggttatgtaatcactagtgattcattgagaaaggttcacactaatcatcatcagcaccacgaaaccacaaataatcgaaaattaaaaaatgagcaggatcattggcctcatagagaagattagatcgttccacatgaagaaaattcggacatatgcttgtctataaatttattttaatcctcggccagcgataaacaaggaaaaaacaagaacaaatcagcagaagagtgttactcggggtgaggtaattctaatcaagaagaggactcggaatatgggtggtgatatagcaggatggtcggcaaggcaagaagatgccagttacgacgaacgagaccaaatatcaatcgtcaccgtcctcgagccagatgcaaaaatcaccacgggaagaacaagaaccgaaaacgggttgcagaacaagaaggcaggtcgagatctgatgggtcag

>TaeST2.5834.1

ggataggatggggaaagctcctgacgagagggagatgaagcctgacaagaaaacagcactggaattgtctgtgcgcgcttatgctgagaaaggagctggtcatgttgtcaatccagcaattggcacatcttttgactcgttagatgaggcttatgagttctacaacctttattcctgggaatgtggttttggagttcggctatgttgctcggaaaaggtgcatgcaagagattgtttgtgattgtgcggtaggtgatcgcaaagtgcattatgtttgatgccataatgtttgatggactgtttgagcctaacttcctctagattaaattagttgtgacttgtgaggtttgatcgcaaagtgcattatgttgccaggaaaggaatgctaggatctttgaacacaccgccagtatcgtcgacagagttttggaccagattagagaagatcttcaagtctggagggcagcggggtgtgttctggatcttactttgtaaaagcctcttataaaagcctcttgtaattggggtgttctatcctgggggtggcgccctcgggaggtctttcagtgcgagaccgcctagcctgtctgaccttgttgtactcttctcctctttctaataaaattcggccaacggcccttcgaggtttgatgccatca

>TaeST2.5869.1

atttaggaatatactaatttagttctccaatcttttcaccattttattgattcccaagatgactcacctgattagaataataaccaagagaaccaattaacatcacaacaacaagccacagtatcaattgattgatggatctacttttcctcgaaggccgcaaccatcaccggcatcggcgatgaggccattgtatccttctcttcatgcacctctttctcctcggcgttggcggcaacctgtagatcttttgcgattgcgaggtggatgtgggtgatctgttcgaggccagagaacatgtcgtcggggcagtcgcaagactccataaaggcatcgagcgagtgctcgaagtctttgagcgtcttcgcacagccgggcttacgagtggtctcgacggtagcgatgacaagcgcggcataggcaatagcgttgacgcccacaaacatggccattggagatggaagaggttactagatctcac

>TaeST2.6041.1

taagagggagtaacttgtaataataaaatggttcagttcattatatcactttgagcatgtcatggaaaatgtatcatgatataccaaaagcaggtcctaacgatagtgaaagagatcaacagatatggttcttctaattctgagctcatcttcgagagaaattacatatttacatatctcctaattctgatcccatctttctatagttcagtctttaataattatttcaacatcaacagtatatgtgaacatcaggagggaagcagatgtgtgccttccttttgcatttgactcggttgaaacaataaaacctctccacataaaaaaagcaatctgcaaaagcatgttccaaacattttagagcatgctggcaatacaaagcaataaatggttgtacagcaaagcagaagtgcatatgagctactactgtgacgtttagttatgaatcaagtggagcacaataagatcatgttcataacctgcaaaacaataataaaagacctcaatttattattttccatggatttaagtatattccatgagaaatttctgctgtaaccaagtctcaagctcaaaccaaaatattttagcatcattacttgagttgttttctgttttctgtaagttgtagatctgatgctacacgccctgatcagtgcgcagtagtaatatatgattcaggtataatggacactcatgatttttttcctttcttaaaatgaggtatacaactaatcaactatacacaccggatactcaagttacatggtaataaatattcagagaagaaatatatcacgacaatgatcgcaactcatttcatgtagtgcaaatcacaaaagggggaagatgctttcatggaagaatctaaaatcattcactgcataacaaatattgtttatctactaccaattatatatcgcagttgatgtatgtacattaccaagacaaagcaagtaacaggaagcacattattattttgagctgaagtgtatgcagatggcacaaatactcgaggcatgttctaattattctactggctctaggtccaaaagaatgagcaagaagaaatatatatctctctcagaaaaatataggattactaatctgaagaatataaatttgtctgataaaaaaaaatccatgcatgacttcctacctcataagcatgcggaccatttagaagccaagctaacatcaagtacaaaaaatacaaaccgtcattattggcaaaatgccgcacttcctacctcagaagcacgaggcgtgcaataattattgggatatcctaccatgaatcagcaggcacaggtaaatggaacatacacagtaatagttcaca

>TaeST2.6143.1

ctggaatcaactaaggatggtacctttattatcataacatgtgtttaaccatgaaaatatagggtgatatgatcctcaaaaaacacagtaaacataattttaaatctctttgcggcacattgcaaacaacatagataacaatctcctcccacttttattagcaaaatagcttacacataatatttcttgaaactagtagcacaaattgcatataagaaacgaaacgaccaacttgaagcacattttatatgccctagcttatagctcggaggagagtgagacgacatatcaaggattggtggaggatgcagttccattggccatcacaccaccaaacccacaacagtagatgtccctagggcagcaaacgggattgtatcccataagcctgccagctgcctgcgctactggtgctgccatcatcatcagtaagcatacttgtgcaatgagaaaaacaacgaagacctggaggatgctattatttgccatcgcctaagttcacagagagcaatagaaaacactacgcaatactcttctcactggtatg

>TaeST2.6144.1

tgatcctctaaaaacactaaattaaacataatttcaaatctctttgcggcacattgcaaatagcatagataacaatctcctccacttttattagcaaaatagcttacacataatatttcttgaaactaataacaaaaattatatataagaaacaacacgaccacgttgaagcatattttataggccctagcttatagctcggaggagaatgaaaaaaaatatcaaggattggtggaggacgcagttccattggacatcatgccaccgaacgcacaacaaaagatttccttagggcagcaaactggattgagtcgcttaagcctgccagctgcccgcgctaccggtgcttcaatcatcatgagtaagcaaacttgcgcaatgagaaaaacaatgaagacttggaggatgctattatttgccatcgcctaagttcacatagagcagtagaaaacactatgcaatactcttttcac

>TaeST2.6145.1

taagggatgccaacttttggatcttgtcaagacagagagatgtgcattgagacaggtaatatataaaaatctcctcccacttttattagaaaaatagcttgtacataatatttcttgaaaatagtaacacaatagcttgtacataatatttcttgaaaatagtaacacaattacatatatgaaaaacaacacaacttatagctcggaggagaatgaaaccacctatcaaggcttggtggaggatgcagttccattggccatcacaccgccaaacccacaacagtagatgtccctagggcagcaaactggattgtatcccataagcctgccggctgcctgcgctatcggtgttgccatcatcatgagtaagcagacttgcgcaatgataaaaacaatgaagacttggaggatgctagtatttgccattgcctaagttcacagagagtggtagagaacactatgtaatactcctttcgct

>TaeST2.6146.1

aatctctttgtggcacattgtaagcaagatagatagcaatctcctcccacttttattagcaacatagcttatacatatcatttcttgaaactagcaacacaaattacatataagaaaacacaaccaccgttgaagcacattttgtaggacatagcttatagcttggaggagaattaaaccacatatcaaggcttggtggaggattcagttccattggccatcacgcggccgaacccacaacaccaggggtccctagggcaacagggtggatcatgtcccataagcctgccggctgcctgcactatcggcatggccatcatcatgactaagcagacttgtgcgatgagaaaaacaatgaagacttggaggatgttgttatttgccattgtctaagctcacaaagagcggtacagaacactatgcaattctcctttcactggtatctccgtgtttg

>TaeST2.6142.1

gttaaccatgaaaatataggatgatataatcctctaaaaacattgaatttgacacaattttaatctctttgcggcacattgcaaacaacataggtaataatctcctcccactttattataaaaatagcttatataataacattttttgaaaatagcaacaaaaaatatatatgtggaacaacaaaccacacgcgagagcatattttatatgtcctagcttatttatcggaggagaaaagaaccacatatcaaggcttcatggaggatgcggtcccattggccatcacgccaccgagcccacaacaatagatgtccctagggcagcaaactgggttgtatcccataagcctgccagctgcctgtgctatcggtgctgcaatcatcatgagcaataagacttgcacaatgagaaaaacaacaaagacttggagagtgctattatttgccatcacctaaattcacatagagcagtagaatgcactatgcaatactacgttcagttgtatgtttgtgttaggtccaagcaaa

>TaeST2.6723.1

atttatcaaatgtagccatatgggagtccctttatatattattgcaaacataaacacacatacattttttgggggtacaaacacatatgcatgaaaccatcaaaataacggaacaacaactactactggagcataataatatgagtaggacactacagcatctcggctccccttcattgtttttccttcttattagtttccacggatttttttaaagtgaattagcttccacagatttgtttggtcctttcgctaagcttgcacaattcagccatagtaggcccacccaagacaggaggcccaccgaggccaggatttaacttcatgtagttacaaaggcacatgtttcccctggaccgctcaagacctttgcctaccgcctggcagcactctttctccgcaacgggatttgaaccaggttttgttggtggattgaaagcaagcaagcatttgtccctcatggtggtcaccaattggttgcctatcatttcaacacatgattttgagtccactggcaacgggagactgacgaagatcaacgtcagcgtaatcatcaacaaacctatgggagcccatggttttagtgccatgccgacagagaatacccctcctggtggggaggtctgaactggtgttgtag

>TaeST2.6853.1

ctcctcgcctcttttcccctccccatcccttgttctgccaccactgtattccttctgactcgaatccaagggagagacgacctgatctggtgggcttgtggcgacagagatggcaggggaatccagcggaggggatgtaagcagagataccaaccatcaaaattcagagagcaatatcatcattgaagacctggaccaaacttttcatggcaatgtttctgaagttgaggcctctaatattgatttaacagaaacggatgcatcagatggcatgttgcttgatggtatcagaaaatgctgcaaaagagcgatgagagtaaacatgggaggaatgttttgaaggagcaaagcaggaagaggtcaagggcttacatgggaacaacccaagaatgtttgaataaaggaagagcaagtgcgtatagcagatttagcatagggtgttcttccaagatagtcgacagtgtttgcaaaagcaattacagaatggagcttgtgaagaactctggattcggatacatgcttgtgaagaactctggattcaagcaatttttccaagtccta

>TaeST2.6935.1

tcgatcgaccacacacaagaaaccaacacaacaatgtagtagccgttgcacacaggccgggcacaccgagcaactgtcaaacagctcctactattcgtccccaaactaagaagatggtgggagaaacacacaaaccggccggagattattacagacataatgtatatgtctaggtgcggtcaacacatagctagagaaattcctgatctagagtagtacagtaccgtgcctgctgctagctaatgcatacataacatgccatcccttgctgctaattaagttcattacaactagcccagctcagatcgaacgacaacgaccaaacacacgcaaagcaacacaacatagtactacgtactagcttgcacaccttccagggttttggagcctggacccgatcttgacatgcgatcgaggtagcaatagcatgcgcctcagacctccgcgccgcctgcgccttccgcctggcgggttgcagaaagcccggaccttggccacgatgaacttgccgatgcagaagaggatccccccgtgagcacgaggaaaggatgaacaccacaccggcaaaaaagccataaaaaaaccccttctcgtacggcgtcatgcctagcttaacaaggccaggagatggggcaggtgagtggagagtgtgatccatgggtgcggaggtgaacttgaacatg

>TaeST2.6936.1

ctgtcaaacagctcctgcatgccatgcattcgtcgccaaattaagaagatcgagggagaaacacacgaagcggcgggagattattacaaacataatactaggagtagatgcctgctgcggccaacacatagctagagaaactctagatctagagtagtacagtaccatgcctgctgctaatgcatacgcagtacataccgtgccatcccttgctgctaatacatagctcagatcgaaccaccaaacacaggcaacgcagcactacatatatactacagcagcttgcacaccttgaaccatgatcttgacgtgcgatggaggtggcacgcacctcagacggccgcgccgcctgcgccttccggctggccgccagccccgtggttgccgccgtcgcctcggcaagcctgaaccaccctcacaaagatcttgacgatgcagaggaggatcacccccgtgagcacgacagcagggatgaacagcacacaggcaaaaaagccatcccaaaaacctttctcgtacggcgtcatcgctagaggaggaggtggagatgagtggagagtgcgacacagagctgaacttgaacaaa

>TaeST2.7021.1

aacacagctttgctgagatccttgatacatatgtaaagcggaggtctgcttcccggaggcatattcgaggaaaactgtacgaagtggcagcctaatttgagctaatgatatccaagcaccaaaagactagtatggtagcagcgatgatgctaaggatgaatggagacttactgctgtttactgaaaaaggctttcaccccgctttatatataaagcaacgatcatcgacttgctgctgtttgaacaatgcttccagtctcagttggacgctcattgctgtttgccatccgtcagctgccggtatcaggtggactgaagttcgacggtaaatgacagttcaaagatgatggctggacttccttttcgcaatagaggatcgtgatatggtggttgagcctatgtcagccactggttccactcagccgagaaggccaggcataaggtcttcaccttcagcatccagtaagagtgttacggcttgcaatttaggttctagttccagatcagctgccctgccaccactagcaccacaagcagacagggctgcaaacccttaacggttcgatgcacggactatacatttctccgtaaatgcctgtgtactggttgttgctggactctggggacgcttccaatcttgtcaaaacatcttgcagacagagcttgcaagcttttgttgctgggaacagtcctctcgtcaggatatattcttcatacagtacttactatggg

>TaeST2.7283.1

ttggtgtttaagctggagagaaagaatcaaataaatggtctcattcatagcaagaacaagagagtaagtacatgatcaccaacacaaagaatggcacgccggcgcacgcacgtactcgacacctcccacaccaccgcgacccatacatacacgcacgcacgcgcccacacctcacaccccggcacgaacacacgcacacacaccaagaacaataaaccaagaaggacaagaccaagaagggtcacacgccacgctacggagagtcgacgacggatttagcagaagaggtatccgaaggcggcggcggcgaatgaggccgcggcgaggggcacggcggccgcggcgtcggaggcgggcgccggggccggggcctcggcggcggccgcggaggccacgagcgcggcggccatggcggcgacggcgacgagcttcatcctgagaccggccattggcgacttgacttggtggttcctggtgctgggaggaggaggccggagggggtctctcgctgctgctgcttattcggctgtgagatctctctaggggggagctagagagatgctgcacgcggatgagggagagggcttgagagtggcggctt

>TaeST2.7613.1

tcctctttgccatttcacacatgcatcccctggaacagaggttgggagagatggcaatggcgatttggatctccctcttggcgatcgatgcagttggttgttggtgactcatctgcactctttgtcccactcccctgctgcctccgcgccccgctcgacctcccgtcggttgtctgcaggatatctcgccccccgcaccaaccacggccccaatttcccgcctcctgccgatttttgttcatctgattcgggaggcaatgccaagtgaggttcagaatgtgcgcggtcctagcacaaatggtggctgagttcttattttactagcatgtaatccaagtgaggttcagcaagtgaggcaatgccaagtagcaatcaagctgagcatagcagtatttttggttttagtagtggtctgtatgtttaatattacactgtaattattaatgtttaatcttagcaaactatttggattattggtggatttacaatgtcattttagtgcattttgcagtgtcatttttaa

>TaeST2.7901.1

atgatggagtccaggagccagaggatcccgaggatgattctacgtggagttcgacttccaggagtagtttaggaggatcccaggcaggaggcctgcgcctctttcgatctgtatcccagtttgtgctagcctttttaaggcaaacttgtttaacttatgtctgtactcagatattgttgcttccgctgactcgtctatgatcgagctcttgtattcgagccctcgaggcccctggcttgtaatatgatgcttgtataacttattttatttgtagagttgtgttgtgata

>TaeST2.7954.1

gttacaattaacagacatgtattgacctattgcggctatagagtatgcctaagccaatggccagacatcggctgttcggatccacccttaagccgaggcacaagtccattgcggttgattattgtttggggattcagtttcatggtgagatatgattttctggatcttgttgtcgctgaactgtgatttgttggtttgggaactaaagttggttgcttgcagcaaggggattcatattggttgaaaccttgaagttgaacatgaagggcacacatttgattttcattgggtgttgcccttgtttagcacattagtagatggtctcttactggattatggcagtagtagtgtggtattgttgtgaattttgttttcttcttttactagaccaattgtttcaaagaattttgagatgctacaatactgatagacatgtggctatcccttcattttatcaaggatgactattgttgatgaaaggacatttacatatttataggttgcaaccagatcaatgaccggttaatctaacacccggttttaaagagagaacaaagtaggaagggtgtgtgatggcgctggggccatgtgttggatataacatgttggatagtctggacaaccaaatttctcccctatatatgtgctggatttgggggagctctaactgttcaaacagttggaattttcaagagcccactgagagcctatttgatgtccgaacatgcccgggcgtacgagggagaaatgagcgccgaccttggggacaatcttaatcatttgtttgattcatgtatatgcattgtagcccgtagcaacgcacgggcattctactaggtgagttgaggcagacagcaagggccgaggttgacaaataaaatgccattgtgatggtaagcaaaatatcaggttctaattagtggaggactacaaaagttaaagaggtcttgatgagatgattgacatgacatgaatagttaatttgacatgtatcatgggcacttcttcattattcagttgtctcgcttgaatttgtggaccaactggtggacatagttcagttttcacaggtagcaaaagctgcaaaactgggtgtgctagattttgttcaaagtgcaacttttatttgataaagccgatcaggtttacatgtgtagatcaatgcagtaccaactgtaaaagagttacaacgcaaggggaagagtctccttttttgtattacactgggattgaacctgcatgttgctggggcaaccctcttttattttggtctttaactctattttctggtgtatctttgtttatcgaaaatagttgtctgccctgccctcgaca

>TaeST2.8244.1

cttgccggcgaatcacgaatctcgtcccaagttcagtactcgcatagccttcttcccaccgagcagcccctcacgaaccctagcttgctaaaccctactccttcccttccccgatggatccgcagcaaccggagcctgtcagctatctgtgcggagattttcaattcagctcaaactcattattttttttggcaacatatgatctttgctttgcatgctgtgtctgtgcaatttctcatgaactgtctccttacttgggcagttgttcaatatgaagctcgctgaagacaagaaacaaatatcattcactgttaagtgtgtgccctgagacaaagctactacaatggagaaatgtattaggcagtactcgctcctaggtagtaatctttccattggactttggcttgaagtggtaaaaacttgtcaaacgtgtattgagattttagacatttgaggattgagattatgcatcttttagctggattttaaaacatggttattattgctgtcgccaggaatgaagtgccctgttggttgcacgcaggctctggctgttatttgctgcctttgtcgttattactacctgtgtaactattttaagacgtttttgtagttcaaattgaaatgtaaaaatgtcttatatttagttacgaaggtagtaccttaaattc

>TaeST2.8573.1

gatctgatcggaaagaagaacaatacaatactatagccaagatgactgaaatgctgacatatgatgggacagaaactaaactggacacgcattacaatcaggcttacaaagttacaagtaaaatcttggctagctaatatgtgaagagcaatacaaaaacctaactaaacatcaactcaccggaaagatgaagagaataacaatactgcaggctagccaagatggctgaagcggtgacatgagatgccacagcaacctaaacacacgatcttattgggcaaagatacacagcagcgtcacacgcaaactcgatcgatctactgctagatgttgcagtcacaaaagctcaagagggatagacgggatgacgggagatgagagggttggtgtggatgagtatggggttgaatactggtagaggggtggttggcagggcaaagtcttgcaaggtcaagaacagcggttagtgcaacaatggcaagagctcctttgaacggttgaagaccgcaccaaggaacggtgacgcggaagagagggatggtgaggaggtggggcgcagaagagctcagggaggatagagccggcggtcgatcagcgcggcgcgcggcacgcccagctccccgacggcccctccttggatcctggaagcttgggcctggtcacggaggagagcaccgggacgaagacgaggctggcatggagagggccgcggaggctctgtgccggcatggggctgggcaccgtggcgaggcgcccagagggaagagctgctgcggtttgcatggcgactactgctgagagtggtgcgttgctgggagggaaggggagggtgctagctagctgcccgggctttatgtagcggaaggaaggataggtagagtggagaggaggaccggaggaccgtggcgtggagagggccggagaggtggtacggtggacataaatggcctgcgggggtgcaaaagggtggtagagcacggcg

>TaeST2.8637.1

actaggaacataagaagaagcagaaatagaaaaaagtagttccttcattctcatggcacacactaacagtacaagatggccaatggtggtgcgctccatggctattgctttcctggtgctggccatgatgttttttaccttcccatcctgccatgcgcgcactcctgcatctacaaaatatttatatcatggattttgcaagactctcaatccctgcaataaggacagctgctatgatctgtgtacaagtaaaagacaaaactacgcatattcctattgcaaggacgatgttaagattccaacgtgttgctgcacatacactgatggttctcctgcttgagtcatacaatgttgaatacatttatatttgatttcaataaagatgtttttaatcaatcgagttaattagggaggtcatagggcactctcagttcccatttcacttgtaccaaaaagcatgccaccaaggtttacatatacttgat

>TaeST2.8768.1

ttaacccatctcacgtttcaattggtatatcaaaatttatagcactttctcaaaaaatcaaattttatagccaaaattttacacaaacacagtagaaaataacattttacataaaagaaaaaaacatcacaaaaaaataaaattacatatattttcagccaatacacgaggagcaaaacgctagaaatccaaacaaacgaagtcttggagattctccgagctgagaacaaactatcctactttgcagccaccaccctgctcagcactcgacggacaacaaataatattttgagtagccgatcttgaccgacgagaacgctccaacaccattccagacaacaaatttgtgctactaacaatttttgtacagaaagattttatgctgcacattcacatgcccttctggacgtagatctagccatcttctccgacggcatcgaagggagatgccatatcctccaccatatcgcccagatccatgctactgagtaagcggatccatgctcatcggagcgagaaccatatcagccagtatcttgcctcaacacatgctcgacgttgcctgcgcgtcgacaatccttagggaagccggaaaaaatcaaaagtagtagaaggtagggagggacctggtggggcatgcggctcacagcccgtcgaggtcgatggagaagacaacgtcgaggacggcgtatgcggcggaaacgggcccctcggtgaagaaggggcacggcgcatcccctggcggcttgggaccgacagcgactgggcacagctcctcagccaccgcggcgcgactccaccagacgccttgatgcggcccgctcggtggctctgcacggcaccaccagatggatagggggaggtctggatcgtcgctgagctgttgctgccttggtctgtgcttcctccatcttcagacgatgtggtggagtgattggggaaagaaagtaggggtgagcagctcggagggtggttggggaaggatctggagatgtggcggccgctggggaaagatctggagagaagtcgacggctgggtaggtggaagaagacgagagagaggggcaggggtgtgccagccattggatcaagaggagcgaacggtttggatgctcgatc

>TaeST2.9120.1

gagtcaatgtacttccttttttgagaatcagagggttacgctagtcactgggcagtgggccgggactgcattttaccgcacagcatcatggtcccagccgtgcgttaattgcaccatcttaaattcttaatcgagcggacgagaggcgtttgaaattttccattactggcctgcgcattaacgcggaggtgccccgccaccattgacagagaggttgctgcaagacacagcagagtgccagttttgcagcgcctcaccagattagctagatatggctactggaaagtctggaaattagatgaatgaattacattagccaggctagcaagctactctagggccaacaagaaccaaagaactcggggattgttcgcctccgcacgctagttgatcgatggatggatggaccggtaggagtataggcttggattcggctagctgtagacggtgacgaggcggcggaagcacctggtgcaggagtacttgcgcttgttcttgaggcacagcgggaggcacaggatgcgccgctcgctctccacgtccgtcgccaccacgggaccgccgcaggacgggcaagcgccgggcgccttgcccgtgcccaccacccgctccaccttcgccccgacgatcatcttcaccgctccgcccacacccattgtcgccatcgatcgacgatcccctcgatcgcacgagaggaggaagaagaggggggagttggtggtttggatttgaaaagtctggg

>TaeST2.9387.2

aagaagctggccgggtcgggcatcagcatgggtaacgccgccaacatccccggcaagtgcggtgtctccgtctctttccccatcaacaccaaagtcgactgcaacacccttcactaagttatctgccagcatcatcatcacaccaggctagctagccgactcggtgtctactgttgctgctccttgcatgtgttcgctgttgttttctgcatgtgttcgacctccatctgttgcccctgttacagatcgagcaaattactgatcgaatcatcaataaaataatgtgttgagctttattttgtatgaaattcactcttatcaataata

>TaeST2.9387.3

aagaagctggccgggtcgggcatcagcatgggtaacgccgccaacatccccggcaagtgcggtgtctccgtctctttccccatcaacaccaaagtcgactgcaacacccttcactaagttatctgccagcatcatcatcacaccaggctagctagccgactcggtgtctactgttgctgctccttgcatgtgttcgctgttgttttctgcatgtgttcgacctccatctgttgcccctgttacagatcgagcaagtgaaataaaataatgtgttgagcttaatcttgtactcctatgacattggctgttatcaataatgattataaagtagtttcatgcccgcatattgctgctggaacttttagcaagtgttattaacgtgtttgataccaacaccgcacattcgtaaatcgtaattttgtaactaagggcaagggaaaaaactttgatgtcaacttgctgaatccagt

>TaeST2.9502.2

ggtgcccttcctagtctgtaccaaatccttacacaccgacgacacaggatcgaaaatttctcggcggcggcggcgacggcgacgaccaagatcctgagccaagctgggcgggctcgcgcagcaaagatgctgatccatctcctctccgatggctcctaggctcccagatcagctcgcggccgtcctcctccaccccccaccccctctcgcgattcagcactagctcaacgctcgtggatcctcgcctccttcccagccggccgctctctggttctgcgaacaggccgccacggttcttcgttctgcaggatccagtaatggagggtagcagtgcgatgtctccgctaggagagccctcaattccaatgatcgcagcatgtgcaccaacactatcaggtgtgagaatagcgccatggaatcggtcaacagtgttcaaaatcaaggatgtgtgtatgtgatttagtactaatccttcatccatggcatgagcctatctggcatcttgttcatttatattttcccgtgctgctgtggttttcttattattacaaggtagatcgaactgatcaagtgtccatggaggttgtcatctctctcccagcaggaagctcgattgccaggaagcactaacacgaggcaaaatgatagatattttcgttcttgtaccagcaagatgcttgttgggcgatgataatttcagtctttaccacttgttcttgccggccatatactaaaaatgatctgtatgccacatgtactcaagtactcaaatgcttgctaagcactgataattccagtccttaatttgctctacttccagttctactagccttttgtgatgtgttctcatgtactcgagtactgaaatgcttgccgagcagctatcaatccttaaattgctatat

>TaeST2.9551.1

atatctgatattactcaaagagtaggtgatggtagatactcactacagtaatcacccgtaaaatatctaaaaaaatggccctatctacatgccattggcgcatttgttatgcaatgtgacatgaaatacctaagcaagggttggtatcatagaaaccaaaagcaggccatagtattagtgccagataacttatgtacactgtttttcatactgcacaatagtaaactacatggttgactattggatatataactttgtccacacgccttcatccgatttgctcgatatataactttgtccacactccttcatttgatttgcttgcatgttgtgacacacagatctgctaaaggacatatcatgttaggcaaactatttaaacagtgcatctcatggacagggaaaaaaataatttggtctatatgaaggagaatcactctggataatatacaatgatctggatacagtgtagcctctttaacataaa

>TaeST2.9797.1

aggtgcagcggcagggaaagaacagagcttcagcaggggaaccaaattagggtccgtcagtaggctggctggcggcggcggcggcgacggcggaggcggcggcgacggcgatgtcttcctcctcccgtgcgtcgtaatcaggcggaggtcgcgggcgtagggctccatcaaggtcgcccgtgcaagaagcctcctccctggaagatactagtgttgcgtccatgaaattaactatggctcgatctcagggctacaatcaggaggtttgaagctgcaggtggtgatgactggcaaccatgattggaacagcgccaaagaattggaacaggggatgaactgagtcaacaagttctagagaagaatgcagagattgaaccaaaaatgttattggcaggtgtatttacaggatttgtactgcattaatagctagaagaagaatgtgatgccaaaatcttgtaatggctgaatgatgtaatggttggatgatgtaatggtattccatactactatttatgtggtaattgatggtaca

>TaeST2.10094.1

cgctccgaatgagaagaaaagaaatggcatccttagcccgcgccgccgccaccgccgcgagagcggcgctccgcccggcgcccctcgccggccgtggcctcggctcctccctgccgtcttcctcccccgcacgggccgcccgcctcctccgcaggtcggccgtggcggggctggagacgctgctgcccctgcacaccgcggtcgcgtcggcgcgcctcaagtcctgcatcgccgtcgactccacctgctggtgctccctctcccaaggttttaaaaagcgcatctgatcatggttaggagctgcacagctgagaagtgaagccaccgagttaaaaacaaagtgcatttgtttgctgatagtgcttagaacatgtatcccagctattttggccattgctcttgatgaatgtactcttaacaaaataatggagctttgcccccttttctccgggaccttgattttttgttgagcctggaatgatattggatatcacattattgcttcattatgagcttgaaatcatcctgtatttgctataagcttgaaatcaccctgcataattagtccttgtcgacttaacgtga

>TaeST2.10447.1

acacatgaaacacagcatagaccaatattactagtactacatccatccatccattcattccagccacacttaaaacacagcatataagtacggtagagtactagacatgcatggagctcatacatggttttggacttattgggcggcggagatccgctgcatacgtacgtactactagtaagtaggcaagtaccggtgttcctttcagccgtcgatgcagttccagatgaggttggcaaggtcaacgaggagcttgaaccaaccgttcattttgcgggcgtattcctcgagtcgaaccttttggccctctcttcgtctccacgcctcctggccttgcacgccaccacccgaagtgacccgtagacgaggccgtaggcggcgacgcaggaggagattgcaatgacggcaatcgcccacttgggcaacggggtcttcttatggttggactggggcggcgtggcagactgcaccggggagaggcttcgcaggagacggcgcgcctcctccatcttgatttgcgatcagctgctgaaccttcactctctcttctctcttggcgcgcgcctgcgtgagcagagtgaggcgatcgactgag

>TaeST2.10639.1

ttgaaagttatcagaaaacttgatttccagacaaaaattagcaatcttgatatccaaacaatatcaacaagtatgccttgttcaggacactgaaaatctcacagtaatagcacaatctgaaggtttccaaatactcatgaaggaaagatttgtaaacacaacactcatatcatatttattcgaagttcaaactgaatttgtcggttatcctttgatccttctcattccgagaaaaaaacatgtctcacatcttctttgcttccttgcacatgaaaccctccattgttgcacatatctgtagaacatcta

>TaeST2.10753.1

agcaactctcaaacagctccaaattaagaagatcgagggagaaaaaggcgaaccggccggagattattaaaaacatactaatatgcatgtgtaggtgcggtcaacacatagctagagaaactctagatcgggcagttcattacagctagatcagatcgaacaaccagacacacgcaacgcagcactacatacatactagcttgcacaccttccagggccctggagccgatcgatctggacatgccgtcgaggtaggatgcagctcagaccgcggcgccgcctgcgccttctgcctggccgccagctccgtggttgccgccgccgcctcggcaagcctgaacacccctcacaaagatcttgccgacccagacgaggaacatccccgcgagcacgacagcagggatgaacaacacaccggcaaaaaagccatcaagaaaacccttgtcgtacggcgtcatcgtcatcgctacaggaggaggtggagatggccagatgagtggagagtgtgacgcagagctgaacttgaacaaagaccaggccggggtgggagaaaggagatggaggggttattaagacgagctctctctctctctctctctctctctcactcactcactcacacacacacagcacacacagaggctcacggatgagattc

>TaeST2.11106.1

gcactttggtacctgagagcaaagaggagggtgagaaggtgagaagaaatgaaagaggaagaagaggaacaagaagagcaaggaagacgttgcctgggtcgatcggctggaagattgaaggtgttgcagagcctgctacccaattccaacaaggtcactccatttttccttctttctttgtcgctgaatttggtgctcctgcgtttatgtttgtgatgaaattaatgggttcatgtttgcattggtctgcggggttaggcatcaattcttgatggggccgttgagtatcagaagcaactgcatctccaagtataagtcattaagcatgcctggattcatttggatgatgatctcagtttgagggagatatcggtctcatcgatgacacatgcaagcggggtaaaacttgagtttttaccatttgtcagagcggaggtttttctggggttgtgcatgtcgagcctgtttggctgtttatgagcaggctacactatagtcgatcaagcccttgcaagatagtaatgcatttttgcattctaggttacaatggcgataataaacaagacgaccatgcagatctgatagagaagagacccaccccggtgacctcctacaaccatggtcgatggcagccctagcaacgtgactcgcggtcacaggagaaggatctccgtaccctaccaagctcgctggagacggatccataggagcacaaccagcagctcaagtagttttacccgcgcaaccatcctgctaatttgctggacctgaagaataccgacgagtcgacggacacaggacgttcctgcaggctatgtcctccgagcctcgtgaaatcatgaggacagaagcttccgtgcctacttcaactctgcaggaccaatgaagcttgaagtttacaagttgggggatgccagtgatactgatcttgcacgctgcttgctgctgtctacttgatgtttttaaatttaagtcaaattaagcgtcatgtgtcgttaaactttagttatgtccagtcttgcgattttggttcgtgtgcctgtgtgctaaactactgttggatcttggatgctatgttttgagtcaagatatgattatgtacaatctatctattgttaagaaacgatgagcaatcaggtttgtttcgataaaaggaaactatggactatttttttaggcgaggaaaa

>TaeST2.11495.1

gattatcttggaggccagatctgagcgaggtcggggcacgcgttctagccgcggatttggcgctccctagcagcgaggcaaggcggtcgccggggaggccactgaagaatatgagtgatccgtagttggtaacttccctaatggcgctgctatcctgttcgtgtcactgggccaaggattgtggtgtcgggtttgacggccatgttcttcatagtgttcctcatcttatgtggcagagtagtgatggcagtgggctacgctgggaaggcaacaggatgctgatctccatcgacatgccatcgctcggctccgcacagtctactcggtgtcccgggccccgtcgcctcctccgtctgctgccttatagggataggcggcccttttggtcctgctttctttgagcctgcgtgctcgctctgctggctagctatgccatgtaacgcacttatcaccggtctcgccttttggctctgcctcgaaacttggctgtctagcttcggttccgtttagtttggtcttgatactttgtttgcacgctgcctattgttgtgggctttattaatttaaagccggacgtctctgacgtctatgttctaaaaaaaa

>TaeST2.11601.1

taaatatgagcaaaacactgtaattagggagatcaatctatacgtacattattgtccaagaataaacatatctttggagctccaacaaatcctaggggctagggagtggccagctcaaatgacctcattcaaaactcaccataaatcacacggaaaacccacttaactaaatagcacaaaatatggtatatttgcgtattcatacggaagaactcatggctgcataggtcaagaaaagagaaataaaacagagcacctgcttctgttgccgcttccatccatctgtgacgtcctcacctctcctccccaattatcagacttgcgccgccatcacaatatcaactaaacagatagagagaatactccaacacccaacccggccttccactatcaaagcttccgccctagagccgaccagaacctctctttggcttgtctccgatcccgcccctcgactcatattcccctccaaatctatgaactggagggagccggatcccctgacgtacggccccctgccgttgggccgctgacgggtgggtccgggcccacacgtcagtgtgtgggccgacgagccgcgtccgaactggaggcgttgtccagatctgggggcaaggagacggggagaacgtcagggttgctccctgagctggatggtggtgacagcggacagagacatggctgggcaagcagatttgtggggcaacgctgcgacccgagcgaggccgccgacggtgcctagcaagacgaagaggaaattcgaaagagagagaaaggagagatctggggcttggatgcgtggactggggtaagtgcgaggacaccgacttaatcaaggacccacagcacagaggccttccagattccagccatccactct

>TaeST2.11641.1

tgtctttctctctgtcaccagtacgccgcgatggaaaacgttaagattccaacaaaagttcttctggatgctattatgcaataggttggccttcaagcgcaagttactcaacagacaaggcaagcaccgttggtcttgagtcaagtgttcagttctatccatctgagtaccggttgaataatggaaagtattgatgactacactcggagcaagccttgtcgcgacgctgcaagtgttgcccccctcctcccaaagggcaaggtgaaggaaggtgcctgtgtggttcttctgcagctcgcagcgaggattgttgtgtccaacctgcacaagaatgccaagaagtccttcttgaagacgattaaggacatgtacaagcactacaacgagagatctgggttgcttctttgtggcttccaaacttggaaatgtttgtctgaaagttagtttagtttcttgtttgtggattcacagcttctcacatgtaccatcctcttagacatttaggtttccaaagaaattaaatgaagatatggtggtatggtctcaatagtacaacttaaagcctttcatgttttatactttctctgaaatttgctcttgaagaatcatgtatctatgttagatccggtgagaaaaggcggtttccaagcaga

>TaeST2.12031.1

atacaagcccccgacgcctactcctccggcgtacaagccccccaccccgagccccccgcctccgccgtaccaccactaagaagatccaccactctgtcaagctgggagcccagaatgcggtcgagaagagttgtgcgagcaggccaagaagagccacgtacggcgtcgttgatcctcctccggtgtgtgttgctgccgaactagggccgatctctcaggagatggaataatgcagatggctcccattagtttatcttaccagcttatcatttgtgttcgtccagtgttagcgtctgtgttgctagtgtggcaatgtgcccgtgtcactgtaaaaccattgatcaccacggaaatggaagtaaaaggtgttgtttcctgttgcatgtaacgtgatcgcgtctctgttttgctgctcttctatcatcttggc

>TaeST2.12070.2

caagttcagtactcgcatagccttcttcccaccgagcagcccctcacgaaccctagcttgctataaaccctactccttcccttccccgatggatccgcagcagccggagcctgtcagctatctgtgcggagttgttcaatatgaagctcgctgaagacaaggaacaaacatcattcactgttaagtgtgtgccctgagacaaagctactacaatggagaaatgtattaggcagtactcgctcataggtagtaatctttccattggactttggcttgaagtggtaaaaacttgtcaaacctgtattgagattttagacatttgaggattgagattatgcatctttgaaagctagattttaaaaaatggttattattgttgtcgccaggaataaggtgccctgttggttgcacgcaggctctggctgttatttgctgcctgtatcgttattactacctctgtaactattttaagacgtttttgtagttaaaattgaactgtaaaaatgtcttatatttagttatgaaggtagtaccttaattctcct

>TaeST2.12235.1

tagataaacctgtggtgtttgaacgaatacacatgataaataggggacagatcgattgataaaatcatagcataataaattaaggactgagatgtagcacataacataacgtggcatgacatactttagcaatcagcagcagctcacgtcttgtcttgcataagcagttatagttcctgtaaaaataacacggacgtcgattcggaccaagtcgcttgattccccatggccatggcggcgtggcgtacgaagaggaggcagcgcagctctggaggaaggaggagggaggagtctagaaggagatcgggtgcatcgatctgatgatggtttcttgactcacacgatgagcactagaataaccccaaaaccagatcatcatcatgcaccatatctctctagctagctaacacctcacctctggccccttttatcttctctctcctccgcccccctcctacacctgcggactctctttcctcatgaactatagctcccccgacctctccttcttggaagtaggtgccggaggaaagaggaggaggtggtggtgatgatggcgaagggcccaaggtgctccaggcagcatgattgcttcttggaagttgggggatgaggaggagagggaggcgctcctagcatcacatggtgtggaatgatgaaggccatc

>TaeST2.12360.1

atataatctagcacatatgatagattcataaaattgcaacgcaatttgaacaatttgtatgtatcactcgaatttaatctctggatttacatgccaaatcgccgaacaaactatgtgcacctccaaatcggcacaaacaattgggtaaaatgcatctgcgatactggaatcaacactaatattctacctggggaggaaacatcaagcattgaagtacacatagcattgaaggatgcaatctggacaacatctgaaagttttctatcttagctctgtatagtccaacaatctgaaaggattccagaaaatcaatctaaccacgtagcccaaccagccaagtacacacccatgcatcattaagtagaggaataaaaaacctgagagttggagggatcagagttgctttgctgacggagtaccacaaccatccttgtatgatatattgccaaagttatccctaacaaacatgtatcaacagtcgactcatatgtaacaaataggattcagaaaaataatccatttgtcggttaggttcctcattcgccgaaatgcctgcggattatcgcccttgaatcaactgctgagctgagctgggagactgggtggagagttttacacgaaactgtttttcgctttaaccaaattccctttgttgtctttcctctctctgttctttcctcaactggaacttcactgacaaagtttggattgcctccttccaccatctgcacaaagcagtcttccaaggcatgaaatctgtgtccaaattgtttag

>TaeST2.12412.1

acagcacggtcacacccgaaccggtgaggccttatctactttcatgacaaaaggagtgaaatgcacggaaatgcataggagctctcgggtgctatgcaggggggtagagcacccgagtgctcataatccgcttcctgaaatacattgcaatattaatagttttttccatcggatgcatgttttagatacagagtagaaactactcaggaaccttgtttacaatcagatttaactgccccatgtaactccgctgggatatctgcaatcttgagtatgttctctttagttctggcacacacacaccaagggcatgggcgatggtatactggttctgctgcccggacttggacaagaacagaagagctggtcttaatgtcgcatgaagttgggaaacagacattgttgtacataattgtcagagctgtcaatccatctagcatcgcctgagcctcagcctcttccacagaactacatttgtcaatgctcttggataaatagaagaaacaccaaaaaaaatccccggcgatggccccgatatagctcctccaagtctgagcaaagaatgaagcatctagatttaacttgattgttcctcggtcagggggaatccagtgcctcaaacttccagctgcatcgccactctgtctgaaaattcagagtaaacgctgttttcactctcttcatctgactgaacattgagtgcttatcaatgtacctgatccaccaatatattttcactctcttcatctgtctgaacattgggtgcttatcgatgtacctgatccaccaatatattcagcatagtgtggttctctgactgaataattgatagtactgtagggtgctctattgtgggaagtgaactatctggaattatcaatactctacacctgcattctcgtaaaagttgtaaatccttgtgacatgagacaataaatgcatccagaagccctccacatgtgcaatcaactgcaacagaccaggacttgttagcaagttgttgcatgacaaaaagcattatgaaggtttcgaagaagtaccgcatccataatatacattatagaaaaaggcatactacatgaaagaaccagctatcgtttataccattcctttgcattataaagtgcagtacccaaactccgacaaggactacaagcataattataacaacttagtggcatcatccctgatacgggatagcatgagacaacaaatctattttgcattcatgttaactgatgcaatgagaagaaatattaaagttactccaatgaactggaatcaacagcagaaaggcttgagcaaattgatttattttctgaagg

>TaeST2.12622.1

ttcagaaataggactttctctttttagtgattttcttgtaagcaaacagacaagtaacactagcacatggaagatggttcagacatcacacacaacgcatgcaaccaattaaagtgacagcagctcacaagagaataactactaacttaatatccagcaaaggatcacaacaattcagcaaggatacatgagggttcagcaaaggtgcacatgtaacagagtaaatttcagacccaacagtgcatatactcagagaacaggaacaacagaagagagaagttcagagcctatgggtcttgatgatcttcgattcgaagttgtagatgttgcaggcgaagaagagtagcagaggcacattggagacgatctgtagcagcagaaacttctgcgtggaagttgaagaagagtagcggaggaggatccttggggatgagctgcagcgtggtgaagaggacttggtgaagatcagcagcggagggcccgaccttggggaggaatcgtggtggccttcgcgggaggtcagccgaccacccgaagcagagaaccacctggtgtctggcgccccgcaggagatgccgcagtggcggtcgtcggagttgaagcagagcggcgcgggcgtcgatgttggagcagagcggtacgggcgtaggtgttggagatgagcagcagcagtcggtcaggatggagaagcctcgcagcagaacaccgagaagtcgttggagaggatcaacagctccgtttcttgaggttggggatgaggaagtacagcgggtcgacgtcgtcgttgaagaggagtagtggattcgatctggttctccttctcgcttccctcgatccccttttctccctcctccgatctggtgttcctttggttcgatctggtggtaggtgagcagcgtggagagcgagagcagttagccccctggttgtttttccttcgacccccactcctcttcttggtggttcagtcgtgcgtgggatcagacaccacgtctggtgtgtcgcgccagaccccagctcctct

>TaeST2.12679.1

ccatccctcccactcgacccctcctctattctgtcgctccgcctagggttcctcctaaccgccgccgccgccggtcctcaggtcccccgctcctcgacaaggccatctccggcgacatggccaacaggtaggcctacatcttccaccccagctcccacgggcgcggcccctccctcgggcgcatcccctccgtccccggtgtccagcaacagctcctggcccggtccggccagggctccactccggtggacggcgacccctttccccctcccctcctcctggccgtgagccaccaagaaataattttgacatggatgtattgattgtaccaaatagtagtacaattgttgccctctggttgcatagaagaaatattgacatgatgaaagcacaatgggataatgtggagagcctttctcaccaccatgcacccattgatgatggtactatgggttccatccgtgatgctattgctacaagtgttttcccttgatggttagctctagggtgtatgcttttattgggagaaccatgtaataatttacagatcttttttattcagtacttcttctcaagtactcgtactacgtgagacagcgatgcatgtatgatattcgtatgcaagacaatgatcttcctcatgtttgattataacatgttgtggtgtttgaaagtcaaatttatgttcatatgggcaacaaaatgataaataatagtgcaatatcgtttgatctgaa

>TaeST2.13123.1

tgaacgactctgaacttgggactttgtgattagagtgacagaaacagaacatcgatgatgcaaccagttgagatttgcatccaagaagacgatcacactaacatagtaattattatttattgatggtacatgtagcacatcaaggaacgaaaaatacgagaacatgaactggaaatagatagtacagatgacagttgatacaggcgctcagtaccgaacatggaaatgagcaaaacgtccgctttttccttcccggcggtgctacctacagaatctaaagcgacaaggcgagctttagctccttgtaagccagcacatgagccagagatgatcatgggccaatagcagggatcccaggaagcaccaccagcgggccggtaatggtgctggttcccgtaccggttcccccgagcaaaggcaagtccaacgccttagactcgccgacgccaccacccgcacctgcgccacgcgtcgctgcaaccttctcattcagctccctcccttcgcatgacgcgagcaccaccagcagcaggagcgcgacggcgagacactttgccattgctaactgggaggcggctctggtaatagtagtactactacagaggagatagtgttgtgtggctgtgtttgagtgagcttgggcttagcatcgtgtactctgcctcttataggtgagttgcacggggtggcggg

>TaeST2.14007.1

ttttctgtccctcctcctgtgtgcggcgccgccgctgcgcgagcgcttccacccaatctccccgccctcctccacagctccggccaaggcgctcacttctccggccctcctccatattcctgacctccacaccatccacttcctgtcctttgcttctccaagactgcagcatggtgctcggcggctgcggcatcggagctgatggtgcgcgagccgaccagttcaacggaggtagtagcacggggcagcagcagcagcatggagggcggccacttgatcgggaaggtcctggaactgcaaataggagtggcagtggcaatcacgatgcaaatgcaagtggttcccaaagatcttgcaaaggtattactatgctatgcttgttcacacgatgcaaatggaaccatatgatcttgatttgctaggttcatttaaagtgatgttgtttgtaactttgcaatgcagaaattaaagacaataacttatatgatcc

>TaeST2.14096.1

gggataggaggaaaaagaattaagaagctactcctcatggaacatatgaagaacaacacacatgctttgtgtttggtagttcttctgatgatgtccagcactttatggtcatgtcaagcaactgcaagaaacaaatgtacaactagattgtggacttcatgtacggttttccgagcatgccagacaccaacgcccaacaatgttgctatgtgcaagattagctatgaatggcgtggttacagctttgaaaaaagttactgccacggttctatatcgtgttgttgcgtatggtgaatgctatgggaatgatggatacaatgtactttcatgtgccaaaaccttgtgctagtaagatatggaaacttacaataaattatacaaactgattctggctcaactatgcaactcaatgaaattatacaaatctcgatttaa

>TaeST2.14170.1

acgctatctctctctctctctgtctccaccatcaagccaggatgaggtcgttgtacaagatgctactactttcgcttgcccttgtagcgctgctatcctcagatctgacgatccaggcgacggctaatggtggcggctcgctaatccctgaagactgccggcagacccttgtcatccgtgggccctgtaaccccaaggcttgtcaacgcaactgtcaaaccaacgtcggccccggtgccgttggcgactgtgatgccggtggatgccggtgcacatactgtactccatctcagtggaattaagcagtgattgttgctt

>TaeST2.14228.1

tgcactaaagactgttatagcagaccagaatcatgacgagaagcacaagtaaatatatgaaactgttgtctggttgtatgtgagcacacataatttgtttatttatcaagtaagcaacacatagtgagtaattttcagaagtgcatcagcatcatatgcaacactatagcaatgcagctggtcctctgctctctgaaaaaggttccagttttcatctcacacctaacctaattaattcacccagagcaagagcagagcatgagagagagagagatcttaaagagtcggagcagcggcggaagaatcagtcagacctggtggtggtgcatggtgcatcaacccaaatcaaaaccaacatggtcatggcggcaggctgcagacctccatggcgtggttgtggacaaggatggagcctgccct

>TaeST2.14251.1

aagaaattgtatgtaggtgagcgtaagcagactcctgccctactgtactgtacaccatggcgacgctgaagaagggcacgggcgtcctgtgtttcgtggccaccgtcattgtggtcatggctaccacccttcttctctcctcgtgcgacgcccacaaagaggcagatgagactgctgcgtttcccttgccggtgccatgctactccatctctttcccaaactgtaccgacgacaagtgcaagaagttttgcgacagcgtaggcaagcggccggcccccaaggcattctgcaatgacaacaataactgttgctgtcctgtcattcaagtataagcgtctgctgaagcagctgagctaagaaataattaaataaaactatattcgtaattgtttttgatctgggagcttccagcatggactgttgcccggactcaccaagatgtttttaaatcattgtaaaccgacttttcgataatttgttgtgttcggcagcttttgtttatcttgtgtgtatctctagttcgtgtatgtggagaccgattgaaaataattaagtttaaccaaaatggttgatttgtttttgaaaaggag

>TaeST2.14335.1

aactaagccaacatagaaaccattttctctgtaaaagaatagtagcttggaagacttcaagtagcatcagacaaacagagagatggcagcaacgtttccttccatcgtgtgcaagatccttctcgtcattgtagccatgcttgccctcctttgctctgcctatgcaagagacgcatcagaatatgggcagtgtttttcccggcctgaatgcagcaactactgcaagcagcaaggataccacaggggaggggaggtcatgccgcccaacttcatggactgttgctgtctcatatgagcattaccgtgtaatgttccattttatgtctaaataaaataaaatgctat

>TaeST2.14810.1

gagtggcgaaccgtatcggcagtatcggtggcgcacggcggtgccatgggtaggtggggcagctgcctaggtcttgggcggcgccgtggttggtgatggcgttgaagtttcagcccgtgctgctcggtggcagtggtggcctcggctgtgtggtgaactctctaggttcctcgaggcagtgttgcggggctgcaccgctctacgccggcaatagcggtctacgacggcgtcacgaatgaagatgcttgattatgtgcttcagaatgaagatgcatgattatgtgcttcaggagctgctagtcgtaccatagaagcaattaatgaacctcttctcgctattttaatttcaagtgcaataatctgctggaggtgttttccattcagacaagtgaagttttgcaccctgcaaatgtgcagatgtattctggaagaagaaaagcatacccagtaagatgcaatccttgccatcttactgagcaagttcattcatattatttttaggtcctccttgacaatgtatttgatcaattgtctaaagaattcctggattgtcagagtaaaataaagtacgttcagaata

>TaeST2.14816.1

ttttcagaagatatttgtttgtccttaaagaaaagaaaattacatatacacactgcaaaatattacaacaaacctactactaaatcctttttacaatcattttttgatagtaacattttacacaaaagaaaagaaacaccacgacaaactcgaaattacagacatttctagaaaaaaatacaggagcaaaacacacataacttcaatctataggaagtcccggagaacgccatgtgctgctttccaagcgcaaccggtccgctcagtgccgcctagtgctcgacgaatggaaccggagaacaccatgtgctgttttgcctttctggaggaagacatgcaaggtaatggataagttaaaagaaaggttagtgtagcgggacaaacatcacggtcaaagcgtcggcatagatctgcgaggcgatggagcgggcatgttccccacgcgggagatgtagggaacgaagaaggccttgcggatcccgtaggagcaggcgcgggcgcagcacgaggccaggagacttggtcggcgggctcttaggacggcaaacttgaaggcgagctggtcgacggcgaagagcacgacaagagcttgagggcatgctgaggttgatggagaagccgacgatgaggacggcgtacacgacggactcgggaacctcggcgacgaagggcggcggcgtcccaccacggcgcaatccgtggcggcattggtctggcgccggctcggcgcagcccccttggccaccgcggcacgaccctatagggccgctcagggcggcaccaccggatggagaggtggaagcctagattaggggaagaaactggtggctgggaacgagcggctcagagagtggtgggagatacgcgcggtcgtggatcgggaagga

>TaeST2.15104.1

atcgaggggtacctcgcgtctcacacgcctcatgcgggagggagaagagacgcggccgcggtcacaagagacggcgagaaccgcgcgcagccgccgtcgtcgctgctaacgccgtcggacgcctgcttcctcttcttcctagggaaagctggccttctcccccgagtgagtgcggtcatgtggcttcggagcgaaatggatgagttgcctacctagaagagggatgcgatggaactgaagtatgcatgtgcgtcggcgtcgaaactgaaactatgttgggaagcagaaaccgaatttaagtgatgtttgtgttgcaccctaggtgttgttggtacattctgatgcatgcctgtgtatggcctttgatgttcttagtttatatgccattgttcttaaagattatgatttgttgctttcagtagtacttgatgatatggccattgatgcttcagcgtgttcagcagtaaagatcagattttaatcagtgtgttgttgaagttcatgtttaaaatggaccaaatttgtccctgca

>TaeST2.15140.1

tatcacaacacaactctacaaataaaataagtcatgcaagcatcatattacaagccaggggcctcgcgggctcgaatacaagagctcggtcatagacgagtcagcggaagcaacaatatctgagtacagacataagttaaacaaatttgccttaagaaggctagcacaaactgggatacagatcaaaagaggcgcaggcctcctgcctgggatcctcctaaactactcctggtcatcgtcagcaggctgcacgtagtagtaggcacctcccgagtagtagtagtcgtcatcgacagtggcgtctggctcctggactctatcgtctggtcgcagcaatcgggtatagaaaaggagaaaaggggggagaaaggcaaccgtgagtactcatccaaagtactcgcaagcaaggagctacactacatatgtatgcattggtataaaatggattaagggtatcatatgtgggctgaactgcagaatgccggaataagagggggatatctagtcctttcgaagactactcttctggtaacctccatcttgcagcagaataagagagtagatagtaagttcaccaagtagcatcttgtagcataa

>TaeST2.15148.1

cgttggctcatgttataatcttcatccatcaaaggagcataagttgttaggccgcctccacagctgcaatctggtcggcgaacacctaaagaaatgacagcagcgagcccatctagtagatgtgcactctcttctacaattccatccaattctagatttgcaccgtgtagcttgctattcatgtgccagagccatcagttggccgcgagatcttatgggtttcacatctagcctaaccccaacttgtttgggactaaaggctttgttgttgtaggctgccccaacaccaccaattggagatccaacaccatcttcccatgtttaagacaaatgcacagtccaaagttggtggttgggtgtgccacatatggagttgtcagacatcaagtggaacattcaataaaagaaacacaaatccattagctgaatatataagcctacactttagctctgcagtaacttctttcagaagttcccacactatatattcacatgttgccagttttcatgtatcgtgaaacatagtacaacgcaatttctaagcattgtgaaatttaacataataatgattagcgacagtaatcttgcatttctttatttccttagctagcccatgattccttaaactggaaaacttgttgtctagataactaaaagtgtttggaaggtaaacttgacttctagtattaagctaataaaattgtagaaattcggattagtttatacctcccatggaaaatgttggcaatatgaccttcaaattacaagtcaaattttagcattattttacctttacttatccacaaaggagaaatttaatttgacaatgtgaaactagtattctataggctagaggattcatggtaactaaacaacataaccagcaatcactaactctaggagacaaaattccctttcaagatctcctaaaaataacctttgcctaaattcagaggacaattaaaaaacatcctgccatccactaccaattgttacaaatttacgggaggtaatacaatataagataaagaattttcataaaattcaacaaagattgggagagagaagatataacttcatgttctgctggtgacaatttgtggtataccggacgaaatagaatttctatttatttgtcatattccattgctacatagtgaaatgttgccaattcaattgcataagtacagtcaaagaacaatttgtaataaataaagtactacttggttggagttttgataatcgtgggcttacccatggtacatatgcatcaaatcccaaaacccaaatagataagcacctgcagaggtattaatccttttcttttggtggtaacactcaacgacgtcactatgctcgaaaagagcaagcacatccacacacacacacacgtcaccatcgccaccatgagcgacaggatcaggcgcaacaacccgtgcctcggcgtagccaccatgagcgaaaagaccaggcacacccacccacgccttagcctcaacatcgccaccatgggacacaccagcagaaccatccaccaatgcggcggctgc

>TaeST2.15273.1

gctgtctgtcatcttcgtctccggcgtttctcaaaaaataaaataaaaaattcttcgtctccggcgagcaaatcggcgaccggggcaggatgggctacctatggagggtgcggctctcgtcgttcgcagccggtgcggcgacggcgtcagcggtggggttcttgctcctttacaaagaccacctcctggcccgcgccgcaatcgcccgacaggtggaggatgtcaagagaatttctgaaaagcactatgaagcactgaatcatcagatctcagcactggaaaacagaaaagaatcaggaactaataaagaggcatcagattaattatgcacgttggttgtgtacaatggttaaacaactgccaatgtaatttgtggttgctgcatgcgtattactgcagcatttaagctgtaccattttgttgttcaaattgctatgaatcctcattgtcatcttcatggctttgtgagatggacagagaccaataaaaaacaattttgtcgttaatgagtcggcctatctcactgttat

>TaeST2.15600.1

tggatcaaacgaagagttaccctgcgtgcgccacacaggcaacagttcaggcaccgcaccggcgcaccgtcccgcccctctccctgcgcgcctgccccgccgcccgccacgtatgagaccccctccccccgcgccgggcgccggcccagctcctctgaggtaccgccgacccgcctgggaagtccgccgctccgcccccaagtcctggcttgacgggctccggtcaggatctcgctaggcgcggctgttgggagttcccgagtgtaacgacacagctggtgcggcggtggtgcgaacagatgtggagaagttgacaaagtcttctcatgctcttacggagaaaggagaccaccgctgttccgatacccaagtagacaaagttcagaaccgcctgcaagaacgaaatgataacagggtgaaaatttattcctgtctttactgtcttgcagcaatgtaatatatgcaaggaaataaatctaactatattatggtaaaactcaggtgctagaaattgttgcagttt

>TaeST2.15694.1

tcccctctcccactcacgtttatcctgagaacctcgtgcccgagctaccgtcgatgcgccgtcgccgtcccggtggccactggcctccccggcgaccgggagcttgtccaggaacttcctagtcgccgactccgtcatccccgtgcatccgttcgaccggagccgcactatacactcgggatcgagctgattttccgccacggccgtcgtctctcgtcgtcgatctcgtcgcatccggtcgcccccgcgcctccaatccacgtctacgagcttagtgatggtggagcttaggagccaggccacaccaccgacgattactactacaccgagggtgcctactactacgtggaggccgtcgacgaccatgagtaattaggaggctcccaggcaggaggccttgccttttcgatcgatgttgcttttgtgctagccttcttaaggcaaacttgtttaactcatgtctgtactcagataatgttgctttcgctgactcatttgtattcgagccctcaaggcccctgacttgtaatataaagcttgtattattttgatttgtgtcta

>TaeST2.15754.1

ctccttgcttgcgagtactttggatgagtactcacggctgctttgctcctcctttttcccctatctatactcggttgctgtgaccagatgttggagcccaggagccaaacgccaccgacgatgactcctactacactggaggtgcctaatactacgtgcaagccgccaacgacgatcaggagtagtttagttggatcctaggcaggaggcctgcgcctctttttatttgtatcccagtttgtgctagccatcttatggcaacttgtttaactcatgtttgtactcagatattgttgcttccgctgactcgtctatgatcgagctcttgtattcgagccctcgaggctcctggcttgtaatatgatgcttgtatgacttattttatttgtagagttgtgttgtgata

>TaeST2.15854.1

ctgtgcgcacagatgatgaatgaagatcgggtgccatggtgattgatgttttgagaagttgttgagtcagggcttaagaacttgagaataaattcttgcggtgacaatggatctattccgatactatgttgttatatttactaattgctccttttctacttgatgaacctgaaccatgtgaaactcgtgtcttgtggggctcgagtgagcgtgtttttatgcgttatatcttccgatgctttaatttggtatatattccaaagcttatgaataaactgttttacccatgtcacttggtgtat

>TaeST2.15857.4

cgccgcgtacagcccagctaagcagtgtagggttttagccctcgtctcccctcccactgccgtgctccatcccacacaccccgccgcaatggccgccgtcgccgccgcaagatccttcctccgatccggatctgctgcctcctctctccgcggggctgccgccagagccgccccccgcgctggctcagctccgctcccgaggcgactcccggcctctgctccccgcgtcctcctaaggtcaccggtggagatgaccagcttctgcgtggagtcgctgatgcccatgcacagtgccacggcgtcggcgctcatgacgtcgctccttgccaccccggctcgcactgggtttggttggctgacagaagctggcaatgatgacgtgtgacagtttctaggtggaatgtagcagattataatctcctcacctcaaatattcagatggatgatttcttcttgtcggagcctgctgtgatggtacctggtacggacctgccaaattgaatttgaaagtactctttggtcgccttctcagtctgtactatcctgggattttcgtttgtataagtgtctacgggattattatacctgattgtgccaagatttaaccgacttggtctgattatgccttt

>TaeST2.15857.5

cgccgcgtacagcccagctaagcagtgtagggttttagccctcgtctcccctcccactgccgtgctccatcccacacaccccgccgcaatggccgccgtcgccgccgcaagatccttcctccgatccggatctgctgcctcctctctccgcggggctgccgccagagccgccccccgcgctggctcagctccgctcccgaggcgactcccggcctctgctccccgcgtcctcctaaggtcaccggtggagatgaccagcttctgcgtggagtcgctgatgcccatgcacagtgccacggcgtcggcgctcatgacgtcgctccttgccaccccggctcgcactgggtttggttggctgacagaaggtcaggacgaaactagatgaacatcaagccccaaaagatggatgatttcttcttgtcggagcctgctgtgatggtacctggtacggacctgccaaattgaatttgaaagtactctttggtcgccttctcagtctgtactatcctgggattttcgtttgtataagtgtctacgggattattatacctgattgtgccaagatttaaccgacttggtctgattatgccttt

>TaeST2.15857.1

cgccgcgtacagcccagctaagcagtgtagggttttagccctcgtctcccctcccactgccgtgctccatcccacacaccccgccgcaatggccgccgtcgccgccgcaagatccttcctccgatccggatctgctgcctcctctctccgcggggctgccgccagagccgccccccgcgctggctcagctccgctcccgaggcgactcccggcctctgctccccgcgtcctcctaaggtcaccggtggagatgaccagcttctgcgtggagtcgctgatgcccatgcacagtgccacggcgtcggcgctcatgacgtcgctccttgccaccccggctcgcactgggtttggttggctgacagaagatggatgatttcttcttgtcggagcctgctgtgatggtacctggtacggacctgccaaattgaatttgaaagtactctttggtcgccttctcagtctgtactatcctgggattttcgtttgtataagtgtctacgggattattatacctgattgtgccaagatttaaccgacttggtctgattatgcctttcgtggagaaaaaaaataatgcttagttggaagtttctgctttgttttgcttctgctcttacgttatcaagttgttggagaaaaatgtactccaatcctatctctagctctccgtacctgcttacttttaattttaatgtatttagcaatt

>TaeST2.15858.1

atcatcgcaagaacaaaatgttcacatcatcagtgtttaaaataaaataaaaattgcaagctgaaaggtgcataatccatttaggagatcaaattgtatattactgctcagcacatcacatttgttctcagaaacacaactgctgcagcaactaacaatcactcatagatagaaatgcttgataagaaggaacttgcggtagttcgagcgaacaattccaatatgaactcagaagatccatctttcacattctagacaactcaacaagtagatttgaccaacaggcttgggaaagcacttctgattaagatcgatgtgattttcactcttcaacattgacaagttcatactccacaagtgaaagattgttgtcctcctttacaccgaagcttgctggttcagttacttcaacacggccccatttgtcaacagcaagcctcattgaccctttgaacatatcaatctttgcattgcggataatgacagtgttgcca

>TaeST2.16158.1

aggcccgtagccccttcgccgtcaggcacagcactggtcgccggctcgccgccgtcgccccacttccactccacaccagggcaccgcgccacgggcaccagtcggggaaaaaccttgctctgctgcagtgctgcgctgcccccaatccgttgtccagctccggtttcgaggcggaggccgagggggaggaggcggaggcgcggacaagtcgcggaacagcgccgagaattagatggagcagagcttagaaaggtgctggatctggaaggggaaaagctcagaggatatgacatgaattaccatcggtagtagactggccatgtacttgaatcgtcgtgaccaggatgagaagcagcgcaaggatccatgtcagctttgtacttgctgagcaactgaagttgatcaccatggtactctgtcttcggttcttgcaatgttggtaagcaatcatgcaaattaagaagcttggtatctgcaacaaatgacttgtgtacgcagttatatggccgagaaaaccaagtacagtaacacaatttagtagtatgcggattgacttgacttgacttggcctcggcgctcaattaacaatgtggatgaaacaaaacgttctaatcatcagtagatggccttttcatgtagcaaatgtaacttgcatttcacctaaaatcggaaaatgcaacttgccaa

>TaeST2.16213.1

gatcatgaccaagcatgacaagaagggaggctcgtcaaccacctaccagcacacctatcatgaccgatgatgagacgctgaaggttacgagagatgatgctggagagatgcccttcctgtaggtctacacataagcggaagttcatgggtatgcaagatttgatggttaaaatggttgatgtgatgtgaatgagtgtatggaaactggttgttgtgatgtgaatgagtgttgtagtttcacgtgttctgttctgtatagcatattgtaataaacttattttagctgttatttgaagattttcatatgctttctctcttctgtctgtttgatatatacatatatattgcttattaataagttgtgaaaaatctgacatttgggaccca

>TaeST2.16411.2

tttcccttccccatttctagcagcgagatcttggaggcagagaggggagagccatggcgattttggctggttagctttgtcgttgatctctggagagcgagccggtggttaggcgtccggtcccccccccccaacccccctgttgcttctccttcaagcgcgttgaggcagaggagaagttgacgcatggtgattcggcggcggtgtcttatctgcttccattgatttcgtgatgctcctccttttgctgtggtgttcttcgagccttgtgcctcccttcttcccacaaggaggcatgattgtggtgtcttcactctgaagtatatggagatcttcacaccaagaacttagatggcaaatcttttctccaatgtagatattcgaaatcttaggatcaaatatgtgaatgacatgtttttcagccccttgaacagctgcgacaaatcttttgtcactgctttctttggcaatgtatattcttattttccttgtcctctatttcattttcttttctctttattagcttttttttttctttgcctattctggtctctttgatttttttgtgtagggaggcaatgccaagtgaggttcagcatgtgcacagtcctagctcaattggtggatgagttccccttttgagtagcatgtaacatagaaccactagtgcaaatcatttagattattggtggatttacaaggtcttttttagtggatttaaagtggcatctttaatgcatcttgcactataattcttagtggttaatcttagcagttttttcagtgcaaaccatttggattgttggtagatttacagtgtcattttagtggattttacagtgtaattttaatgcatcttacactgtaattcttagtgggcatgatagaaagtattgtcgattttagtggattttacagcaaatttcttggtggtaatattattgtaattatacttctttgtcaatgtaatttttaggtgacatcatcttggtcct

>TaeST2.16722.1

cttaactcagagactcaccacaagtacacagtgcaatatacggcaaaatgcatgaacttccattgagactcatccaagcctcgaagcagcatgacttaaactcttgcaccatcaagaaacgaaccaaaaacaaaaatactagaaccacatctctgctcctattgagttcaactatcgcagataacagacagacgtgacataggttccacataatcaagacactgtcctaaaagcgcattattttaaatttgatagtctgcgctctccttagaaagcttcacggtcaccaccatagccaccgccaccaccatagccgccgccgccgccaaagccaccacctcctccatagccaccgccgccgcgtgag

>TaeST2.16953.1

agtagattcaggactcacgcacaagactatttggaagaagaaattcaggcaccaaatcaaggtgagtggattcaggcccgcactcggagaataatcaacggcacgcacatcgatcaacacgggtataattaacaacaagatagacttgcaacaagagaatgactttagctttacagtccgatggaattcagcagaaattgagattattcttccacccaacccaacacgcatcagtcagaaaaatccagacaccaaccaaacgcctaccaccacagtagcagccacttgcctgccttgagcgccgccgatcgaggtcgaggctcccaccaccgcaccaccaccatgatgccacgtcagtagcacgggacgacgacgcagcgcctgcgccggggctgcggcgagcagacgacgaacagcagcatgatgacgaaggcggccatcaccagcggcagcaggttgaaccccgcctcctcctcctcctggctgcagcacatcttccccatggccgatcgcccgctcgcttttctcacagacagagcagtttcttggcttggaggaacaaggagactgggcgaggaacaagggaagaagtccttctttcgtggtgcagtgggatttctgctgtatctatctgtcttaaagaccactcccctctctcttcctccccctctccgcgtccggtgggtctctgaatgaaacttctacactatttccctatggcacttgtgaagttggctcgcttgcgtggtttgtggc

>TaeST2.17030.1

gtccgtcagtcagaggcagaggtcgagccgtggagtagatctcgccggctgggctcgtcccggggctggtgcgtcctgcctgccgccggcgacctcctcgtcggtgagtacgtgcagcagcccggagaggtgccaaaggctggatctcgcagaacaaggacatgaggtacttgggcatggaatgggaatcacaattgttgtggtggaaatgaagaatatcctgttatttggctgctaccatcgatgttcttttagattgaacattgccatgagcgtttctctacgcaatgaatatattagatcaagcatcatgtcatggacaccataagacttttctgcgttgatagtgatgtctacgggttggatcgtacttctgtttatcagctcactgaagtaactctcctagctcaaatgggttaatttcgtgtttacatgatacacaaatcttcagctatccatcttcatatgaattggtctcgcttaattttgtaatcttctggatatatgcttacatataataaatgatgtgaagatagatcatagaacaaaaccataatctttagtcttaagcaagttagggtaaactagagatagaacacaacagaagccacacacacgaatgacatggaaagcagcaaagaaatagtaaaagaagctcttgacttgtagattatgtcaaagtgataatctagagtcaagcttcttttactattagtttcgatctaccttgtcatctataggattagttattatatctaagtatgttttcagaagattagaagattaagcaaggtcacttgataggtaaattgatagccgaatgttttgtttatcatggaaaaccagagattagcctatttgagctcagtgatagagttactcaatgagatcataaatagaagcatgattcaaccaatagacatcaatttgatgaaggcatgaaaaatcttattgtgttatgatatgatgattgatccaatatgtttttgccaagtgaagaaaaacactaacatggcactgagtgaaagcgatctatacaaaggcaagatttgaatattatccacccgaaagagaatgtcagatatgaccaccatcagaagaagcaacaatgccacaagtgagctctcttgtatatccatgtgctattgtcgattcagcgtcgcatatgttgagcgtttatgttctacatgtgttagatttagaatattgtggtgagcacttccattcttgccaattgagatggtgaggctcaggaacacttcatttgccatggtgttttgattcgtcttctgcagcaacatgaaactctcattgtaattgtacccacttatgcctgaatactgctattacttcagttagattcaagaagcaccaagtttctgcagcgctcctgcattttgctaatgatgaacttccttctccatttctttcttttgtaaggaagagttatgtacttatgatgtatgactggctggttaattttctgtcaattcaacaaccagtttgtatggtggccagtgcaatttgcccgtatgaaccattagttattatatatgtactga

>TaeST2.17107.2

ctcaaacccaaacccaaaggagagagatacacaaacaagagcacggacaagtttaggcatttcatgtatggaggggaaaactactctatgctttctcatggtgcttctgctgctaggaaactgcgcacatgctgagaacagctgcaaagagcaccgtactttggagataggctgcataagaaaatattgtaggaaaaactgcaacagagactacgaaggacatcgtgtcaggaatgcttattgtactggctttatccccttcgtcttttgtgtctgcgacgtctgtgacggctagaagtactacacatgtaaggagacaatgcaacatcacatgatggggttcatttacacaaatgaatcatcaagtttggcaataataaattttgtctacgtaaatgtgtgaaca

>TaeST2.17107.3

agtgaatacacaaacaagagcacagacgtatggaggggaaaactactctatgtttcctcacggtgcttctgctgctaggaaattgtgctgatgctgagacctgcaaagagcaccgtgctaacaagggcgcagcgtgcataaatgaaggttgttgggcagactgcgccagagactaccatggacattatgtcaagaatgcttattgtaccggcgtttaccccttcacaaaagtctattgtgtctgcgacgtctgtgaagagtaggagacaatgcagcatcgcattacggggtccatttacacagaggattcttgaagtttggcaataataaattttgtctgtgtaaacgtgtgaacatgattactgcctagctagttaatttgtaacgtggttgtttttgcatgaacaccatcatgttttattcacagattccgtgtgccttcatccacaggcaagtaacctggaaacaagtttggcttttaacattgagaagctactaacccttc

>TaeST2.17113.1

tggcatgaacatcttaatcaccagatccatctatataaaacagcactggtgctttattattagtcgcccttggtttacacttggtacggtctcctatatagttctataccggcacacaataaatcacagaagcagagaggagggaggagcaaaaaatagagaaaattacagcggtgaataaataaataaaatcgaaattactgcagtgcagatgcagccagccagccagctgattggatccactgagcgcgtcagctcggcagatctacctcgcgttctcaccgtcgtcctcctcaccacagcagcagcccggagagcccgagcagcagtagcgtcgccgcggcggacgacgagacggccggcgccgagcccttgagctggggtggcgggctgtagtagtagaaggggaagtagggtaggaacgggttgggcggcgggggcgtcgggtacgcaccgcctccacctcctcctccttgtcctccttgcgacccgccgccgccgcctcctcctccgtacccgccggtgggcggagggtagaagtagccaccgccaccgcctcctccaccggagggagctgggtaggaagtgggaggcgggtacaccggcgccgcgggggtcaccggagccggaggaggtggcgacgggtagttgtatcccgggttggggttgcaggggttcccgcaggaggcgcagagcgtgcaggtcgtgccgtccttgcgcacgatggtcaccccggtgtcctcggcgccggccgcccgcgccgcatcggggagcagcgagcagaggaggagcggcgcgaggagcagtagcaggaggaccggtgggtggggagccatgactatgtgggaggggttggtgatggggatgggagtgggagtgggagcttgagtgagtgagtagtga

>TaeST2.17173.1

gctgcaggtcaaccgaagacacatcctatcattcttggatcagattaaaattgcgatcgtgatcgattggctgcatcatgggagactgcactaagcgcgttaacgcactgttgctacttcttctagtatgcttcgctattcatgggcagtttgaagtcatcggtgtcaatggcgagcgtattcctccgtggtgcacaaagagtgcaactataccaatctgcaacccggataaatatagatgctattgttgtgtaggcaattggcaatgctacaaaacaatggacgagtgtagagccaagtgtgcatatttacctccctcctcttcctctgtacttccatgaaaccggattcttatcatgagctggaagaaaaatgaataagatccagacaataaacaatttgctatgaacttatttggcaattattctttgcagtttttggtttgggaattccatgttgtactccatgcaacataaatattgttctgttagaaaatatgctagatcgaaattaa

>TaeST2.17227.1

gagtaagaagtttgtgtcttctcttatggaagggaaagctattcttctttgccttatggtgcttgtgcacctaggaaattccattcatattgataaatgcaaggatctagttcacccgcaaggaaaaacatgcaaggatctaattgcttctatcgaccgtgagtgcgatttgtcaaattgccaactgacgtgcaaagatttgtttggagcgaaactcacagcctcatggtgtatcaaccctgtcccatactattggcattgtaggtgtcgtgtctgttggtagagttgcactgaacaatcatgtcaaaaaatggacaaatgatgtgatccaataataaaatctttgtgtgacatgcatctaagaatacataatgattgccaaatttaggtgttc

>TaeST2.17329.1

agaaacgggaataagaagtttgtatctgaccttatggagtcgaaagccattcttctttgcctcatgttgattctgcagctaggaaactccattcatgttgagcagtgcaggcaggaagcttcttttacgagccccgattgcacagaagcaacttgccaaagtgcttgccagaagtcgtggggagatgacgtcctgcatgtggtatgtaggatcacaggcataaccttggaaacttgttactgcatcgtatgtgtttagagttgcactaaaccatcgtgtaaggcaacggataatgatgtgatccggtaataaagtcctttcgcaacatgcttgcgataaactatagtgacctccaaattcgggtgttttatttgttttggtcatgtgtgatcttaaatttggttcttttgtgtttgcatggcctcgcgattcacaagtttagaaacaaatctacacgac

>TaeST2.17346.1

ataggaaaatttagtctgcgcttatggaggggaaagttattcttgtttgccttatggtgcttgtgcagctaggaaactccattgatgatcattgcgagatgcgaactattctttcaagtcctaagtgcacaggctcaacttgccagacggcctgccaaaaaatttggggacctgacgtcaagagagctgagtgtagggttgtcgaccatcacaagtattgtgactgcatcatctgctactagagttgcactgaatggaaaatgttaggatccaataataaaatcttcgtgtgcaatatattgatcaccaattttggatgttctattttattttgtcgtgtgcaatattttttcctcaatcatggcaatacactgaaggccgggaaaaaaaagttat

>TaeST2.17418.1

aggcaggcaaatcagctccgtacggaattcttctttgttgttgtcgcacgagccatgaagagagcttccgtttcttctgccatggcaatcctagttatcttattcatatgctactccctgccatgctcagccgccgaccaagctcatacggaaggaaaccatgttggagtgagtcctccgtcgccgtgggtagcgccgggcccacataatcaaggtcgtggacgctccagcaccccaccgccagtgccgcaccgcaagaacttacagttacagcaggctgttcttcaagtgccactgatgccatgaattatcttgcacaagtcttgcaaacttcatcactttaatttttttagtgcattgtggtgctagtgccactaaaatgttcctagagttcagaaataagtatggtagtgatggttgtacccttttctacaattatgaactttaattaaaaattatatttacatgataatcctatgga

>TaeST2.17573.1

ataaggaaagccacagtagaaaagaaataggacaccatttctgatggactttatcaagaacaatacaagggccctatgcttggtagttcttctgatgtctgccactttttcatgtcatgcaactgggagaaacaaaggtacgactgcatggagagctttgtgtggagattttaagatatgtgagaacccaacggtgaacaatgttaatatttgtagacttaactgtgaatggtctggttacgtcttcgatgacagctactgcgaccgtggtaagtgttgttgcgcattgataccgccaaagaaatgatgggtactacaatgtgccaaaagtttgggcttgtaagatatggtgaacttcaataaagtatgcaagttgatcttgatttgaatacgcaaa

>TaeST2.17592.1

aatagattaatctttccttgcttaaaaaacaacaacaattggttcctctgttctgaagaaatgatcacagtcaaatttctgataaatactttattgaatcttttgcatgaactagacagatccatggtgcacatgtatcttagcctatcttacacgcaccgatttattagcacgacccatccattgtttctacgtcgtttaattagagcagcagcaagaaccaccattgtctggtttacagtggctttttttgaattcgaaaaatgagcagtggttcctgcacgcctcttcggattcatggcctttctcacagcttgcaaaacgcacacacaagtccaaggggattggttttgttgaac

>TaeST2.17633.1

cgccgacctcgccgtcctcctcacccaccgcctccgacctcgtccctccctcccctccccgcatctcccggccacctttacgcccgaggagatcgagcgagcgcccgagcgcgcccgtcctcgcagcctcctcccatgattccccgcacccgagccccacgagaggagctcctcccgtggttttacaagccgccgccggcgaccaccagccacagcttgccggagtgcgacggcgccgccgttgcgagcactttaccctgtctcatgtggtcgcccgcatcacttccccttgctagggtttctgctcgggccgtggcctagatgaacggcctcggcagttgtggtggcgcgtgtgagatccccctccccgacaaggctgcaactggatcccctcctctcgaccgctggatccgctggatccgctcctctcctacatggccgccatcggatcccctcctctccgacagcaacgcctatggttccagcgcaaggtcgacaaggtcaccctcttcgtcaaggtcgacgaggccactctcttcgacctcatcctggtagtatttgccaaaggttgttgttgccgtggaggtcaacggtcgtcacaagcctacaagagaatatattgacagagagtttaatggtgtaaaaccacccaataatttcggcgaagttgatcactggaccgcgtgggcgtacagaccacggaccatctcgttgcttctgatgggaacctgctttttaatgtgagctgctgctggatcccttttacctgtggagttatttttagttttgtacagagattcttacagcaatgatgttagtatgcaccgctaatggttgacctactatggcattttaccattgttaagatcacaagcggggaaaaaagagttaaagaattgtgatgtatgtttaacatgcaactgtttttttaattctttatcagttgggcaagtggtgctcttaatccagaaggaaggttctctgctgaccgtgtctcatctgtaaaaaatttactccctccgtcacataatataaaagcattttttacactacactagtgtaagaaacgctcttatatttgggaccgagggagtattttatttatgtattggcggaaaagt

>TaeST2.17657.1

accctagccgccgtcgtcttcctcttcctcttcgtcgaagccatcttctggtctggtgctgttggtagctatggcgctgcgcaccgttgccgcgaagctgaaggcccccgtcgcctctctcaagcaggcttgggcggtctgccgtcctcaggccaagggtcccaagctctctaaaggtcttgacttggatctttcggatccacacatccgccgggttttcgacgagaacacaaagctattgaacaggaagcggacgagggagactgctgccttaattgttttcatgactggcacgacaggcggtgctctccttgccatgcgttaatgatctaacgagaatccctacctatcgtttatctgcctatcaagctaaaagctagctatctccctacctatcgtttagctacctatgaaggtaatagctatctgtctgctgtcattttgtatttaagggcactctatatctgagtattgtctatctgtggtgtctgggcagatgtgaaatctctatctaattatgtataagacttaatgaggtctgttatttgtaatggtcttcatgatatttgtaatgtaagatctctatcttgcatgatatttgtaatggtcttggtgatctggttctgaatgggtaactcctatcacacataaaaca

>TaeST2.17764.1

aggtcgggttgtcgcaggttccatggagcccccctcccctcgtcaagctcggcaccaagaatgcaagtacttgatattccattctgttcttgtaggtgaatggaacttgcgttgttggtagaactccgttgaggacttctacccatgttgaagagcatgttcggtggtatccaagagtagctagcttttgtactggcagagcggtctatttgtagatcccaatatttatgattgtgtggatctgggtgaagtttgaagttcctgggtatgcaagattcgatggttaaactggttgatgtgatgtgaatgagtgtatggaaactggttgttgtgatgtgaatgagtgtatatattttcccgtgttctgttctgtatagcatattataataaacttattttagctgttatttgaaggttttcatatgctttc

>TaeST2.17832.1

catagaaccattttctctgtaaaagaatagtagcttgagagacttcaagtagcatcagacaagcagagagatggcaacaacattttcttccatcttgtgcaagatccttctcgttattgtagccacgcttgccctcctttgctctgcctatgcaagagacgcatcagaatatgggcagtgtttttcccggcctgaatgcagcaactactgcaagcagcaagggtacctcaggggaggggaggtcatgccgccgaacttcatggactgttgctgtttcatatgagcattaccgtgtaatgttgttccattttatgtctaaataaaattaaatgctataattaaaatgaaatctttggattgcatgcaaatctgccgag

>TaeST2.17892.1

ttattcttcaggcaaatcagtagactagaattcagatggacagaaaacattgtagacaaaaacagtcaacggtacataatagaaacagagattaccaatagttcaagttgatttttttttcttactgaaaaggtaaagcacctgcctgttctgcaataaactaacagaaaaaaaggattggtcccggaagtcatgcatcattagttacttaattactccagtcgcaatcagataaggtgtgccatttccttcagcaaatttaagccagagaaggatggtgcaaaagtgattacaacatactaggtccatcctctgtgtaccagtcccttacagagcctgttgctgagaaggaaaacagagcttgcgcagaaaccgaattttgctctcaagtttcttctccccatgcggtgcaagaccgagccaatggactccacctactgctgtcgctctcttgcagatgaagatgatggataagtctgtgta

>TaeST2.17893.1

tgactaaatatatactaatcagccagcttaactggcaggattttccccaatttttttgacagagcagacacgaacactaaaacagtagagtggcatttatctatgatcaacagatccgccaaagagcataacaaatttctatatacatttaacataatcaatcattcgaacttgattctaacgatttaagttcaatcgaaggtcgcatgacaattcacagaggggtggggggatcgagggagcagagacgggtgctgaacctctcggccggagtcgcttcgtcggccggcctcgtagttcgtgcgccatgaactggatctggaggcgcgcttggactggcggctgcgcaccttgttgtggaagaggcggtccaccggcgagatcgagcccctccgacggcggaatcgggtgcggcctgctccgttcaggacttgccacgggttggtgggaagccctatccagctactgatgggattcgggccggccgcacggcgcagcccgccggcggcagacgcgctccctcgctccggcgcgcgtgtgttc

>TaeST2.18161.1

agactaaatattactccctccgtccgaaattacttgtcatcaaaatggatgaaaatagatgtatctagaactgaaatacatctagatacatccatttcaatgacaagtatttccagacgaagagaatactattaattatatgagatcataaaggtaagaaatcactaacatagatcacatctcataattttattacaagatggtcatgacattatttctcatcataaatcatatgtaatggttgctcatggctctgtcaaggtatgagatcgcttgcatccaggagcgcaacctttttggcattgttctatcgttggaaagcaatcacctcctaggtcgcagcagtaaactgcatttacccgtgagcatgtgcacatttggaccttgttgttgttcatttgttcaactgatcgacctgc

>TaeST2.18294.1

ctgggagtaaaccctcgactggctcgctctccctgcgaccggtggcgacggcctccgcctccagcctccacctccgttcggtgcatctccgtccgacgaccggatagcgccgccccccgcgccctccctcaagagaaccgcctccacagtccgtccctccggcagcagcgacggcgacggaccgggcgtcatccatctcccctctgcctcaaagcttctatcacctccggcgacgacgatggccaagaggtcaccctcctttcaccaaggtgctgtatcatgagctagcagagtatgtgcatctgtgctcctgccgctccacttccaggtgatggcgtcgggttggtcagagagggtgaagctctcaagttcccgccaaagtatcaggtttcctaaagatagcaaataaaaatgttcaaagaagttgatgctggaagtttcagtaaagtatctatttcaacagcaaaactttttgctatggacgtgtcacatatgggcatttttttaatggagctaaatgcttattcttgctggatttttaaccgtagttagtatttaaaaaaacatgattagcacaataaattttatcctcagtagtgttgtgtaatttgtgcgactcgaccgcataacttattgttagcatgtaattgggttgaagtgttaatttgctgattatgtgtgtattattattttctccaaaaaaatcctgaattattctctatcttcctgagaacttgtgtcatttctgaacttatcaaggaaatatacagaaattttaattact

>TaeST2.18302.1

tgatacaggctgccaggaagaaacaacctagaggatggatgctcgaacatgcaaggagaagcttcatcccatgctggaaatcagtgctatcatcaaccaagttggaagcaaaggcatctccctttagtcccttgatagaactaaaaagcatgagagagactcaggaaatttggaataaatcagcagccgaaagatcctcttcagttccaactaaaaatcttggtttgctgggaagagttccgcaattaacaagtagttatcaatccagaaggctgttctgtactgctgcattgacggccttcaaacttttaaagcatgcatgagttgagagttcctcagaagagaagttacttctatctcaaatcattttcaagtctgcttattattgcttgcatgctatctagccagtgtggctttcgatctacgtagctacagaagtgcctgtttctgaccttatgcacagatgtggtgcaaaacatattttatttcagtggcttgctcctaacgcgctcttatatattttattttagtggcatgctcgcttaagcgcgtatgtatcccttacatttgcctggatgtatctcacatttgcctggatttaaagctacctatagtagcatttgaaccgctctgaccactgagcttgcgtacatggtcgatcgtgcattccttgaggatatattaaacagttcgcgtgta

>TaeST2.18357.1

cagtcgatcccctttagtcctcctcacccacatgcgccaccggttgtgattagcagccagcgaatttctcatcctcaggtactaaacgcaagccctaatcatggcgacctagaatggggcacgcagcagaggcaaacttgtgtggctgggtctcagctcgatggaggagggccatcaatgggagggcagcgtttagcccaaaatttggttgccgaaggggaggttgatgtacacgtgctggatctgcggcacaatatagaggttttccttccagctattgtcaggtctcaagggtcaggttttcttcgaagatatcatatctcaaaagaactcggatggattacctgatttagtaggttttcttgattcattgtagtgagaagcaactcaaaaggttggggcatgacttcatccggcatcggcaccaagttgcacgtgaaactttggtttttcctggcttcgatctttatcaggcatagtattggctgactttgatttttgtgattgtgttttggtcctgtagtttgggctggcttctactttcttgggtgtgtgcctggtttggctggctagatgtgtttggttttccatggttgtggaattctgtatgttgtatcctactccataaatcttatttgcatgcacgcacctgtcagcagtttggaattgtgtccacgtgaagtgagatttcgtttcctaatg

>TaeST2.18499.1

ttttctcatcgtacagcagcaatatttcatgaaaaaatctgcatacaatacttgtagatctatactacaaaatcttcacatcagtgcatacatctcaacattaataagatatctttttgcaatgaatatgctttcacaaacggagacagtgccagcgtcgacgcacacgataacttacgtgatgcatgaacactatagaagtacagatccttgcagattcacggatctaactagccacacacaagaatacctcacagatcatgctcatggacacacgaacaactttaagaaaatatcacacaagcacacacacgtgcactgagaaaatcattcagaggcttggacaaccagtagaagtatgtgataggccatgtagactgtagaacgccattcgatgaagcatagcagccaatggtggaggacgctgctggggtgctgctgctagctaggagcctaggacgatggaggaaggaaggaaatcttcagccgccgattccgtcaaggtgagcaccgccgccggcgtcggcgtgagctccatacactaggctgaggagaacacagacctggtggagagcgtggaggggtggagtggtagggaatctcgccatggacagaaaaaagcggcggcttgagtgggatgtgtgaggcaagatgcggatt

>TaeST2.18575.1

acgccaccacaggcgcacagccttgcatcctcgtcatcttcgcctggaccccttgctttctctctctctcgggatcgctccacgccacctccgtctccgccaaccagggcgcgggatccctcgggtagaattgcgaagctgcagatcgagaagtgagccatggcgaagatcaagccgagagcactgctggcacagagcaagcagaagaagagccctactaaaatcggcccaacaatgtttgccttcatcgtcgttggtgccctagtggtgtcctcactctacgcatacaggtactggatgagcaaaggaccagctggagcagaaagtgttgctgggaactaagcccgagcaagtgtggaatgattgatttttgttgttgtatgtatcatcttgttggctaatttctgttgttgtactccagtaaatggtacgtagacagatttatttattttgccatgtttagttcctcttacccacctggtacattgtattaatacttgttttgttacactcttttctttgacttgatatttttctcgcctgggatgatattgctggaaggattggtatcgatggcagctgttgttgctt

>TaeST2.18742.1

tctgatagcacaaaatacttgctaccatgttcgcacagacccatttgaaataatatacatgtacataatacattctccagtgtacatttacataatgtctcatagtttggaagaaaagatatatgtcaatggcggtacataaaactaaaaatgagtgtattggtcgctagggagtaaaaggacctatcatgagtaattgtatccagtatacagagaatatttctctctgtcaaataactccaatggggcagcccggtgcatgtagctcccgcttgcgcagggtcggggaagggtccgaccactttgggtctatagtacgcagcctttccctacatttctgtaagaggctgtttccaggacttgaacccgtgacctcatggtcacaaggcagcagctttaccactgcgccaaggctccccttcgtcaaataactccaatgaatcatagtaaattcaatacattgtttggaagaaaatattgatagaagtccattgggcgctgaggaataaaatcgttggggactgaaaaatatattgattttagcaaaaagcaattcctccaagtgagaaacattcagtccaaaaccacatcaagaccagcatacctacaaaaagagaaaagagtctgatcattacagttgctagcaaccgaaacctggatcctgcccaggtcatcaaattaaacaatatgaatagttcaaatcgaaaggtcatttgtatccagcatagtgtattgcaattataacttgaaggggtatgcttgaacaaaactgtatgcagtccctctctcagtgttgacctccagtctgatggttatttatgcaaagactactgacgctctcaattcctgactggcacaaaccagagcattgctaactggccaaattagtgaaaagtgttatgtgctgataccttaaggaaatgggccttatttgccactatcctattaaattgttctctgtcacctctgatattttccaaatattattgttactttgaaagtaagttgtgatccttaaaaaattcagagccttgatgtacaaaaggcatcaggtttagccagagagcactcgaacaactgtatactgttatctacaacttccatagttacatttctttcatggctccgcatataggatacatcacgtcacatccaatgaaatggatgtgattactactccctccgtccaaaaatacttgtcggaggaatggatgtatctggacgcaatttagttgtagatacatccattttcatccattttttcgacaagtatttccggacggagggagtataatattattttggaaactctaatctgtaacattgtggcaatgattttcgacttcagaaccatgttatctgcacgttagcatgtaatctactaatactaggacaacgttctacttcgactatacttgatgcataatggtatttgtcctatcggtcact

>TaeST2.19420.1

cccaaattagaacagaagcaacgcgaacagagtagggcttcgggcctccggcggcggacctatgacgcggggcgaacgcggtcgccgcggtgctgacatactgggcttcgatgtccggcgggcaactcgccccatccccgccccgccattctccacctcacccccttccccacccgatctagcgcctacctgcgccatccgccgacgctggagaatagaactccggtgttcagggactgatggagtcaatgtcaagtatggctccgagtgccggatcgactacagcgatgggacttgaagatgatgcttacttttatggcctgtgtgccaagtgtgtttccttgcttcagaacaacatggaacaattatctaccttcgtgcttatattatgcaaaactagaagtgctctgtttgaatgtgacactgtgtaactatgtgttactgggcatgtccactaattcctttatgaattggaacctggacctatttatttgctatcagtgaggttccatcataatgcttgttatgctaaaattatttgatacaattagtttatcatgatgatgtccactttagttttcaaca

>TaeST2.19485.1

taataatgtgctcttcatctcacttatttatttatgtacaccaagacaaggttgttagaatcgcaatgtcgaatcagatcggagctctgatggtaggatcataaatcatagaatattaactatcaggatcgtagaaatatagattctatgaactaaaatcgtagaatcagaggggttagattgtattgtaaagtcatagaatcgcaattctgacaaccttgtgaccaaaatcactatatcaaacagacaaacagttcataagcatcgtgagtaccgacagggagaaacaataaaaaaaacatgattcatataagtttctactactcagagccaacttaaaaaatctcaagagtctgatagccgagtcggatctcctccttctccccaaactccaacagagagatggcgcactctggcaaggtcttctcctccccagctgtcacggatgccgagcccttgggtggcgcgatagccaccaccacgacgccgtcatacgccagcttcggaagtgccatgagccccccaccaccctcttgttctccgcggccacgtcaaatctgaaggaggcagcggagctggtgccgcagcaccaagccgagagcacgcatagctagtcaagccggcaggtggaggaggagagccccttgtactgcagcggatctcccctttctatccgtctctctctctgtgtgtgcgcgcgcgcgcgtgcgtgcgaggaggataaggaagaagtgagggatcagtgcttctctcgatttttccgtggccttcagcggaaacgatcagccctttct

>TaeST2.19562.1

cctctccacatgaccacgcaaaccctaatccgcctccacccacagccgccgcctcctctccctctctactcccgattcagatccatgcccagcctccctcgacgcgcgactgccacccccttgatctcccgcgccgccgccccaaccatatcccgctccacctccgtctcccaccctctatcgctcctccaccgcccccacactctcccctatgcagcctgctcagatccgcgatgggggcacacgcggccgtagcggcggtcccgcatccgcgtcaaggtcagccccctgttcgtcgtcatgggcgcggtggtcatgaggaaggtggttgaagtcggcgagatggacgttgtcgcctacccgctgcccaggaccaaggacggctgccccgacgctccggtcttcacaatctcggtctaggagaggaggggttgaccactgttttattcagcactgcttggccttattcgcatcgccacaagtgacctcgccggaccacttgccatgaaggtaattgaggtagaacagaagatattacacttgagtttagtttagaagggtaactccacttgggatggaacagatgatactacactcaagacaagcaacacagattatatgtatgatgtatataaatctccgttgtgattgtgatatgtaattgtaatctgttatggtgatctgaattttgtagttgtgaatctgtatttgtgcactatatgtttttctttgctaaatggagattctttacttttttgtgtggatca

>TaeST2.19663.1

ttccatgccaaattaaaatgttttagctgaagcatctgaagacagtactattacataccaaaagtcatacacttatgatagttgcaggttattacaaccatgatgaagtgttcaaaagagaaaccactttaggattctagatactcagcgaaacacagttcctgggcttcatctccaagaaggaacgttatatgactgtagcttgttacacatctgttatctggctaccgaattcggagtactcattctcattgggcttctttgcggcaccttctccaatatgcctctctgcaggttgcctaggagatggtctcgagtccccgtcatgaatggttggaaatccaaaagatttgaagaactcgttggtcatccgctcagcttcatccagaaagctaccgaagcctttctcaatagcttctatgtcactgcgaagtcctggaaatgcaaagggctcttttg

>TaeST2.19678.1

acaatagaaccctatggtgttctcccggtcgaccggttactgcatcttgagcctcagctccagatacgcttgttcgcttgaataaaccagctgtttggtagatatatacatttccttcttgttgcctctttgtggggttgcgattttttactccatgtcggatttgtcgcagagtctaggaaatggcaaacacagctatccacttgaatctggaaatggcaaatctgtttggtttgctttcttgacttgataggatcgtgatttcgtggtcgagcctatgtcaaccactggttccactcagccgagcaggccagacataaggtcttcaccttcagcatccagtgagagtgtcagggctcgcaattcaggttctagttccagatcagctgccccaccaccagcagcaccacaagcaggggctgcaaaccctttacgtttcgatgcaaagactatacatttctccgtaaatgcctgggcactcaggaaggtgccaaggagatgctggaccaaggcgtggaggcactacgagctgaagagcacgtagctgcagatgctggaggccgacccggtgagccgaggattggaggtagcgctgattggtgtttttatggagagataggatgccactgtttggtgtttttatgcattttggtttgcttgtaactgtgtttgcactagctgggagtaagtgactttgcaagaagtgtaggtgttttaattgaaggcatgaaaagccatgacaatatgtatgctcttacaggatgtgctctaatgctgctggtttattataaatcttggattagtaa

>TaeST2.19777.1

gttccattccaatcctcgctcgattccttcttcccctccagcgcctcctcctctgttcttctatccctaaccctaaccctaaggcgacggcgccgttgcccgcggctctcccttcaccgacggcaccatctagtccgagtccgagctggaggagcacgtgtacgtaggggaggcagcaaggctagcacgccgtcggttcgtccttcgctggttcctcaccgagcgccacgaagaggaaaagataatggtgaagtagaagggaaagggtgatggtaaggataatgcgaaggcgaagacaaagagaagagcgtgaattacttaattaggcaagattcggagctgcctccaccggttccgaggaagaagaagaagcctaaggtgcagattaggaaggcaaaagactttcccaatgccccaactggagagttgatttgtaggttaaaaaacaccctccacctcctccgcgtctccccgaccgcaccggtgccggtgtgcgtgccgcgaaagccgctgcctccttctccgcaacctgctccaagctgcctcatgcatctactattgtatgattgctttacttgatgcttgatgtaaccatatttccatataaccatataggtgttgttttcttatttgtgtgctcatgtgtgttaggt

>TaeST2.19800.1

tacatcaagaaataaaggttgcatttttctacatacaaagtatatttgcattcctctatcctacactaatgcagttatcaaacaagcagccagttcctacttcttttctgctcgctgctatttcatggatccctggccgcagctcgaacatcacagtgcggctatacctctcatacgtactagacaaaccatcaggcaaagctaaatcaactgcataaatatgcatttaagctaaaccgtctgaaattctaacggtcagatataatgggcaggttaatgtgaacatgctaattgctgcccagggtaagacaagtcaatcaaaaggcaaaacggaatagtacaataatactgcagagaattttatttagagacgtggttaaaacaacttgcaagaagcggacttattcttgatattacacgagaacacgctaaaccatgtccgattgatagctgcatcaacaccatagctgcagatccttgcaaagctggggcgcgcctgaaattattcagattaacaaatcaattctctacaaacaaatggcgaacaaggttctaaacatcatgtgtcttaataatctcgagtaattcatggtaaacatcatgagttgggcctcactcacacagggatggacatgacagagagaaagagagagaggggggtaggggtagcgagggggcttacggccttgttgttgtgctcggagacggcgaagcgggcgagcgcgatgacgtcgaggtcgttctcctggcccatcggcgagtccttgatgcctcccaccaacggccccgcgtcttccgccatggcagccttgtcccgtgcgctctgcgtccgggtctgagtaaacggcacgacgatggcgagtagcaagagcagggcggccaccgatcctacgac

>TaeST2.19897.1

cgccgccaaaaaggcggcggaagaggtggtggatgtggacgatgagggtgaggacgacgagggagatgaggaggaggaggcggttgacggcgacgacgacgacgacgacgacgaagtcgacgacgatggggaagaggacgaggacgagggggtggagggtgaggataaggggtcggcggcgcagcaggtggtcgacatctccgacgaggacgacgacggcggtgatgaagaggagggcggtgacgatgatgacgacgacgacgacgacgacgatgacgaggaggatgacgacgaagtcgacgaggaggaagagcccgaggaggagctaggaactgaatatctggtgcagccgcttggccgtgctgaagacgaagagcattccagtgacttcgagccagaagaaaatggtgatggtgctgaggacgaggagattgaggatgacgatgctgaggatggtgaggatcctgtcaaggcgcaatcctcatcaaagaggaagaggtctggtggggaggatgaagatgatgacgacggtgatgatgatggtgatgatgatgagaagcctccatcaaagcgatagtcaatttgttcgtgctgatctgtgatcttctctgcttgggccaagaaggaaaataatcctcatggagaggattgttggtaaatgctgtagctttaggacatctgtggtaggtgtagcagctcaaacttgagtctacccatgttagttgttagtgcatactgctgatgttgattaacagcctccgggaacttctgtatgagcattagatatggggcttgttatatgcttgtgtagcttctgattaaaacctttggaatgtggatgctctgttctgaatcttgttttcatgatatttctcttgaactagcttgctgtta

>TaeST2.20007.1

aagacttccccgcagggagcgcggcgtttcgcggggaagaccttttctccgccgccgttgcttcctccctcgctcggatcgcggcgccggcacgcgcgaggccggcggtggatcaccaccggcgcgcttccagcgaggtcgaccccgagtgctccagcagcccatatgatcgtccacgtcgtcgccggcttccccgatggatcgccgagagaaaaaagctcccaaagtggtcggacccttccctggaccctgcgcaagcgggagctacatgcaccaggttgccctttttaaacaggcactgcatttaaatcccaatttgtactgtcattacggtgaggaacatgtaacctctaatgcaaaagacaggtattacagagtgtgagagtatatgtatcagccgatattatctctactattgctatacactgcattgtatcactcaagagtagtatcacataagcttgcttagtgtttgcccaaaagctgctccatgtttctgactctcccaaccagacctgaacaaaagtaacaccttaaactcagcacacagcaagtggtgatgatgttgccttcactggagacagtcaatggcgaaggaacccctggccggtgttgccgcaggccgtgcagatccggtggaggggtgacctctgatgggagagtaggtagctgtctgagggcagtggcggtgtctccccgtcggttaccacagcaggaagaaactagacagagaacaatcactgaatttgtaagggaatatgggcaaggttagaggaagaaatatgacaaacagggatttcat

>TaeST2.20781.1

aaacacagatgaacataagaaggagggacgaatagaatagaagtaacatctttgtttcatggcactcatcaggagcactacaacacgatcgacgatcgtgtgcctgatggttgttgctttggtggtgtttgctactataagctttaccttgccatgttgccatgctcacattgaatctactaaaaaggcggtgtgcgagacgctggatgcatgtatctctgacgctggatgcaaagctatctgcgagggtagtggcatggttctctactataacgcgtgtgcgggtcgtgattttccaatctgttgctgcatacctatgtaggggggcgctcttccgccgtgttgctgcacagaaatataaaatttattggcggttctcggcccgagctatgcaattctgaataattatatgcttgtaatgcagctacttatctcccatatttgtcttatacacacaagtataatgattatcttctactgcctgtccttttcttttctggaaaaa

>TaeST2.20783.1

ccagtgtgggcatggcaggatggcatggtagaacatataatagcaagcaccacgaaagcaacaaccattgagcacacaatcgtcgatcgtgttgtagtgctcttggtgagtgccatgaaaacaaagagattacttcttttctattccaacgcccttcttatgttcttgtgtgttctggtcctgcaaataaactctttcattcgctgctatttatattagagaggcccaattacatcttatgcaatgattttgcatccaagtatttctcctaaattattgttggcatatcttgctggtcctaccacatcttaggcaaatattatgcaatgatatttcatttatttct

>TaeST2.20779.1

ttcggaggagtcgccgccgcccccgcccgactcctcctcaagacgccgcccccgccacccaactcctcaagacgctgccgcccgccgcctctacgccgacgtatctcctccggcatacccctggttatggcagagaaccgtgagatctttctcaattctgccactgaagatgaagagagtgctgccatgtttcttcagagcgaaatggcagagttgccttgagggacctgacctcgcggaagacggatgaccactttgctgccacaagtcgcctccacaccgtgtagcccgcaaacatgtctcggagcatttgccaatcaaattttatgttgcaatgaaccgttgactttcgttgatcgaacatcttgtatttttcatttggacagtggcatgtatgcatgtaacattttcatttgaaccgttgatagtccttgcaacagattatatgagcctttgtgttattttgcataatctagttgcaccattgttgcaagtacatttcttctatgagcttttgaagcttttgaaattgtaaaaaattgcatctttaattatagtatactaattttgtaaa

>TaeST2.20780.1

acacacatgaatagaacaaggaggaatagaatagaagaagtaatctctttgttctgatggcactcaccaagagcactacaacacgatcgacaactgtgtggtctgtggttgttgcattcctagtgcttgctatgatatgttctactttgcgatcctgccatgcacgcattgaacctaataaaccgccggtgtgcgtgcggatggatccatgtttcgagcgcactacatgctgggaaatgtgcgtggatatgggtttccgtgtccctcatccccgttgtaaggggagtgtagatcggctttgttgctgcctagacaggacccgtacaactcattgatggttctcttcctgagctatgcaatgctgaataactgtatgcttgtgatgagcgtgtgctgatcttaataaaaaaatattttttca

>TaeST2.20906.1

gtgccagaaggaaaggaggacaaatgggagggaccggttcaacctttttgtttgtttttggttgaccagttttcaacccgacccgttttcaacagacaactgtcaacagttcattggatggaaaaattgactgacatctaattacaaatcagtcaaattacaaatagacactccctttttgtttggggacccaggcacaaattattcttctgtgcaaaataagaaagaaaatccaagatttttctttctgcagggaattaatccaagactattttagaccttccctagtatcatgtcagtggaataatcattgggccccatttattagtagatcaatataatgcccatcacacccaacttcatttcattgtaatgaccaccgattattggctaacataggagctgatcaacagttcaacgcacaagagttattatgtttacacacagaacgtgtgtacagaaaaacgagagtaaacgactaccagcccaccaaaagagccatggttctcttcatcaggcaacggaccgcaaagcatactgaattggcctccccccatccatcatcagttggtcaagatttctgtcgccactcccgacagcaactcggccccaaagcgacgtttgtttcccagattaatttctctt

>TaeST2.21017.1

ccaaaggagagagatacacaaacaagagcacggacaagtttaggcatttcacctatggaggggaaaactactctatgctttctcatggtgcttctgctgctaggaaactacgcacatgctgagaacagctgcaaagagcaccgtacattggagacaggctgcctaagaaaatattgtagggcagcctgcaacagagactacgaaggacatcgtgtcaggaatgcttattgtaccggctttttccccttcgtcttttgtgtctgcgacgtctgtgacggctagaagtattacacatgtaaggagacaatgcaacattgcatgacggggttcatttacacaaatgaatcatcaagtttggcaataataaattttgtctatgtaaacgtgtgaacatgatactacctagttaatttgtaacgtggttgtttttgcataatcaccatcatgttttattca

>TaeST2.21165.1

gaagtaagaaatttgtgtcttctcatatggaagggaaagctattcttctttgccttatggtgcttgtgcacctaggaaattccattcatattgataaatgcaaggatctagttcacccacaaagaaaaacatgcaaggatcgacttgctactattggccgtcagtgcaatctgtcagaatgccaggtgacctgcaaaaatttgtttggaccgcaactcatagcctcatggtgtctcaacgccgtcccatactattggcattgttggtgccgtgtctgttggtagagttgcactgcaaaatcatgtcgaaaaatggacaaatgatgtgatccaataataaaatcttcttgtgacatgcatctaagaatacataaagattgccaaatttaggtgctcta

>TaeST2.21173.1

tcttatagatagaaccatattttacaaattaaatatagtgagacgggccaaatctatgtgatcttattttgagcaagatgggagtaaatattctctagccattagtctatatataaagatacaatgcttctagacgcccaaaccgagtcaggagtaagaaatctgtgtcttctcttatggaagggaaagctattcttctttgccttatggtgcttgtgcacctaggaaattccattcatattgataaatgcaaggatttagttcacccgcaaacaaaaacatgcaaggatcgacttgctagtatcgcccgtgagtgcattctgtcaaaatgccagacgacctgcaaaaatttgtttggaccgcaactcatagcctcatggtgtctcaacgccgtcccatactattggcattgttggtgccgtgtctgttcgtagagttgcactgaacaatcatgtcgaaaaatggacaaatgatgtgatccaataataaaatcttcttgtgacatgcatctaaaaatacataacgattgccaaatttaggtgttcta

>TaeST2.21180.1

aaatagaggcgcctcctcctctgctctatccctaaccctaacccaaggcgatggtgccgttgcccgcggctctcccttcaccgacggcgccatctcttccgagtccgagctggaggagcacatctacgtcggggaggcagcaaggccagcacgccgtcggtccgtccttacctggttcctcaccaagcgccacgaagaggaaaagataatggtgaaggagaaggggaatgagaagggaaagggtgctgccgagggatgagaatgaagaggatgatgttgagaacatcaagttacccactgtggtgaaggagatcaacgattgataactattcttcctacatgtttcacttgtcatacaataccaaatattatttatgtgaatgttgcttatcttacttgtagacattcatcatgtggatgttgatcttgctattttggagatgaatgtcaaaatgatctttctcatgtgaaggctacttttgcatttctgttatgttgaaacctttcaagtcatgaactatgaattgtcactgctagcttggagatgaacctctcatttgtgttatgttcaacttgtcaattgccatggacttggaattgttagagcaagtttgtaatcgtgtcattcaatgatgataagaccttatgtttgtgga

>TaeST2.21211.1

ctggctcggcgggacagggcacataacgattcactcccaaatcccggtcgaccaaatccctaaccctcgttccccattcagtgcagcggcagggcgaggcaatatcgctcacggtcggcggcgaggccgtatccttgcaccacggcggcaagcctccggagctcagatgggaggatctggcattccgcggggtgaagatggaggacgacggggagccatgggcaaagattacgtctgtcgtcactgaatccatctcccctctctggttctatagttcatcaaagattggtattttagatgatttgcacgattcaaacacaggaccatgagatgccagatgtggtgggtgcatgccgcggcacgaggtagatcacaccttgcttgcaagaaagaggtcctggagcagagtctaaaggagaaagaggtcctggagcagagtctttggtttagttgcaatcatgtttgcaatggctgggaggtcctaatgcatgaatgagcaatgttgaagctcttcttttggtgaactcagtgtgctgtagtagctactttgtatgtgtactgaatgaaaatggcaatacaatgaagcttctgaattttaca

>TaeST2.21257.1

taagaaaatttagtctgctcttatggaggggaaagttattcttctttgccttatggtgcttgtgcagctaggaaactccatagatgatcattgcgagatgcgaactattctttcaagtcctaagtgcacaggctcaacttgccagacggcctgccaaaaaatttggggaccagacgtcaagagagctgactgtagggttgtcgatcatcacaagtattgtgactgcatcatctgttactagagttgcactaaacggaaaatgttaggatccaataataaaatcttcgtgtgacatgcatgcgagatcgtatattgatcaccaatttcggatgttctattttattttgtcgtgtgcaacattttctcctcaataatggcaatacactgaatgccagaaaaaaaaaagttacatccag

>TaeST2.21280.1

ctacaagtctatatataaagataacactatgtatacttctcgacgtccaaacataaacgagaagaagatttgtgttaaatcctatggaggggaaaaccattcttctatgccttatggtgctttttctgctaggaaattccactcatgctggtaattgcgaggtgcatattgcttatggcagcgctgcatgcatagaattatcttgccatatagcatgccagaattcctggggacgtcacgctaagtcatcttactgtgtggccgtcagtgttacccaacagaattgtaactgccttgtatgtgactagagttgcaccctgctatcacgtcaaacaatgaaaaatgttgggatccagtaataaaatcttcctgtaacattcatgtgagaatctatattgatcacaaattttggatgtacttatgatttgatttcattttgccgtgtgtaatcttt

>TaeST2.21281.1

gacgcccaaacataaacaagaaaaacaagcttgcattgatcttatggagcggaaaactattcttctttgcctttttgtgcttttgctgctaggaaattctgctcatgctgaaatgtgtgaagtgcacgttccttattccggcattatttgcatagaattaggttgccagaatgcctgccgagattcctggggagatcacaccaagaaagcttattgtgtacctgtcaacgcttccctttggagttgtcattgcatagtatgcaatgactagaatgtgtctaccccatcatgttgaacaatgtacaatgatgtgtccagtaataaaatattcgtgcaagatgcatg

>TaeST2.21282.1

acagaaacgagaagtttgcctctgatggagggcaaaaatattcttctttgctttatggtgcttttgctgctaggaaattcatctcatgcggagtgtgaactgcacgttgcttttacaggccctgcatgcatcgcattgacttgccaatatgcatgccaaagttcctggtgagatcacaccaagcaagcttattgtgtgtctgttaatgttgccacatggaactgtagttgcaacgtatgtaagtggagttgagctagtccatcatgtcaaacaatggacaattatgtgcctgtgagaatgtctattgagcaccaattttgagtgtacctgtgatttgatttgattttgtcatatgtaatctcaggtttagtgattgtgcgtctgtatggctctctttcgtaagtctagacataaccatatgtaacatgaatgaaccaagtgttagaaataatgggcttggcccacatacattt

>TaeST2.21284.1

gaaatgttctctagctgcaaatctataagataacatcatgtatgcatctcgatgcccaaacagaaacgagaatcagaattttgagcttgccttatggaggggaaagctattcttctttgcctcacggtgctagtcctgctaggagattcaattcatgctcggtgctatgaaaagcctgcttactggatacctttgtattgcctagacgaaaatttctgccagaacaaatgcgtgaagaagttcggaaagctcgtgaaaggttcttattgtaaaatgactggatttttcatagtctcctgtttatgtacgctctgcgattagtgactatatcaagttggacttttccatca

>TaeST2.21285.1

agaaaactataagaacaactttgtgtaagattctgtacacccaaacagagaggagaagaactttgtgtctactctaatggaagggaaagctattcttctttgccttttggtgattttgcagctcgggaattccattcatgttgagcaatgctttagtggagttgtatttggaggtcatgtatgcacggagtcacaatgtcggtcctcctgccaacaaaaatggggaaaagaagtcgtgaactctttctgtagggtagacatcccaggctttttccattgttactgcattgtctgtgactcctagctttgcacaagggcatgatgtccaaccatggataatgataggtccagtaataaatcatcttgtgacgtgcatgcaagatcccataatggaaaattctaggtgttctatttgatcagggttgtctccagaaatttcaggctccaggcgaaaatcaaaatggggcccaaaatttcaaa

>TaeST2.21286.1

agatacaatcttgaatacttctggacgctcaaacagaaacgggaataagaagtttgtatctgaccttatggagtcgaaagccattcttctttgcctcatgttgattctgcagctaggaaactccattcatgttgagcagtgcaggcaggaagctgcttttacaagccccaattgcacaaaagaaagttgccaaagtgcttgccaaaagtcatggggagatgacatccttcatgtggtatgtaggatgacaggcataaccttggaaacttgttactgcatcgtatgtgtctagagttgcactaaaccatcatgtaaggcaatggataataatgtgatccggtaataaagtcctttcgcaaca

>TaeST2.21287.1

gcttgtgagtgatattatggagcggaaaccaattcttctttgccttatggtgcttctgctgctaggaaattccactcatgctgaaaattgcaaaacgcaagttgcttttgggaccactctgtgcgcattttatacttgcctgagtacatgcatatctatatatgggaaactcgtcaaggatgcggattgcaagcccgtcagaccttactatactaattgtacttgccttatctgtgtctagaattactctttgacatgatgtaaaacaatgggcaatgatgtgaaccagtaataaaatattcttgtcgattgcatgcatgtgagaatgtatgtgatcactatttttggatgttattgtaatttgattttattctgttgtgtgtagtattagaatgg

>TaeST2.21270.1

taccttataacccgagtgccaaatctgaggtattgtggtttgcaggtattcgaatgtatgcgtacagaccccaggtatttgagtattcaaatgtatatgtatacaccccggctacaaacgcccacacatacgtagtacgtactgctgttacacataggaatcaatcgagtggatcgattagctagctatatacatactactagtggtctagtacacgtgagagcagatcgagcggctcgattagctagatagaggctagctttgcttgctggtggatcgatgcggccgtgttggagcactcacccgccggcgaggaggagcactgcatcgagctgagcatggacgggtagcttctgctgccgcgagttgctgctgcttgctccgttgactgctgctctttgaggaggctgcggcacttcggcgtaggcggcggccgcgtggagtggagaaggagtggacggagaggagggagagccggtagccattcaagagaggatggggatata

>TaeST2.21466.1

tcatcaactccacttacaagagtaccacccattaatagcctctgttccccggtgatgatgaccaagtacatgaggaacaatgtgcaaggacttgtgctactttcttttcttcttgtggtgtgtttgacttgtctcggaggcacatatggcgaaatcataaatgacaaggggagcaacatgatgaggaaagtaagccatgtaacgagacccccttgttataaagatgggcatcagctcccaggaagggaattctgctgtaaaatagataacttttgctggccaaatctcggagaatgcttcatcaactgtccctgcaagattaattgcacaccagcagcagtagcaacacaatagtttcgttatcacccctattttggttggataaatcccaatcttatggttctcaaaataattatgtgctgatgagatcataaattttgtatgttttagaaatcaactatgttattttaatattcaataaaactagccaatgggttgtttgcacaattttgtggctagatttgtcacctaccatttacatagtaaatatttattttatgtcca

>TaeST2.21656.1

tcaacacgaatggcaaaaacgcatcggaaatcaaacgaatttaactcgcgaattcttcaatccattcattcaatcaatcgaacattcactcccgtctttcatttccctctcctcgattcgaaaataaaacaaaggtaacaataaaataagatagcatctctctctcccttcaatggtacgataaatccggatgagcgcagcagcaaccggccggcctagacgacggtgtagaggatgatctccgtggcgtcgatggcggcccgctgctgcctcttcatcatggccatggccacggcgccttctccgggcaccatccggccgccgtcgccggcgccgggggcggcggaggaggaggcgggggggtggtagtacatgcgggccgtctgcgggcagtgggagtggaaccgcgcggcgatgcgcacccccatgtccagcagctccacgtacttccccatggatctcgcgcgaatctgacgacctcctccctccctctctctgctcgacgaagattgacgaccggacggacggagaaagcagccgggaagtggagaggacggagggctcggcagcgctaagctctcttttcgtggaacggggaggcccaggagctgggggcggccatgggaggggtggaggagagcgggggagaggagct

>TaeST2.21824.1

tttttttcacttgaaaaccgatctaagtttgtctactatttgtaatacaattcacattgcattgtcatctactccctttgttcccaaatgtaagtctttttagaagttccaacaagggattacatacagagcaaaatgagtgaatcttcactctaaaatatgtctatataaatttgtatgttgtagtccatttgaaatctctaaaaagatttatatttaggaatggacggagtacatctcctacttactcactactaacattctttctaattcgcttcttgcgccattggcgcaaccgggtcatctagtaagca

>TaeST2.22301.1

tcccattttgaacggcgagatcttggaggcagagaggagagggccatggcgatttgagtggttagctctgccgctgatctccgaagagcgagcctgtggttggcgtccttcccctacccctgttgcttctcctgcaagcgcgtcgaggaagaggggaagtcgacacatggtgatttcgcggcggtatctcatctgctactgttaatttcgcgctgctgctcctattgctgtggtgttctccgagccctgtgtctcccttcttcccccaacgagccatgaatgtggtgtcttcacatttaagtatatggagatattcacaccaagaactcagatggcaaactttttctccagtgcagacattccaaatcttaggatcagatatgcgaatgacatgttttgcagccccatgaacagttgtgacaagtctttcgtaactggtttctatcgtgatggagaagcaatgccgggtgaggttaaacatgtgtgcggtcctagcacaaatggtggctgagttctttttttagtagcatgtaatctgaacaaagtagcatgcaatctgagcatatcagtatttttgatttagtagtggtctgtatgttttttcattttgattgtagtgtcatgtctttatgcctgtaactctgcagtacgtattttgagcagatttttgattctatatcaagggtcctttttttgttaa

>TaeST2.22467.1

aatagaaaagggatcttacatcaaataaggcattgcaacaagcatgatgttatcttagtaaaagaaattacatcattagaagagaaaagggaagaaaaccaaacatgagattgagattcatacatctacgattgatcattttgtggcctagggagcaaggatccatgatttttatatgccttgtccggctgctggtttttatatgccttgtccggctgctggagtaggtgacatggaaacgtcgtctctaccatctcgatggctcgagccggatgttcatctcagccctccccagcttccatcaatggatttttatagtcaaggttaaagactgagaggccctatgcatgttcaaatatgcaaatagcatatacaatacaaatcaagaaaaagagaaaagaagaccacaaacattacaaaaacacaattttgacaaattatctctattgtaaaattgagggagtaactcattattcaagagaactgaccattgacaattcagttacaattatcaaagagaactgtaaattgctaacttaattacatcattcttcacataagcttctcactgacaaaatatcttgaatggaaggtcacatttcaagccattacacttataaaacccaaccctgttgtcatttaaattgcaaattaatcatcaataatgcacaataacttatgacccacaaacgatttggagcccaagaaggagaaaatatcaatgcaaataaatacacacgtagtaagatgcattgcaatagccatattacttgaatttgtaagctggccagcaaaatcagatcaatcatgcatagtacacaaaagagacaaagctttaaccataattggcgcttaaccttcttgctcctgctaatgattttggccatgaaggcatcagagtaaggggaggaagaacgggacggcgcctgccggaagttgccgatgggtgttgacggcctggccaagccatctccctccatgataggtggcagttggcagaaaggtgtaaatatgacgagatgtgaaaataagctcgaggaaattaatatggaccagcaaactgacaaaagcagaatataacataattatattctggattaaactaatagcacaacaatttacagggttagaatgcagtgcacatatatcttagtggcgcaaaaactatttagccaacttcactaacaatttagaaattggcttcagtgtactaaaatagttattttggatataagcaatcaaccacgaatttaacatcagagatatctgaccattttaagtaacgaaaaaagagcccaatagcaagatagcacaacatggagctgcattgaccagccccttttaaagaaaggctttaggatcagaagttgtgtagttgatcaacttggaaatataaaagcatttccttcaaatcagtacaacacctccgacaaatgtgatgtgataagaaagcaatgaaaatatacactgctccacacttagttttgtatgtaaaaagaaagccatggcagatgatttttttggatcacagaaacccagcctgaatacatctgaatgatataatcaattttcgaaatgtttggcaggcacaaaaaaaacacacgagtaaaaataataatatatgattggtatctgtatttgatccacctgggtacatgcacatcctaaaagcaacaaacaagctgccatgcaacctgatgtgcagcttatcacatggtacacagaacttcacaaattatcaggacatcaatcaagtattaaaatgcatgagcaagcaggtaaacttaccccagaacttgtttcccaaagtacttacaacacgctgacaacaatataaagccggtgacctgtactgatctgcaggactacatcattgtaatataccagacttgtttcccaaagtacttacaacacgctgacaacaatatcatcagtgtataagtaaatgatgaagctggacaacatagaagcgccaatagagtcaatcggtgttgtcaacccattcttatagttgggtgttcaatccagacaaacaaccaactctttaggtacgctattctcacaaaaattcttctgggagagacaactgttggcatataccagactaatttacaaccatatcatcatcagtatttaagtaaaggatgaatctacacgacaaagaaggacctcatggtgccaatcagaagtaccatcaaataatcaaaagaaaggccatatttataatcaatcggtagaaatgcgatcatgcttatcgagcaatgggaacaagaaaagggacatttttcttcaataagtaattgctatggtgcaccggcaacacagatacagcgatacggaaatggggacaaaggatacagcaatttttagaaacagcaatacggcaaatatatataaataattaataaaatgccatataaagaagataacaaaatcaatgcatgagatgaaatgagatcgttgccttgatggctctttcaaaccctcaatgaacgactgaccattaatttcctcaatcctcagaactgcataattaatttaccattgcaagtatagcatatgctagctcattttatttgcaaaaagtaatttaagttgttgcagagaaatactcaaaacaatatctcaactaaaaatatatttagtccttagtttgacatgcaca

>TaeST2.22529.1

ttcgccgccgcatctatacctcaccgtctgccgccccctccagcctccccgagtcttctacaacatcgcctccacgccctcctcatctagcctccccgaagcctcgtgcatctccgctctcccctccgcggtgccctgctctgacctcgccaatgcattgcacatctctagccttccctctgcagcatccttctccgtcctcactcctcgacgcggtccacacctgggactgccatccgtctacctttagcaactcccatgaagtgaatcaagcgcacgggatggagggagccacgtcgtcggccattacggaaggggcgacccaaaagacatcaaatctaatggttcctctccctgatggcaaagttgccactttataagctgattgtgacatatggggcacgttcttacacgaattacaagggcgatcagtgcactgtcttcttgcagctgggacatccatgtagagaggcagactagttactatccatatgtgaaccttgttctttagctgatgcaactacaagttgttagaccttattgtactatgttcatttttttggagctcgctttttgcatggtccatatgcatgtggaccttgctgtttagattggaagatggatttgtactcctatgaagtaattttttgatatttagagtatcttaacttctttactatgatgatattattgtgcttatttctggtcaaaatgtcgtgt

>TaeST2.22527.1

cttgtcacacccatctcatcaccagcgacggtcgcgcctggacgcatcagctagccgaggaaaagcggggtggtgcgacggtagggagcccatgtagcggcggtggtggagacaaccctagatggtgccccgcgagccacccgacctccttgccttgccatggatctgctcctcccatttcctttccgtcgatttgctttttttcgcagtgccaccgtggattgggtcaatttggttttcacgtatggatctggtttgcccccatttcctttgcactaatttgggtttctccttgctttcatttttgcctctcttttcttctgcagctccactagttctttgttgctgccgctcttttggttgactgccatggaaccgtttggctgagatctctgctttcttcgtcgtcgtccatcggtccaagttgttcttttctgctctgttgcctttcggttgcttctgaagcatgctgcggattcgttgtgcctccttccaatctggtgttctgtgctccatttggttcatctaactcttcatgaaccgtctctatactcatgcatgtacctcgatgttaatttttttaacaagttgttgaagaaggcagatggttctaggcttctcgttgcatcctacactccctgtccggggaatcaatagaaagggttcgcccaccttttgagatcgagaaactgaaaggatccgtagaatcagaagcatcaaagaagtccagttggggcgggggataaagcgtcgggagatttgaaaagaaggagaagcaaggaaacatgaatcaatagatcagttggtgttacaagaggtaatctaagaagttgatctacgaacagaggaaggctagtcttgttgaaaggcccaacgcacccaacaactctgctggtgctggcatctatgaaaagggatgctgaataaccttgttgtcttctatccttcccatgggagggctactgattgatatagattgttgtgtttatttgtagaataaaaactgaaactcctgatgttggatttgtatttgtcatagggttttctatcttggtccgcctttttcattccatatatatgatgtctactatgcaactttattcttgtagactcgtgttgggcctccaagcatagagttttgtaggacaatagcaattttcccttaagtggatgacctaaggtttatcaa

>TaeST2.22831.1

tgatacgtgctggtgtgtaattagaagcaaaatgcatacagcacatcaacaagtagtacgcacgtgcacatcagaaagagcaagaaagcaagctcaaaaggaaaaatgtgtttagctggttgaacccaacctcggcagaaatatcatttttttatccaactagtcatcttccccagctctctttctctctctcactgtcactggtatgctcagccgaacagaaccaactcatctctctctccctaactacagagcaggccgccgccggctcgatctgccacttccggcaatctccggcgacaccgcggacacccacgaaatcccctcgactccctctaccatattaccccaattatttgatctgccaccgccaaaatcgccgccaccactcaatcctagcgccgcctgatctaggtccggtgggcttgccaccggctgcagcggcaacgacgtctccatgcctacatcccccccagcgcagccacacgcacgaggcatgcagccacgggcgtcgggcgcgtgcggcttcaccggcgctgctcggtcgccatggctctgttgctaacggtgtggactagccacatcctactgctactactgtaaggtaaagagatcaaagtgaatagatgctcatggactgattcaaaccgaggtggagatagataagaaatcaagcaagaagggataacacaacaaggtacttgattagtacctggtggcgcatgtaacccaaagatcttcaccgcagtgaaggacagcagtgtggcagaaggtgaaaagacaagtggtgggggtactccagcgtatgtgtgtgtgaggtagaatggtccagtgaaagggatggctcctatttatagtggggtgaatagctatgtcggtttgaagacacattacatttggcatgcatgcgtggttagtcttgcatgcatgcctccacttgtgtctcttgtgggttgtactatggacatgtgtaatttgcttggctggccattagttggcatttactttatgtgaaagaaatgagacaagggtttataaaatatatggaggtttggagaagaatttgttttgcatgactaaatgctatttgctctaataataaatgtagttgtttctattatt

>TaeST2.23083.1

agagagattcgccaagaaaaagaagaagcgagaggagagaaaagcgaaaccctaaacccccgtattcccagaccttcgatccgatccgacccgaaccgaccggcggcgatggcgtctgcgccggtggagaagctcaagagcctgtggaactcccaggtcatggacgaggagcagtgggcggtcaactatagagtgctaaaggctgcggggatatttgctggatccatctttttgatgcgcacctttggtgacatgatggtagtttaagggagtcgtagtggctgagcagggtaatggtggttccatgcccattttcgttgtgtgccttatgatcaggcttgtggaatatttttactcagacttgtgggtttcttgttaaggtggtgtttgaagcatttctcggcagggttatttgacttgttctttttgagtcgagcacttgtcttctatga

>TaeST2.23100.1

tatacggttgtcattcatatgcaaaaaaatccttacaaactaagactactcatattagaaaggagaaagcttatattaccatggagagaggcaaggtgattatgcttgtgatggtcatccttccatgcctcataatattttctcaggctcaagcaactgatcaggtggatagcggcaagattgagttatgcatatgtccaaagaaagatccaccatgcccaaatggtttgtgcttctgttgtgcactgaagcaagaacgttgctttaggactaggaaagaatgcatttgtgaacctggtttccaccaacctccttcagatggaagctactcccgtcccctctctttgccttaaaacactgtttcaaattgtcgcgatgaaaagtattgtcatgaatatcttgaagtaaaagaaataagttataatca

>TaeST2.23103.1

tagaagatactactcatatctgaaagcatatattgccatggagagaagcaagatgattatgctcgtgatgatcattcttccatgtctcataacattttctcaggctcaaagatctgatgatatgaatagcagcaagatccaaatttgcttgtgccaaaagaatcctccaccatgcccaaatggtttgtgcttctgttgtgcattggagcaagataaatgctttagaaccagccaagaatgcatttgtggttctgcttgccctcctttagaagcaacctcccctcatcccctccctttgccttaagacaccatgtcagctgattgttgccatgaacatcttggaataaaaaaagttgtaatcgctgcatgctcatctttatgttcttctgaattgaattttttatagttatattatgttttaca

>TaeST2.23555.1

cgcaagaacaatagcaggacaaagaaaactagctaactactctagttttccttatggctcttgtgaagaacaacgtgcagactatgtgcctggtcgctcttcttgtaatgtctaccactttcttgccctctcatgtagaaggaagggtgatagataccaaggagacgatcctcacttcgtgtttactttggaaaagctgcacaatcgatttatgcaagcaaaactgttcaatacgtggatatgctaagagtgaaagcagttgtgtatcatacaacaaagctaactactgctgctgtgcgacgaagtgaacgacaccgctatgatggataccatccatcaccccctgctacatcaaattatttcatgcgaatagcaaaaaaagaccattggatgtaattgatgtacctgattcaataaactttaaattttactagtagttggttaatgtataactgtcaagtg

>TaeST2.23557.1

cccagaagaactatacaacagaaggattgtgtagctattatccatggcttttggaagcaaaatcacatgggtactaggcatagtggctttgttggccatcttaatcactacccaatcatatgatacatcagctcgtatagggaattgtgatgatacgccgaattgctcagatagtaaatgcaaggatacatgtgctagccaaggttacgaggcaaaagcgagttcttgcgtaaaacaatttggaaggacattttgttgctgtagcaaggatgggtcagaataatgttcttacaagctcgtatgtgcaagcagcgaagaggaacaatatgtgtcttctcctgatataataaatgcaataaattactactata

>TaeST2.23981.1

taaaacataaataagtcatacaagcatcataatacaagccaggggcctcgagggcttgaatacaagtcctcgatcatagacgagtcagcggaagcaacaatatctgagtacagacataagttaaacaagtatgccttaagaaggctagcacaaactgggatacagatcgaaagaggtgcaggcctcctgcctgggatcctcctaactactcatggtcgtcgtcatcgggctgcacgtagtagtaggcacctccggggtagtagtcgtcatcgacggtggcgtctggctcctgggctccatcgt

>TaeST2.24082.1

tataataatatcctcccattttattaagtaagtaactagtacaataacctcttgaaagtagcatcacaaatattcaggattacataacacatgaaccacatatatcaattatatgttgagcttactacttgggggaaaataaaaccacacatcaagatacaatggatggtgcgggtgaattggccaatccgctaccgaacccgcaacaacagatgttcctagggcagcacactggattgtaccccaccggtctagcagctgcatgtgctagttgccatcatcatgagcaagcagacttgcgccatgagaaaaacaatgatcacttcgaagatgccattgttcgccattgcctaagaacacagaacaacagaaaatatataattatgcaatagtctgtcaacca

>TaeST2.24527.1

atacaatacacatgaatgatagaccgccaaccagattaagaattagccatggctcgtaaggttttcaacgttgtggcatccatcctcatattggcgttcctcatgtcttgtgatgtggttcaaagcacttgccatgagtataccagcaaggtcacttgtcatgactcttcgacatgtcagacgtggtgcaagtacaatggtgccgatgatggtcactgcaataaaggttcatgtgtgtgcctggattgcgggtggcgtgtgatgggttcaacttgagttgttccattgtatccaaaaaatgtatttggtgacaagtggtcttcgtgctccttgaataaatgtgttacaataaatcaagc

>TaeST2.24604.1

atgcaaaattaaagtagttttctcctcagtcctgtccatggcagtcatcaagagcaatcacacacatgccatgggttgttcaatcttgattgttgttatgatcatgtcttccactttgatcttaccaacctattgctatgaacttgatcttcatgaagaatgcttgcctattcaagtctgcaatacaaatgtctgccaaaacttatgcagacacgacggcaaccgttggctcagggcctactgcaggactggaccaggcgatctgaaaatatgttgttgcgcttattcctgaatctctagtgatgcttgttgttgtttgtggcactaatattgtcgttctctgtatccacccgcttggaatgcattctcaataaaatagttttttagtgactcaatcaaaattttaattattatgaccaacatcatatgtgtatgatcgatatcatgtgcacatatctagttagtggctcgtctttacgtcttaaaccagcgttgaaaatattattgtatcgactgtt

>TaeST2.24665.1

tagctagaatgttccatggtggggccttataagatattgaaaaacagttacatagttgaatccaacgtacattgcgacaacttttataagagaagaactatcggcttcaaaagaatacaacaaagtactcttgttccaatcaattaaatgccgctttaaaaaggagaaactcaaattgatggggatgtatggttaaagatggcaggggtgtctttttggcacactttcttgcactcctccattgatacaaagcaagcttccagcactagacacaagtagcaataattacccgagcagcccttactatagatgcacagtccagtcggtaaattgatcttttcattcttcatgccctcgatgatccggc

>TaeST2.24762.1

cgcgaccaaaggccttttttccaccagtgaaggaaataatcgagcggagaagagggatcaggagttgaggatgcggctctccctccccctcgtcgccggcgccgtcgtggctggagtgatttctgggaatgtgatatttgggccgccgctccagaagtactgggcagagaagcagcagcaagatcaggcggcaaaagaagcccaaaccgggtcgacttaacgaagatatcctcatgttgccccgagtcagtgtcgccatacttccctcgatcagtcgcagacgcataattctgaaactagttcaatgatgtgaaaatgtacttgggttattcgatagcctcgttttttgttagctttgcttgaattaactgccctatctttggtactggaaggcatcgtttgtaccatgtacagaatgaattcgaatccaagccaccattctggtcaaacttttgtttgaattcagtttcatttctttggttcaaa

>TaeST2.24951.1

aaattggattaaaaggaatactgggcagctggaagacacaaaccatattcaacaaactactgctaacgtttttcgaacaaggatgaggggtagccgcatgatcattgttggtcttgttcttctggtgttagcctcaggtacggcgacgggatcgacgatgggaggatctctaaccggaacaaattgcttagtcaaaacccctatgccttgtgatgaaatcaagagatgcatcgatacttgcaactccttgtctcctgtgcacggcaaacctgaaattagttgcgttgacaaagagtgtagatgcacattttgttgaagtttgacatgaatctacaagcaaggcatatatcttgttatcggagtagtagtagtagtaatcattgagtgtgtactggcaatataccaacggtgttctcaaaacaataaatggcatgaaaaaaaatccaattgcgtacgaaacatcatgtataaccaaatcgctagcgggctgttgctagcttat

>TaeST2.25087.1

cggaatcggaggtcctcggtcatccgatccacccgccccagccaggtcgtactccgcctcctcctcatcatcatctacgggattctgctcgctgcctctatcgcgaccttctcctccgtgccccaggccgcgcgaacgctgttcccctcctcaccgacgggatctcaaccgccggcctcacccgcgagagcgccgccacctaccttagagggaacggcgccaccggccgcaagatcatcacaggttattgggtcaatgttaggccccgggcggcgatttcatatgagcgaaatatgaaatcaataggacacctacgagactggttctgaaaaacagtaagttaaaggatgaggtacggtcactaaagaggcaacgcggtgcttaacgatggggtgctggggcggaagcaattggatactctgagctggagcagatggtatgcggtgctcgacgaccacacgccggagtgcgagggatgagccagatgctggagcagagacgatgtcggatcacggccgtggcgactgatcagctggatcacggcgcagacgatggactagtttagcagaattcaaattcatgtatcaagggtagaaatgtccagtcatttcatttgcttgtccgctttactttgttgcattatcacgggcagtttagtcttggcacttaattaggatggctaaattatatttgagtttcatttgatggatatttactgtgagaattatgatttttatgaatctacatattatatgtatggttttgagtattgtaa

>TaeST2.25111.1

cataggcaaacaatccctacaaacgaattcgaatcattatatcaggaagcttatactaccatggagagaagcaaggtgcttatgctggcaatccttccgtgcatcctaatattttctcagggacaaacaactgatcaagtggatagtagcaagatccaactgtgcgggtgccaagagaaatctcatccctgcctgaataatttatgtttctgttgtgtactggagcaagatgtttgctttaggatcatcaacgaatgcatttgtggtcctaattgcacctctttacatccaaccttccctcctcccctctctttgccttaaaacactgccagattgttgcgatgaaaagtaatgtcatcgatatcttgcaataaagaaaataagttgtagtcattacaggcatagctt

>TaeST2.25118.1

acttgtcagccatatgcaaaaaatccttacaaactaagactattcgtatcagaaaggagaaagtttacatattactatggagagaagcaaggtgtcgcttgtgatgattatccttccgtgcctcttaatattttctcaggctcaaccaactaatcaggtggatagcagcaagatcgcaatatgcctatgacaagagaatactccaccctgcccaaataatttgtgcttctgttgtgtactggagcaagaactttgctttaggtccaggaaagaatgcatttgtggttctgcttgcggccaccctcctttagatgcaacttaccctcatcccctctctttgccttaaaacactgtttcagattgttgcgatgaaaagtattgtcatgaatatcttgaacttgaagtaaaagaaataagttataatcat

>TaeST2.25461.1

tttggaggttttgggtgcttgttgtttaggttttgcgtcaaggactcaaggtttggtattagtgcttatctgggtggcatagtttgtgatgttttgagggtaccccgaatcttagacatgtggttgagtattatcagtctctagtaatggaaatggggagaaatgctcctaaatcttaaaaatggaagaacatgggtcatgatgctagctgagtatcgtgctcttttctcctagatgtaattcttgcaaactgttctctaattttcacttggtgttttcactgaaataatctttttgggttgtttttcctgtgatctgtgtgcatcttatttgtcctctctgccaagcagcgtctacaacgtcatctcccccaggtagtgcatcgtctacgtccttggctgcaagtagtgtcgcctcgcctctacttcctcgtccgagcagcactgcctacgtcatctgcctcgtcgctcgagcaacaccacctcctctacttctagacagacgatattgtgacttcacttaggagaggggatctcagattccttaagcaagcccttgatagacatgaagatccggttataaaatgtggtgtctacctcgttggagaaacttgaactccaggtctaccggagattagtgaagaaaatctgagtctaaacttgaaggtgtccttgtcacaaatttacagaatgaaccaatccttttcactttgctttattttgcagccatatcatcgagagggagaaggaatcatctaaggcgcatcaaatcgagttagaggtctctgcatccattttagaagttttgccaaaggtgaccacgacaaactttttatcctgatacaagcacattatttgaatgagatggcgtctattgttggtggcttatcctgatacactagttgatggaggattggataatctacaagccgttgaaggtttgcaagacctacaattaagttggtggattggtaatgtaatgcgccttgtttgttgaacctggaaacattgacaaattctgtttgttttggcacatagccatgtatgctcgtttttagtgtgtgacacatctagaaacatatgtacttttccctttgtggcactaattcattttggttgcgtggcacatctagaaacatatatgtacttctccttttgtgccacaacctgcttatacgtttattgaatgaagttcaagggtt

>TaeST2.25594.1

tttgctcaggcgaactactgaccatcaacaaaatacaaatacaaatacaccgtgtcgttcaacaaaagcattcttccaggtacaaaacccaaacctctgaacacagtgtaacatataacatacaggcagtgtttatgttcacgggtggcaacaccaacacggaggagtgtacacccaaaatgtctagtctttttatcacacaagctatacaaatggtaccagtcttccacctctcactcttcagccggaacctaatctcatcctcatcctacccgatcataatatatagcaaccagtaaatttactctacctcggctagagatattgctcgccaaaattgccctggtaccaaaagaatattaactgaaacaaccaatagcccaaatcaatctacaacttgaaagatacctttgggatcctcatgctccctctgtaaaaaaagactgaaacaatcgacatcatctatgttcttcaaaagccaactcagtagtttcccattggaggcatcccatatgccggcatc

>TaeST2.25642.1

gctggcctccggctccgggctgcgagcggcgccattgttggtttctccaagtactggctagtagcttgccttggcttgtttggatgaagggttatttagctcgaagggggcatatatatagtggattttggaggcagattgttaggactagtcttcgaatttactccatgaacatgaattcgagctacatctggtttgagagaaagaggggatcaggaggaggaagacgcacacacgccacccacgccacaactggcctccctgtcctgccgt

>TaeST2.25674.1

aaatggacaagtacatgcgatgtccatcatgaacgatgatgcagactagatctacaactacagaaccatccatcacataaacacaaagtcagatgttttgcatcaggaagaagcagtacttgaggaagcagattacatgttttcccgccatgaagcactagataggcttgttctatttatttttgctgttctagagtatttcagcagctgaatgcttgactacaacatgttgtaggttgcttggatctgtataaaatacgtatgtgatggaaatttgtagaatgtgcgttctgagatatttccgtaatattcagctaattggtggtaacttgattcata

>TaeST2.25697.1

cggcaacaggatttgcagagtacaagggtgggctcatcaaggtgtgtggtggtaggctcgctcggccccagcatcttttgttgaacggacatgccgcctcttcagcatcggctacatgagagccatggtgagctctcttctccctgcctccgtttgcttgctctgagctcgtgcttgtgagaaactgcatgtcacctcgcttgaactccctagctacaccacaccttgtttaatacactagtcatgctcttgcatacatgttttgttggacgaaaaaatgtacaatgatctgctccttgggatattaattcttatgatatatcttgtaatttacattattgtaaaagttaaacccactacacgtgttagcagaaattgttttagtttgtcatacataattgcctatgcttgttggtagctattaagtgtataattcaccatggaagcatagtaatttcatggacatcataagattacatttatgttctgaacttaaatatttaaactcagtgtttagattctgtatctgtaatcataagcttcacttgtagtctggtcgttcttagacatattattgtttgagttaagttcaccgaacaatacttaaaattttaggaatcaatgatgccgacctccttgcagagttgtgccaggtaccaaatgatgacctactcgcatagttgtgatagttaccgatgacctcctcatactcaccggtaacaagtctggtttcacatttgctgggcactatgcttttggtgtatcatatggctgggagaaaacacacagtttcctgcattgtcggataatgtcaatttgttctgtaagatctgagtttattgtactataaatggtgataatattgtgcctagtatgtccacgaccttttacattgtatggataattggatagactgaactaccgtattcccctttatactccgcagtcaaactcatatgatagagtagatattttctacctgtagaatcatggaggtattaactacctgatgtagtcatagaacaacatcatgtttgaatatgccaaatgtttgggcctaatgatgttctgtattatggcagctgctttattactttctttctaggtttatgtgcagtgttggattaatgatttaagtgcattagcggtttatcttcttgttcacaagtctgtttgcattggatgtacataactgtcatttttacatcttgcatatttaacatacatgacaatagtagtaggtattgaaatatatgcgaaggtagaagctatcgctgctgccttgtgggtcgtaattctttctctccagacgcgtatactgcctgatgtatcgctcatgctcatattctgtattgagatagtcctaatatccatcgggttgtagattgatttcccctgaaaaagctatgcttctttccttagatgataagcatgttcgacagctatgtggaatccttaagataaatggtctttagtttcttttggtgcatctgcgtgcacataacagtccgttagtttctctttatataatgaagaaaatatgttgttatcatttgtttattattatctatcatctgtagattgatttttaggccatcagtacttctatgtataatttgtagtctcttgaatgaataatttttctttaggtgtcatgtgtcttgtgttactacatacatctggcgtgaagtacatggttccagaactatggtaacccgtagtttgctctactggtgatagtaggatgatggaaacatcactactaaatgtagttcatgtttcttcaactaatcatcatgtactagagtttctcttcttcttgctactgtttggtttttgacgtcactagcttgtactgaaacgtgcagactttaccccttgctagatcctttggtggaggtgacccatgtgatggtgattcgctccagcagtttcggtctgacactggtggtgctacactactactactcctatgatggcgacgcattggtaggctcggctgcctccctttgttgtcactgtgatggtgaagctcagcctaggctacaaccacttacgatgagctcctcaggtccccttctacccctcctactcgctccatgattctttgctttgcctttgtgaggtgtaattgtttaatttttgtcatagtggagcattggatgatatattgtatctgtcatgaggtacatggttcatggatatgccaacctgcagcttgttgtactagtgatagtaggataatgcaagttttgacaatcaatacatattcctgcatgttagtgtctctccgtgtaagtactaattctggtgagaagaatacaattattatttaaccagtccagatttttaacatttgatgtacttccaaggcacacagatctctactcttctacataatccacactttgttctggaagcaagtcagttgtaccatgctgttacatcacaacaaggtgtcatttgatgtagtgctgctgttgatgctccctagagtctagatgtagatgtactgctgctgttgatgctctagctgtagcctgtactattgttgttttgcttcttcagtgttagtttcgctactactagctgttggctgctactagttagtacttagtagttactgctagcagtgctagtagctgctgctgcttctattgcttgtgcaattgtgctagtagccagtagatgttcattgcatgacctgcactcgtagctagtagatgtgctggtacctgtacgctagtattgttggtgtgttcaatgtcttagtagaacatcatcaggttgatgtgttgtagtcaagttttggatgttttgccatgccatgcttagtgtttactaaactgaggtatttgagcatttatgtgtaatttctattctcttgaatggatatttttttattggcaatcatttaaattccactgtatctcacttataatttatgcagatcaggatctaatattattgaggttgttgttgttgtgagcatgatcagaagaagcctggagatgtgcacaatagttttagttgatgttgccaccatgttttggtgcatgtgtatttatttttgattgtatgcaaacttgatggatcatgcagacgtgtatgtgctgttgtgagtttcattgattctggatttgatcatttaattgagagaactattgattgtttgctgttga

>TaeST2.25751.1

cggcgtcttcaacggagattggatctgagctctcatctctggttcctctgtgctgccggcgtcaacgtagcgaaccacgctccagtcagctgcacggcgtcaggtgcctacaaagaaggttgacaaagaaaatagtaatctgaacttgcaaaatattcatttacagagatctagtcctaataagactccaggttcaaggccaactgctatttctaaaaatgcgctcatacaagttgcagcaaggcattcttgaatgtttaaagcaatggatccaaagaactagctggactagactctagatcatggacaatttagtcttcgcagttaattaggatggctaaattacatttgagtttcatttgatggatatttgctatgagaattatgaattttatgaatctacatattattcgtatggttttgaatattgtaattgaaagcctgttttttaatggttgcaataggctat

>TaeST2.25844.1

cccacgtggttcagaatcatctcgcccccctcgaggatttggccgccctgactcgagacgacgccgcgccgcctcctcccccaagacgtcgcgccgcctcctcccccaagatgccgcgtcgcctgactcgacgccggcgatctcctcaggcctactggatttctcaccggtgcctgccgctccgattcttgttggggtgcacgaattacctccgtattgggatccagttgtcacgcctaatttcggttctcaagcaccagaagaagcaacaataaacaagctggagggataactttctctcagattctcaaagatagatgatcatgacaccaaaagatggatatatctgtatgcgaaattataactgatcttgagctattccagaagtacttgtgttgtgttatatttgctgcaaaattatgttggggctgtcatgaactgttgttgctgcgaaagatcatgttgtcatgataaattatgattcctgggatttgggattcgaagtcatgttgttatgataaattatgatgttgttgtgatgatatattgataatgctgctaaattattcccctgtagcagcttttctcatttttattaggtggcctttacaattctgtagcaaggatataagggagttagttacattgtaaa

>TaeST2.25930.1

agcaccataccagcagcagacgcactcgagtaccttctaggctctccgttgccggttgcgtgacgtgccgtcgcccgcgcctcccaccgcagcacgccggccgagcgcccatggcgtcacccgaggacaccttccacccgccattctccttcaaattcgtcgggatccggcctgatccggatggatccggatgccgacggcccattcccgacctccctgccttgcctggttcgtcggcctctccgatgtcgtcatcttcgtcacgggatccagcgctaccgatggctggcctcctcctcgcctggatgtgaccccccccacgtcggagcctccaacatgagcagcagcagcgaacgcctcgacaagggcgtcttcggtgttgtctttattttaattttattttaaagaagtttgccagatcagtggccagggaaaagccatggcgatgaagtctgattgatgtctcgcgagcgctcggtggcgcttgatgtggtggatgcagaatagtatagctcagagtagtaggccagatctgaaaaaaaattatctgtacttacatttttgctgtgtatttcttcattttttgataaagttatgtatatggtatatatatatatatatatatatatatatgtgcaaactccttttttctataaggcaatgagcatatttaatcagaatttttttattgacttaa

>TaeST2.25994.1

ggctcaccaccacgccggcctggccatggcggtggccggtggtggagaagaaggggagagggagatgagatgccaggcccgatggggaatggaggcgtgctagggattttgatcccccatgatctgatttgtcttttttttactttggccctcctcctttcttctttcttcccaagtagatcctctcttctatagttgccccccggctacttcgtttcttctcgcacaagtccattcttcctcctcttcttcgttctcttagccacttagtccggatcttgaagtttatagtgccttctcgcacttatctgcaaaagaaacaaatgactagtaaatgactctttatatgattttcatgcaaatattcaatattttacagcaataaatgatggtcatatatcaataaaccgcatatttgagtgacaaaagatgtgtatgaaatgtgcttatcaagttcccccacacttaaacttttgcttgtcctcaagcaaaggaatatgacaagagctagataatgaacagtgagctgactggagaatgtccgagtgaagtagatctccaaacaaggcatgctttggacttaactagtgaaaatcaatggttaagagtgctacagagcaatagaatactagtaagcatgaccattttaaacttttacaatgataaaagaggatcaacaagtttaaatgatgactgtgccaaatggttcctaaagagagcatgatcccaagaacaaaatgaactggaattaaatcttgtgatcaaatcacttatttacagagataaagcaatgattatattattattagtctcaccataggaacataccaccgatcccgagaacatggtatacacctggctcaaccaattattagttttttttgtttgtctccccaaaggaacataccaccgatcccgagaacatggtatacacctggtttgacaattacatttttatttatttattactgtccaagctaccagatttcacatgccaccgatccagggaacatgacatgctactgtaggtagaccagacttatttattaaatattacttatcttaaatgaacaacgcataagcacaaaaacaggaatcatcacttctccatgtctggttcacatgctaccgatccagggaacatagcatgctaatccatagtggttaggtgattgctctcaatgggaacacacttagcacaaattcagcatctaaacatggtgatctaggtgtatcaaggtaaaagcagaaaatgatcaagttttctagtgtactagggatttgagcactcaaaactaataacttccacaaatttcagcaaagcatgcatggtgtagatcactagtttacaaggcattcagaaatcaagttcacaaattcacatcaagcacaatgccaaaggtgaatttcagcttgacaacaagtaataactagctcaagcaacccaactagcaattgaatgcagcatgtatttatgatattcagaatgggtcatgaaacatctcaccgggctttgatcatgtagcactcgctcgatcacctcaccagctgcagctcttctcccttgcttcctcatggatgctccagcttcactcttcttcatgtgtaccccattgcttctcctcatcgccatgctttgagtccaccaggatcccatgggatgtcatctcgaacttggtcaacccacaatgaatggtacacaagcgtaaacctgaaagaacactcaacaaacaagccaatacaaatgatagggacaatgccctctaagtcatcaatagagtatcctatcacataagcatatcta

>TaeST2.26073.1

ttaaatcacaagtagtaatccgaatttatacaaaccactaactaacaaacaggagacatatatataattgtttcacatcaaccacacagtctttataattctcagatgcatgagctaacacatagataacaaaaatgatcaagaatctgcaacaagcagtacagaagtgacaattttaactaattaacacacacaggaagtctgaactgaacaacagtagattactaacacttatcaaatctgaataatacaaatcatatgcgtctcaatcttacagaaatttcaaagagagtgagaaagaaagaaagcaacaggaggttgaggtagtagtagcagcagttttgttgtttagaagagaacagcaacaattttgaggttcaggtaagaagaacagtagcgaggcagagaagaggagcagcaaaagtgatgtggggaagaagaactagcagtaggcaggagaagaaggagcagctggagcgcacaagcagcagcagttttcagcagagaagggtaccggcagggaggcgcaaggaggtggagaggatgaacaacaacaggggggttgcaaggaaggagcagcagcggtgcttggagaagagcaacaggaggtcaagaggaagaaggaagatgcagcagcaatagcaggttcagagaggaagcggcaggaggggcgcgcgaggtgggcgctggtggaggcgcggtggtgaagtggagctggtgacgctggtggagtggtggatggatggcgcggcgcggctgggatggatggatggtgcggcggatggtgctcgtggctgagtggaggagcagcaggcgcaccgccacgccggcctggccgcggcgacggccggtggtggagaaggaggggtgagggagagaggtgagatgcgaggcctgatggggatcggctgcgctagggatttttgatcccctcctctgttttgtctttttttactttggccctcctcctttcttctttcttcccaagaagatcctcctcttcttttagtttgctcccctacattacttctttccttcacgctcaagccttgacgtatttctttcatgcccttctctcttctcgttagccacatagtagaagtcttgacgattatagtgcatttgcgcacttttctgcaaaataaacaattcactagtcaacattctcttttatggttatcattcaaatattcaacaagtcatagcaagaatggatggatatatctcaataaaaggtgaatttgtgtgcccaaatagtatataagaaatgtgcttatcaagttcccccacacttaaatctttgcttgtcctcgagcaaaggaaaaaaaatttgatggtgacacaactcacactgcctagtaaatatgcactttccgatagatagtaattccgacaatactggaactaagcaaatgtactctactgaagctaaattgtgataagaaatattttataacacactgtaaatgttccaagtcaatggccttgagtcaaagtgaatactaataatcaagagctggctctgtcttcagccctgagataagctgggaccccaaaatcaattagaaaaacaagttacttattgaatttaaagcacaagtgatgatattatatcagtctcaccaaaggaacataccaccgatcccgagaacatggtatacacctggctcgaccaaattattagtatatttttttgttatctccccaaaggaacataccaccgatcccgagaacatggtatacacctggttcgacaaattatagcatttttatataatttttttttagagtccaatctaccagatttcacaagccaccgatccagggaacatgacatgctactataggtcgatcggacttaaaatattataattattttatttagttatttgaaaattacttagcttaagtaaaacaggaataattgaaagaggaatccataactaatggcactgtttttcacatactacagatccagagatcatagctactacttaagtgccaccagttacttagcttaacgtgggacagtcagacacagcacatcaattcattagtaggtttcacttagactcaaggcattcccattgaacatttacatggaggtataaagtattcattatcacaaattaccataagaagaatactgctgcatagttttagtattaagagaatcaacaatcttcttggagtgaatcctactttgcagtgaagagtaaggtgtcacaatgcaaaaagcgtttcaatgcaataaaatgccagttctcgtactaaattttctaaaggttgcttcaagtggaacagaatcacctggtatttgtatactcttctctcctactttcttctctccactcttctctgtttgtgttccctgtttgtgctcccctcatcgccatgctgtgactccaccaggatcccaaggaatgtcacctcaaacctggtcagcatgtagtaaacagtgcagtagcaaaagcctgaaataggactcacacaaacaactacaagtgatacggtcgatgccctccaagtcatcaatggaatatccaattatataagcataactataaagcacatgtttaggatggcagaaatcagcatggtcaatactcacacgaggatgacatttatcattaagtgcaagactagcatgggagtgatatgtttcattaagcataggattagcacgaggaaagtaactatgatcatcacaaatgagagtaataccactaacagggtatatatcatcatatccaagcaaatgagttttcaaatcaatcttaagtgatagtatcatgtaaagcaaggtagtagcaaagaaatcacaaggcatcatgtcatcaagaggattgagtaaacctgacacatcacgctctggtatgacaataggcaaatgaatctcatgatcttcatctttatctacagcagcttcttgtagttcttcagtagtagatgatgagtcttcaacttgatcacttgggaattgcagctcttgaccatcttggtccttgtcttctagttcatcttcttcaatctgaggtatctcttgctcaaggcacttgctaacttgctcaacgaaatgtgaaggtgttggaacctcacatgtatcaaggggaatctcagcaacactcagctctagctgtgaagaaatagagctgccagcgttgatgttcttctcaatcttctttacttcttccactagctcattcccactcttgatcttcttcatttcatctagtataaccttcctaataggatcatcctcggcactccaactgaactcaagactcaaaagtgtgaacttgtctatgtcatcaaactcatcttgagtaggcacttgtgttgaaggtgttggttgaccaaatggaggaccatttaactccattggcaacatctcctcgtgctgaggtatatgaataggagctgaataagactgttgtggagcatagctgtggctctcctcttcatacctaaatccctgcatatgattactagaagcaagcggtgagatctcggggttgttgctcctatatgacatattcgggttttcaggacacccatgtgagtaattgttttggtatgagctatgctcttgagcgaagctcatgccaaaacaatctggagtgtgagagtaaccacgctgacattcagcgggcgaatggccctgaaatccacaaatatggcatgtaggagcaacataaggattgcttccggaataaggataataagaactaggcctatcctcaaaaactactcctcgctgctgagctaaatcatctaattgatgggaaatcttacttatcagttcttgaatactttccgtgttgtttgtagtgttgctcgcataagcatgatgatctgaatagtaagccatatccgagaagacaattcaacctacaaaatgaacaagaaaagaaagagaagataactagaacagtggttaggggtaagagatatatcacaaatatatggcaccagatagaaacagaagcaaaacctagtctatagcctaacacggtcacacgacgctagtccccggcaacggcgccaaaatgatcgattgaaagtactgcgggatatttcacccgaactgtgagtgtcgtgttctccacagggactaggcgacagcaacaatgctcggctaaagctaggtgacagaactcagattataaatgcaagaaaataaacctaaactaacaaggtagttcccgaagatcgtcgccaaagaaaatgtaaaataagcaaaggggaggttcagattgtctatattgtcttttttggtgttttcctgttacttgagaaactaaaatgctaacacaaaagggaacaacaggacatgaacacatatatttatctaacaagcgtatgacagagtgaagaaacaagataatggacaaggatgaaaatgcttcatgtcacaacatatattacaggtacagagctaatacatagactgaaaattgaatgatcacacaactgactaccacagagaacaatttcaatacaaaattaaatcacaagtagtaatctgaatttatacaaaccactaactaacaaacatgagacatatatataattgtttcacatcaaccacacacacatatgtctttataattctcagatgcatgagctaacacatagacaacaaaaatgagcaagaatctgcaacaagcagtacacaagtgacaattttaactaattaacacacacaggaagtctgaactgaacaacagtagattactaacacttatcaaatctgaataatacaaatcacatgcgtctcaatcttacagaaatttaaaagagagtgagaaagaaagaaagcaataggaggttgaggtagtagtagcagcagttttgttgtttagaagagaacagcagcaattttgaggttcaggtaagaagaacagtagcgaggcagagaagaggagcagcaaaagtgatgtggggaagaagaaccagcagtaggcaggagaagaaggagaagctggagtgcacaagcagcagcagttttgagcagagaagggtaccggcagggaggcgcaaggaggtggagaggatgaacaacagcaggggggttgcaaggaaggagcagcagcggtgcttggagaagagcaacaggaggtcgagaggaagaaggaagatgcagcagcaacagcgggttcagagaggaagcggcaggaggggcgcgcgaggtgggcgctggtgaggcgcggtggtgaagtggagctagtgacgctggtggagtggtggatggatggcacggcgcggctgggatggatggatggtgcggcggatggtgctcgtggctgagtggaggagcagcaggcgcaccgccacgccggcctggccgcggcgacggccggtggtggagaaggaggggtgagggagagaggt

>TaeST2.26128.1

atttctcaagcattggcaaggctacggtaacggggagtgtcagcaactacaattaattcatttatccatggaattgggataacaaaaagaggtctcaagaaagagaaaataacttgtggttcaagcagcaagaagatagccagcctaaatgaatcaaaacaagaacaagcgtcggtaatttacaaatgaggaagtgtagctggtgatgtaattactttcaatagcgcttcagctctgcaatcgacagtttgacctcatgctagggcagcctcagccagttgcctcttctattaaaggtaaagaacagaagcctttttattgatattgctgtatggaaagtttactggaaattatgattctaagtgggtgtgaataacatgctttgtcttgaaataatgacatatggagaaactatagtttccatactaaacttggtattttgattcacacaaagtttctgtctactgatgattaaactcatatttaccatatgaataaagtgacatcaaaaggaaaatgaacaacagagagaattaagtatgccttataagcatgttttcacttaagatagatttgaatgacttcacaaatattcctgtcaatatagattgtaaagtagcagtagcctatacagatagctgcagttgttggctgcacatgacaacttcatgttattctgcgctcaccataataatcaatttttttgttccatgtaaagtggcatgcttctttcttactaattgtcaattcttttgtatagggtccctcagctacttatgttttgctagacaatgagggtatgtagatagcatgtatgttttttacattgttttgcgcaggttgagatgcatcacatgatgttttccttttacatttatgggtattacagaaccctttctttttccacaaggggcagtagctgtgccattcgatttggaggacaaagagttgatcacaaaggggtatcattcaggatatatacacaaaagagtatagaaccaaattatatgatgtaattgactactatctattaacttctcatgtagacaatgttcacaagttcatgtattaagtacattttattgtgtcacttgtgtttactcattttttaatgcaagtttactgctactcggagttcctaatcgcagactcaactatcttgacctaccgatgatctaatttgtggcatta

>TaeST2.26133.1

tttaacaactaaaaaatactgatagaatttaactagtacagcgagaagaaatacacagggtgaacacacatatatgctgacagaacactaattaccaaagcaatttctaatagatcaataatctcccaaattaacaatatattttaccagtaccaaactgaacaaataacaagttgcttgcactagtcaaatctgaatattacacaacccgaaacattacacagttctgaacattacacttctaaagtttgcaataagttgagagagagagagagagagcaacaagattcagattcagagaagtgcagcaacaaggttcatgggttcagagaagggggaagcaacgagcagcagagggagataggcaagggagcagcggcaggaatattagagaagagcagcagcaaaagggacgaggggaggagcagcagcagttgggaagaggcaagggaaggaggaggagcagctggttcgagttgagaagtggaggagcagcagcaaggcgagggaagaaggagcagcagcaaggcgtaggagaagaagagcagcaggggcgcgaggtgcggggaagcgctgtggtgctggtagcagcggtttggatggatgatggtggattcggggaaggagaagaagatggtggcactggtggatggcgcaaggtggtgatgtggtgggaggtgctggtggcacgccgccacgccggcctggcc

>TaeST2.26184.1

tcaccctggtatgtagcatcagtggagcgaaatgtgtcaatgccacgataacatccacatgtccaccttcaggcctccctgactcctcctgccgaccatgcgcctctgccatggcgtgtccccacattcccagccacgccttctcaagtactcccaagaagataacggcaataacagactgtgccaacattataaacttaatattagtaattattggacatacgtttaacttgcacatgaactatgtaaaatgcaaaattactaatgccagcttaataaaatgactccgcggtgtcgtacatggcaaaccaaagatcatacttggtactatctcaggtagcaacaaataagctaaaagaagggaaacttaattatagtgcattgttgtcaacaatgagatgtatacgcacattctattctgatagcacaaaatacttgctaccatgttcgtagagacccatttgaaataatatacatgtacataatacattctccagtgtacatttacataatgtctcacagtttggaagaaaatatatatgtcaatggcggtacataaaactaaaaatgagtgtatttgtcgctagggagtaaaagggcgtatcatgagtaactgtatccagtatacagagaatatttctctgtcaaataactccaatgaatcatagtaaattcaatacattgtttggaagaaaatattgatagaagtccattgagcgctgaggaataaaatcgttggggactgaaaaatatattgattctagcaaaaagcatttctccaagtgagaaacattcagtccaaaaccacatcaagaccagcatacctacaaaaagagaaaagattctgatcattacagttgctagcaaccgaaacctggatcctgcccaggtcatcaaattacacaatatgaatagttcaaatcgaaaggtcatttgtatccagcatagtgtattgcaattataacttgaaggggtatgcttgaacaaaactgtatgcggtccctctctcagtgttgacctccagtctgatggttatttatgcagagactactgacgctctcaattcccgactggcacaaaccagagcattgctaactggccaaattagtgaaaagtattatgtgctgataccttaaggaaatgggccttatttgccactatcctattaaattgttctctgtcacctctgatattttccaaatattattgttactttgaaagtaagttgtgatccttaaaaaattcagagccttgatgtacaaaaggcatcaggtttagccagagagcactcgaacaactgtatactgttatctacaacttccatagttacatttctttcatggctccgcatataggatacatcacgtcacatccaatgaaatggatgtgattactactccctccgtccgaaaatacttgtcggaggaatggatgtatttagacgcaatttagttgtagatatatccattttcatccatttttccgacaagtatttccggacggagggagtataat

>TaeST2.26187.1

tgcaagaattgaacatattaaatttaagaaatagacaaaatgtagcttgcatttggacatggtgaatccaaaatgtgaaaatacaaatataagatttcgtacatccttggctaacttttcatatgtgccgtcttggccaaaaactcatgctacaactttcgaagtactaaatctcgctaaattaatgaataagcatggagccaaagtcgaggcacacagaaataccacggaagccggagtccagattcatgatcttggttgtccctgagaaatagcagaaaaatgatctcgactattgatgcacaacttggcagcagaaacaaacgcgacagttcaagcctactggaaaaatgagctcaaccactgatgcacaacttggcagcaaaaacaaatgcaacagttccagcctactggaagaatgagttccctgctgatgcacaacttggcagcagaaacaaacacggcaactctttttgtggataaattaggctatgttcagaggcattaaaatattttacagtttagttatgcaacaacgaataagataattgccagattagagaaggcacacttctcacaaaaaaaagaagagaaggcctcactaaagaagcatattatgtttgtatctaataacatagagatgctactaatgaatataactaaactattccgtgagcaaaatgaaaacatgcatgtagctagaaagaatacgcatgtggctacaaaaaaggaagcaaacctcaagcaccaaaatagagttatagcaatcccttaaaaaagagttatagcaaagccgaagatcaagatgcagcaaaggagatcgtagaacatgctaaataaacagatatgaagtagtgagagcaggtaaagagaacttcattccccaaggcattaaaatattttacagttcattttgcaagttcaaaattgacaccatagcgtcccagaactgtagagtaaaacatcttgcatgctatgcatttctaccatgtttcactttgaaagaaagctcgattgcaactgaaatatctctccttgtatctcctataacctacacacctaaattagacaagcaagttagaatgtcattatttataaagaaacaccacaacaacctaatgaactttgtcccttctttaataatttcctttcttttctgtagttccaatttttcaacatggcgctatgattcatccctatttaatcttacattgagatccaaatttagtgtatggcatatatgttaatacacccaccctaaaatctgagaagagcaaaaaaaactgagaaagtttccttctttattctaagttttcgttgaactggcaactcaagttttacgc

>TaeST2.26210.1

aagccatagatcagatccaaatccaagcatttcttagatggccggcgcgggcgaccaccatgagtgcttaatcaagatccacacaacacaattaagctacacagataaccaggcccgaacacacgcccgttctttctacaaccgccacacgcacaacgagacgcattattcagcagtccagtatagctagctgcgtaggctcaaactgaccatctcgtcggacgcgcgcgcgcagccatgctcgatcgctacgagccgccgccgttgccggtgtcgtcgtttcctcccccgccgccgctgccgccgctgccggaggtcgcgggggtgccgccgccgccggcaccggtcaagtcggcgatgatcgtcttgatgatgctgccccgcttggggttccggttcccgtcgggcttcctcctctggccctggccgtccatggcgtcgcgcgcgtgcaagcaagccccggcaggccggcccagagaagcgtaggcgagtggacgagtgtggtgggtgcgtgcgcgtgagctagagtgggaggc

>TaeST2.26271.1

gaagagttgccatgggagcgacttcaatagaaaagcaggttgtggtgcaaaaatagaaaaaactgatctgttccattggatgggagattgatggctcagattcgggtggggcggtctgtgcgtgaaatttgcaaaaaaaagtccctccctgatagttaatttcgtcccgatgcccctcctcattgacccaaatcccgtggaggcagcacacgaaccaaatcttctccgagggcgtcggcggtggccgtcgcgcagatccctagtccaagttgggtgagcactcctagtagagcttccgtcgtagattcccttctgaagcttccaacatcggtgggttctttgcctcctctctcaagtaacaactagcgccctcctccccctcgtcggcggcaagccggtggtggtctcctcggctccttcccctccagtccgtgcaacgaggtggttcattttcctggtggcgggctcctcggctccttccgaaccgacctgtgtgacgtggtgcaaatctacaaggcatcaacacgatggttaatttctctggacaagaagttcgatgaacaagaagcacttcatctagaggcaactattgtgtgtcatcaagatagcaacgtcgatctagttcttaatggcaatgcttgctcagtgatgattatcatagtccttcaaacttagcatagcccgccatgtacgaaagttgaagcaattttctctagtaattcttgttgatcttgtcaggttattgatagctcaatatgaatgtcttcttgtgttatgcccggtatgttccagtttgacatgtactaaaaaatgtgccgtggatagaccagtttgacatgcagtcaagcattatgtgcctagaatggtccattttaacgtatacccaattttctgttcctgagatgcttcagcttaaacgtact

>TaeST2.26283.1

taggcagcggtctggactctgaagcatcctctttcttcttccggtcttctccctcttactgtatccaactgcacgagatctgaagcatcgaatcatgaacggaggaggtagcgcgacgcccttctcccgcaccaccaccaccctcagcaccacttcttcttctaccttgcaagcaggtgagtcgggacaaggggacagggaagctgctattcaagccgagatggccagggttaacaacctgccagccaatagttcctacgcgattcaccgcatcaaggtgctgaacaaacttcgccatcttttatctataaagaggaccacatctcaggatgaggagcttgaactccttttctcaagcctttcaatttaaagtttatcctcagtatctacatgtactaggtgatgtcactgtttcggtgtatctaatcatcgattgcccccgcctgtccagtgtagcatatattcccaatggttatcaagtttgaaggaatcactttttattcaaagtgctagggaacttgatgagttgataaatgatgtgttctcttgaacggaattcctagcggggatgaaatttatcatgtgcatgtaatgtttttgacccaaattgggggctgccataactctagccatatggtgatgcatagaacaccagctcaatagttatgttatcaagcatcactggttttgctattgtgacagtaaaggatgcatacctggacccctacagaatatgataatttggtctctattgtggaagcatcaaggactctgggaagcaggggaaagttcagctgccttgtttgatattttgagtggatacattttggtaaacctggaatgcagtatgtttctgactagtctggcaccaaaaaagacttctattattgtgctcctacatgtttaagcccgtagttgtaaactagtgggcttccgaaacggatcctgtttgaattacaatgatgttaggtaggcatgtgtgtttttcttctttctggtacttgtagtaacttaagttcttaaattaggagttggatattgtctcttctaaaagctgatgctttggtattcaggctgaattgcatttgcgaga

>TaeST2.26374.1

tgttttgtttatggtatttgcaaagctttaatcatggacactcacagataagcactactccctccgttccttcctaaatataagtcttttaagaagagatttcactagaaaactttcctacatacggagcaaaatgagtgaatccacgttctaaagcatgtctacattcatcctccctatatgtagtcttttagtgaaacctcttaaaaagacttatatttagaaactgaaagtaattgcaagttgtaccctagcacagaatgagaaaacactacccaagatacatatcatttaaaatcctttacatacaaacataaagggttggttctaaactgtttgccacgcccctaagaaatactcatcacaacagcctcgagctggctgataagtctactcatcagaataacagaaacgaaccgggatctccaacacatgggtgacctgctggaccagcatcgggacacgcggctttagccagtcaccgccgcaattattttcaagtacaggaacagccatatcctttagcaatatgctcagcagatcgacatgatttgtgaccggatcctcctcgaaacgagatatgcccaaggcttccttaaagcacttattcagatgtaagagcttaattgctgagttcttcacgagtaggatggcttccagggcgccccgttccagcttggcctcctgaaccatgagctggggtaacagatgatgctcgagcttttggatttccagcgacaagcctgccacgaccgagaggatttccagctccgggcatttccaggacgactccatcatcagcgggcagaggagttcatacgactcgcacgctatgcgcaccgactccagagagacaagcttcacaagcatgtgctcattggagtggcccagctcatcggagcgggcctgcagggcttggatttgggcatgggagtggattttgtagaccatcttttccttttggcggactgcagcagaacagttgctgctaccagct

>TaeST2.26381.1

ttcgttcaaggtcctggtaagtaatatacacaaattagtagtagtaatatggctactactcctgttgcagcttgacgacctctccagcctgagcaaccacaaactcacacatccaccaacaatcaaagggaagaagcaaggagttcgagcacaaaaaacatgccttttttcaatgccgccaatttggagtcgcggagtagctgtgaccaggaggaggccgtgagaggccagcaggaagcagtacaggtcgtgccgaccggtgacatccccgggtaagcgcgtgacaggccccgcgtctccgaccagtgggaggagacagccacagcgtccagcactacggcgggccgggggcctggtctccccggatcaagctcggcgaggttgacgacgatatcaatgtgcccccatctccccagatctcgctggtttttgtgcagcagaagagggagggagcccgttgggtagcacgatgaagggatggggagcacgagatgagggcggagagggttgcggcggtgcgactcgccaggatggtaggggtggctcgctgtggaggggaagggtgagagggtttgggcggcgggatctggatg

>TaeST2.26461.1

cgaacgcccacactgcgacacctctctctatccttccccattcctcttcctcccactcctcttcctcttccacccacgcccgaccagtcccatccgccgtcgcgcgctcccgatccggaggagcagatcatcccacgcccgagcccgctgaacccagcctagctcccgcgcccaccgcccctgtgcaggtccgcctcttcagagatccacatcgtgccgccgccagttcaggatccaacctcagcgcctcctggctaggcttgaggaacgaatgccgggagagccttcgccacgccggtggtagcggcggcgaggccaacgcgccgacaactactgttcaagggggcatgcaggatgtgcttccgcatcaccctctcctccactccaagacggcggcctcggacggggcaggaattggataacacgtccaccacctgatcgagcgcaaccgcaacagaggaacgccacctctgcctcgtcgtgcaggagatgcttcactagggccgaagtcaatgtgtcggagcaggtgcttcggggcggctgggtttgtctacaggtctgtcctttttgtgttgaatttgagcacaactagtctggttttgttttagcctcatttgtcttgagatgaacaaattcacatatatatttccagtgtgatgcgaggcacatgttaattcagttacacgtagtctttgatttatttgtactttgtttagcattcaggtatgtttgtttgtttgaaatatttgaaaatttgtgccaaggtgcccatataagtgctgattattcagtaagtcaatagacaagaaattgcataccttgttttttgatgatgatggaagtgataattttggtgttc

>TaeST2.26524.1

gaaacctagccgcgttctcctctgcggcacgcaacctttgtcccctctagatatagaacaacacgcctcgtccacccatacctctagaagtctagatctaggcctgacctgtagtttcctgatcgtgttcgcctcgatctcggggtaagctcagttcggcgtccatgatttgttcccatcacccgtttgttatctgaatcccaggtttctccttctgatcggcgagttggggcttgcggcgatcttggagaaaagacgggaggcaagctgcatctgcgcacggaagcttccgatctgcccggctggcgtctcagatctgttgctgatctgatgcttcacggcggatctggttgttcccggcggtctcaagtcggcgcagcggctggcggcgagttttccggcaagcacgtgggatcccgtctgctcctctacctcgtgcgacgacggccaacggctctttgtgttgataataattattcgatgatatgtcgataattattagatctaagagatcggaggtgacgtaaggtaagagcatgtaacaagcgaggtgcttgtggagagggcggagaagattgagaaaaaggagatgtgaacatctagaggcctttgccatgttgggtgcgctcgcatggcaggccgccaccataataaagatgtttttttgtctcggggggagatcgcacgggggttatcttgttttagccccgggttctcccccctgtgtgttggtgtgctcttttgtctgcacctctcgtccgatccgactacatggcacaacaattctaccgtcgcgaatcgatcatggagcaaccgacgaaagaccaaaggtacaatcgataatttacatacagctgaaccatacataatcattgacacatcacaaattttattacgtacaaggattatcttgaatccatataagtaactttggtagatagagattgtatgacaccacaaatttgcaacacctacagcagtccagcctaaacaaattgcatggcggatttacatattgttgtaacaattcgagtatatttggattcttcagtcagaagttgtgtttatttgtatgatattttgtactaaatctaattttaattctctctatataaggatttctttaataagaaatacatattagacccttttctgtggtctcatgcttcatactgtaagctgtcttacatgtagcttgaacagctgtatgtggggtctttgtattacccaaaatgaagcctacagctatccgccggtgaacacatctttctcgttggagcttgaagagacgtgagtgctctatggttctggagaaatctgctgaacacatcttacctcaaggaattacccgctcggaggccggttaaaatgcactcagcctactaatctggataatcatttattagagcgctattttaaccagttttgctttacatattatggtgaacagtacatccatttgtatcagttggttaaacataattagatattatatttgtggatattctctatattgtatgatcggaacagtatagcatatattctattaaattgttttatgtttcaactgttttagatgatccttgtttttgatt

>TaeST2.26731.1

gttattttccgccccggtcgatccaggcacggcatcggatgggagagagcaggggagggccaagaggccagcggtgtggggtggagtagcgaggccgaacaccgggcagcggacgcctcacagggtggaggagaggccggcgatagggaaaagcaacatcttcatggatgagatcctgacggctgaagatggtgtgcacgggaggagcttggaagatgtcaaggtgagcttctctcaatccttctccccttcacgccccttgcaattttttcttgtttcagatcatatgaaagtgagtatagccttgttggcaattgcatctgctatttctttatttgctttttccatttcctacaagaagatgccagacattgtctcatgatgattgtgttggctgcaagggcgccacgaggagtggagtaagctaaatacctgactggctggatcatttttgggctctttacagatttgccgacttcaaatgggtatctcatagttgaagcaaatggtggtctgaatcaacagcgtctttctgtaagttcttggaccacgttagatacagcaagcaagcaatacattgctattacctatacaaaagtccagtgccatatgaatccattgacaccactatgtaacttgaagtcatgtactatcactttgattgagacggctttcatcaataactttgtgctggaaaggcattagtactatctttgtacatacatagttgtattattcatcgaaagatcaccgatatttgctttcatctttttggtcacatgccatcagatcttctattgagagatatatttcttctgacctgagagcttgagaattagactctggtctctaccatttgctgtgatgttgatgtctcattttgtatagtccttagaaccttcacactatgccgagtaataattattgacagtgcagttccatttttttctcaatatagatctgcgatgcagttgttgtggccagcttgcttaatgcaactcttgtgatccaaatatttcatttgaatagcatttggcgcgatcctaggtaactaatgctattttgtttgctcactgctttatttcttaacattagataattgcatttttatatgatgtctgtctgtctggagcagttgtctcttgcttgttgttttagtgtctagagcagtttgtttgacttctaaagttcacttctgttagttataaacccgtgaatatttttgaaattccacttcttgctctaatatgtattggttcttcctgatgttttgtttctcagcaaatttagtgatagttttgatgaagatcgttttatcgggacactcagacaacatgtaagagttgtgaaggaactcccaaaagatgttctgctgcatttcaatcataatataagcagcatacaaaatatgcgaactaaagcctactcatccccagatcaattatttgttacacatcgaggtagataacaaacaatagaaatcaggaagtgttgtttgttatctacttcgatatcgtactgatttctattttttcacaattgcaggcgccatattagtgtttcttattatctaccttgatgccatactaatttctctgattccaaaattgctggcaccacttcctggtgcctgttggtgcaaggacccatgccttccatctgagaaatgtaaaggatggatcaattgacatagtcagctcatgtttagttctttgtctctcagacggcatgcatctctcaacacaatcctaggttaatgaactgctaaagctacaatgtagatgcattgtgactttattttctaggtactaacttcagatgtccaatttgttacctgtgccaatgtatcctcaggatatttactttagataaacaatctgctgcatactaatgtataattttacacaagctatcctcgatgtttgtttcgctttgtcattcccaacatcacatagtaatgcttgcagcttctttgggaaggtggaactaatacatacattcaacttactcaagttcgaaaactgctcctcttgacccgtttcagctttgtgggtgagtgaatgcatgatgatggaagcttggtgttctcataatacaagggaaggagctgctgagctaactctcatgtacttcacatgagctgaaagaggttggtatgatgctgcgaggcatggggtttgacaatacaaccttcctctatcttgccttcggtaaaatatacaatgctacaaaatacatggctccccttcgccagatgttccctcttttacaaaccaaggacactcttgcattgtctgaagagcttgctgagtttgag

>TaeST2.26845.1

ctgaccaccgtcgaacccctcaccggcgcccttcttctctcgttccctcggcccttaaccgtcgttgctactccaagaaaatcccaagccggagcccccggcttcgattcggacttcgccgccctcctgccagagcccctccatcgggtgatgtctgcgattggcatcactgacatcttgcccgccagccgtgcgtgccaccacgcgctataggggtagggggatgagacagcggtgaggagggagaggtgctgggtggcggatctgctgcattggtggagtaggtaggagaggccttgcagactcggcacacgtacggtgccagcagcgtcgtaggagacgacgggaaggaggggaggtggaagtggaggttggcggtgaggctttgcgacagcgaggagttgggagccgccggcgccggcggcgtgcaga

>TaeST2.26848.1

gcgccgcgctcgcgcgggaaatccggaaggggagtggcgtggtaccgcggcccagggtgtcgcccgtccgtccgtccttcccatcagagcgcttcaatacaacaacccccgagaggcctcgtcgcaccagcagctcttcccccattccgcgtgggggctagggtttccttcgcgctctccccgcctcgcccctcgccgtcgccggccaggggctcccgatcgggccggcgccggggcctcctcgccgcggagggcgggctccggccggctcgccctcgcgggatgcggccggtcccgagtgtagagcaatggggaatggagccaagctgctgatgatgttagggaagggcaactcaagctcatggaccaatgtcttgccgccacaagcccggcagttgtacatactgctcttatttggggcttggcaaacctaacctgaggctcctcaggccaagacccagcgcagcgactcctcgtaagaggaggcattcgtctgcacgtgcccatcaacaactcctgcaactattctccactaacaccgatgacctcgtccgggcactaacctgtcctggatgagctatggtgatacctacctaccgaagtactattataggcatcactaatctttcttccttagtttccctccgtcttcactgcggttattaccactaatattatgtaaggttttattttggtttgctgtgaaccaatttggaaattggtggatcatcgtggtagtaccatttcatgttgttggattctagatgcctgtactgacttttggcgaacgaatgaatgaatggccaattatatgacgccacactttctga

>TaeST2.26874.1

tgccccaaacattttttattcgattaactatttgtcacatacatacactacagtctgctctaaacattaccaccaagacaccatacatcaagttcacaaaaggacatgtaaaagatgcaagtatgagacatcagttgtacaaaatttagaacagtaaaactgagcctaaagggagctctctctcgctcccagtcattttggccgtcagaatacacatttagaccgtttctgaccttttgatcatgtttctgcgccaaaaaaaaaaagaaatctaaaacatctagttgacgaagatcattggcttcatggttccgtgtgagtgctctcctttggtttgtttcctacaaaagttaaacaattctcaattataatacactaatagaagctagactacagtagcaaaattccaacaaagagatcatcacattctcaaaaatataaatagtaataaatacagagaattcaatagccatgtgttaacactaacattgatcctcggctgagatcaccccagccggcccaccatctccataaggagttgataggtcaacaaaggagtttgtttcttctcattaaaaataacatgcttatctagctatttaatttgatttcatggactacacttgtgcagacttattatacaaaattgtagatcttactaggataacttaagtaactaggcttaactatgatgtactttccatagcagatgtgtgctaagcatagagttttttatactattcttcctgaaagcggattgacaagctggtgaatacatcccttattttgactttaaatttcaaaaaataaacaagatgggtcaagttaccttcacatgccagctcgggaaagatgtttttccgtctagctatgctcaagaacgcacctggtccactatggtagctacatactgtagtgatgagattccttgtcccaacattgatggatgccctcacccagatataagaaatcttgctccactaccagcaacagaaaaaagggtttcttcagatcctcacaaccgaggtatacattcatcaagatctggaattctttcagtttctcctcgagataaattattttaatattcacttcactaatagaacagtactatatgaaaagtttttcttttgtacaaatccaatatgaatcccaccatctgctaatagtatatgttgttggaaataaagatggtcagactacattaataacacaagtccatgaaatgttccagatttaggcagcaactactttgtttatcaaggaaatcgacaacataacaaaatatcatttttatctgtgataatcataaataagcatccagtcaggtcaaataacagatatagtatccaagacagaagatgtgaacacgagcgattaaaggctcacaaaatagtgaaagcgacacaaacaagtttgaatattgatatactcagccaaatatagtttagtgctgaaatattaataagtaattgctgaagggaaactcaaaataaaatgtgaatgttgcgtgcatagctttgcactgttaaggtcatgaaaccagcccacttacaagcatggcatcaactattacatcacagggcaattgaattgctgcacatacatgaaccattactctacaggaaactatctcataatttcttgctctctagtttgagagtaatttggtttagttcatctttacaacagttttaagttattaggaacaccatgacatctaaataattccaatagcatttatgtcaaccatgcattatgttccaatatagtatacacactaccacatcacaacttttccattaattttggatggttaaggtcataacagtagctttatgtactgcccactcatacaagcatgacaactttatatgcatagctttgcactgttaaggtcatgacaacaacccagttattcaagcatgacattagttattccttcacaagtcaactgaatagttgcatataaaagatctattatactctacaagaaaagtgctcataattatttcatcaatagtgattgcttgcaagaatgtgccagttggagaagaggatattaacagtaggcgaagcatcatggaaaaaggacatcataagtgtcaaatgtcaacatatccctattgttgttctacaagataatatcgcgatatgactaatagtttcttctagagaatggttgaattaagttcattccatatgtaacacatcttttacttaatctgctaacacactccgttgaaattggtaaactatatcatttaaagttccactggtagatcataaaccatcatattgataacacttacctatctccatctgtccaacttttattcaatacaagctgaactcagctgaaaaataaagtcaaaatcaaagcatttatatgcctcgcaatagataacaggaacaatgtagtttttacattcaaaatctcaaccaaacagatctgagcaatactttaaaaatgtcaaaatcaaggcctagaaacacctcacaatggattaggaacaatttagttgttaaaggaactattgctacgcaagtacaattttgtcaaacatgaacaccaccttccagtgtgtgcatatgtgtgtgtatttgatgattgactagccaagtttacatgcatatatctgaatattaatgaccttatttctgatagcatagaattctgaatgagtgttttgtttcacgcttctcttgaataagagaaatggagacatgataatgttaggatcgaggaggtatgcacatgaattaattttcaaatagatatcagggctttaaatggaacatcttgaagctttccttccccactagaaaaactgagcgagatttatctcaataatgtttcagttataataaccaagagaaactatatgtgcatacaagtgttttagaagctacaatatagtagtggcaagttaggatataggttccattcgtttcatccatccttatacagtgtaatttctacggattgatagtagtgaccacaatgcacatgcaatacaatggaaaacactaatacaagtattgttcactctgttcgattggtaagtttaccttctattataatacaagcagtctccagtatatgattttgttcaaatgtccagcgtcgaaacctaaacagtaagaaaccagcatgtaacctgttagtcataaattctgcaaggtgtaaaattgataatataggttccaacagaaacaacaaaattctagaatgatatatcaacaagtcaattagagttaaaaaatagcacaaactggagtgaaatgtccatatcatgtagttcacaacagaaaacaacaaaacaagttatttatcaacttatcttagcaaagaaataagtaatgactaatgacatgtatattgtgtaggggcagtgctgatatgctgcactgtacccata

>TaeST2.27022.1

ttctctctctctcctctctctctctctctctctctctctctctctctctctctcgcctctctctctctctctgcacgcgccgacgccatggacaaacctccggcggcagcagcggccaacaaggttggcaatcctccggcggctgtggtgttcttgtttcacagctgaaggtggtggtgatagctcctgcagcgactgtggtcgtcaatccaccagcgaacaacgtggccgtcaatccaccagcagaggcggcgtcagcgtgtggcgctgcggaagttctgctgctgcttgttcccttctccattttggtgatcggcctgcctctgctgctgaactggtacctccaaaactagagaaactatgtcacgtctggagtaagactgcaggagtgcgccacacccgatgaggacgacggtaaataaggtggcgcgagcgcaagtgtgtgttaagttatcgtgtgtggcctgcgtgccacctgtgtggggcttataagctgtgtgctgtactagaatcaagtctgaagataagagtaaactgaactctactccta

>TaeST2.27033.1

gcagcgcatactcttctctcaccactctctctctctctctctctctctctctctctctctctctctctctctctctctctctctctctctctctctgcaagcaaagcgcggccgcggacgccatggacaaccctcaggctgcggcggcggccaacaacgtggtcaatcctccggcggcgggggctgcgcaggccaatcctccggcggtggcggcggcaccagccaacaacgtggccaatcctcaggcggtggcagctgcggcagtgtggatcaacgtggccaaccccccggcggcaccagatgtgggagctctgctgctgatagttgggctcgtcttgttggcgatggtgttgctgcttaggctggtgctggcgtgactctgagtagtacgacctcatcgtgtgcgcggctaaggagtaccactactacttattacgtataaaactataaattataacctgcatatatatagagagtttgttgttcgtaccatctatgaacttcattgcgaaccaagtttctctgggcctggaacagcattcgacatgcttttcctcgtctgaatgatcgactaaataaagcggcaagtttgtaacccctgcttctacttcaggcttggagaataactcagcatggttttacagtttggattgtcgacaagataaaccgacttgtactcatgtgcgcatgaattgtttctgaagcaaatctacagcttggtactatatatcaactatgacctatattgcattttatccacggatgtgtaaatgtagcaaattttgctgaaaaggcctagtgaatatatataacatttctttgagcgaa

>TaeST2.27033.2

gcagcgcatactcttctctcaccactctctctctctctctctctctctctctctctctctctctctctctctctctctctctctctctctctctctgcaagcaaagcgcggccgcggacgccatggacaaccctcaggctgcggcggcggccaacaacgtggtcaatcctccggcggcgggggctgcgcaggccaatcctccggcggtggcggcggcaccagccaacaacgtggccaatcctcaggcggtggcagctgcggcagtgtggatcaacgtggccaaccccccggcggcaccagatgtggtaatttcccacagatttgcagctttaccagtatccccgccccgatgaattgacttgagatggaacctgtggtcgcatgctcaactgactgctgcagggagctctgctgctgatagttgggctcgtcttgttggcgatggtgttgctgcttaggctggtgctggcgtgactctgagtagtacgacctcatcgtgtgcgcggctaaggagtaccactactacttattacgtataaaactataaattataacctgcatatatatagagagtttgttgttcgtaccatctatgaacttcattgcgaaccaagtttctctgggcctggaacagcattcgacatgcttttcctcgtctgaatgatcgactaaataaagcggcaagtttgtaacccctgcttctacttcaggcttggagaataactcagcatggttttacagtttggattgtcgacaagataaaccgacttgtactcatgtgcgcatgaattgtttctgaagcaaatctacagcttggtactatatatcaactatgacctatattgcattttatccacggatgtgtaaatgtagcaaattttgctgaaaaggcctagtgaatatatataacatttctttgagcgaa

>TaeST2.27167.1

cccgtctctggtcctagcaaacgaagcccaaaactatctacccatcctcgtggtttatcaagaaaacatcacataccatggacgccaggcatgccaccctgtgtttcctcctagttctcgtgttgcacggaaatcctactgcagaggccgaggattgcagatacaggaacgataagctgccgctatgccaggacgttttgtgcaagacacggtgctggctggagggggcggtggtgaacgccgaagtaaaagagtccagatgcgtgggatcagggccaggctccacctgctactgcctcttctgcaaaaaagattgaaccttctgaaggttccagccgagcgtcaaggccatccttagagacgaaatcctaccgagttgaattgaagacgccatgcatggatgctgttaaaaatcttgtactagccagtagtgttatccttttgtgggaaatcagtggatccttttatatctatttttgtggggtaggaaatcaaacagtgaatcaaaaaaggccggccccttcggatttctatccttcccttgcgtaccatgagtttatgtatatattcaattaact

>TaeST2.27167.3

ttctctggtcctagcaaacgaaacccaaaactatctacccatcctcaaggtttatcaagaaacaatacataccatggacgccaggcatgccaccctgtgtttcctcctagttctcgtgttgcacggaaatcctactgcagtggccgaggattgcagatacaggaacgataagctgccgctatgccaggatgtgttgtgcaagacgcaatgctggctggagggggcggtggtgaacgccgaagtaaaacagtcgaggtgcgtgggatcggggccaggctccacctgctactgcctcttctgcaaaaaagattgaaccttcttaaggttcctgcctagcgtcaaggccatccttagagacaaatcctaccgagttgaattgaagacgccatgcatggatgctgttaaaaatcttatactagccagtagtgttatccttttgtgggaaacagtggatcttttttatctatttttgtgggggaaggggatcaaacagtgaatcaaaaaaggccggccccttctgttgcacatatgaaccatctgcgacaatagtgtgtaccataatctcc

>TaeST2.27443.1

agccacacggtttatacataaaccaagcaccgaggtgagcagcccggtttatacataaaccgagagccgcgatgcgccgcccagtttatatataaaccgagggctgtcgtgactcgtgagctgcccggtttatacataaacctaaaccgagggcagttgtgggccaccagcggtatatgtacacgtatgtatatccctctgtaaacattgaaagtgacaagaataaacgaaaataagcaagcccgattcgaacttgtggcaatacaccggttttgtgtgtgcgcatgtgtacaataaacgaaaagaaccagattcaggagctgtgtacgtgtgttccttaaggcaagaatcaattatcttgatgcttgtactgcgcaaggatacttgaaagaaaaaagcgtcggcacgaccgtcgccgtggtggactgctgctcctgctccgctgctgcttgccgccgctgctcctccactgctgctgctccctacttctgccacttgctgctcgctgctgctactccgccgctgctgctcgctgctgctgctgctcctccgccgctgcttgccgccgcagttactcccaactgctgctacttgctgctccgctgctactactcacgaggaaggaagagaggaagaggtggagaggaggacgagaaaagaagggacgaggaccagcttggccgagaggaggagcagtggtggcgaggcgagccagaggaggagtgtcgacggcgaggctagcaagaggagtagcggcggcgaggcaagcgagaggagg

>TaeST2.27642.1

atacaaccttatagccactagacccgatattaagcccaaagctagtgttctgtacttcctctctttggccgatcatggctttcaagatcaccgtcctggtattggtattggcgctgctccttacgtctttcgatgttggtgcaagcagggaattgcattgggaggcgcccccagccagagttcgggcctgcggcagccaagtctgtgccgagacatgcgtcgataatggctattgtggcgggatttgtgacgaatctatctgcaagtgcgtcctctgcaaatcactccacgccggcactgaaaacagagaatcctaatctaaggtggatcaataaacaaataatcgatcctctctttttcttattgtccgagacaatattgttctttgtctctttttagcagattattattcgaggagatctgatgtttttattgtcgtgagattacattggctcggtggattaatcatgatcgttgattgtc

>TaeST2.27681.1

gtagtatttgccaaaggttggaggtcaacggtcgtcacaagcctacaagagaatatattgacagagagtgtaatggtgtaaaaccacccaataattttggcgaagttgatccctggaccgcgtgggcgtacagaccatggaccatctcgttgttactgatgggaacctgctttttaatgtgagctgctgctggatctcttttacctgtggagttatttttagtttttctacagagattcttacaacaatgatgttagtaatgcaccgctaatggttgacctactatggcattttacaa

>TaeST2.27759.1

tccgatgtgagcagcggcggccaaggctcgaaaaaaaatccccaatcctaaaggcaagcccatctccctctagcttgccaatgacgacgggggtgtagatcctgtacctagatcggcgaccttggccactgtaggcaccgttccatctccgccccaacgaatctaggtcgtctcgccggcggcggtcgccggtcgggctgcccgggcggacctcttctgtgctgcgctgcgacactgggcgagcaaatctccaggtagcccgcctacccccatgcctagctcggacatctgctgatggcaaagaacagacctcgggtcttcaggaatgcaggacctctggagacccctgccacatgtgatcgaatgtagatgaaaagagcgtaaaaactaatagaatggagtaccatttctgggaataaatgatcattcaatggcacataaaacttgatgagcaagaagcacgagcaccggaggcaagcgctacaacttttctattctctgcattcaagatagcacaacactagaaatgtccatgccatgttatgtattgcatgtctcttgtgtatcttctgctgctccagattgataatgtactcgacattagtgagtgagatggtccagactctagagtgacatgtactcaaatatttgtgcctgagatggtttcacatgtactcagatctttatgcctgggatggtctaattaactagacgtgttctcaactatttatgtgtgggatggtccagtttcacatctactcagatcttcatgcctgggatggtctaacttgacgtgtgctca

>TaeST2.27796.1

actgcatgaagtgacaatctcaccaacatcaattatatcgaagctggataaataagcatataaatgatgtctgatcatactacagaggaaaggtggaatactactgcatcaaagacgggcacaattcaggattcacgcatacggcactcggtcaattgtggcagctagcataatgcatactcctactcattatttatcagtccctcattagtacctcaccagctgatccatggacatgatcgctgcgacgctgcctacgtacccctgcttctcagtcctactcctcaccgctgccgaggaggagggccaagaagctgatgatgtctcccgctgcgtagatgaagaggcccccggtggagcagatccggaagaa

>TaeST2.28013.1

ccgccgccgccgccgcctcctccacccgctcctccgcctgaaccctagccatggcgtggcgcggcgctgcgtcccgcaccgtcctcgccgcggtccgccgcccggcaccctccgccgcgctcggcggcctccgcgcccctcctcccttcgcggccccgcgccgccggatcccttcccccttcgccccctcccactccacctccccgctcggggccgcacggcccctggcggccatgatggggtcgccgctgacggcggcggtggtgctagggcggatgacggcgcacccgtctgccagcgcccgggcctgctgcgagctctcccagggtacttgaatttgccatgttgattcaccatcctttttaaagaggatacaaatcttggattcgtcttttggaaagctattctggggccgtccttttaatccttgagttattagaagcatgtctccaacaaaatagcttctctccaaattactagcctagactgaaaataatcttcctgtcatgtgacatggtttatgtcatggatttagcttctacttttgtagcgaaatacctttgtgttgcctgctaagaggatttggtggcacattttatatgatttcttagattggaaggtatctctgttccacgagcaacaactgtggtcacactgtcattttagtttccagttgagcaatgatgcttgagatgtgtgtgtctgatttccaat

>TaeST2.28013.3

ccgccgccgccgccgcctcctccacccgctcctccgcctgaaccctagccatggcgtggcgcggcgctgcgtcccgcaccgtcctcgccgcggtccgccgcccggcaccctccgccgcgctcggcggcctccgcgcccctcctcccttcgcggccccgcgccgccggatcccttcccccttcgccccctcccactccacctccccgctcggggccgcacggcccctggcggccatgatggggtcgccgctgacggcggcggtggtgctagggcggatgacggcgcacccgtctgccagcgcccgggcctgctgcgagctctcccaggggaatggaaaagatgggtgatgcgggagaggcaagggtagagcatagctaaggtacttgaatttgccatgttgattcaccatcctttttaaagaggatacaaatcttggattcgtcttttggaaagctattctggggccgtccttttaatccttgagttattagaagcatgtctccaacaaaatagcttctctccaaattactagcctagactgaaaataatcttcctgtcatgtgacatggtttatgtcatggatttagcttctacttttgtagcgaaatacctttgtgttgcctgctaagaggatttggtggcacattttatatgatttcttagattggaaggtatctctgttccacgagcaacaactgtggtcacactgtcattttagtttccagttgagcaatgatgcttgagatgtgtgtgtctgatttccaat

>TaeST2.28108.1

aagaaaaaaaccctaaccagatagaactcgtcggagggagcgggaggagatccaaagggcggacatggcggcggcgaaggcggcgttggagaagatgagggcgttctgggactcgcagtgcaacaacgaggagaactgggcggtcaactataaggtgctaaaggctgctggtatatttgctggatccgtcttcctgatgcacaactttggccataatatggtcatctaaacgggactagatagggcaataatggacattcaatggtgtggtgttgcactgcctttttcgttttctcatcaacatggtcatcacacatgttcttagtgtggattaaggagtgaaactgttgttcttagacattggttggtaataatggcagccaaaatagtactttatatgtattactctatcaggtgttctcatgctttgtgaattatcaggcaagtcgtcgcaaacttgttggg

>TaeST2.28161.1

atcgaagagtgggtctctctctcgcggcggcgcaggcgtctctctccttctcctccggcggcggcaaggcattttggcatctgagcccaacagctagcagatggcaggcggcagagttgcacatgcgaccctcaaggggccgagcgtggtgaaggagatcttcattggactcacccttgggctggttgctggtggtatgtggaagatgcatcactggaacgagcagaggaagacgcgatccttctacgacatgcttgagaaggggcagatcagcgtcgttgtcgaggagtagttctgaagtgctgaaccagaagttctacatctcgaagtcttttgtcattttggttttgccagtaatttctgaaactctctggagagcatgacagagtgattgctgtggagattgtcatgaaaataagcacaccaccatttgagaacatcgaaccattttgaatttgctttttgtcgtatcaaatatgtcttgtctcatatgtgttgcaaaccaattcagcgattggaaggcttccctctgacat

>TaeST2.28231.1

acaagatcttttaaagggttggaatcaagttggagaaaatgagcatggataaatttgttaaatagaactcctcaaaaaccaacaacaatctcccccacttttattaccaaggtgtgacagtacaacaacgtaactgaaaggagctaacaaatttctgaaacttatcaagagacatgggaagcaacagaatcatgccagccaacacagatccagtacgctcatgtcaatgatacactacaggttgccgggacagggaggagatcaaacccagaggtgggccgttagggaatggcgccctggcagcactcggggtggttaaaacaacaaacgggagtgagacaacagccgggcctcaactccgggttcacaggcctgccctgcaccttcaccgacgcagcaatcgtcaggagcaggcagacctgcacgaggagggaaactaggaaggcacggaggtgacccatcgccatcgtctcgtctattcttggcagcagagatgatacgggagagaaagagagagaacaatctttctacttctggacggatatttt

>TaeST2.28307.1

tcatcgtcccatccaacccctgagcaccaccagcgtagcgggaaggcatccatccatggtcgtctccaccttcagcggccctgggattggggtcggcttcggcgtcggctgcggattcggagtcggatgggggttcggaggaatgcctctgaacgtattcggcttgggcatcggtgggggatgcggagttggtcttggtttaggatggggctttggaaatgcttatggttgtcaatatcgatcttcaagggtccagtttcagggcattgagtttcagaaaaagtctgaaggagatgaagcaccaaagcttgttgaaaagtctcgtccttatggctagtatagctgcgatcatacaattggcacaggcaatgttaccattgtccgcttgattgtagtcaattgtctacctggaaagttgtttcaaagcattttgatgttttgatgaacttggacgttttcatagtaacataacgaggataatccatggtactttttccgtttcggataatggtctttatatttggctaaaattgtccattaacattctcttggttgggtgcaccctgcaagtttttattgttactcttgttaagtacctgca

>TaeST2.28421.1

gtcagccgttcccgtgtagggattagccacccactacgcgcccgccccgtgctcgacgtgcattcctcagcccccgcgccccctccgctctctcctccccctcctcgtccctgtcccaaccctaatccttttccctctccaccccgcagccgccccctagctcctcgtccccttcactcagtccatccctcctcatcccagatccagcggatccgaggcgtgcagacacggaggcgagtttctctggcgccggtggcaacctgcaagcaaagggcggcggcggcgctcgatttgatctgcaggagagagagagatggagaggagggacgccaccatgggagaacagggccaacttcgtggtgggcatggaggcgcagaggaggatggcgatgggggatgggagcagagtagcaggacttccaggcggtgctcgcgtcatggtggagtggaggagctcgtcgtccatcatcacaatccacaatctagatctgagatagaaaagaagagaaagaatcttgtggaatttcagatctgagataggaacggggagaaagaatcttgtgaaagagcctgacgaaaaagaaaggggtgaatcaccactttctcccattcatgtttgtttctgcattttatttttaagatgcaggttggccaactgtaacaatagaaatatatgtgcaatttttgtttgtgtacttcaataaaaatatattttcacttgacctcgagggcggttgcatccagttattgtttccgggagatttattggtgaaggtctaaa

>TaeST2.28770.1

tccccttcctccccctctcgaaccccacctccctctccaagactaactccctttcccaattcctcccaaagaccttgtatccagatccatggtggaatagatcgagaagaggagcgtaacactcggccactctctcttcactcctcagatcccaggcctgaacaatgaaaaactgaggtagctcgtggccttctcaaagcatctccgcccttgccatgctgtcaccatcaatgggtctaatgactaaaggagatgtacatgttgcgccttttcggccacccactgttgtcattcgatcccaggggcgatgccctgatctagatgccactagctccagtgctagtggacgctagaactcttcagagacggttctccagggtccccggcatgaccatcgtgtttctttcagcatcattgctgcttagcgaggaagtctagagggggagatgcttgattggaaagggttggagtattagagtgaaatgtggccccagtgcccacaacacttcatttcgttatatcattatggtattattgctttggtcactgcataagggatattgtgatttattgtatcacattcttaattttatatatatccagtcattattatggtttatatttaatgattccgcatgg

>TaeST2.28775.1

tgtcagggacaaatatatatcacagcacaaacatatatattttcacatgagcatagttgacgtaggaactacagtacacacacagccacacatatgggcacaaggtactacaccaaggttcaggttacacgagtgataacacaaacgaaattacacaagccactaaactagtactcgcagatcaacagaatattactaaaccactttaatactggacagttcaccaattcttacaaggcatcgtacaatatttttgcaagaaaaagagagagagaagcagccgtttgggattcagagaagcacaggatcagcaacaagtaaagaacagcatcaagttgttttcttcagtgaaggaacactagcaaggaaagggaataggatcagcgtgaggataggagaagaagcagcagcaggaggttgagagagcagcagcaggggcgaagttcacaggtgggggagcagcagcgaggcaagagaaggagcagcagaggtcgtgggagaagaagagcagcagcaaaggagcagttcacaggtggaggagcagcagcgaggcaagagaagaaggagcagcagaggtcgggcgcaagcaggataggagaagaagaacagcagcagtcgtgtaagaaagaggagcagcagtttggggatttggagaaggaagagcagcagcgaggctagggaagaaggagcagcagcagtcgggcacaagcagaggagaggactcgcgcgacggatcagacggccgtctggtgggtcgcgcgagcgtgaggtggaggcgcggtggtgatggggtggcgctggtggctgatggtggaagatggatggcgctggtgggtgatggtgagatggtggaggtactaggcggcgcggggagatggtggaggtactggttggcgcgccgccacgatggcctggccgcggcggcggccggaggtgggagaggaagtggtaagtgggagagagatgga

>TaeST2.29058.1

agcgcagctctgcactttggcccgtctccaatctctcttccaccttcgccgttctcctcgagccgccgtctgccccccgtctctctcccccgcgcctccgccatggcccagcacggcttcctctcctccctccgctcccacctccggtccccgccgccgccgtcccacacccagccccgccgcgggtaccacgtcgagctcggcgcacgcgagaaatcgcttttagaggaagatgttgctttgaagaggttcaaatcatacaagaacagtgtgaaacgggtctcgaaaatcgggaatgttctcactctcgttgtctttgctgcctgcagctatgaggtcgttgcactggcaacaagcacccaatgaaggaaagtgcaatcggtgtgagcctcttagatcttccagaaagtgattcatctcataaactggcattatatcagtactcgttttgagcgtcctgtcgatcttatatatcatgtaaaataaaaacaaactaggctggccttatttgcgagtcactttctagtctctgcccatttgttatttgtacacaaatcccgaggagtcgatctgctattggaggatgtttcgataaattgttgacctcg

>TaeST2.29242.1

cctcgagctctcccgttcctatcttcttctcttctcgtgtagggttactaatcctgctagggttcgtcggagctctgctgttttttctcctccctcggctatgttgctcgtttctgctctctgctctattttcatgctatttgtgttgtaggttttgaaggtattgatgttttgaagctgtttgtgtatgcaggatcagtaactgcggattttcttggatcaaggatttactttggcgaatctgctccttttttgtatggtttgccctgtttgttgctgtcctggtgctccatgcttttcgtttgtgccagctgatgaagtccttgaagtttttgatgttgtccttgatgattttttgctttacgaggatggtggtttcagaagatgcagcatctaaaataaaattcagacaaaatatactggagatggggcagcaaataatgatatacctttgcctgatcagaagcaatttgatctccagaggaactaaactttgggaaatcgaaggagaagtatattgccagcaataatcctgtggagaagttacccagaaatatcagaagacaggaggagatctgagctttgtttctgcttccatggtggcggctgtgtggatgtcgaggaacaaagcagagggggcaaggaggaagacctagtgagctgtacgtttattggatctttagtttagcgggttttcaggccctttttctttctgggctagacagatagcagaagaaagaaacagaaactactgagcctttttgcattattgggttgtcgactgatcccagttttgggtcagtcagttttctattggattggtttgtttgtacttttgcaacaatcgactgacccctatcttgtgtcggtcgattgacctctagccaatccctttccatcct

>TaeST2.29440.1

tttttgtgtttcttcttcttattttgcttttggtgtgttgttttttttttttttttttttttttttttttttttttttttttttttttttttatgtgcattttcattttcattttcgccttttctatattttttcttttttctttttcatgttcttttttcttattttctaatttgattttatttcttttttacttctcttatttttattt

>TaeST2.29781.1

gcaatcttgtctcgaaaaaggaaaagattaggcctcctgcaatctggagttgtgaaggcggcgggatgatctaccggagctggtcgctgctctcgtcgacggtggcgatctggggcggtgccgccgccgcagggctcgccgtcgtctccctctctggcggcaaggaaaagtttcaggactacctgtgccgcgaaggcgataggctgaggcggcaggacagggccaccatggccagcgccaactgatggaccatgaaagaaagcgccgcggagacgaagaaacgagcatttcatcatatatccgcatccgtgaaggcgtcgtcttggtccaataatccttgtttgccgtgctttaaacatggtgtggtctggtgtcctcatcgaatccaatagtctttgctcgacatgatgtggaacttggtacggcctgatgagcttcgctttccggcttgccaagtggctcgaaacgttgtcttcgcaacaatttttttgtccttgtcagcgaacaatctttgtcccattttggatttgatcactgtttcggatatgtgtgaataataaacaccgtgggatccagttgaaatcc

>TaeST2.29798.1

tataacaaagtgcaacttaccttttaaccagattaaatcaaggaccaacaacaaacaataaaagatcaagaataacttatatgcaatgaaggcatgagagataagaaatctctacatgagacggctataaacactggagagcaacacctataaatacaaataaaaagggttcagggttcattgcatagtgggctcagattcgcctttcctcgatcatataacctacccccgtcgtcttttgtcaccaaatcaatttgtttatatagtggagctgccctggttatcactcaacaagaatgaccatccaaggacataacaataagtatcatagaaccaaaaaggattctagagacaagatggaggatccaacttgatactctcaggtgccttgattgctcaaaggcacacttatttcagccacggtcaatcatcatcgccctagcaagaatggcttccaataggccggctgtccgtggacacgtcagcacatgtacgtggacatttgcggatagaactttcggggtcagcattggagaagccctaataatgcaatcatgcattttttttccttcagtgtgttcttaaattcctccaaaccaaagaggcgctaaatacaaatacagaatggcaacaacatagcacactgtgcttgagaccagaactgcattggaatgggctcagattcgcctttgctcgatcatgacccttcccctagtgtctccatcaccagatcaatctgttcttacaagggtgaaactgccctagttatcactcatcagaaatgaccatccaaggacagagccactaaaataagcacatgattgaaacggcacaaagaaacaaggggggatccaacttaatacccacagtgcctcgattgctcgagagcatgcttatatcagccaaaaccgatcaccatagccccgcgccagctaaga

>TaeST2.29940.1

tttggtttccaaatccaccaaatatatatgcaatgagtacatgccaattgtctggaattgaggaactgtggaccggcagaagctttgtgccatactaccattattgcaggtttgcattaatcatatgagcatatatactggcctacaggaccatgtgaggaactatattttgggttcactgatactaatctgataacttaaattttatcaa

>TaeST2.29945.1

tggatttcatacagtaaaaccaaaagccattcaaagaacatgtgacaacatggagtaacaagaattagattgaatggtatcacagctccaataaacacagcatatcaagaccgaaatgttaagactacttgcacaacgcaggaaacaacattagaagttcaatgttctaccctatgagacaccaaaccaccaagacacagaagctacataggtcggtgtgtaccagacccgctcaatcaacatctagacagcagataaactaacggactgaaacactgacagcaggaaggcagatcatttcttcttcttggccggtggctggtcctcatcttcctcgtcctcctcatcctcatcctcgtcatcatcatcgtcatcctcgccgtcttcaccatcatcatcgtcgtcgtcaccatcatcatcatcgctccccgcgccagcatcatcgccgttcgcctcagggtcatcatcctcgtcatcgtcctcgccttcttcacccgagaagtcctctccagcatcggcatcatcatcatcagcatcaccagtttcatcatcctcatcttcatcatcgtcatcatcgtcatcagaccctgcatccttgttctcaggaacagtctcggatttgattctgccaaattgc

>TaeST2.30341.1

tttgcatacaaaccaagcaacggttgattgattatcacaaactaatattcatatagtaattcattacatcgatttgcgcatctcatagagacgcaactgtgatcacaaatcatcacaccggatcaaccacccttcacagtaagcatcacacaagaaacgagcaacaaattactccatgtacacggtataaccaaacccagtaaacaaacaacaccaacgctccagctaaggaggaggagggagctcgaccgatcagacggcgtcggggccttccttcttcagcggtgccgggtacggccccggggactgcccggcgacgccgggcggcggcaggggcgtgccgtagatctgggccccgacgccggcgcgcatctcctcgcggcccttgtgcttgccgaggaagtagcagccgaagccgagcaggatggtgaagaatatgaagggcagcgagatcaccagcacgaaccccattgccctccctccctcccggatggacgccttcaatccaaggctggcttggatgcttgagcttgactgattat

>TaeST2.30372.1

caccgcaaaaagataaaataactgacacaacaaatgcagggccaaaaggcacaattcggagtacctcaaggataataggtgcataaaattcagtcacaaacacatataatcctcacattacataaaactcagtcttggaacaaaaccagactagcattaaacagctaaattggacagaacacaggtaagacagcttccacagccatagcagcaaacacgccaggaatcactaga

>TaeST2.30639.1

gagatacacgcccctaacattgcaatacaaggtcagatcgagaaaaaaaactttgtaatacaagatgagagctacacaagttctactcctagtccttgtcctcttcgtcctatccccagatctggcaaaggctaagctgcactgcacggatttcatcatagtcaaccagtgcgacgtggacgagatcacatgccaacattactgctacaagcagttcaatggaaccggcaagtgcatccccaacaaaggatgccagtgtcatttctgctatgcaactccaccccagccatcccctgctagcagctcctagtcgtagtttgtctgtaattttctgtttcttcaacagttatgaacatgattcgtttctagtgcaacaaccatggcgccataattaattacatgtactgtggcctatactgcgaagttatatgttaatgcaatgcctgttttgctttatatataaatgattgttatagtctaggagcgagaatacatgccataaaaatcatgtttattataaatgttttggtttatgtaaatgtttgttatata

>TaeST2.30658.1

acctctctctatatagaccacaccaagtaccattagtctcggcgaaaacacaaccaaagagcaacaaaaactattgcaacatctacatggagaagtgcacgaaacgcctccatgcattagcgctccttcttctcgtatgcttcgctactcatgcacaaggcagtggcacggagtccggtgacgccgaggcttttgcgtgctacaagcttaatgttttcccatcctgcaatccaaagacaagtcgatgctactgttgtacaatggatcagtgcaaaacccgctatggcacaatggatgagtgccaagctcactgtgtagcttcgcctttgggtgtaaatgagccagcggcaccctcctcctcctcctcctatgtacttccatgaaagtgaatggagtattatgtttgtttctttgagaaaaaggttggagcccagctt

>TaeST2.31389.1

tttggcatgtcaaaaagattctattgctgttgtgaatataacatgggttgtgacttgtgatacgcatggcattttgtaatttttcaacaatggacatataactatacatatgcgaagatcatcttgaagcactcttttgagttttgatgaggctgctaaccgttattcgagtactgactatatcgggagcggctaggcgacagttgactgaaatccgagaaagggtcagtcgattctcctcctctttcaaccgttagaccaagatccaaccgacactgttcatcttcaaccttccatctcttcttcctcttgctcacctgcccattcagcccagccctaaccgctggcccccgcctcgccgccccgc

>TaeST2.31495.1

tcctctgctgactttgtccgttttcttccccatttccagcggcgcgagcttgaggcattgattcatggcgatttttggttaagagatctcagtcgctgatctctcgagggcgagccggtggctaggcatccggtcctcctcccccctcccccctcccgtcccctgcttcattgtcctcaaacgcgcgcagagagagaggcgaaatcgatgccatggtgatccggctgcgctgtcttctctgcttcgcttgacttcgcgccactcctcatcttcctcgtgtggatctgcgaaccctgtgtacccctcctcatcctccatccatggttggttttgatttgttaatgctataagagagacatgcattgaggaacttagcttcatcatgtttgagacaggcaggggggagagatctgcctaaatcatacttagtatgtggtgattgcaaaattggggttgtttacctgtgcttagtcatcttcagcagagtggatcgtcttcgggtttctgtcccagatcatggaggaatcgccctcttggttggcacttattcccgtttcctctagctattctcccccctccatgttcttcccctctacagtgcatttttgtagtggatattacactgaaaataatagtggagttattattgtaattttatagcttttcaatgtaatttttagtgctccagagcttaatttgtcatggcattttaggtgtgtgtgcttagatgacatcatcctgatcctttccttttcttttcgtattgctttagcttagtgcacatgtacatggtaaagcttgcctgtaactttgggtttttttgccctcctttttgttgcttctctgtccgagcgggaaaagttgggttgtggacccgtta

>TaeST2.31558.1

acaacaccacctagatccagattagaaagaagctccagcatctcctcatggcaatcctcggcaagagtactagtgttgtgtgcctcatagccctagtgctcatggccaccgttctctcctcacgtgccttctaccatggatgcgacatcggtccatttcccggggaaaaatgtcccgagctatgtgaaaaagcatgcggtaagcccggcgcttgctcatcagacggtcccgagtcttactgttgctgtgaaccagtacgccaagatggatccatgaggcctcaggaggttactgaaatgatacaaacatagtgtcatggatcaccagaaccatgtcgaataaatgtgctaggtcttgttgaacttgtattgaatttgatagtatcgtgtgttcctcggttggatcgaatgaacttgtatatattccccatttaataaactttgtttttgttggcccca

>TaeST2.31610.1

tatgctgatcggatgtgttactccaatataaatatatatcatatgtttatacaagattcaaaacagatacgaataatagcgacatatctttccctactaataaagcagcgattgcttctggtggtacgtcgtcggcgattttgcaaaaaagtggctaatctaaaaaacgatttgttcttaaaaaaggaccctgcagtttatattattcaacccacgaccgcaggaaagggaagggccgatgcttggttggtggtctgggcggcggatccggcggtggggcagacggatcatgtcggagtagcgcgaggctgacggagaggtggcggccggaggcagggcgtgctccaaggcggcccaggcggcggatcaatgggcgctcctctcggtcgtcctcctctcgaccgtaggacaggcaagaggcggctcttggttgtggtctgggcagcggatccggctgctccggcgcggatacggcgatggggcggacggatcgaggcggaggagcacgaggccgacggagaggtggccggccggaggcgggccgagctccaaggcggcggggttgttccatggtgacttccctatagaaaacaaagattagaaacagagagaagaagaaggtaaaagtcgagagagagaaggagagagggggaagtgacagctcctgggttggcctgctcgtggcacagctggagctcctctccaggcgcggtcggaggcagggaggccgggctactggagtggcaaaaaggggggatttgcgaagggggggatagaaacgagtgcggttgcgtgcttttgctgtaagaagaagaaatatgtttattatcagaataaatagtaccacctcgctcctttgtaatcaatcgaatcaatttatatacggcggttgcttgatgcagcaacaagcagagaagggaacagattaaactcaatggaaggctaaaggaacaacttaagcacacaagcagcggctgctttgaggcatcaacaaccagaggaagggaacagcaggaagaagtacaatccaatcgtcaggaggacgacggccccttgaggaatagaaaatccgtcaatacagtatgaagctagccggcttggatcaccggccacactcctcaaacaaatttaccagtgtacaagttgaaaatgcgtgacacacggtgtcaccggtggctgtcgcctgccgccccttggagagcagcgagccctgcctggcaaagcccgcaccttgcacaggcgagcacgacagtgcctcctccagcgcagcatcgaagcgcacggacggcgggaagcttcgcggcaggacccagcggtggcgaatgaagatgaggacagtgagagggagaggaagaatcgagacgagaggacgacaggataagatttcagcggtggggggaatcaggggataaggcgtgc

>TaeST2.31679.1

ccaccaccatcgcctccaccctctcctccgcctgaaccctagccatggcgtggcgcggcgctgcgtcccgcaccgttcttgccgcggtccgccgcccggcaccctccgccgcggtcggcggcctccgcgcccctcctcccttcgcggccccgcgccgccggatcccttcccccttccccccctcccactccacctccccgctcggggccgcacggcccctggcggccatgatggggtcgccgctgacggcggcggttgtgctagggcggatgacggcgcacccgtcggccagcgcccgggcctgctgcgagctctcccagggtacttgaatttgccatgttgattcaccatcctttttaaagaggatacaaatcttggattcgtcttttggaaagctattctggggccgtccttttaattcttgagttattagaggaatgtctccaacaaaatagcttctctccaaattactagcctagactgaaaataatcttcctgtcatatgacatggtttatgtcatggatttagcttctacttttgtagcgaaatatccttgtgttgcctgctaagaggatttggtggcacattttatatgatttcttagattgaaaggtatctctgttccacgggcaacaactgtggtcacactgtcattttagtttcgagtatagcaatgatgcttgacatgtgtgtctgatttccaa

>TaeST2.31847.1

atctcccgtaacaaatcgaagagtgggtctctctctcgcggcggcgcaggcgtctctctccttctcctccggcggcggcaaggcattttggcatctgagcccaacagctagcagatggcaggcggcagagttgcacatgcgaccctcaaggggccaagcgtggtgaaggagatcttcattggactcacccttgggctggttgctggtggtatgtggaagatgcatcactggaacgagcagaggaagacccgatccttctacgacatgctcgagaaggggcagatcagcgtcgttgtcgaggagtagttctgaagtgctgaatcagaagttctacatctcgaagtcttttgtcattttggttttgccagtaatttctgaaactctctggagagcatgacagagtgattgctgtggagattgtcatgaaaataagcacaccaccatttgagaacattgaaccattttgaatttgctttctgtcgtatcaaatatgtcttgtctcatatgtgttgcaaacaaattcagggattggaaggcttccctctgacatgaatatgatttagatttcactgtcatccttttggaagtgagctcacctttttggcttaatgcaatggtgggatgcctaacaagctactccttgtgtttgatattatcatgaatggaacaagcaccatcattatgacttataactggtgctcttct

>TaeST2.31916.1

atttgttacatagaactcctcaaaaaccaacagcaatctcccccactttattatcaaggtgtgacggtacaacaaagtaactgaaagcagttaacatttctaaaactgtatcaagagagatgagaagcagcagaatcatgccagtcaacacagatccagtacgctcacgtcaatgatacactgcgagttgcggggacagggaggagatcaagcccagaggttggccgtcagggaatggcgctttggcagcacttggggtggttgaaacagcaaacgggagtgagacaacaaccgggtgtcgactccgggttcacaggcctgccttgcaccttcaccgacgcagcaatcgtcaagagcgggaagatctgcacgaggagggaaactaggaaggcatggaggtgacccatcgccatcgcctcgtctattcttggcagcaaagatgacccgggatagagtgaacaatctttctactcctgaagagatatttt

>TaeST2.31953.1

ggggctcgaggatttggagacgccgccgcccgactcctctccaagactccgccgtccagcgcctccccaagacgccgcagctcgacgcctcgacgccggcgtacctcctccggcgtactggatttctctccggtggttctcaggcaaccagagaagcaagattaaacaatttggacaaactattggccattcaaaaactggatgatcatgacatcatcaagagcaattcatatgtatgacaacatcattctcagtggtctgtaaaaatataactaattgtgagctattgcacaagtaccctgtgttgcattatgtttgttgcaaaattatggtgttgctgtgaaagatcacggatgagatattattgattccaagtcatgttgtttttgagaaatcatgtatttcctgtgatataattgatattgttgtgatgagatattgatctaaagtcaggcaaaagttatgttattgctatgaagaattgctgcaaattattctcatgtagcaagttctt

>TaeST2.31965.1

gtcctatccaaccccggagcaccaccggcgtagcgggaaggcatccatccatggccgtctccaccttcagtggccctgggattgggctcggcttcggcgtcggctgcggattcggagtcggatgggggttcggaggaatgcctctgaacgtattcggcttgggcatcggtgggggatgcggagttggtcttgggttaggatggggctttggaaatgcttatggttgtcaatatcgatcttcaagggtccagtttcagggcattgagtttcagaaaaagtctgaaggagatgaagcaccaaagcttgcttcgccagggcttgttgaaaagtctcgtccttatggctagtatagctgcgatcatacatttggcacaggcaatgttaccattgcccccttgattgtagtcaattgtctacctggaaagttgtttgaaagcattttgatgttttgatgaacttggacattttcatagtaacagaatgaggataatccatgtattgttttcgtttcggattacggtcttcatatttggctaatattgtccattaacattctcttggttgggtgcaccc

>TaeST2.31990.1

gccggcccactgctgcaagacctcactacacatcagttcctccccaaatatctagatccccaaatctcaaacccctagcttggcagtgctcccttctcttgtgatgtccaccgcgagttattctgtatccggcattcgttgccataccccagtataccaacttccacttatgttctgccccattgtcgtgagcaagttgtggttctcgtctccgcaagtatcctcatgatggttaggtgttctacaagtgcaaatcggacagacttgcgcataaattctagcattgggagctacagtatgtggagtatctgattcagtaacggtaccttgttggtgcactacagtatgctagagctacaagggaagagcttgagcatgagaaagcagtgaaactagccattgttgcatccagtatcggcagcaatgccgctgacgatcgcagactgttgacgatgatgaatgctttgatactgatagcaagagagatattgttcctcatgaagttaggccttggtctgggtttactggtatgctttctgatgctctttgtaatgttgatgaaatgatatgagcagtcacattagtctttcttgatgtatctttgtttctatgagttggtatgggtttgttgctatgctttatgatgctctttgtaatgcgtatgaccagtaacattagta

>TaeST2.32040.1

tttggatcagagtgcacttttttattacattttttataaatggtgtactgtagacaagagtgcactgcattttcttttgtagtctcttactgcacatggcaagcgagtgaaatgtgtcaaaaataatgcatccagacacaaaaaaaactacacacccaaactgctttccctctaggacacaaatctgtacaattcgctattgaacacatcaaattcacaagtggtcttctaaacaagaatatttcctttcttgttttttgcctccatagtggatcaaagaaggagtgcagaaggcagtagcacctatctgaaaactgcactagagatacatcagcaatgaacttcaccagagcacatcttttgtaggaaaaaaatcaaaccgcacactcagcaccaactgagttgattatgggggcttttttgcaagctgcacccgcgcacacacacgtcggtgtggccgtcagaatgcaagaaactgaacctcatcttttggagtaacactgatgtgagctgcgagaaaactggattaagcactgcaattttcaacacaaaaaagaactaataaagatctgcaccccaacattgctcactcggggaggttcagcagcaacagcacaagcgaactgctcaccaacgacaccggtggcgatgcagttcatcgaggggaggtagatggtcacggctgaagccgtgggactgacgccgacagaaggttggtggaagagggggttcagagaggggggaccaactgaactgcagcactctttctaggtggaggaagtgcactcgtcggggtcgaggaagagcgcaacaacaaaagaaatcacgaatccttggcggttgagctccgagacggcatgggacatgccgctggctcacagatccggcatctctgcaggaggtaggggaacggagaaggctgggccgcgaaggtgcgggcccaagcgggacgacggtgcgggtctcctccccgccttgaaccaccaccacccgtcctcgccccccgaggccaccatcgcatccctccccggcactggaccccgtggatggacggcgcggcggcggcctgtggaggggactaggggcactggcggccaggggagcagcgaagtccggcggccttggaggggcggctataggtgcggcagaggccagatctgacggggcgccatggctgctctcggggacccgagggtggaggctgtggggcggcggccgatgtcgatccagtggccggcacggtggggcgggaggtgagggcggcggttggaggccggggtaggcgtgggagagggaggcagaggaggatccccccgatccggtgttggttgggcctggcgtggccggaggtaggtggatgggcggggagg

>TaeST2.32057.1

tttgattaatatatagagactggaaactgaagtactctgaataacataacatctgcaagtaagaacagaagatcttaatcagttttggcttccaaaatcacataattaaatatgtaagatctagcgaaaaggaaccagcatcatgacttgactgcatgccttgagtactcgctcaaggtgcccctactggtctcgttttgggccaaaagaagaatagaagatgttcaggcggtagcataggctgtcatgctcatcagggccaagtgttgaatgctaatagtgcttcatggcctggctccaccaagtcttaacgcctgcagtggcaaaggtgaataatcctctccgttattgaaaaatatgtgatgaggactcaagtcaatagcatcatgtctgcccgaaaagaagtgtccatcgtatctgccactactttttcctgaagatgccactacctattgtgaagtttaatatcattggaccaaaacctatttcaaattgcaaaaacagaatgccgacataaaccaatgatattcaactactaacaatcacaagtaaaaaaagaggaatgcatgaatatggaaacatctgcgcgattgagtacaatccaaacaaaacctacatatgtatacatagctagccatatggagttgtaaaccaagacagaggccacccaaggcagaacatcggatagttacaaaggaacataaaaaacactaaagatagaaaatagtaaagacgaaggaagcttgtacaaatacacaaaagcccatgacttccgaaacaccgatcaagaagcgtcaaaattagtacactgaccaacctacatgcattgttaaacctgaggtaggcagcatcaatctattcttccaagaacaagagggtaatgtactttaagaaaaaggctagtgtactttaagaaaatcgacaatgggaccatgcagtataaataaatggtatggtcgcaactcttccttctctctagctctaattctgagcacaacacaatgatccacccctgttttaaacagtatactgaaatctgattttagtgggcagcatgtacgtatagacaacctagccactccaacttcaaaagaatcaagttccaactccacaacacatatgaccattacaaaaccacttttcggtaccaccattctggccttaccgtcatgtggcaacctaactgatcctcatttttttagaaaaaacaactaccctaccgacccttcataggcacaactcaattaactcggtggtcagccaagcattttgtgactgctgcaatcttcaagtatataatgtagccaacttattttttgatactaagagatataaaaacaagctagagtagccaaggtttagatgttgtacctgtcagcatatctgcactctatttggaagagcgtcggatggatccagccatctttggtcagagatgcaccaaagtctgacctgacagccccttttcttctcctgtggcctcctcgaatattaaaaatattgcatattaaatttctcttttacaaatggaaagcatcaggaaagctcgatgggaattgaataacttccttgaaataaataagtgacaactaaatgaggcacaaaaccaaggcaaaaacttcatgatttgtatgccttcttttcattgacggtgatgcttagtttagtgtagccatgagtgaaaaagaaaataaacttgtgccttatcgatcaaaatgtcactacctagacaaatggtccaaacaaaatcacctctatggacaaaagattccaatggataatttattgtaatgtgtatgatgaatcatgaatatctagctctaaattagcaggtcagggaaaaatcaagaaataaaaggcaatatttcttaggggataagatgcatctttcacatctcagttaaaaaaagcccgaacatcgccagtaaaagtgtttgtctttggttcagttttcctgacttccccaagtgcttgtatagttgaaaaatatatcataactgaatacaatgaaacatcaagaagcaagaactgatgagatagaacctaccctcaccagcaaagaagagaatatttccattaggaagtacccactttagtgcttttgacattcagagagttcaaaggcaggtaagcaaacctagcacatgttcagctgccgtgtcaggcccagattcttctcgaacatcagcatgagtgatgaaggccctatcatttgtagcctccaagggaccttaatctttgtagggggggcaaacaaccacgatcttcattgatagctgcaagcctgcaatactgtcactcgccacttgtctccaccttctatggtagcagctgatggagacattgctatcctccatcatcaccctcatgatatctaagt

>TaeST2.32420.1

tgggagtaaaccctcgactggctcgctctccctgcgaccggtggcgacggcctccgcctccagcctccacctccgttcggtgcatctccgtccgacgaccggatagcgccgccccccgcgccctccctcaagagaaccgcctccacagtccgtccctccggcagcagcgacggcgacggaccgggcgtcatccatctcccctctgcctcaaagcttctatcacctccggcgacgacgatggccaagaggtcaccctcctttcaccaaggcgctgtatcatgagctagcagagtatgtgcatctgtgctcctgccgctccacttccaggtgatggcgtcgggttggtcagagagggtgaagctctcaagttcccgccaaagtatcaggtttcctaaagatagcaaataaaaatgttcaaagaagttgatgctggaagtttcagtaaagtgtctatttcaacagcaaaactttttgctatggacgtgtcacatatgggcatttttttaatggagctaaatgcttattcttgctggatttttaaccgtagttagtatttaaaaaaacatgattagcacaataaattttatcctcagtagtgttgtgtaatttgtgcgactcgaccgcataacttattgttagcatgtaattgggttgaagtgttaatttgctgattatgtgtgtgttattattttctccaaaaaaatcctgaattattctctatcttcctgagaacttgtgtcatttctggacttatcaaggaaatatacagaaattttaattact

>TaeST2.32496.1

ttgcagagaagtagttggacatacgattcattccttccaaagatcaagtagcagatgggttcactaaagccttgccagtcaagctgtttgaagaattcaagaagaatcttaatgtaggatagttgagattaagggagggtgttagacttagagataactttgtgttgtaatctcaacaaactctctctccctctcgttcaactttctatctctgtaaacgacaaccgtatcctagcgataggcaatcttgtacgccacggcagaggctttctgcctcaatcaatatatacccgcggctcccttacgagggagtaggaacgcttccatctaaca

>TaeST2.32709.1

aaaaaaagtgaaatccaatagcaaaattttaaaacacacgtatcctcatacaacttcggcatcaaataagccagccaaacaacataaataataactcaaagttaccattttggacctagcttgtttgaattccaagtgtttggcctttatatagtcccttgagccttgtaaatcattgctgttgggagactgtacaacaactccaccatgaacaattcaacatatttacaagcacctattcttgtacgaaattcttatcagaagcttattgcaatgctttaggtgttgcccaaaggcccactattttagtagcattccgtttactcctttcccaacgtgaccactttctaccagttcacagagttagtgacgagaatctagtgattgcagccgatttaagcaatactatgtgtaggtcgttggcagcccaccacacctgttagagctcacggagccattgttgagctaggactgcaaaatacatgaaagtttaccaacttaattggttacccttgcgatatacatagctaagttttgaactatatagatagagtgcttaatcataattactgcaaatctgaagctgcacaattccaattaaatttgatgcatgggagatgacttaccttcttcatgaccctggtagttgtcactgagcattgcgataattgcaacactccaaataaattcaatgccgaagagatgagttgccttcttggcaatgaaccctcaatagtgaatttctaaggtccctgaatgaacatatgtggaagatattctacagttctagatatatggcacaaccttagttacagaatgattagtatacttacaatctaactgagaggccccacatatgcaaattcagacctccgataggaaacactgaatctcagcaggaaagatcaaatacatgcatccctgttcgaggtattaatatgggtaattcttaattgatgcgctagatgttatcttctcatccaattataaaagaactcgtgattataagaaaatcttactatattccatcaaaatatacaatcctgtgaggctaagataacagcaagagcataaaattctacaaaaaaaacaacttgtcacgacattttagaaatagttaatattttctcactggaaatgtctatttgtgagttgtatacttaacatggaatatactgatctatttgttgagttcttagtttcagatgttcgtatgcgactttatatttgctctgggtaaagaatgagcaaaatatcttttcttgaatatagcttcttatacccattttgctggttcatgataacctaatggtgatctcttgttgtagcccttatttcacttgattgaataaacaaaagcatcacatacaaaatcttatgcaggttctaacaagaatgaagtgagatagactactgattctacaatgtacaatatctggttaaatgatatttggccaagtattttttttctgcttctatcatctgtgagctcattattaagtgttagaaaataagttactttaagttgaattgaagtactcaaattagagctagtgcagtatgccacaaagaaaatattgtttgtcgagccaaaccacattaaaaaaaagaaaggaagcattagaagatcggagttaatagtttgtaccttcttcaacttgcataattgaattgaagtaataagcttttgtttcgttgccatggcatgcccggtgcatctccaatcctccctattatttcctcctttgacaac

>TaeST2.32869.1

cggctgaggtccaacaaatccccaaccctaatggcttgccaatggcgacggcggtgtagatcctgtacctagatgggcgaccttggctactggaggaagctttccatttctgcccctagatgtacctagctgggcgaccttggctactggaggaatcttggtcgtcttgtcgccggtggtcgggctgcacgggcggacctctcctgtgctgcgccgcgacgctgtccgagccaatctccagcacataagcctcgatgagcaagaagcaccaacaccgaagatgctcatgtccaagtggccagattacgaggagagagcaagacttgcttgatgccaaaacccctgtaactgagtggtctgtccactctgctttgcattgagtttttatcagagttgtagatcatggaccaatggaccaatccttgccgagaattgaaatcagaattatgtaattctgcatatggtagttgttaacctgctttatgcatgtttctactgattaattctgcttgtaataagcgtagattattgctggatcttggttgcttcgcttcctgccatgcttttgctattggaaagaattattgtgttcctgcatataagagctactgtgtttggatgatggata

>TaeST2.33087.1

aatttatctcctcaagtttagtttagtacaacacacacaaaaattatgcacaataaaacaacaaacggctccagctcccctgatctacttcaggaacacatacaacaacataggattgagatgggatgctgttatcttacaacaaactggaagaagaacacagataactagctatccaaactcatgacctcttctagcagtttctcaaaattcaaaaccacatatgtggcaattcggggcctctgcccaggcatttgccaccttcagccctccccaaagttgcctggtgacagaaaaaaatgctagtgcaaagacgatgaagctatcacctctatctagcggtttctcaaaaatcaaaacacatagcatgtgggaaactcggggcctctgctaggcaatttccaactccagccctccccaaagttgcctgctaacagaatgtgcaacgatgatagtgcaggcaatcagactaattatcagacattcagtctgactgaatcaccaaagctaaacgagcaatcagaaaggtactcgctggattccctcccaaaatccacgagcatcacatcacagattcatagccatatgaaatcaatcaatcatccatccatcaatccatcagtttatacaacaccacgactcagatttcgatttacatgaacagacacgacctgtttgagtttgacatgaggacatggaatttggcagtactctgcagtggagcgagccaaggacacggagtgccatctcaatcaaccgggaaaggggtagatcggcggctcagccaaggcagcgcacgacggcggaggggcagagcagaattggaggagccgctccttcttttgttcccatcgttggacgacaagtgctcagatccgacagggatctaaggtccgagccgcgggatgcatcgacccggtgagggatcgacacggtggagcacaggacgacgccgggagggagaagcgggggcgcgtcggcgccggtctagagcgagggagccggggtgtggggtatgtcggccgagaagaaggctggggatggaggatctccggtgccagcgcgagggcttcgatggaggcgcaacgcagtcggtctcgctggggcgtgg

>TaeST2.33564.1

gtgagcctaaacggcttcgtgcccctaccgccattaggcctcctgcaatcttgtctcgaaaaaaaaaagattaggcttcctgcaatctacagactagacaaggcggcgggatgatctaccggagctggtcgctgctctcgtcgacggtggcgatctggggcggtgctgccgccgcagggctcaccgtcgtctccctctctggcggcaaggaaaagtttcaggactacctgtgccgcgaaggcgataggctgaggcggcaggacagggccaccatggccagcgccaactgacggaccaggaaagaaagcaccacggagacgaagaaacgacatttcatcatatatccgcatccgtgacggggtcctcttggtccaataatcctcatttaccgtgctttaaacatggtgtggtctggtgtcctcatcgaatccaataatcgttgatcgacatgatgtggaacttggtacggcctgatgagcttcgctttccggcttgtcaagtggctcgaaccgttgtcttcgcaacaatctttgtcccattttggatttgatcactgtttcggatatgtgtgaataataaacaccgcggtccgtgggatccagctgaaatccacttggctgttctgtttggatactgtcggatatgtttttctttctggagagcttcagtattttttttccgatttataaactcgaactcttggc

>TaeST2.33590.1

gagactgccgaagcacactgatcacgggctcttttgctagcactagcagaccctaaagtgagggaagaatggaccattcttttgtgcttatacaaccattcttttgtttttcatttccagcttccttttgatcagtacaagcttctcactctgtttttcgccttattccaacgaaatcatcgataaaaaaaaacatgagaaagcacaagcataccatataattaaatcactccacacacagattaactttatttaataagcatttgatcatgctgcagctgaggcgaatcactaaaggaagcaagtggcgaccttggcgtggaaacagaaggcgaatgtaggagcagtgtataccatgaagctgtcgtagcagttccacgcaacttctccaaagcatggcacaaggtcgtcgctgatctctaggccgacgcagtggtacttgttgtcgtccttagccttggccaagcagttggccgtcacgacgccggcgacgaagcatgcctggaagctggcctccccggtgaccgagttggtggcgaagcacaccactgcggtgttcacgacgcacgccaaccggtgattcttcaggcttatgccgacacagagtttatcctctgtcagcagtgccgtcggcgccggcgccggcgtcgatgccggtgtggcggagacgccggctccatatcagcgctaggaccacgagggcctggatgatggccatcgagcttgctttgtttcttcctcttcttcttttttgtttgtttagggcttgg

>TaeST2.33863.1

tcctccgccgccgccagccaggagggcagctccaccccgctccatctcctccctgcagccaccatggccggcccaagcaagagctcatcaccggagtaggagcatcaccggcctcccctccgtcaacccctagcaccgcagcgcctccccgagtccgttctgacgccgatccgagtccgaggacgccttccccgatgtccttttcgtcctctacttcgtcttcttcacggacagcaagtccaccggagttcctcgcctcgtctccgtcgatattctccattcccgaccgcttcctcatgtcacaggtatactagtagtcgccgtggttcctcgcgcttcgcccccgctctggatcctctctcgcacaggccgcctccgtcccctcttcgttcaatctgtcgtcaccaaaatcctcatgttgcaggtacaggttatgcgatgatcttgacgcgagagcgatgtttgcttgtttggagttctacttctgcttcttcttcgatcagggataggttccaggttggcaacctgggctagcagggtggatgtcgtttgagtttctgtttgtgtttcatccgtagtcggatgttgctctcatgtattgtgatgttgtatttgtgtggcattgtatgcctcttgtatgtatccccatctattatgtaatgttgatgtaatgatatccaccttgcaaaagtgtttcaatatgcggttctatccttggtgggaccttcgagtctcttttggatagggtcgcatattgggcgtgac

>TaeST2.33863.2

tcctccgccgccgccagccaggagggcagctccaccccgctccatctcctccctgcagccaccatggccggcccaagcaagagctcatcaccggagtaggagcatcaccggcctcccctccgtcaacccctagcaccgcagcgcctccccgagtccgttctgacgccgatccgagtccgaggacgccttccccgatgtccttttcgtcctctacttcgtcttcttcacggacagcaagtccaccggagttcctcgcctcgtctccgtcgatattctccattcccgaccgcttcctcatgtcacaggtatactagtagtcgccgtggttcctcgcgcttcgcccccgctctggatcctctctcgcacaggccgcctccgtcccctcttcgttcaatctgtcgtcaccaaaatcctcatgttccagtaatgttgcgattgtgaggattcgttcgactactcccgttcgtcttcttcatggactcgttcttcttcctagcgggatttcagttgcaggtacaggttatgcgatgatcttgacgcgagagcgatgtttgcttgtttggagttctacttctgcttcttcttcgatcagggataggttccaggttggcaacctgggctagcagggtggatgtcgtttgagtttctgtttgtgtttcatccgtagtcggatgttgctctcatgtattgtgatgttgtatttgtgtggcattgtatgcctcttgtatgtatccccatctattatgtaatgttgatgtaatgatatccaccttgcaaaagtgtttcaatatgcggttctatccttggtgggaccttcgagtctcttttggatagggtcgcatattgggcgtgac

>TaeST2.33893.1

taaaagagacaccagctcacatgggtataacaactaacttaatatccagcaagtgcaacaacaattcagcaagaacaaacacatatgggatctcacacatacagtagcagaatactaactaacacatgacaagtacgaactgaacatattatcagtagcatagagtagatgatggaactatctcaaacatcaagcagcagaacaatattacaagttgaaccacctagttttctcacgaacacatcaatagttaactgagattcagattacaagctgtagaatattcagagcaaaaattcagaaaactacattaagagaagagagattcatagttgtctcctgcgctgtagatgttgtgggcgaaggagagcagcagggacaaagacgatcagcgaagccaagttggagaggatcagcaaggggtcgttggagaggatccacagctccgtttcttgaggttgaagacgaggaagtgcagcaggttggcatcgtcgttggagagaggcggtggatcgggcgcctccaccaatctcccggcgccctcttctccctttctctccttcgatctggttcttctcctcgttcccctcaatcccctgcttcgatctggtggtggttgagtggtggaaggcagcgaggagttggagagcagcaggtcggcgatcaggggcggcagcaaggtggaatggcctctgatctcctcttcttcttccttccctcgatcacccttttcttgcctgattcgatctggtggtaggtgagtcgtggaaggcagcaaggagttggggagaaaaaggcagtcggtttgatgattttctttccttcgaccccctcaactcctcttctttctcgtggcctcacgcgtgggatcagatatgcctctcgctggtcgc

>TaeST2.34311.1

tttttaaaggcttgattgaccctttggctcttgttggagcgcgtaattcaagaatggatggaaggtctgccgccgcttgttttttggtgatgcttgtgttgttcggaagcactacatctgcagagaaatgtttgacttcttcgttttatcggctggtgttttgtaccaagggtatttgcaagtctgtatgcgtgggttggttcggttaccctgggcagaagatccgcgaactttggtgcagtaattttttctttggcacgtgcacctgcaaggtttgcgttgatggatagagcttaatggatatgctaagtgcacttcatgttatcaataaataatccatcacatcgtgtccttaaatgacgtatgttatgca

>TaeST2.34424.1

tttcgtagtctgtcagacaacctctgggccaccctgcacaggcatgagcaaggcaggaacgatcctctacagccgtggattgccccaatcagggggaatcctggccgttaatcacgctctggcccagaggtcagaatgcatcaaccagactttagtgcttgcctccttattggtaaaggatgcagcggtacgagcctatagaacagatcctgttc

>TaeST2.34472.1

gagatacaagcccccaacgttgtaatacaagatcagataaaaaccaacaactttgtaatacaagatgagggctacacatgttctactcctagtccttgccctcttcatgctatccccagatctggcgaaggctaagctgcactgcacggatttcatcatagtcaaccagtgcgacgtggacgagatcacatgccaacattactgctacagccagttcaatggaaccggcaagtgcatccccaacaaaggatgccagtgtcatttctgctatgcaactccaccccagccatcgcctgctagcagctcctagtcgtagttttctctgtaattttctgtttcttcaaggtcaatgtacaacagttatgaacatgattcgtttctagtgcaacaactatggcgccataattaattacatgtactgtggcctatactgccaagttatatgttaatgcaatgtctgttttgctttatatataaatgttcgttatagtcaaggagcgagaatacatgccataatatatagtcatgtttgttataaatgttttgctttatgta

>TaeST2.34685.1

ttctaatcgacgtgtaacagttcggactgctttcttggttctgtagaactttacaaggtccatgtagtagtagctgttacaaacattctatcaccaactccgacaagcggcaaaccaaaaaaatttaaaacaagcagtgttcctgtaaaatccctagatattacacgaataatttggcggaaggccaggctctccatgggcgggtcagcagcgtgccaaaaatcttcctctaggtgaaactatgaccacacaaaactgtagtacctagcgcgccatccgctgccgggcatatgtagatcaggtggacacaagggacgccggcgatgtggttgttgtgttcaactgcccagccgcttgtcggaaactgatcgggcccaaacgaagcagtgcggcaggcaagcagcacgtcctctgcgccgtctacttgtttgggcagtctgatgctttgtgatgttattcgagtgatttcgtgaggcctacctgtccctgctgccaaaggatgagccaccacgaccaccgtaagacgatgagcgttcaccaaaggacgatgatctgtcaccgaaggatgacgagccaccacgcccaccataggacgagcgg

>TaeST2.35170.1

gcttacttgctttcgggactatttattcttggagtcatgggagtggtcaagatgacaggactgttatgcctgttcttgctttgccacttttagttcctggttcggaagcaaagatgtgcactcgagatagccggacctatacgtctgtaccatgcaagcatgatccttgtgttgatgcatgccacaaggagggctctatcgaagggttttgttacctattcttcggagcttgtacatgccaatgtgagtgttgaataagaaacatgttgcaactatcatagggtgtgtgtaacaaataataatgttgagtagtatgatgacttgacaatgggcaatgcaagaggttgagaatctaccaaatattccttaattctatatctactaaaggagggtgcgtttctccaaatgtttggattgtccctacgtcacatgtgccagaattattgatgtgggtcgagccatgtaagtgggcttcctattttaatgggaaatagaaaaatggtgagtcaatta

>TaeST2.35243.1

tgaagattatagtgttttttgagcatatggtgagagaaaatcaaatcagaagattacatgatacttagtggggttaatttgaagctcatcccccaaatatagaaaagtaaaatacattctctctcatgaagagatagtttcttctgaccaacctaatgataacatcatttgttcttgcttgtcatgacataggacactcctgtaataaagaacaacttatgacaaagtcgaaaacctgatttagccccaaatctgtttcttcagacactgaattgttttttccaactcagtcttctcagcctctcgagcttcagatgcttgcttaatcatttccaccgtccccacttcccgttttggttgaaaaaattcttgatctgaatttctcctacttgagcccacctgaccatatttggcctgctcaaatggccttagatagtagagtgttctggtacaaaccactttctcagctacttcaacattttcataagccaaaatcttctcggacttcttcttgccctttttgccgctgtcctccttggacgccgtcttacccgccgatggcctcttatccgccgtgggcttcttcccctccggggtcttctcgggcaccgacttcttctccgccctgggcttcttccccttcggggtcttctcgggcaccggcttcttctccaccttgggtgccatgtgtactttgacggtgtggtgcggtggtcgagtttggttggaatgtttgggctggcggac

>TaeST2.35331.1

gttttcttattctcattagaaaattaatttttaaagaaatcacattccaaatagttcatgcatatactccagcctccagtagggaaacttaatcatgcaaagtaaaatattttcccattcaaatgacatcaagctaagcatgcgatcattacaacatattatttttattgcaagatattgatgacattacttttcatcgcaacaatctggcagtgtcttaatgcagcggatgagcatgagggaaggttgcatgtaaagaggtgcaattaggaccacaaatgcattcgtc

>TaeST2.35400.1

taaaaggattgccatcttatttgttctccttcactactccaaagtttcatagcaaccaacaacagtagatatcaacgccatatacggcgaaaatgaagagaagtattgtgacacaactcctggtcctcgccttccttctgctaacatatggtgcgacagcatataaagattgcaagagaaaagtcatcccacaacccggtggtcatgattgtgatttaagtgaatgccaggatcaagtgggtcaagagctcttgaagtttcactgccctccaaattgcgcgtgcaacgccagtgcgaagtgtgttccttttggttgtgagtgcacaatatgcatatacgctaataagcaaagtacacccctccaacaaacagaagacggatctagctgatgatgtacaaggacatgttccctgaaataaatgtgattgagacatatgaaaattgattttggtattgccatgtggattaaatgaaattagagattatatatatgctataattatgtatgattttcacaagtatatctaccatatattagtaaaaca

>TaeST2.35551.1

tgattgtcactgcaaagccaccgtttgaagcataactaacatcagactggaccatcccggattgagcatatcacaagcatacatatcttgatgagacatggtagcatcatttttagtacatggccaacaagacagagtaaatatgatcaccactcaacatgcattgctatttaaaacgataagctctactatcttgatggtcataatagaaaagctctagcattcccctccagttctaccacttcttgctcataagcttcttgttctgggagaggtcacaccaagcaaacacgattcagttgagcacagacacgtacagatgcataatcaaaaaacacagttaaatatatcttccatagaaattctctcaatcgtcagaaagggaagactttgataaaagcttcttatatccatctgcggtcttgatgtttccggttgtcagggcatcatgcatctacaaatacaaggaaaaaaatatactttacattacaaggaaagaaataggaaattatgttctctaatcctgatcgtgtatatgtattgaacaattaataatgcaattcaagttcttttgctaactcatagatgacaaaagtaaaaccttgatgctgcagcacagaggcacatgtcagaccactgaagagaaacttacttacgaagccttttatcccaaacaagttggggtaggctagatatgaaaccctttcacgaggacttcccaggaggtcacccatcctagtactactctcgcccaaatgggtttcatacccaaaagatggctagtttttatgttggctcgccaagcctatcacaacccttagatatgaaaccctttcacgaggacttcctaggaggtcacccatcctagtactattctcgctcaaatgggtttcatacctatgggactggctagtttttacgttggctcgccaagcctatcacaaccctcctcctttacccgggcttgggaccggctatgcctaga

>TaeST2.35620.1

tttgtttctcttttgctaacgatttggagcaaataaaataaattgtatatattactcatttcaacataggtttatttgtagcaattcaagactactaccacacctgcaaaaaagaagtcgagactaccagggatcaacatttccctcaaaagaaaaaaaaaactgcagggatcaatacggccatggatactgaaataggcaaaggacttcaggtcacaccaatcctctatggcttggtatcctcc

>TaeST2.35653.1

ccatcgacacaggaaggaaaggaatatagctaaccgcttctcatggctcctaccagtagcaacgctcgggtcttttgtctggtggctctcctggtcatgtctaccaccttcttgtcctgtgatgcagcaggtgggtggacgaagttctgcgtacccgagaaagcctgccagaccccagcaggacttcagggcgactatgtgtgcaaggttgactgcggacgccaaggttatgatgaggataaaagccactgcgggagtggcatttgctgctgtgaaaagtaatataagccgtcaatatgttttgttatgtgctggaacgatttcgcgtagtgagggagacttattttctatgcacacctaggtttagttcatacagaaacaatcagtaaactgtaatggttgtattcttgagacaaattctgcaatggttgatctatatgtcttccttcttttgtgaaag

>TaeST2.35711.1

ttcatttcgaattttttcttttcgctttctcgccccctcgtctcttccccttcctatttttccagcggcggatacctcggttggtggtcaggtgtccagcgctctcccatcagttgcgccgccgcatctcccggccgaggtttcttaccttgaccgtgtgcactctgggttgaattctcttcctgacagcaagcctagaattctggtatggagaggtaacaaagttaaggatttctcggaattacataagagcaacagtcgttcgtatggcaagaggccgttgaagcgtttatcaccatcagtttacgcacagaagtcttgtgaaaagttttcatgttcagtaacctatagtatcccctttgccaataagatttcaacatcagaagttcatgctccagctgttgataatgtctcttttgcctgtaaagctgaaacggcttatgcttcacggtttggaaatgatgttgtgcaacaaatagttggacttgttcagtcttcaaatgccgctaagcctcagacttttcaagattggtcggagtcccttgtttcaagatttatactggatttacagtgtccagtgtaattctaagtggatttacactgtaattcctactggatttacactgtaattctatgtgaatttacagtgtaattcttactggatttacactgtaattcctactggatttgtagtgtcttagtggatttgaactaaaatgcttggtgtaatatttagtgaattttacaatgttttacggtggattcatattggatttacagtgtttttcttagtggatttacagcggtacttatagtgtaaatcttattaggaatatgcaacttgtattctcatgaggccataggtcgatatatatgcatgtacaggtgtggaacatatgcaggaaactctttatacaatgggataaatacaaaggggtacatgacttatattataa

>TaeST2.35713.1

gtccctcaaacaagatccccactagcccacctccctgtagctcgccgacggcggcggcggtcagatccggtgcttgcgagaggactacgcccaccgctcctcctccatgcgcctctcgacgccgtactgcgcatcatcgaccaggaccctggccgccgccgccacggtctcctcctccactcgtgcatctacccaggaatccaggaggcatcacctggccttctctgtgccggattccgacaggtttgcgaccaagctgaagaaccctggcacagacttgttcgcgaagacgcaacccgatggccgcgcgcgccaggtgttcttcctcgaccgggacggtagatttgacaactggatgcccaggcttagataggaacagaggacatagcagcagaagaacagatgcattggatagcttctgctatcgattttccagatcatttcactagcactttgacttattgagagcggcgaagtgaaggaagctctgtgtgcgaaaataaccacacctttgtgtgtaaaaagagtgctgatatattttcagtgtaactggtgtttctgttcacgcttaagaagtgcttcatataatggcctaattacaattcaagttgagcgtgttaatcattatatataataaattgggtttctga

>TaeST2.35847.1

caccagcagctaacgccgagcactccgtctcagcatctcaccaaccagccgaagcgatggcctccagcgtggcggcgatgctgaagaacaagatgctcgtcgcctgcgtcatgctcctcgcggtcctgctgatggcgacgcagatggcggcggcggcggcgggcgacgacggcgcggcacgccgtcgcctgctcgatgcgtgccagtacagaaactgcaactgccgcacatgctacattttccactgggcctgttgcggcttgtgctgcccgccggcgtaggggacggcggcacccacgggccgctgcagtttctcacctgcagaggtttgaataatgcacatgtacgtggtcacgttacacgtggctgctgttgtctgtcccaatgctagtcgtggtcgtcgtcgtcgttgtcagtggttctgatgacgcgcgctctttgcaatttactgtcaagttctgcgtgtgcgcgactgtgcatgcaatgcacgcttaagtgaatgggaatgctacttcatctatct

>TaeST2.36031.1

ttttctggagaacttaatgtttaagtaaaatgtacaaaacattataggaaaataacacatacaacagcagataaaatttcatttcactcctacagctcactacatcatagacaatacatgacaatcattcaattttttactaggcagccgagtacatcattcaatttcaaataaacagtactgctcctggaagcaaaataggccgccacacgccatggtgccccaagtaaccaacaacgctgcttgcccccttggcccagctccttgctgccgacgatggacgagactaggatgcggagctccctgcacatgacatattgtgtcaatgcttgcagcaatagaaggaactcacataccacagaacttgcacagtagtgtatcaacacccgaaagattgatgaattgaactgttatgtaaccaagttagagctggcagaagttacaggtacataaacatccatagagctagcatgttgtatttctgtagaaagaagcatcaaatcactattctacacaaaaagctagcccactattctacacaaaaagctagcccatacatgcacagacaccagaacggtacatagaatgaagcatagggctagtacttgctaccctgacatttcaggcattctagtgtttaggagatttgacttccagcagaaaactaagtcgatagacttagcacttgtgacttcaactaacttgaccagtgctaagttattaatagacttacttgtgacttcaactaacttgtggactgattttgctcaagtttcttaatagacttacaatgtttagcttccaaaaattaaatcacaaagtttagcttacagaaacaaaattacaaggttttctatctgcaatcagtacaaacttgatagtgctacaagtgctaatgatccaacagcgtcccagctccacacatcctagtactagatttcctcacacttgatagtgctacaagcaagcaagcaggcaaacatgcaaatactagtagtactggttgtctaatgaatgggatacatgattctcctcaaataaattgacaaggtttccttcttttttctagttaatgacaacaagtcatcaatcaagcaagcaacatcacattggtacagcagctagcatcgttcaacatatatggggtgtgagcagtcaacacctactaattttattttatttgtttgcatcaagtcagcacctactagatgggatggaaatgcatcttctgtattagttgtggagcatgcgtgtattattaacaacaaaatcaagggtcgacactagtgaaagagcagacaggaatatttgcttgcaaccagacaacattgcttgtgcgaggtgaggcgacgcgaagagcagctcctcgctcttggcgccgagcttgacgaggggagtgggctccatggaacctgcgacaacccgacctatgtaaatcataaggtcttgattagatataaacagtagtactgaatgatagatataaacattagtattgcctgagctcagtttttttgcaaggataagcatgtcctaaaccgccattgtgagatcgttgaagtaatagatgccagaatgtgagaatggataaaacagaacatgaatgttctcaaatgccatgaagtgagaaagcaggtagtcagaatgacgaggcatgttagtgccatgaaatgtgagaaggtttggatggttaaacctcctcctccagctccggtggtagaggaggatggacctgaggtagcctttcaagtagtcttttcggatggatgtaaaacttgagaatatagtatactacagtagaactaattttcatatgcagcataatttcaaccacccaacttaattctagccctccattctgaaggatctactactgaaccatgcagaacaaacaacaacaaaaaataacagcatttcgaacagtataaacagagagcagccatgccaataaactaataataaaattccgtgatagataagagaaacacgggactacaacacagaatcgacagcagctgctacatctaatttaaataagaccagatctacagcatattttcaatcataaggtggcattctaacttaaattgacaagccatcctaacttaattgacaagccatccattcctaaggatctaatttgagaatctgacatagaacaatctcagaaacaggggactacaggtgtttatgtcccttgccatagttcatttgggtagcagttgtacatgtggtatgtgcttatgatttgggtagcacatttctctcatgcacaaagttgtaccagtgttgttattaatctataaggttattactgttctgaacacgccacattgcagtgctagggtatt

>TaeST2.36075.1

tgcaggaaaatcgagtcccagtaccaaggataatctttatatatgttcaccgtatcctgagcaaatttacatacaaatatacatagatactgcacacacgagtaatataagcactactagcattaaacaaccccgtatcttcttcactaattatttattcatcaggagctcaccctcaggttttagcggcgcccgcgcgcggcctaaacaagggaattaagcgaaagaacgccaccaataaccaacccgccatggaaccatgcatcttgctcttgctcagctccgccgtggatcaggcggcggctgaggagggcgcgggcgtcatgggcacgacctcggactggtagcggtggaagttggcgatgtagatgccggccttgttgtccacgtcccgctcgccgacgtagtagatgccgtcctggtcgctcggcctcacgcgctgcggccgccggtacccggccgccgccgactcctcgtactccccggacgaagagaagcaaccgcccattgccttgctcggttcttagcttcttccctcggagcttagctagctagcttcgatgatcttgggctcttggcacggtggggga

>TaeST2.36157.1

gtttgctatcaaactctcaaaaaggaaaaacaatgttactctgttttttggagaaccttcctgtagagtaccttgacagagacatatgcgatcgaggatatattcgatggattttcacgcaaaacttggaggatattttttgcagatagtttggtgttctcatggttattttgtagtgattttccttccgttcattacttggtaaccatctaccgcagctcagctattgtccagttgctgtccttatggtgagtgattgatggtgcatgttttcctattagtttctttctatgccaatgtctgaattttatcttgccgttttgcatttctccaggcctttaattctcactgtgattccccgagatgcgctattagtaggcatccacttcctaacaaaaatttggcggtcaacttgaacgagtcatttatctagctacaacaaccttcattgcttttgcagtcaacttgaacgagtctatacctgccaaatatttacacgttagtatagccagaaagtcagatgatcgttccatgttggttagccgtgataaatcaaatttctagagtagcagatgattcgtgtcaggtttcatttagagattgaaatatgtcttgactctaaaataagtagtacaacgtccagttctgcataggtgaaagcatactatctaaaactggacggccttctaaagctctgaaactactagttgtgtaatatgtatccccttttcatactctatctttgctttctacttacttacttacgaagccttttatcccaaacaagttggggtaggctagatatgaaaccctttcacgaggacttcccaggaggtcacccatcctagtactactctcgcccaaatgggtttcatacccaaaagactggctagtttttacgttggctcgccaagcctatcacaacccttagatatgaaaccctttcacgaggacttcctaggaggtcacccatcctagtactattctcgctcaaatgggtttcatacctatgggactggctagtttttacgttggctcgccaagcctatcacaaccctcctcctttacccgggcttgggaccggctatgcctagaagacataggcggagttatatgctttctacttactaggaaaaaatgcgtgagaacgatggcctagaggacgcgaattgcactcaaatatatgcaggtttcttgccaaactaactgccactattttatttcaggtgcttcttctgtaacttagtctattttgctggctcttctcatttcctggcgaactttttagtctgtacagtcgaatcgttggatgttgctttctactatctatagaggttctatccaaactattagctgcctgacttgattttttgaaatacaccgtcctgatagaccaagtggacccacacatttccatgggattttagaaaaatatgtgaatttgcatacattttctaaatttctatcttttcttatccatgcttccccatagatatatagcaggctcaaaaaaaatgaatgtctgattgtgtccaaatttcaaggaagcgtgcttcacacttctctttctgcaatatgtttcagagtatatgatttaattttcctactttcctagttacaatactgtgtacttcattcttaggtttgctttgggaaaaataggtatgcaggttattgaaagctccatttttctccattatatagatcatatagctttgtgatcaaaggtaggttgtcacatatgggatgttgcttgtgaaagttatatacagccatgagtccctggcaaaataatcgcagctcatgttactaggatcagggaacctcgaccatttttctgaaacatgtcgcagctcatgttcctactttctcagctacactactatgtactaaattcttaccttcacttttggaagttacattgctttcttttccctcataaacttgctaacaatattggcgaagtaatcccgacttcagatttcccattgaataaataactaatttagtttgttaaaaaattgtaggtcaagatccatgaataggagcaaggcatcacaccagctctgagaagaaaaggatatgaagacaaagatagaccagtgttctgctccgaagcaaaaacattttgcatgtaataatgaactataatttaccacaatctctctaaaataggcctacctattaccagtccgttgcttgcttttgtattaggaagtatgacccagtcatatatatttacattcacgttgtttagttatggtttgtatgaatgaaattacctttttaatcgagcttcaaaattcttatgtgcagtgaaattgtatctagcactttgtaaagaacaaatgaaacacatttcaa

>TaeST2.36184.1

tatgacagggacaaatatatatcacagcacaaacatatatattttcacatgaacatagttgacgttggaactacagtacacacatagccacacatatgggcacaaggtactacaccaaggttcaggttacacgagtgacaacacaaacgaaattacacaagccactaaactagtactcgcatatcaacagaatattactaaaccactttaatactggacagttcaccaattcttacaaggcatcgtacaatatttttgcaagaaaaagagagagagaagcagcagtttgggattcagagaagtacaggatcaacaacaggtaaagaacaccagcaggttgttttcttcagtaaaggaacactagcaaggaaagggaataggagcagcgtggggataggataagaagcagtaggaggaggttgggagagcagcagcaggggcgagggaaggagcggcaaagacgtggagaaagggagtagcagtagccgcaggagaggaagcaagcagaagggcacaggagaaggagaaggagaagaagcagcagcagattggcgatggggaagaagaggagtagcagaggagaagttcacaggtggaggagcagcagtgaggcaagagaagatggagcagcagatgtcgggcgcaagtagagaatcagcagggacgtcggaaaagcagcagcagcgtggggatggtcgcgcgaggcaccagtcaaccttgatcccatagtctggaagggtgcgcgagatggtgatggtggtgtggggcgccgggtgatggagtggctagtgggaggtggtgatgcggatggatggatggtgatgaagtggtgctggtggcgcgccgccatgctggcctggccgcggcggcggccggcggtggagaagatggggagagggagagaggtga

>TaeST2.36241.1

atggcgtcgcgcctccgctccttctcccgtcccgtcgcagccgccttcctccgctcagcttccgcccggagccccgcggcctctctcccccgcgctctctctccaacccccagggcttcttccctggggcggcaggtggcgctggcacgatcactgcagccgctgcacagcgcaatctcggcggcgaggctaacgtcacggcttggggcagaggtggcccgggcagtgtcgcaggaaactggtctgagtgtgcctcgataatgaaagaggttgggacagacgcagcggacttttcaagaaatttattcccttcaataatgggttttgcttgttggcattcttccgcacattaatatgtgatgtactgctcagaagcaattactatgtgcacttcatatcagctcgagccttaactgaacagtgttatatttgttaacgatctagttatgggaagaactgctgctctagtttgtccttttaagactacccacaatgggagtaacataggtagtaacatcacatatttctaaataaaatagatgtggcaaacaata

>TaeST2.36514.1

cccattccatgtgcgtcatttacttgcgttcccttccagcttaactccctccatatccctcgcctctgcacttcgccttgcagcaccgttccgtcgttgccttcttccccacatcatcatcgtcgcccctccacagacgccgggagaccgactcggcgatggatcctccgcccgctcagtccatgtctgcgaccgacgacctgcacgacaagagcctcgacggatgctgttcttgctgctacgactgctgctccagcatcctggacttcctgtgctgcgcttgatgctgctgctcctcctcctgccgttcccacggcttacggcggaagcgcgcggagatgcctgacttatttcggagtactaatatagtcattgggtgatatattttatatagcctgtatgtaataaggagatttatggatcaagtgctgtaatgtgttgacttatcttccgctaaagtagtcgtgcaatttggtctgggaaatttacccaaat

>TaeST2.36847.1

tgcgtgtgtcttggactattgccttgccgaagacgacgatctctcttcgcctctccgtcctccccaggtctcctcctctgctcgtctctgcatttagtacttcggttgatggcttcgcctgcagcagcagcagagttcactcgggaaactgttcgccggtcactgatcgcaatctctcagtccctccctgagacttcgctcctgaaccccaaggccaacccaccaagcaccacccctggagcaaacacatatcgtgatgatggggcagcgaagtgcagatcgaagctgatctcgatctcatacccgtccactcctgatgccctgtccacgccgtgcccccagaagaacgctgcaaccgtgtgatcactgacgcctaagcgtgcatacataagtgtttttcttcttcttctgaagttcttcacccttctgctgtcttggtcttggtgtggaatggatgtactccagtgcttgtgtttcctgtattcaaactgaaacatttgggaaggaaatggagacaattgtacagt

>TaeST2.37230.1

tttttcatttcgcctctgtagctaccaggttcccttccactccctctcgttcccttgtcattcccaataggcaaccagaacagagtcgagagagagattcatggcgactcggtggttccctatccgctcgattcgctaggctggccggcgttctccctccggctgcttcactcccgtccggctgcccaccccctcctactcccccgctgttcgcgccgttagtctgcagcatctctcgaccgccgcacccacccccggcctctatttctgcctcggcgacttcgctggtctttgtgattcggggatttcttctctaattctcagtgggttttcactgtaattccgcttattagctgtaatatccaattggatttacagcataaatcaagttgcactgatatcgccagtggttttagtgtaattgacgttactggggtctaatagcaagagaattaagtcggtcattttggcttcatcttacatgaaggagtgcatcttcatcttgctggagagggaactgttcacagggaggtggagaagattgtcaggtgaggatctcttacctgtgcatccatgattttctccaggtttgccctctggatcttggaggaattcccccggtgatttcttttttgaatgggtggacaagcacatcgacacattcaggggacaggaacaaaacggagcacatgtgacactagttaaactctgtgttgccaaaaactatgactggaaacgaattggatttcagacagttccaaaaacacatatcatcttggatttattagtggatttacagtgtctttttagtggattttacagtggtttctttaatgttccttacagtgtaattcttagtgttttatcttagcagtttttcagtgcaaaagtgtaattctagtggattttacactgtaattcttagtgtttaatcttagcagttttttagtggatttacagtgtaattttagtggatttttgcagtgtcactttaatgcatctttcactataattctcaatgggcatgatagaaattactatggtttttagtgaa

>TaeST2.37388.4

attgaacataagtcacatttttcctcatggcactcatcaagagaaacacaaggatggtcatggacaacatagttgttgctttattggtcattgtgttcatgttctcaacgttgccgtcctgccaagcaattcaacgccgctcatgcagagatttagcgggttgcactcatgagtcttgccagagtgactgcttagaaagaggctttcgcccaccttatcaattggaatgcgaaaccaaccagcatcatcttcagtgctgttgtagacagttcggttccatattaaaatgagagccacaaagtttcttagcttatgcaatgcaatgttgtatactgatgccaataattgaataaacttatattttctaataaaaattggtattagaggcatcaatcctattggattgccatggaacattttgatgaaggaagactgacc

>TaeST2.37388.6

attgaacataagtcatatttttcctcatggcactcatcaagagaaacacaaggatggtcatgtacaacatagctgttgccttattggtcattgtgttcatgttctcaaagttgccgtcctgccaagcaattcaacgacgctcatgcagagatttagcgggttgcactcatgagtcgtgccagagtgactgcttagaaagaggctttcgcccaccttatcaattggaatgcgaaaccaaccagcatcatcttcagtgctgctgtagacaattcggttccatatcaaaatgagagccacaaagtttctcagcttatgcaatgcaattctgtatactgatgccaataattgaataaacttatattttctta

>TaeST2.37413.1

agtggtgatttggcgaaatttggaaatactagttctgcatcatggcatggatactgttaagggagatatcatctatttatccggacaaagcgcatatgcgaagcctttgattcgaggtaaaaatggtgcatcgcctctccctacaaggtgttagttttagcatctagttctgcatcatggctgtaaactgaacttggttcagttaaaaactgcactgtccaatttggacagcaagcttagatctgaaaaatgagttagctaggcttagaattttgagttggcccctttacataagttttagataaagttccaaggtttctttagagtatttatttgtgtcgtttggatgtacggtttgagagcagccatcaatttagtttctgtcccgaaatatatgttcctgggtacagtattttgatatgggagattaggccctgttagaggtggtattaaaataaagttacaacatgaaaattttagaggctgttctgtagatacttaaacacatatcacttgtcaggtttcgttcgttatattaaatactatgaaattatcaaagtgggatgtactatgttggacagaattttatgtttggacttgggtgaatgtaaccagacttatactctcagattttgtcatagttgatggtgtgaggacttataatgtgataaagtatatacgttgttgcaacaccaaaatcattgaaatatatttcctttttttgctgccgatgacatctccataaagtaaggatttagataacatcatatgttcagagttcagttcttttcttatctgaaactaacatattttacaatgggcatgatggatgttgcaagttcattgatgacagccaagggttcatgctcggaaacgactacatcttgctctacgttgtcggctcagctagagtccttaatcatgaggagatgggacacgtcgattgttagagtacacttgacaggggtgaccgacgctgactagcgcagccggtgggcgttgtgctcttcaaccactcagaggcggacttctccgtgaagctggtggccatgtcgcatagatgattgtccaggtgatttcaacgccggaggtcgccgaggtggtggacctcgacgccaccgtccgggggagggaggattcgggtccaccgacgtctaagctccgagccttggtagatacatctagggaagttgtctgtttaggttgatgatggaacaccagggaaggtacccacctttcgatgatcattgtgtgtgtctgaagttaaatggaatcttgagaatgttatgtgatgttggttaggtttgcactggacgtattgccatccatgaatactacatctaacttttatttttttaattcctaaattattagtagtagcgtgggaaacaaatgagcgctactagtagttaggttgatgaattcttctagtgaactatgcatca

>TaeST2.37434.1

acccaccacggccacgcaccgccgggccgctcctcctcctcctccttcgcccaagatccatggccatggaggtaagatgcagcgggcaaggctgcctcatcgacatcaagcttcgtggtgatgatgctgggcatggacgaacagcaagtagcagcatcaccaccattgcgggaagctcggtggtggcgagatctattcattgaggcatcaccaccactcctccgacaagcacgccgctggttgacagctcttcctcatcttctaattcaatttttctaggtagaagagatgcatctccttgtttttttaaacaagtctattatttaatcaatctcttgagatgcatctgctttcgtgtgtgattgtgtcttctgtgtggattttctgcagcacacatagaaagaaagatcgatctaattttgtttcctctattgcagttttcgactagtgactggtctttttttctgtgtaattttcctcaggaaaattgattggtcttagtgtgtaattttttgtaatctttagcgatactttgattgaagaaatgaggacctttgtgctggcta

>TaeST2.37451.1

ccacacgacggctcaccctcgtcagcgacagctcccctctccctataatccttagccccctgccatctgggtgcgccgtccccttccaggcacagccaccggcgacctttccttcaccatcaccgtctctccaccctgagctccgacctacccaccatgtcaagcaggtccagatccgccacggctcccgcagccacgcgccggcagaccaatcaggggcacgacgtggtctcttccccctcttcacctctgtttgtttggtttgattgatgttgttcgttctcttttattcaatttggcaggcgcgcaggtgcacgacgtgtggttatactcttccactctcgtggccaccattctgcttcctcccctcccctggttcagcagcagcagtagtgacacgcgatttggagggaataagctgagcaattgcagccgtcctcctcgcctaggaggaggaggagctccatcacattagcagcagctcaggacatgaagccgtggctcttcaacgtcatccctggccccaccgacaaacccaggatcgccgtggactacaaggtgtacccgtgcaatgcttgtttccttgaatcagaagctcgatgcaagcaaactgcagtgtttaactgaatgtgttttgataccattctctgttttagctagggagaacagactagtgttttttgtgcacgctattccttcggaacttgtatgtctgtgatcaaataacttttatgacacatgaagtataacagtctgttctgtctcatttgatcaattaatctaaacactgatggtcggttgtatttcaag

>TaeST2.37468.1

agttggtcaaactttacgaagcttgacttcagacaaatcttatatgtgaactaaaaaagaccgaagggagtacataaaccaacaccccttacacaaacttcaatgtaaaatgagagagaattttatttattatctcacatgctattagacaaactagatacaacccattgaagttgttgtatgttaaggcatggacgccgataacggccgaacgttcggtctgggacctccccacaccgaacgattcgttcggaaggaatctgggcatcgatcgaacgaattagttcgggacgagaggagccccagcccgaacaccaagcatccctcccaacagcgcgaacaccctctctccatcgccccaacgggccttttccctgaaaaagcgaaaaaggaaaaataagcccagtaccaaaacaaaaggaaaaaaatccacttttttctgtgacaagaaaaagcccagtttttgttgtgctatgtaaaaatgacattattaacttcaaaagcaaaaattgctactggtaaaaaataatatatatgttttgcaatgacacaaaaggaaaaaagtcatggcaattccatatgtccatacatggtggcaaaataaattcttgattaaaattgccactagcaattgtattgacatgtatgtaaatgttagaaattgccgtgacaaatataggtaattttgccatgtcaagtaaaagttcgttgggcttaaatcatattaaacacataaccaaaatcatattaaaatttgcatatatttgacatggcaaaaaaagtaaaatctgccatgttaataaaggtcaaataaatagtaaaacttgccattacaatagaagttaaaaattgccatggggatttcggtaaatttccaagttaataaagataaattagccatgacaaataaaaagtaaaacttgccatggcaataaaagttaagtattgccatagggatttagttcaatttgccatgttactaagggtcaattttccatcggcaaataaaaagtaaaacttgccatggtaatgaaatttagaagttaaatattgccaaggggatttagataaatttgccatgtgaatgaaggtatattagtcatgggtaatataagtaaaaattgacatggaaaaagttaaatattgccatgcgtatttaggtaaattcgccatgttaataaaggtatattagccatggcaaataaataaaaaaacttgccatggcaataaaagttaaatattgccatagggatttaggtgaatttgccatgatattataggtaaatttgccatggcaaataaatagtaaaatttgccatgtcaataaaactgaaaacttgccatgggatttaggtcaatttgccatgttaaataaaggtcaaataaaaaagtaaaacttgccatggcaatatttcccattttgtattgtttttgacaatttagagaattgacatggcaaaatgcaatggtttagaaggaaaaattagaattgttgatagctatatagtggcaattatatgaaaagcaacatggcaaattgtacttcaaaatatttttgccatgtgaaacatatcataaattgcacggatcgaaacttaagtcgccatgggatcgcatcataatttgccatgattggcatagcatggcgcacgcaacagataacgtagtctttggcataccatagcaggaacaacccgcattcaccagcggcaggaagcggcaggaagcggcacacacgcaccagcggcaggaatagcacacacgcaccagcggctatggcttctagtctggacctcccggcgctctgcgcggctcctccgcagcctcgactactggacctcccggcggccggcgcggctcctccgcagccccggctactggaccttccagcgtccggcgctgctcctcctggaacaccacgaattcatccaccagaaagaggcccgcgtgttatcttagacttctagcataaaaggggagaggagggggtggctgaatgcgtctgaccttgacgacagacgcggaataagagcggggatcgggatcctgccgtgcgggttggcggggacggcggcggtggcagatggcggcggagctcgccgagatcccccaccccacacgccgacctcgcatacgacgccatagctgcggcgtcgaaggagttcggggccggccgtccatggcagggatgcggcgtcgaaggagttcggggccggccgtccatggtggggatgcggtggaggagatgcccgcgatcgctggagcctagccatgccgctcaccatccccacgcccggcgatctgctccgaccgccggaggccttcgccgccgtccccgcctgcccaacgctggcatctgatgttgtgtagt

>TaeST2.37602.1

cgcggctcccgtccgtgccggcggcgggcggggcggggcggcgacacggcgcgacggcgacgcgcagggccgcccccccggccgggtggcagaacctctacgccccgaatgagttaatcacccagttctcttaagctcctgagcaaggcaggtgactgattggttgccttgatgcttgaataactgagcagtattcgagggatcgggcctccattgctttttgacctacactacaagagaatgtgctgagaaagccagggaggagcttgctgacagtgttagtcatcaaatgtttggttgcaaccatagggtgatgtggggttgacgtccagggtctcgaccacagcacagcagggctgtgagcagagtgaatagtgaaggatcctgctcacacatttgctgtattcatccccacctcatgtgcccctgttttctgggttaaccacccccttttcttgtaacactgtcaatcctgtatagatacactcctttaccttctggtctacgtgtagatttgttgtctcactagaggttcataatccaaagaatttttgttctttctgctgtgatagtatattgttgtgtttctttaatggttatagcatattattacttcctgcaaaa

>TaeST2.37621.1

cctcccaaacccacgcgattcagaaccctcgtccaagggctcagggaagcagcgtcccagcgcacgccgccaccaccgaggggaggagccccgccggtgagaggaggagccccaacaccgccgcccgactctgcctgccgccccgactctccccggtggttctcatgcaccaaaagaagcaacaataaacaagctggatgaataaatttttctcaggttctcaatgctagatgaccatgacaccaaaagatggatacatgtttttgcattatcttcgttgcaaaatgatgttgtggctgtgatgaactgtctactgcgaaagattatgttgttatgataaataatgaatgcttggatttgggattcaaagtcatgttgttatgataaattgtgttattgctgtgatgagatattgatttcaagtcatgcaaaattatgttgttgttgggaaaactgttgctaaattatgagatattgatttcaagtcatttttatcaggtgaccttta

>TaeST2.37662.1

acaaatcacacaacaatcgacaactctctccttaaacactgcaactagttaagatgaggactacacatgtgttgtttctggccctcgccttactcatgctagcatcagatgtggtaacagaggcgtcaatggatgatagcgttaaaatcttgaggtgtgcggcaaccagtataccatcaccacagccatgtgacaacttcacttgcgaactggcatgcttcaaccttataaagctgcccaaacaaggaaagtgtactgatttccaatgtaagtgcatgatatgcgaagacacctctccactataatgacaagtgggtggaaactagctaaggtaggatgacatgttcaatggaatgatgcgagcaaatcgaaaatggtatagcagcatggttgttcactaataatcgacatattatgaagaatgatggatttgatctagcttcttgaatgatctaaaattatgaagctattgctacataatgtttatgtgtatttcttcgggcatattatgttagaatatttgggcttggcccaatagct

>TaeST2.38245.1

tagtctgtcagacaacctctgagccaccctaaaccagtgttaacaggcaggaacgatcctctacagccgtcggatggcacccatcaggtgaatccaggccgttaatcacgctctgactcagaggtcagaatgcatcaaccagactgaagtgcttgcctccttattggtaaaggatgcaacggtacgagcctatagaacagatcctgttc

>TaeST2.38267.1

ggggtagcactgccgatagcactcgcagtcgacgtcggacagcggcccctgcttggatgtcttctggtgggatggctttggcagcatgaggaggaggagcaagcacatggcagcaacggcggccgctttcttcttggcctccatctcctggcctcacaatcgtactggataagtgaactactcctgtggtgagtggtggg

>TaeST2.38524.1

tgaaattttggttcaaaatgacctgctcttcaatccttgctgatttgtggttttgagacccacccaagcagggtctgcgctcatgaggagaacctgctgttcctggaggcctggcagacagtcgatcccgctactaccacaagtccgctttgtgacgagctggtcaacacacgggaggagacatatatgattccacttgtgcaaagtctgcctatgttgcaaccttgggcgatcggttctttggattcttgaaaccaatgccttttctgaagtaagtttggcatgcaaggtgccctcacggtttattttcaagatggcgctggggtctcggggacgtgccggacggcgtcgcggtcctgcaccttggggatggctttgggtcgaattctcctccatgcaatcttgtgctcctttgggtgtttctaatctttctgccttctagcataaatagagagatgttctcccattgctattggctgtcaaatttgcgatgcaatccgtccctatgttggcacaatcagcagaacaaagcagctatcatgatttgcccattgatctcttggagtgattcaaacctgcaagcaccagggtaatgactcatgctctactatgtcagattgagtgacttctgcatatgatgactgaagaatttttcatcaattttccaacctgaattagctacagaatttgggaaaagaggagtccaggctctgatgaacacaccgtctttcccagaaaccacttcaactagcccaggactgtgtctattagtcccccagtatttgttgagatgagataacacattgtatgacaggtaacaagcaactgaagcaagaagattttttaaatttcttggaaagtataaccatcttaacccaggatgcatgatgaaggatccaatatgtcaggaccaacgagggaattgcagtatgcagaaccaatgagagaattgtagagcttcatattaagtttattttagctgcttgcccgcttcaagtgtctagattacatgaaacatgtttctttaagtgacaaacctggataccaatttattcaaactattatatttcttatagaagtaatgcaatatattttctctcttgtgcctatttgaaataccgttgtgggtctgtactttgcatctcatattgcaaggataacacattgtatgcatactctctctcttaactgtaacaatgatggagcaggaaagagtgatgcacc

>TaeST2.38764.1

ctaccatcgacacaggaaggaaaggaatatagttaactgtttctcatggctcctaccagtagcaacactcgggtcttgtgtctggtggctctcctggtcatgtctaccaccttcttgtcctgtgatgcagcaggtgggtggacgaagttttgcgtacccgagaaagcctgcaagaccccagcaggacttcacggcgactatgtgtgcaaggttgactgcggacgccaaggttatgatgaggataaaagccactgcgcgagtggcatttgctgctgtgaaaagtaatataagccgtcgatatgttttgttatgtactggaacgattttgcgtagggagggagactttttttctatgcacacctaggttcagttcatacagaaacaatcagtaaactgtaacggttgtattcttgagacaaattctgcaatggttgatctgtatgtcttccttcttttgtgaaagatttatttgttttccctagccctccaatcaaaagaaaa

>TaeST2.39078.1

ccaaagccttcctctggtcttcccaacagaggttgctgccgtcgctgctgctggtggtggttgccgccgccgtgagccgcgaggttgctgctctccccgacgcatctcctctccgtggtcgttgccgagcaggggtggctgctgggcgtcgtgtcgctagcagtgttggtacttctggttcaggatcctttaacccacaagttgtgccccgcggttgaggtaaatcctgaggacgacatgcatggaagtcttgggagtagcatcttcaccgttttcacttcacggaactcacacctattgtcctttaggaccgtgttttgagaacctagtatagttgtactttactacctgtggtggttcatcttgaattggagagataccctcttggtctgtatggcttagctaatttccccttaagactaacgctatcgcctaaagttaccgaggatgttctcggttatggtattgtagccttgtgctttacaccggtgctagttctttgcgatatatatactatatgaattgca

>TaeST2.39287.1

agcaatgggacacgaacacatatattactatcatacaagtaacaaaggggaagaaagaatgctatgacagagacagagacagaaatatatcacaacacaaacatatatattttcacatgaacgtaattaacacagggactacagaacacacataagcacacatatgcgaacagggtactacaccatgattcaggttacacaagtaaccaattaaacaagccacttgactagtatcacagatcaacagagtagtattgaacaactaaaaatagacagtagaagtaactagtacaggaagcaagtactcttagaatctgaatgttacagactagacgtctcaaactacagaaaattcagagaagaaaagggaaaagagagatggcagcaggagattagagaaagaagaagcagcaactggggcatggagaaaggagcagcagcagtacgggattcagagaagaacagcagcaaggttaggagaaggagaagtgcagcagtgaggtaaggagaagagtagtagcagcagattgagaagaggcaagggaaggaggaggagcagctggttcgagttgagaagtggaggagcaacagcaaggcgtaggagaagaagaggagcagcaggggcgcgaggtgcggggaagcgttgtggtgctggtagcggcggtgtggatggatgatggtggattcggggaaggagaagaagatggtggcgctggtggatggcgcaaggtggtgatgtggtgggaggtgctggtggcgcgccgccacgccggcctggccgcggcggcggccggtggtggagaagggggtgtgagggagagagttga

>TaeST2.39393.2

ctccttccgttcctcctcccggcaatggcgtcgagcctccgctccttctcccgtcccgtcgcagccgccttcctccgctcagcttccgcccggagccccgcggcctctctcccccgcgccctcgctccaacccccagggcttcttccctgaggcggcaggtggcgctggcacgatctctgcagccgctgcacagcgcaatctcggcggcgaggctaacgtcacggcttggggcagaggtggcccgggcagtgtcgcaggaaactggtatgagtgtgcctcgataatgaaagaggttgggacagacgcagcggacttttcaagaaatttattcccttcaataatgtgttttgcttgttggcattctttcgcacattaatatgctatgtactgctcagaagcaattactatgtgcacttcatatcagctcgagccttaactgaacggagttatacttgttaacgatctagttatgggaagaactgctgctc

>TaeST2.39458.1

taaaaccagccgttccggactttctctaggcaaacgtaattaacgcaacagaacaaagatttggagggaaataaatatgtcttgcccgccaaaagcattggccgtgaacttatcccgcacatagagtacgaaacaaaccaaactatacacttcatcaaatctatctacgaacacagatccaacaaagggacttgctggaggaacttgatggcttcttcaccaattcttgccggaaccaatgaggaatcaaatcccccaaatgtaagccatcttgtttccattgcgttcttcctttttctctgacccaacagtcttctcccaatc

>TaeST2.39701.1

tactgcacgatcgagccaaacaaaatgcacgcacacattctgtttttgagccactatctatggtcgctgatgatgttcatggggcacaatacacccatcaattgttttacacaaccacacatacatacatacacagaaaatcagcactagcacctgccagagctgcaagagaggatcatcagagctgtatttggggaccaaccgagctgcacaaaatcagaaacctgcaaatgtagctaacgatgacacaaatctgtaaaatcaacagaaaaactagtaactggtcgcccaaccgcaccgcaccatgagcacaagcaaactgcagtgctgtgcactagcaaaatctgaagaattttctttctgaactgcaatagaaccagcacgccgacgcctgcaaacgaatttggaacatgcaagagctgggctcgtgcaagtgtactgcatagctggccggagtcaatgatccccacggcaacgctgctggagctactcatcaaggggaaggaccccttttgtgtgacccctcacccgcaagcca

>TaeST2.39735.1

tggaaaaaaattagcattgtgatgtttgagcaatacttgagcaatactggtcgttcagctagaatcataatgaaaacgaaagtaaataaatacactactggcagtattataggagcacttatttaggacttattaagctaccattagtcatgcaaatcagtacgcagttgcgctattaccacctctactatactctagaggtgcttacagaatccagcagagcaggggcaattaggtccggatgctttgttgttctcatctgaaataacattctgtctcttctgcactaaactcagcagtcaaaaagtctttatcctctatatactccctccgtcccaaaataagcgtctcaactttgcactaactttagtacaaagttgtactaaggttgagacacttattttgggacggaggagtacttaacaccaggaagtggttcaccaatgtccctctccaacaccaaaaggacaatgtctttggatcctcggatctgacatgtccctatgaagaaatcatctccctctggcaaaatcatctccctctggagaaatcatctctcagcgtaggtctcgaacattttgttgaac

>TaeST2.39777.1

tttctttgcaagtaaaataaaatctcataaacgataccaattcttgatttacaaattctccacatgcatccatacatacatgagtacatagagcgagtaaaaggatagcacacttggaacaaacagcagcaagcaaacatggcgaactccggaacacgtcgacgtacgaatggacggtcacatcgacatatgcacaagccgaggaacggtagcagctgatgatgcaaagaccggagacactgaacaggacaaaggtgttggttcgagcaaccatagcgcatttgctggaggcagccttcttcttcgcacatatctacacttcagtccggccggagcgaccggtgacttagttgctcctgtaggacccaagcttggcggcgctggccctcagcacgtactggtggtaggcggcggcgatggcggcgcc

>TaeST2.39978.1

gatctgaagatcggaagtgacgcccccgatttaatcgtacattaatcatacacgcaaatgtgtacgatcaagatcagggactcacgggaagatatcacaacacaactctagacaccaaatgaaataatacaagttttatattacaagccaggggcctcgagggctcgaatacatagctcgatacacaagagatcaacggaagcaacaatatctgagtacagacataagttaaacaaagttttccttaagaaggctagcacaaaagcaacacgattgaagaggcaaggcctcctgcctgggacctcctaactactcctggtcatcggcggtctccacgtagcagtatccgtcggcggtggcatctggctccaaggatccaccatctggttgcatcaaccggaaagaagaaagaagggagaaaggtgtagcaaagcaaccgtga

>TaeST2.40020.1

cgatctctcttcgcctctccgtcccccccaggtctcctcctctgctcgtctccgcatttagtacttcggttgatggcctcgcctgcagcagcagcagcagcagctgagttcactcgggaaactgttcgccggtcgctaatcgcaatctctcagtccctccctgagacttcgctcctgaaccccaaggccaacccaccaagccccacccctggggcaaacgcatatcgcgacgatggggcggcgaagtgcagatcgaagctgatctcgatctcgtacccgtccactcctgatgccctatccacgccgtgccccccgaagaacgctgcaaccgtgttatcactgacgcctaattaagcgtgcatacataagtgtttttcttcttctgaagttcttcacccttctgctttcttggtctgggtgtggaatggatgtactccggtgcttgtgtttcctgtattcaaactgaaacatttgggaaggaaatggaaacaattgtacagggaactgtggtgtgatacacgaactagctgctgtaaacgagtttgtggttccaattt

>TaeST2.40144.1

gatcatactgaacgcgttacatttgctttgcaaaacaaatgacaatttattagacagtaggcagtccatcatcgattaaaacaagacagtgcatacaaataaagtagtcgccctcatcgatggtcacaatttattacatatgggcagacgatcgagtagaaagaaagctactcgtagtacgatcgagtagaaagaaagctacccgtagtaattaattagacagtgggcagaatcgatccggtcggggcgatggccaccaacatctgccggaggaattccttggacggccacaacggcttgccatcttctcctaaagcttcgaagtcctccaacgagggcggcggcatgggcatcatcgccgccgacgccgacgctggcgccggctgaggcagatacatcagcagcaggctctcgcgatccccctcgagcggagcagggatggaagcacccctcatcctccgcgtcccctcgcgcacatccatggcttccctctcctctgctcgcctggcttgattcttcttctctcctgtggtttccctcttctaga

>TaeST2.40575.1

agccccctccgatacccccctttccacgcggttttcagccctcgaggggggactcgaggattcagaggagtcgccaccgccgccgcccgactcctcctcaagacgccgccgcccgccgcctttacgccgacgtatctcctccggcatacccctggctatggcagagagccgtgagatctttctcaattctgccgctgaagatgaagagagtgccgccatgtttcttcggagcgaaatggcagagttgccttgagggacctgacctcgcggaagacggatgaccactttgctgccacaagtcgcctccacaccgtgtagcccgcaaacatgtctcgaagcatttgccaatcaaattttatgttgcaatgaaccattgaccttcgttgatcgaacatcttgtatttttcatttggaccatggcatgtatgcatgtaacattttcatttgaaccgttgatagtacttgcaacagattatatgagcctttgtgttgttttgcgtaatctagttgcaccattgttgcaagtacatttcttgtatgagcttttgaagcttttgaaattctaaaaattgcatctttaattgtagtatactaattttgtaaa

>TaeST2.40597.1

accgaacataagtcacatttcctcatggcactcatcaagagaaacacaaggatggtcatgtacaacatagttgttgctttattggtcactgtgttcatgttctcaacgttgccttcctgccaagcgattcaacgacgcttatgcagagatttagcgggttgcactgatgtgtcgtgccagagtgactgcttagaaagaggctttcgcccaccttatcaattggaatgtgaaaccaaccaccatcatcttcagtgttgctgtagacagttcggttccatattaaaatgagagccacaaagtttctcagcttatgcaatgcaatgatgtatactgatgccaataattgaataaacttatattttctgataaacataggtcttcgaggcatcaatctcattggattgccatggaacattttgacgaaggaagactgaccacataggatgtgtttgttacacc

>TaeST2.40598.1

gcttggaagtcaaaaacaaagaccgaacataagtcacattttccctcatggcactcatcaagagaaacacaaggatggtcatgtacaacatagctgttgctttattggtcattttgatcatgttctcaacgttgcgatcctgccaagcaattcaacgacgcttatgcagagatttagcgggttgcactgatgtgtcgtgccagtgtgactgcttagaaagaggctttcgcccaccttatcaattggaatgtgaaaccaaccaccatcatcttcagtgctgctgtagacagttcggttccatattaaaatgagagccacaaagtttctcagcttatgcaatgcaatgctgtatactgatgccaataattgaataaacttatattttctgataaacataggtattagaggcatcgatctcattggattgccatggaacattttgatgaaggaagactaaccacataggatgtgtttgttacaccca

>TaeST2.40798.1

gctagacacacacaagcgatctgaatcaggaagagatctacttgtaagacagtcagcaacgcacatacatattcttgaatcggagcaaggcaaaagcttacggcgaagggaaactgggctgcgatgcttatgatgatcatcctcgcatgtaccacaatacattcacagtgtcgaacaagtggtcagatggatggcagcaagctccaaatgtgcatctgcccgcagatcccttcgcccagctgcccgaataatgtgtgttactgttgttcgcatgtagcgggagagccttgctttccgacgagaccgcagtgcgtggtcggctgtggctgccagccccctgcctccgcgctcccctgacgccttggcatggccatgggcaatgtgtttggattactgtgagtaatgctgtatgtcatgacaatcttgattttaaataaaaaaatgatgagatgtattcatcttacacaattgttcgaagtgttcatttcactaatgtttcttattt

>TaeST2.40923.1

cacacaacaatctgcaactcgctcctttagcacaaacactagttaaaatgaggactacccagatgttgcttctgtccctagtctttctcatgctagcatcagatgtggtagtggaggcatcaatggatgatagcatgaaactgatccacacatgtgtgcacaaaattctcccattgacaaagccatgtgatagcaaaacttgtgaaaaacaatgtgtgaaacgtacaaggcaaaccaaatctagcatgtgtgtccttgaggggtgccggtgctcgttatgcatagatccggctccaaccatccatgataatgaacaaatgggtaccaattagctaatataggatagtgtattaagaaaataaagtgaagaaatcacaaatggtgtagtatcgagtttgtacactcataagccatatgaagagtcatgaatttggtcttgcttcttgaattatctgaaattatgaagtagatacaaaaatttgtatgcttgaaaaatgtttttgttttttgtattaattgtgatgtgacttgcatgtggatggtgaatgagcgtcgttccctt

>TaeST2.40924.1

atcacacaacgatctgcaactcgctcctttagcacaaacactagttaaaatgaggactacccagatgttgcttctgtccctagtctttcttgtgctagcatcagatgtggtagttgaggcatcaatggatgatagcatgaaactctacacatgtgtgcaaaaaattctcccatcgacaaagccatgtgatagcaaaacttgcgaaagacaatgtgtgaaacgtacaaggcaaaccaaatctagcatgtgtgttcttgaggggtgccggtgcacattatgcatagatccggctccaaccatccatgataatgaacaaatgggtaccaattaactaatgtaggatagtgtgttaagtaaataatgtgacgaaatcacaaatggtgtagtataaagtttgtacactcataagccatatgaagagtcatgaatttcgtcttgcttcttgaattatctgaaattatgaagtagatataaaaatttgtatgcttgaaaaa

>TaeST2.40926.1

aaaacttacatatcacaccaagatcaacaactcgcgccttcagcacaaacgaccagttgaaatgaggaatacccatgtgttgcttatgtccttggtcttactcctgctagcatcagatgtggtagtggaggcatcaagatattctagcattaaagaattcgtatgtgtggagaaaattttcccacctcgaacatgtgataccaatgcttgcgaagagtcatgctacaaacgtacaaggcaatacaaatctggcgtgtgtgttgctgaggggtgcaagtgcaagttatgcatatatcagcctccggccatccataatgaacaaatgggttctaactagctaagataggattattttttgagcaaataatgtggcagaatcacaaatggtgtagaggaaaggtttttcactcatatgagccatatgaagagtgatgaatttagccttgcttcttgaattacatggaattaataaatggtttataaaatttgagaagtgctctataaaa

>TaeST2.41019.1

cgttccccacgcatgaaaccctagctgctttcgttccccacgcacgccgccgacccagagaggcgacctcacccttccctcccatggcgccactgccccaacccatagccgactccctctcccctccctccctctccactccctttcatggtaccagatctgaaggggacatggtctcttcgctcccggtggtccagggcgagtaggcagcctccgctccctccacttccggcgacaggtgcctccatctctgccaaccaacattcggtgaccaggaggtagccatctatggcggcaacaaaatcggtagtggagcaagatttcttatatatgggtgagggcgtcaatcaatgttggtagttgggacaaggaatctcatgactgcaatatatagctaccacgttggaccaggcgcgattatcttgagtatagctagacggaaaaacatctttcgaaggaagcaaacaaaaggactacactcacacggaaccatgaagccaacgatccacgtcaactagatgttttagatttcattttgtacaactgatgtatcatacttgcatcttttacatgtccttttgtgaacctgttgtatggtgtctccgtagttatgtttagaacagattgaaggcagactgaagtgtatgtatgtgacacctagttaatggaataaaaaaaatgtttggtgcacctgatgtgagcctgaagtatcgtgtcttgttgattatacttataacacatgaactctatgtatgtggcaattagtaccatatgttaattaaagcagtggttggctaaataaaa

>TaeST2.41197.1

ttgtgtttctggcgatttggccaggcaccattaagttataaagctttgctttgccctaaaaaaaaaaaccatccacaagagaggtctcaggttcaaagttcaataacaaagaaggaaagaaaaacaacaggatccaaataagtcttgctgggggcacagcacaacatcccctgaataaatcaacaccgcacactagatgcaagaagcttattcatccggaggattacatcgcacaagtaaccaaaccggttgtctccggtcaaactatgtaaaccaacaacagcactggccagggcgggtgcaaaacgtgaaatgctgctgtccccgcgccaacctgctgcacatgtcgtcgcaagcggaataactgcatgcttgacctatcaaaaaggagcaatatcgggtaccacctggaggagatgcagcaagatcatacttatgcgcgccatggcaggacggtaaggtacaggcagacaccattgcaaggaccaggaaagcaatagccatggtcctcatcttgttgttcgggttgacgagggccatgaggagaagaggcgtgactattgtgtttgttcttcttccttgtgtgctcct

>TaeST2.41191.1

gctccgacattcctctctctcgttctcgttccattccagagagtcgccgccccctctctcccccaccccagctgctccgccctcaccggcgacctactcctccgccgccggccaccaccccctccccctcccctcaccatctctcccttcccttggccccagatccaccaacccatggtgctgccggcggatccgagaccaagaccgcctcctccttgtggttgttgctcgagctgctgttagacggatccacaacggccacgcggcagatctgcagcgtcttcggctgatccacgcggatccacggtgccctgagccctccccaggtgattttctgcatcaccccttggcctcagcggttccaccaatctccggcgccgtctgctcgccatcccctcgatcctgctccgagctctgaaaccccacgcacacagacctgaaccctccaccctcctcgctcccaccgtggtcaaatggcccccgcggccgccgtcatgatgcgggcccacttcgctagctcctgctcgtcagccagccggcggctccgcgggagggcgtctcgcgtctcgctcgctccccaggcggcctcgccgccgcgcgttcatggggtcgatgctatggacccgagctttccacgctctgtagatcagtggagtctcgagtaggatagtattgagctcctctatggtcgcagagcagggatctggggcgcccatcaggggacgagtctgggcgaaggccggtggttcgttcgagcaagattgcattgaggcggacgctttgaatcgttgtagcttcacatcctctactgcttcctcgtctgcacggccttggtcacctatacacgcacgcaccaccgagctgcaggtaaatgcaagcaggtatccccttgttgtctggttaattatttagaattgttcctctgattaatacattcatatgggcgcatgtagtactgttgatggtatgaatggctcgggtagcagttgcatcttggtgggtttcaattcatttatagcttagaggtttcatgatagtgtaggcatgcatgccacatacatattcatcaggattcaggaccgagacatctgtgtgtgtttcagcagacagtctctatgattcgatgtttcttcatgctagtagcatagtgctccgtaatttttgttcctcattgttccgtgaaaaattgtgtttaccctctgttacttcttgtaggtagttgtagctttcatgtgatgggaatcaactagacaagatacttaacatttgattaattaatgtagcttgtgatcattttatggatgtttgctggtatggtgcatgaaaaaatgacatgtacgaagccaatgctttggatagatgcactttagatgctctactgt

>TaeST2.41217.1

aattggatcattttgtaacagtttccttgtgatggcacttcccaagaacaagctgagggttgtacactccatggcccttgtattcatgggtcttatgattttctccaatgttttctcatcatgtgatgcatttggagagcacgacatacagtgtaataaaattgaggattgcacaatcaccggatgcaagaatatgtgcaaagaatatggtcttgatccttattcagcttactgctcattagcaccgggagatgaaatgaaatgttgttgcaccccagttcccccattccatgaataacatagaaaatgatgtacacattatcttgagcaagctgaatatggaataagaattttagtccatctaattttgtga

>TaeST2.41288.1

cgccaaccagattaagaattagccatggctcgtaaggttttcaacgttgtggcatccatcctcatattggcgttcctcatgtcttgtgatgtggttcaaagcacttgccatgagtataccagcaaggtcacttgtcgtgactcttcgacatgtcatacgtggtgcaagtacaatggtgctgatgatggtcactgcagtaaaggttcatgtgtgtgcctggattgtgggtggcgcgtgatgggttcgacttgagttgttcctttgtatccaaaaatgtatttggtgacaagtggtgttcgtgctccttgaataaatgtgttacaataaatcaagcattgctttggtag

>TaeST2.41383.1

aagaacagtagcagaacaacaagaaagctagctactcaagtactcctaatggcgcttatcaagaatagcgggcaggctatgtgcctgctggctcttgttgtcatgactaccacttccctgccctctcttgtagaaggaaggatcatggatacaaagggcacgatggtcggcaagccgccgtgttttgttatggaacgttgcacactcgatctatgcgagaaacaatgtttagcacatggatatcataagggtcaaagcgactgcatatctttcacaaaaaaccaatgttgctgcaaccggatatgataagggtaaaaaacgaatgtcaaataagactattcgacgtgattgatgaagaagattcaataaaatttaaactttatctagtaactattggttgatgcataactgccaagtgtgagcaagtgaatcgttgtctgt

>TaeST2.41456.1

gtgaggagatcgatgtgccggatcaagcatgtgctgacggagtgggccattgcggaccctgacccacggaggaccgcggagatgaagcggatgatcaacgccatgtgatgaacccacgttgtcgccgtgctttttcagctgttcgtgagcggcattagcatctgtctaccactagaaagaatttgcttgcttgcttgccttcttgtgttgtgcgtctcccccggaaacggaaggtgtataagaagaagtgcagcagccctggggatctctactaggctcccaaacattcccgtggatgaacctccgatggaataaccacggccgcggccgtctgtctgtttttgcacactgtgatatgcatgactgttggtttcatttc

>TaeST2.41605.1

cttcttctctatcagggcagcatcaaccccctcttctctgtcggtgaggaactccagcagaggatggattgcgttttggagctcctcgatctagcctccccggctcagttgctgccccatgctccggatctggcgccccatgctccggatctggccagagcaggccttgcccccgaccaacaagggagtctgccaatggaggtcgccgcctccccgtcttgatccgtctgccgcctggacccggccttctcgccggtggtggtggtgttcgctcggtggactctgttccattttcttcgctccttcacgctttggcgattccttctgttccctttctctgctggtttcgatttctaatgtggcttgttgattctcatggcaactctcgtacacatataaattggtgagaatatatgtatatcagcgaggtgggacaatttaattgaagaacaaac

>TaeST2.41678.1

atttttgtgtctgtcagacaaccactgggccatcctgcacaggtgtgagctaggcaggaacgatcctctacagccgtcggacttacccatcaggtgaatcctggccgttaatcacgctctggcccagtggtcagaattcatcagaccagactgaagtgcttgcctccttattggtaaaggatgcagctgtacgagcctatagaacagatcctgttc

>TaeST2.42357.1

aaggcgagcaggagaaacccagcccagaaacctgccctcaccgtcgccgcggaagatgccgcagcagcagccgggggccggcgcccacgtcctggactccgtaccactcttcgtcgtcatcctcggggccgctcacgtcctagccctcgtgttctggatctacaagctggcttcccagaagcagccgacgcggagcaagacgcactgaccgcgcccgcggtcgcatctcacgatttcgtgctgtctgttttttctttttagggtagatcgccttcgtttcctccccagtgagtcctcagagatgatgcagctcttctggctggagggcaccaccaccgtcctataagttcttcaccaagatgtggcacatgttcggcaggattatctgttctgtagtctcctttctggcatgtatggacacatccctgatttcccgttttcgatgtccgaataatggagtatatctattagacataacagtgacagtaatttattcagtgggctatctcttgtcttgtctgtaaagatcttagaaataattgcttcaaagaatatagtatctccccgcagaaagagaagaaaagaaagaatatgggaatctaa

>TaeST2.42433.1

ctgcagtttcacatctgactcattcacgccgctctcctcgctcccctcctgctgctgcgacaactgcggatctcgcagcccgacgcctctgtcgacatcgccgtcggtcatcctcgccgctgcggagggctaggagctactcattcgtgcagatgtcacaggcttgccgctctcgttgggtcattcaccacctctccaagctcaatggtggagtgcccaaagatggagtgcccaaggatggagtgcccaaggatggagcgcccatggatgaagcgcccaaggatgatgcacccatgggtggagtgtgagcaaatttgtgccaattcacctatccaagttgaccatattttattatatttggtcaaaaaaaccatgcccctatatatgtgatgtatggaatgtaacatggaccttatgttatgttctgtagtatggatctaatatgtgaactctatctaatatgtgaactctaagatatactggactgtaatatggaccatatggatttgtggctctatctaatacgtgaactctgatgttggtg

>TaeST2.42551.1

ggggagaggccaaggcgcggtggtggacaccgccatccctctcagatccggggctgagagagggagggagggagggaggtgggtcacctacgagagagagcactcacgccctcgctcgtcagtcgtcacccaggggccgccaccgccgctgccatgccatgggcttcgtccgatctgcgctcgccgattcgtttgaagaaggatctggccattgactgctggtgcttcccctccccgggcgatcagccatgacttgggatagtacttctatccagcaagcagcattttggatgtgacctccagctcaagcagagcatcaaaaataggggctctatctgctaatggggtagccggcaatgcagtctaagagctacatctcgtgtgatttctggatatgagaacttgaagaagaacaagcttgatgctattattgaaaaaaaatctatgctacatgtattcctttaagtactgttgccagaaaccttaattagctactgttttttatgaaatggatgtgatttatttgtgaactttagagcttttgagttgtaatttgtgatgtttgaacccttgccagactatcaaattatgctatgtgtgcctgaaggtcaatacaagggtattcatc

>TaeST2.42599.1

ggcatgtacaatgcatagcctcaaggtgatggctcgcatgccatgtaggatcggatatgacgtaaagtaggttcggataggaaagcgggatcctctctaggaggcgggtgcttgaacagaaaaagtgtggtccggtgacaaaagctgaaaaggttgagtgaaaagtagagatgcatatgtaatagactctttatttttctatttcttaatgaggcccactagtgatagcttacattgaagagaaaaagttaatatagatgcctcaaattactttttgtcatgaggcatatgtatccatatgccaccattgtacatgccct

>TaeST2.42800.1

aaaccgcgtctccgcttctcgagcccttcccacaaaccgcgtctccgcttctcgatccgcgtcttcggagaagagagagagagagagagagagagagagagagaggcagggcagccagaggacgcacgcctgcccggatcataaccaagattcaaatttgcccgctcgtcaatcatggttcttgattcattatcatcgcctcacaggaggtcgcaaaacacattctttctgtcatctcctaagaagcctcaatcatctcgtgatgatgttggtagttggtctgccctggttgagcggcatcgctttctcttgacaacgctagtggtgcttgccttcctgtgcaccatatatctttacttcgcggtgaccttgggggcaccacaagcttgctcagggttgacgggcgatgaaatgaccgtatgtcaggaaaaatcagccctgcaacatggaaagttgaaatatggctaactccgcttgacagtttacaatgtgcttgtagtccaaattagtatattctttagtggtggatgctgaaagggcttgtattgatgctgatgtacattttaatggagtgatagaggggattgtttgtgctctgaaaccgtataactgaataggtcccattttattattgtcatggtgtactaaaaatatcatgacactaattcaacctggatatatatatagagcatggatttgctatcca

>TaeST2.42995.1

ctccatcgcaggaaacgaccacacgatggcgtctttctgccgctccgccgccgccgcggccaggtcggctgcgctccggtccaagtcccggatcgcgagcccgttcccggcaacgaggtctcccgttgccgctcctcgcctccgcaggtccacggtgaagatgctggcgggcgcggagtcgctgatgccgctccacagcgcggtggctggcgcgcggctacggtcgtgcatcgccgccgactcctcctgctggagctgcctctcccaagactttgctcttcctcggtgacgaaggctgaagaggtgggcatcctactaggaacagcactcaggatggatcttatttatatgctagatgctcggcttggcgagtccatggctacagagatactgaactttgtttggtgactcggtgttcgggtgcgttctttaactagtagggttctggtggttgaaatgtccttttctctgtatcaactgttggtcttgtgcctaatctgaacatgagtttcgttcttcatgaagacaaaagttgaagtttctccttcatcc

>TaeST2.42995.2

ctccatcgcaggaaacgaccacacgatggcgtctttctgccgctccgccgccgccgcggccaggtcggctgcgctccggtccaagtcccggatcgcgagcccgttcccggcaacgaggtctcccgttgccgctcctcgcctccgcaggtccacggtgaagatgctggcgggcgcggagtcgctgatgccgctccacagcgcggtggctggcgcgcggctacggtcgtgcatcgccgccgactcctcctgctggagctgcctctcccaaggtttgatcaagcgcatctgacattggctaggagcgtaatgttgaagagcatggatcacttgctcctgtgtcattttcaagatattttctttcttctctgaagaataggatatgtcagtgtgcattaatcaaattttgttgaaaaagttgcagatctaatctgttggtgactcgttcacaactattgtttaatttcactctatgcaataacagagttaccgcagttccttaa

>TaeST2.43026.1

tgcgatgaactaacggaacatgtaatctgttccacaaatccaactcgtctttccataattacaactactacgtacgtacagagacgaattgatcaggtggtttgtgaagctatgtgcatacaagtatttttctttctttcgcgaattcatatgtactacgtatttatgtacacaagactgcttgcacaccatgggtggctagcctgccaatgccaagtaggacgcggacgctggtgaccacgtccaccgaccggcgacgcagccgtccaggatggcacggccgatgccggcgtcggcgtccccgccgctggtatccctaccagtggcttcatgcagagcgcgcaactcgctggcgagcatccgcacggcgacccgctggccgggcaccggcagcgccatcggcataatccacgggcacggatgcaccgccgcgctcctcggcctcgccgccatgtcgtccttggacgcgcagctccccatgtgatgtatatctacctcttcacggaacaaacaaggaaagt

>TaeST2.43117.1

gaggtaatcttcacacaacccatgtaatacataagggaaagaggtacatagttggcttacaatcgccacttcacacaatacatgaataaagcattacattcatccaaatacactcaaggtccgactacggaaccaaaataaaataagactaccccaaatgctacacagatccccgatcgacccaactgggctccactactgatcgactgaaacgaaacaacacaaaggacagatcttcatcgagctcctccttgagctcggttgcgtcacctgcacggtatcatcggcacctgcaaactg

>TaeST2.43153.1

acgttccctcaacaaccagccaaactcctcctctctctccgtcatctcagatccagccgccccttccatctcctcttccacaaccagccaccgtcccgatctgaaggtctccgaccagatcagcctcctccccaccacggcgtgcggcgaggagtccccggatcgctcgccgtcacccagtgcggtgcatatctggtagtccgccctcaccaagcattcgatgcgttgacggctcctagcaccagataccgcgacgaacgagctgtgttctgcctcctccggctgccgaggaccacggacgccacttcgtctcaccggctggctcaacatcagcaccgaccactactcacagtgtgcaaaattgttgctacgtcatggatctagtagcacatggatctatcacttttgctcttggtcttgtgttctccagtgcgtgatgaatttgatgatgatgaaatggaggctggacgacgtctgccacacgagttaacatgccaggctcctgacttactttagttcatgtcttcatgaccagcaagttgtcctggttcctgcatagattgtaattttgtaggcagaattgtacaagaacttttcggatgtcccactcgtactttgcagaattttgacgaaggacttgtatgttttaacagaactctgttttacagagtagacactctgaataaattgctgacgaactttatttttgta

>TaeST2.43162.1

tgactttgtccgtttcccttccctatttcgagcagcgagatcttggaggcagagaggggagagccatggcgattttggctggttagctttgtcgttgatctctggagagcgagccggtggttaggcgtccggtcctcctcccccctccccctgttgcttctccttcaagcgcgttgaggcagaggagaagttgacgcatggtgattcagcggcggcgtcttatctgcttcccttgatttggtgatgctcctccttttgttgtggtgttctccgagccttgtgcgtcccttcttcccacaacgaggttgcaagtctcttgctcttgactctacaaagaaagcatcaaatgattcgtcagttggtaaatctgctggcatgattgtggttttctccaatgtagacattcgaaatcttaggatcaaatatgtgaatgacatgtttttcagccccttgaacagctgcgacaaatcttttgtcactgctttctttggaggcaatgccaagtgaggttcagcatgtgcacagtcctagctcagttggtggatgagttctctttttgagtagcatgtaacatagaaccactagtgcaaatcatttggatcattggtggatttacaaggtctttttagtggatttaaagtggcatctttaatgcatcttgcactgtaattcttagtggttaatcttagcagttttttcagtgcaaaccatttggattgttggtagatttacagtgtcattttagtggattttacagtgtcattttaatgcatcttacactgtaattcttagcgggcatgataaaaattattgtcgattttagtggattttacaccaaatttcttggtggtaatattattgtaattatacttctttgtcaatgtaaattttaggtgacatcatcttggtcctttcctca

>TaeST2.43173.1

gagataaggatagatactaccggacactcccgctgcacggcccgtgactctggtccatatgcgttcccttttgcagtagccatccccaggagcatgattggcggcaggagaggcggattcatctgagatagaacatgaacagtacctaaatctgagcagaggcgcgagtggctgggacaagaggaggcgcgcatccagccgcggttccgggccggcggcactggcacgagtttcgccgccggtgcttatccagcgccggggcacagcgcgcaacactgtcggtgacgccctgcgccgtcgtgtattctccggatccaccacgggttgatgaaggatgtttggcgctatatttatctttatgacattttacatctcaagggaaacaatgcaaagaggccgcatcaatttatcgagtctatagatgaatgaatgaatgagagtgaagaggcgggtaacgcaatggtgaaaaaccaagaagtatcttggggcacaacttgtatggggtgattgttttgggtgcaagcgttatatgatattgagagctgcgatagtgcgatatggctacgaagactagttaattctgttgtcaacccatgtagcgaacttaatatcgggtacatttgcatttctcagacataaccatttatcgcatttttcgttagggaggtcaattgatgaatgtccgagctgtgatttacaaatttaaaatatttttataattggttcctaaa

>TaeST2.43256.1

gagtaagaagtttgtgtcttctcttatggaagggaaagctattcttatttgccttatggtgcttgtgcacctaggaaattccattcatattgataaatgcaaggatctagttgctactatcggtcgtgagtgcgatctgttaaaatgccaggggacctgcaaagatttgtttggagcggaactcatagcctcatggtgtctcaacgccgtcccatactattggcattgttggtgtcgtgtctgttggtagagttgcactgaacaatcatgtcgaaatggacaaatgatgtgatccaataataaaatcttcctgtgacatgcatctaagaatacataatgattgccaaatctaggtgtt

>TaeST2.43386.1

ttttaagcactgaaggaatatatatattataatgacacaaagatataacagtatatggaaaacacccttgcatacatcttctccttcagctacaaaggataccacctcggatctgtcatcgttattggactgaatgaggaaacagttaagcatacaggaattatctagaagaattaatggagcgacatgtaggatcacatactaataaacttggtcagtgtcatctacactcggtcattcagtctcccctcattagattttctaaaagagagcctcctctctgaattctattaacacaaaccaattgttcatctacatcataaagaaaactaagaagggaagaagaaacagaaataggaacattcaggtaaccccagagggcacccaaaacccacagatatctaggatctggagtttgccctttcagcggctgcgcaacagagacaaaatatttcattgctactccctccgttccaaaatagatgactcaactttgtaactttgtactaaagttaatacaaagtagagtcatctattttggaacgaagggagtagatgagagtgaacatactcgcaaaagcagtcaccaacaagcaggaacagtccacaaatggcaagagtggctttaaaagtttgatttctctgtgcactttaattgcttttaagctgccagtcaaacgacttcaggtcaccatctttgagatgctcagaagacttgatttttgtcacttgctcgaagagctttactccccaaatcagcgctgatctgcaaaattgaccatgagtttaaccaaataacaaaccacatgaatcagaacagaagtatcagacaccgcttttattctcaaaacaggcctcattcataaatctccaaatcttccataatacagcagccaaccccactgcggtacaaaaaattgtcctggtgttttcaatctaagccctcagaacctaaagaagtcatgatcagtatcaggtacaatttttcagattaaaagtgagctgcaaaatggttcggatcaactaagtatcccatctccgccatgtaactaacatgctttattcctgttccacatttgcaaagtatctccccaaagggtccttgtaaatcgctctgattcccatatgattttagttttaatgtagctgatttgaacattaacaaaatagtcaccgaaataagtttgacatattgaacagaaatatagtttgtggataatatgcttgggacggattcgataagagaacaacaatcaaataatatgacttgtaccactcgcacttaaaactgactaatagaaaggaaaccaggaataattttccgacaatgtgatcatcagcgattcaacatcaaacacagctaccctacagtttgcgctcagtatgatcaggacgaattcgacatgacaaccacaattgaacatgacgacttgcaggattcacacataaactacataacagataggaagccaacttaataaactgatatacagttctctagccacattatcaggatgagctgaatcagaagttgagaacaaccacaattgatcacatttcagttgcaaccttgaagcttaaactgctcaacagaaacaaaacaacctgatattagtagctcagaagaagccaacagtagtagactaacgatgatggatttgtccattttgcatcgcatcaccatcaagagccacatcagagatcagtgagtccttgctagcgataacaatctgtcacatc

>TaeST2.43527.1

tatatccaacacatattaccaatagtagcaagtttcatccatacatatacatagttccatcaatattacacagtttcatccataggtagcaagtttcacaaagtcaaaactatcttattattaagatcttctctggatcttcttcttttcttccaacctgcataacaaaaacactaagaaagagagaatgagttaggagtagaaatgtagcatttcacttggtgaaatgcaatgttgtaggagccagcacttcagatcaagacaatcttaaattagtgtaaagttatgacagatccatgcatgtgtacataaagcaattaaccatagttgtacgtcctagatactctagcaaatactcctgctctattgcaatacagaagaacattgtgctgctgtagtaagtagaggagtaggaacaacatgctgcctgtaggaaactgatccaaggacgcacctggtggccgatgggcttggctggcgctactccggcgaggggcgtcactctggcaaggaggaagcccgtgcagcgagtaggaggcccgtgcggcgaggaggatgctactcccaagactcccatgcatgtcgtcgtcaagatttacctaaaacatataa

>TaeST2.43558.1

tcacaatttaatagattgtctgaattaatccatagcaatttccttgcatgtattcaggctaccttaacacacgctgaatcagacttacaaattcagacttagatgttagtaaagacttggcagtacaaacattacctactgctgttttattaagatggatataatcctactaactacagtagagggatgtgagtacagacttggcactcggcagtatagaaattccctgaacaactacacacgctgaatcaggtattctagatcctgaacacaaaacataaattatctaatgctgcaaatttgagctgaagaaattgcaattgctgaagattgaactgaaaccagaaatttataccatgtagggtttgcagtagaggaatctatgagagaggggtacaggtgcggcggtgtcgacggactggtggacgagctagtcggagcgccggcgtagaagagcggctgtggcgagcggctggcgtcacgagcctgccgggacgcgctgaccgggctggtgccgccttcttacaggcaccagacttggccatgattccggtggcttcgccccgcccgccagagccgcctccggccggcgagagggagtgcgggacagggggatcgaggcggcaggggagggagaggatgaaggtgcagccgtggagaaggg

>TaeST2.43723.1

caaagaacatgcattcggcaaaatcaacgaacaacattagaagaaaaggaatagctaactatctcccatggcacgtatgcagaactacacacgggccctatgcttcgtagctcttgtagccatggccaccactgtcttctcagatcatgcagcagcgcgttacatagatactgacctgtgtacacctgtggagtattgttcgccaccagtccgagctagtggcaacattgcgtgcaaggctaactgtgttaaccgaggttatgacaaggataagagcagttgccttgaaggcagtagtggcacatgctgttgcggaaagaactgagtaccatgaaatgagtttatatatgtactagaaatatttgcatgaaggtgaatgcttatattgtttgtgtgaaacctgattcagttcatacaaagcaattaataataagctattgataattattgttttagtat

>TaeST2.43826.1

ccccatcgtcttcctgccccgatcccagcccactagccgctcctagccaattgccccacccccgcgcctcctcgttgctgtgccgccggcgatgtgcggcgctcccggccacgcgccgctagcctcttcccggccacgcgccccggcgactcctccctactccactccccttgagtcgcgccgcccccgaccacgttgggactgggacacgtcacctcccagctccgtcccagtgaccagtgcaactcctccttcatgtggcctgttggtcgagggtctgataatggacataacacagctactggagaggagattaaaatatggaagatagcaacagagggggaagctgaggctggtagatccactgagcaagagtcccagacccaggtttttagtagatgaaggagaaagtccgcgccaagaagaccattctgatggattcaagagaatcaagagtgaagcatcaagatatgataaatatgtcgatgacaaatatgcctaccgagatactgagtggtttcagatatctttggttgatttggatttgctcacatagaaaaaggatggccacatatatatggtagttacttccatgtcacaaaggtgttcttcagtaggtactgaacaatcacgttgaactttcacaacaatcacattctcagtgttgttgtcatatcatatatctctatagtgtttgatcacaatttagacaattgatctgatgttgctcatctttcaatataatatcttctttttccagattta

>TaeST2.43925.1

cagcatggcctgccgcaagagcaccatcgtccttgctatctctgctatctttatgttgacgctcctcatgtcttgtgatactgttcaaggggagtgcaaaaggaaggaggtcgatactaaattaccatgtcgtaaccccaatatatgtcgtgagtggtgctattccggcctattcaccggcggcgggcactgccatattcagggtgatagatcattttgtgtgtgcgtgggttgtacgccacaaacataaaattgctcaaagcaatccagagcaaagacggagatcttgcaccggaaggagctatattcagggagatccgcgactttgcccgcttaaatttttcatcaagcatttcgttttctttcatccacgggcgtataataaagtactccctccatccgaaaatacttgttgaagaaatcgataaaaagaatgtctctagaattaagatatgtctagatacatgcatttctccgacaagtatttctgaacggagggagtagaacattgcgccttgcggcgtatggttcacggccccaggtcggccgccaagtttgggcggaaatcctgtttgacgatataaacgtttgaatgaccagctttgttgctgagagcctgtgtgatttatgaaatcggatgtgttccatctcaaaaaaagatggtagtttgtatcgatgaattttttggaaaataaacaaattgcatgtatctatca

>TaeST2.44158.1

accagctaattcttgaatcagcacacccatattgtgcctacaaaagcactgattggttaaagtaattcaattctgtgtgcatggagaagtacacggaacatgtgcgcctgctactaccactctcgatacttctcctcgtttgctttattgttcatgttcaatgtgggagaattgaagacataggtaacaggaagataaatctaccatatggattgtgtggaccacgtaaacaatttggaaaatgcaaggaccactgttggtgttgtttagtgagtccaacccccggcaaaaacatttgctacgggacacctgaaatatgtgatgagaattgccgagtttgagttacatgttgagactcttggattaatcgggaaaaaatatatagtcattaatctttgcggcaccgttctagtatatgcacaagacatataaaaactactccctccgtcccatattgtaagacgttttttgacactggtgtagtgtcaaaaaacgtcttacattacgggatagagggagtacttttcatgttgattgttagcatatcaatctccacaacttcggtctcatgtatctttagtcgctctcataaaccatcatgtttctatcttaattaatctcgtgtta

>TaeST2.44307.1

attgagaatgtggattgttactcaatggaggtcaagacatatggaattaccaaagtctgtacaaagacggagaagttcctcaatcgcagatctgctctcccatactttagcttcccaccactgttatcggaaccaactatctgatcaactgatctgagctcgctcgattgatattgcttgggccatacatctggagtaggatggcggatggtcaagatggtgctgatgtagtttgatatgtccgtattaagtaccgcatgtatgtaagcaggatatgctgtcagcaagttgaatttgaccaacactgacgtcgaagacttgggtttatgttgtgtactgatgtttgcgttggaagacctcggagtttatgttccccctttcaat

>TaeST2.44402.1

acatcaatctaaggccttgtttgattcataagtcctaagacttgtcccaactaaaaagtccctccctgtttgctttccagggactaaaaagtcgctagtccctccctggagttattaaatgaccatgttaccgctagtatacagaaaaataacaaccaaacaacaccatgagccggtggggcaatgggtgcagggaggggcattgttgaaaaagtctcaaaaagtcccaaaaaaagactctccttgagagtcttcttcatttagtcccaaaaagcaacttttagtccctagtagttcctcctgtttgattaaaaagtttctaagagggactttttctagtccctacaccaaaaagtccctggaaacaaacaccccctaagttgtctgcaagacctctataactgtaacaccatagcagagagagtaaaaaactcagcatggccaataatccatgctaatattttaaaattttccctaatcatctatctttagtcattgacagaaattagagggcggaaaagatagaaccaccctcacttaaatcacccaaggttgatgtgcgatgtgcctgtcaagagactccaaaataagtccagtgctagccttctctcctttgaccttcgcagccatcacgccagcttcaccatgcacctgtcctcctccacgcacccccttccatcacttcctcgacctcttcatcctttggcgtaggatcctttgtgttcgcttctttcttgaggcatcaaataatgtatatcgtcggacaagaattacaagatgtttagcacaaagtgtttgttcataattggcttagcaacaattaaaaaacactgcatcttcaataaatggtaaaatacttaagaagcaagacatttataagtaactttaccatttccttcaactgtcctttcttcaggagctcaggggctcactatctgggcggcaagcaggctaccacgcaaagattctggatgcaaaaagaaagtaacaatgtttattaattcagccatattcctagtatccacatattagtttgtgttaaaaaaaaagaatgcacaattcttatcactgccgttgtaccattccatcatggttgcaagctccttgtattcaatgtttttgtcaatgtcctccttttacaaaaatatccttaaaatcatccctcccctcttaacattaaggagtgatcttgcaaatacaaaaggattaggatatgaacaccaattataaaatggtgactcgtagcaatgattttcataaaaaaggtcactgaacacaataaatatagggctatgctatgcacttataattaacagtttttgattggtacttataattaacagcagataataaaaacagtacgtaaagttaaaatatggaaaatagtactgaattgggtgtatgaatattgcaaagaacttatggggtatgaacatattattcggaggcataaggccatgtccaacgctcagctgcctgtttggacgcctaacgaataataataataattaattaaatcaattagcataggtctaggagtccccgcctctagtgttgcaaatttagtaggtgtcgtgtgttaacatagtagcaaccaacctgattatcgcttccatgaccaagttagccgtaccgaggaagtcctgatgtactgaatagcacagaagaactaaatacatcttcaaagaaatgacgaaggaaaaccccactaataacaaccaaagcagataattatctaaatattttatcatgagaagatgatcaaagccttttgctttatctga

>TaeST2.44438.1

tcgaaagcacaatctcactggacacacaaggtttgcggtacacagaaacaaccaagtcaatactccggcgcgcagacgggattcagtcagcagacaggaaacaggcttcatggcaaacaagtgtcgagcctgcacgccgaaccaagaaactcatagcgcaatcaccggacagacaggttttccaaggcacaggaacagacaagatcgcagctctacctgatatatggcccgtcgcgtctggattcatcaggttagggcaaaacagccgctttgcatcaggcataagtggggcgacacccgacacggccctgctactagttcagggtcatggttgactggggaggaaccatgagaatgagaaggcaagctagacacactgtctagttatgataggcactcttcagtt

>TaeST2.44487.1

cctcctcgtcttgccgccacaaggtaagccactccacgccatatccgtcgccaacgccatgcccaagctctactctggcgcgatcaagctcgtcatcgtcgtcttcttcctcggggagtgacgcacgcgtccccgactttgtccccgtctccgtccccgaccgattcttatgggtggctggactggatcgcctcccacgctgtcgtccacgtctttgtcttcttcctctacctcctctacgtcatgaacgccggctcctctgcttccccgtcgtcgacctcgtacgactccaacagcacccccacgttgcaggtacaggttatgcgatgatcatgacgcaagagcgatgcttgtttgcgttgagatcttcttctgctccttcgatcaggggataggttccaggtcggtagcctgggctagcagggtggatgtcgtttgagtttctgtttgtgtttcatccgtagtcggatgatcctctgatgtattgtgatgttgtattcatgtggcatcgattatgtaatgttgatgtaatgatatccaccttgcaaaagcgtttcaatatgcgggtctatccttggtgggaccttcgagttccttttggatagggtcgcatattgggcgtgacaagttggtaatcagagcctcgaccgaccataggagcccccttgattgttggccgttgttgagtctagaagaaaactattttgattcttaggattatatatatcggagagtaggattctt

>TaeST2.44515.1

ctctctaatcgaatcggtgactccacgacgccccccgctcggcgctcgctgctctctccccctttccctctgtctcggcgccgtcgcggccatcgatcgacctcgaaggaggcgcggcctgcagcctccaccttcgctcagtcaagtttctaaaacaatcacatgtccaaggcacgcaacgccttagttgtcactggcttggtaatatttgctggctctggcctagcatttcccttctactttgtgaaatcaaagaacaggcctattatcgactcaacgaagcctctgccaccgcaggctactttccgaggaccatatgtgaacactggatcgcgcgacatagggcctgatcacaccgagtaccccaagaagtgatatgtattttcatcttgttttctctttgtttttcaacgggcatgcagaaagccagtgagtggaaacctgtaatgttgataaccggctcgtgatgtgctgaacacatgcatctgtgcgtagttcttcatcaactgtaagcgcttttgatcatttcttgtgctccactgactactccttgcaaagcgctatgccgctgtaccttttttaggcaataataaggaaataaaatgtcaaagctact

>TaeST2.44599.1

acttcgcagcgccgcaccacggaagcacgccggccggcgagcaccaccagcatccaggcggcggctaacggcaagcggtgaacgtaggcagtttcaggatgggtactgaggactctaaagatatgctgaagaatgcggactggaaaacagtgagtggtccagtgattactgagtcaagccagccaattgttaagaagcgtcttccaaagaaaatcagacaagtccccgagtgttactttctgccccggcgatctctaccttctgcattggcaatctatggtgctgtttgtgccgctggagttggtgcaggaatgttgcttgagatttggataaacaaaaagatcaaagaggatggcggcgttgtctgggagatgggcaaatgatgctcatgcaccttgagttggtaacaatctgttttgggatacccactcggcttcatttatgctgcttaagctatactgcagaaataagcgtagatttgttgtttcagactatctgttgagattgtgacaaaagcaaacaatgttttcctgtgtaattgtcacaaccttgtccctgtgtttccgacttggttgtacaagc

>TaeST2.44858.1

atacttcagtttagatgaacaaaacagcaacaagcatcacatcatcgcccataaataatttacagacaagtttattgcaaggtgaaacatagatcaacttagatcacaatgactagttccagcttgataaatgacaagagtgccagcgaacagatttaggaaatctagaccagtgcattgatatcacacagaaagatatatatagatagataaaacaagagaggcagacggcgctatgatgcatcctcttcttccattgtaagttcctgaaaacctacttgacctcggcgactcctctgaaagcctcgatgcttgcggcgttcagcgcgcgttcagcgcggccctcctcttgtcaacgtcatcggggagcgcgacgaatcccttctcatccagctccttgcggactctttcctggtacttggcgtacctgtcctcgacctcctcgtgctgggcggccacctcgagcatgagttgctgctcctcgggggtcaggtcgtcgaagtagcccccaatgacgaaaggcaccctcttgtggtttaggaccgcctggacagacgcctccgacaggtggtacttgcacgaaggaaccgtcgccgccgcaggcttcttcgatccggagacggtgctgccgatcggacgcagctgagggcatccgggggggctcctcgccttcttgcggccctgcttaccagccatcgccaagaagattcgccggcgcaaagtggggatttgtggagggggcgtggagaagaacctacctttcaatcgatcggggtagggactagggaggccgccgtgttgcctcggctaataaaggcaggggcaaaccctaggagctatgatcccgatcggagttggattgaatactaccgtcgcgtccgtctccgagttccgccgtggatcggg

>TaeST2.44969.1

tcgacccacccgttcccacgcggttcagaatcatcgaggccccctagaggatttggctgctcgactcgagacgccgccgccgccgctgcgccgcctcctccccaagtcgccgcgccacccgacgcctcgacgccggtgatctcctccggcatactggacttctcactggtggttctcaagcaccaaaagaagcaacaataaacaagctggagggacaactttctctcagattctcaaagatagatgatcatgacaccaaaagatggatatatctgtatgcgaaattataactgatcttgagctattccagaagtacttgtgttgcgttatctttgctgcaaaattatgttggggctgtgatgaactgttgttgctgcgaaagatcatgttgtcatgataaattatgattcctgggatttgggattcgaagccatgttattatgataaattatgatgttgttgtgatgatatattgatttcgagtca

>TaeST2.45037.1

aggcgaggaggagaaacccagcccagaaaccttccctccccatcgccggagaagatgccgcagcagcagccggggaccggcgcccacgtcttggactccgtaccactcttcgtcgtcatcctcggcgccgctcacgtcctagccctcgtgttctggatctacaagctggcttcccagaagcagccgacgcggagcaagacgcactgaccgcgcccgcggtcgcatctcacgatttcgtgctgtttgttttttctttttatagggtagataatcgccttcgtttcctccccagtgagtcctcagagatgacacagctcttctggctggagggcagcaccaccgtcctataagctcttcaccaagatgtggcacatgttcggcaggattgtctatagtctcctttctggcatgtatgcacaccatccctgatttcccgttttggatgtccgaataatgaagtttatcttttagacataacagtgacagtaatttattcagtgggctatctcttgtcttgtctgtaaagatcctagaaataattgcttcaaagaatataatatctccctgcagaaagagaagaaaagaaagaatataatatctattctcgcggggcaaatgcacccggacaaacagtaaatgcataaaatagtgaaatcattcaaaaaaattacaacaaacatttttgagtgttc

>TaeST2.45146.1

ttatgcttgccctgctagactagaccaagggaagatataagagtaattatgtgtgctcaggcaggagctggctgttccatggaatgattgaaatatgtatgtgacccctgaatcaaatggcacgtactgagactgggatggaccctgatgttggttgtgcaacctctggtgaaccatatatgtctttttttccttttctttttatcaccagtttcagttctatctgcacctacattgtgatgtgtttcttttgctcccatgtgtacatgctgtagctgtggtgatgctgcattttcttcatccttatgttttcttctttgccggtatatatattggtatagtatgtggattcattcatcattgtttatggtgttgagtgagtcttgttgatcctgatgtgttttgtcaggtcacactacacatgcctgtgaggtgaagtgccagagctccacatgaaaatctgtaggaaccaggcctgtcatttttaataaatattacatcatcaaagctgttttatgcatgctactggtaactgtatattctctcctaacagttttattacttgttgtatgcaggctgattggagcctacaatcatgctattttttcctttaaacacctatttgttttggtatcaggtgctccttgagctgtgtaagaacaagcagaccatcatttcaccatcttctccaggatgctagccttcagtcttcagaagttggagctctaggaagaagaatcctgaagaaaacccacacccagcaatgcgttttagctcactggactagttatctatttatttagttgtattttagctaaatactagatttgtactattatctcactattaccagatttgtattttagctgaaaaccagatttgtactattaccagcaatgtgttttagctcactggactagttatatgtatatctgtatcattgtaatttactttgcttggatgttgtatgacatattatgccttattggctctttttgaatcaaattatctaattgcacaaa

>TaeST2.45334.1

agaagaatcatggatataggacagaattgatataactcacattacaaacacacacatatgtatatatatcacaggacaccacacacatatgaagaatactgtttcactaaacaaagtatgacaagtcgaatatttctcaaagtgacaaggtatcaactagtcaggacaaaggacatactaatcaagcatttatattaaacttcgaaactgaaccaaacagtaggatattacacttatcaaatctgaacattacatagtactgataattacacttctgaaggtcacagaaatttgagagagagagagagagagaacaacagcaagattgggaatggagcagcagcagggacgtgaggagaagcagcaggaggaggttgagagaagaagtagcagcaggttggggcaagggaaggtgaagagcagcagcagccagctagagaaggggcgtgagggatgagcagcagcaggtgaagagagaagaagagcagcaacagcgagattcaaagaagaagaaggggagcagaggaattggagtagcagcagtagggacgagggggaaagcagcagcagttggaggtgcgcgggagatcagacgccggcgaccccaatggctggtctagggcatgaatagcaaggggaagcagcagggagaggtggatgggtggtgtggcgctggtggatggaggtgatggtggtactgggtgctgttggtgtagtggatggaggctgctggcgcaccaccacaccggcctggccgtggcggcggccggaggtggagaaggagagatg

>TaeST2.45338.1

tatggaagaaaagaattaagatcaagggacaagaatagaaatacttcacatcacaagcatacatatactcacacgtacatagctaatacagggactaagaattaaagatgttcaaactactcacttgacagagagcataaacatcacaaaaatagacaagctaggttcttacaaatcactagttgatatagagcacatatattgaacagtactactagacaaatctgaacaacacatgtcatacaaatctcaattctgaacattacacagtactgactattacacttctgaaggtcacagaaatttgagagagaaagagagaagcagcaggtaggttcagagaagaaatgtagcagggggagtacagagacatagcagcaggagcttgggggtagcagcagcagcaagaggagaagcagaaaggggcgctggagagagagcagcttcagatcggcgcaagaagagcaacagcaggggggacgtgagaggaaggagtagcaacaaggagatggggaagaagcagcaggggcgcaacaaggaagaagcagcagcggggcttggggaagaggagcagcaacgggtcttagggaagaggagcagcagcgggtcggcgcagtgggagtcgcgcggggcatcagacgcctcgtctggtcgggcacgcgagcgtggggtggaggcgcggtggtgatgcggtggcgctagtggctggatggatggatggacggtgtgtactggtggtggcgtggagatggattggtgccggtgggtgctggtgatggcgcggtggcgcgccgccacgccgacctggccgcggtggcggccggtggtggagaaggtggggtgagggagatggagat

>TaeST2.45395.1

aacaataacatttctctatatctggtacaataataacatcatttatagtacatttggtcaagcaaccaattagcagaagaacaataacatttctctatatcttgaatgctacatatgggataattaatatatcgacttgtatggatggcttcactcaaaacaatacatatgccttctgtttatttgcctgacaaatggccatcagatgaatcaatcaaataggaggaatcagggagcatttcaaaccaaaacaaagaatttatcctaactactaaagtgcagcagctttaaagcctctgaattattttgctcccactccaccaagaaagtgcaggccttcaagaaccaaaagatttaagagtggaactaaaagaacagcagtccaaatcctcgtaagccatgttgtttatatccttaaacccaaaaggttgcctcttagcatctctgttgttctcttggtccttattaggtgtcagattatcctgaagaaaaggcagttgatatcaaattatcattaaataaatccatacttaacatgtagatcatggggaatggtgagatgtcatattggggttcgcaacatgaatagaagaccactaatttatagagggaaaaaatgcctgaacacccttaagaaagaccatgatggcgtactaaatatttcgaaacgcgaagttattttgtcgtcaaaagagaaaaattgatgaacaattagaagacatgtaatgaagaaaacatatggtagaatacgaactgcattattatataaatagaaagaaacaagactgtggtatataattattacgataaacacaactcttcatttaaatagagaagccatgcattactacatgcaatcacacagttaggcaagcaaataggctagtcacattcatgttaagtgatcacatggtatcaatatagcagcgatatgtagaaagctgtcttaattatacaaagcagattcagatactaatggtgtactcacagcatggccatctgaagtccactcaatgatatcccatttgattttgatgttcctagttggatcaagagggttgtgcttctggaaagagataggcaagtatatgtagtacctcacgttaatacatgatttggttacatgcatgtcaacacacactttttcacatcaactaccaatttaaacgaaaatagccaataaaatcggtaggaaaactaacacaacgcagaccaagatttacttggtattagaacaaatcataccgattcaatctttgctcaatgaaaactgaggaagaactctataactatcctaatctgcactgatcattgcatgatatcaatattcctcttttacagaactaaatttcgacaaccatgtaagctctaaacattgttcttgattaaaaggacaaaaggataaacaaatggaataagtctcactagataatttggaacaactgattcaacttctataatttgaccctgctaacaaatgatttattggataatttggactgctgtacaaaatttaagagttgaaataattgctcagtttctaatttttttacttggcacatccactgaagaaggtgagctcattctctgaagcaaataaaaaatgcttgggaaaatgcttagaaacagtagtagcaagttaaacatcaagttggaagagtaaggcctgaatggcatctccctttttctaaaaggcaaaagatcttactggcctcatgactgaataagatgtgtagatgaagaaaccatgcacatgaagcttctcaatacaacttaaatgaattcactaacttgaagtaacaatgaagaaagaagaaaggattcataaaatgcagattctctcttctttgaacatatttacatgactcgacatctccttgtaggaaaggaaaaagcaaagaagtataaaataagaaaaaagccatgaacaagcatcgagggatcagtataacaatttcgaaatacaataatttcaatatcttgcaaaaaaatcacaaaaaaaattaagtagttatgaacaattagaaatacattatactaaaggttgagtacaatttgtcttttggattgagcaattatatagctgcagaaattttgcaaaaccaacacataacaagaacctaaggctctctagactccttttccttgctaaatatgcaggctttccatggaaaactttacaatgccatcagcagcccagcaccgaacagccaggcagccagcgcccagtcacaacaatcatgcacgcaccaacaagccagcaaaaaacacaacaggtgcccggacacacacacataactaagccactaagaaaacacacaacaagaactcgtagctagaaagcgagcgttacggccaccagttaacatgacgtccacagaaatataaccaagctcgctgatttcccaacaacatagcattcgcaatgcaatacgacacaaccatgcgagctttg

>TaeST2.45451.1

attttaggcccccttcataaattacgcatcatttattcccttgattccacatcacccgatggaaggcagcgcatggtcctcattgattggacatgcatgcacgctgcatccatgcggtagccactgcatgcgctggattagttaataggggtaattaatagagcgattacagaggcaatttgtaagatttattgctctttcttggtctttgcactttggctcttgggcccaataaacccggacggagggagtatttga

>TaeST2.45496.1

cagccttcctaccttccatggacgagctcgacggagacgctgtccagcccgaccacggccacgactaccccacgcacaaggtcccttcctgctcccccaccttcttccatggttcaagaagctacatacactgctgctgctgtttgtcaaattcattccactgaagccatcttcttcgtccaccaacccgcccagaaaggtagttattgtgaattgatgttcgcactgaggccggaggcgagaggcgagggaacgaaggtcggcggcaagtgctcaggagagaagaggaaggcggcggtggctgcggtttacacgagagcgaggtgacctgcccagaaaggtagttaaagtgaattgatgttcatatgaacttcaatgttgtgacgtttttattagatctgagttgcaatcctcaatttttttaggtttgagtagcaatgctcctgctctactagctatgacattttccatgttttttcttctaatatcgtatagcctccatgcttatgggttcaacttgacaagcttttttcatcttgcatcctgttttaagctctgttgatggttgatttctgccatgtttatggagggagaggatgccaagtactaggagaaggatcagtttagcaagtacaggctgtaattccttataattacggattgctgagaagcgcaaccttttgatctttgagaaccgcaagtttgctgacattgaaaacacattcattatgcaagacgaatgg

>TaeST2.45517.1

attatgctagtttattctttctgaaagtcttctagtaatgttggcctcgacatcacacaggccagctaaccgagttttgcatttccatcaggcaacaacatcaatacaactacaattacaaagtaatgcatctaaataatatacttccaatcccttggtgacccagatccataaacaaaattgaagggtggatgcgaggctaatagtactactagataacaacccctccaataaccgaaggatacccgataaatttctgctcgtttatcttccccttctctctcctttcccacaaaccgcgtctccgcttctcgagcccacccacaaatctccttcaagagagagagagagagagagagagagagagaggaggcacacctgcccggatcataaccaagattcaaatttgcccgctcatcaatcatggttcttgattcattatcatcgcctcacaggaggtcgcaaaacacattctttctgccatctcctaagaagcctcaatcatctcgtgatgatgttggtagttggtctgccctggttgagcggcatcgctttctcttgacaacgctagtggtacttgccttcctgtgcaccatatatctttacttcgcggtgaccttgggggcaccacaagcttgctcagggttgacgggcgatgaaatgaccgtatgtcaggaaaaatcagccctgcaacatggaaagttgaaatatcgctaactccgcttgacagtttgcaatgtgcttgtggtccaaattcgtatattctttagtggtggatgctgaaagggcttgtattgatgctgatgtacattttaatggagtgagaggggattgtttgtgctttgaaaccgtataactgaataggtcccattttattattgtcatggtgtactaaaaatatcatggcactaattcatcccggagattctgatggtctcacacacacaaacacgcgggcgcgcgcacacacacacatctctttattttgtgtgctcttttattttgtgtactcctgagagtatgtactcctaccacatagatgatttttctatatctctttattact

>TaeST2.45563.1

attctcacgtacgacaagctttattcagtgttgctgccaaaaccacagcctgacaaaaaatgttcacaatattatcacttatacacatctcatgtctattacttatcttattatacactcgatttactatgttggatatatgatactccctccgtcccaaaaagcttgtcttagatttgtcaagatacggatttatctagacatgttttagtgttagatacatccgtatctagataaatctaagacaagctttttgggacggaggtaatagaagtttgtttatatataacttcttgactaacggtctgggcttgcagtagaat

>TaeST2.45659.1

tagagaatatatatgacatatatagctagatctcatacattcgaaatgagggacacaatgtaacagtacaaactctcactggtcaacaaaataagtaagaaacacacatgtcttcggactgaacaaatatcatcatcacacaacaatatttttgatgaattacactcacacaaacacacatatttatataacacttgtactagagtaaacacatggactataaactggaacaaattagatgaacacacacatacacaaatacaaatatgatactataggacactgttctaatactctcaaatgaacaaaataagtactactaatcaagcacaaatctgaaattgaccaagtttcaagcacaactcaaatctgaacattacaaggcattacacatctcagcttataaaaattcagagaatatagagaggataaaagagagatagaagcagtttatggttcagattaggagcagcagcatgaggggttcagaggaggagcagcatgagcgcaagaagatcagcagcagaggtacagaaagaaggagcagcagcgaggaaagtgggagagggagcagcagcagttttaagaggaggaggagcagcagcgagattcagagaagaagaaggggggtagaggaagtggagtagcagcagcagggacgagggggtggagcagtttggggatttggagaaggaagagcagcagggacgtgaggagtagggaggagcagcagggacgtgaggagtagggaggagcagcaggggaagaagagatcgtgcgagcggtggcgcggcagcgaagatggagatggcgcgatgctggtggtggtgtgaggtgcgtctcgtgggaagtggtgctggtggtgcgtggtggagatggtcgtggctggcggcgatggtggtgatggtgtgatggtgaggggcgcaccaccacgccagcctggctgtggcggcggccggaggtggagaaggaggggtgagggagagagatgag

>TaeST2.45783.1

tgcagtccatgtgcgagaagaatggccagacccctccgtcgatgccagtcattgctccggcgggcacgcgtaactcccgacaagcatcgaccgatccttctccgtctgccactggcgcgaccggtcctacacctacacctacacctacaccttcatgatgaagatttgcttaagatcacggaagcttgggttgatggagtgcttgtatttagtttttgttttgaccttgtgaaacttgctacctatggatgaaactgtgtaatattgatgaaactatgtatatgtacggatgaaacttgctactattggtaacatgtgttggatatgtatgtgttcatgac

>TaeST2.45788.1

cgaccacgatggcgtctttctgccgctccgccgccgccgcggccaggtcggctgcgctccggtccaagtcccggatcgcgagcccgttcccggcgacgaggtctcccgtttccgctcctcgcctccgcaggtccacagtgaagatgctggcgggcgcggagtcgctgatgccgctccacagcgcggtggctggcgcgcggctacggtcgtgcatcgccgccgactcctcctgctggagctgcctctcccaagactttgctcttcctcggtgacgaaggctgaagaggtgggcatcctactaggaacagcactcaggatggatcttgtttatctgtttgatgctcggcttggcgagtccatggctacagagatactgaactttgtttggtgactcggtgtttgggtgcgttctttaactagtagggtttcctgttggctgaaatgtcctttcctctgtatcaactgttgatctcgtgcctaatctgaacatgagtttcgttcttcatgaagacaaaagttaaagtttcttcttcatcc

>TaeST2.46008.1

aactgttggattttttttagtttagcatgcatatgtccccgacactacgttattcaaacctgtacatgtcttcatacaacaatctattactgtagttttttgaaccaataaaccaatctataacctgggaatggttttttgcttcacaggtacaaagagtcacctgaaacgaccagggatgctactgctacatggaatctctcacaagaagcccgagatctctccgcatcactgtcccagtgtggagaaaacccaagggtgaagcaacccatgtttttcggacaaagtattgatgatgatgatgattgggcaaggtcaggagacggcatgggagttgggatataacttaatttatggtacctagctagctcttgtgttgcaggacagtgcccggtgcctagagttatttatctgtctctttttgttatgtagctctttagttagcactccctctgttcacttttataagacgttgtagacatttcagacagttctcaaaacagttcaaactcagctgtctgaaacgtcttataaaaaagaacggagggagtagttactagtcaggatgaataacaacattttaaattttgctctgatgcctttaatttggtcttctgtttagttgttgtgtttgtggctgggcactctgttctctaggtgttatttttagatgattaatttccaattttcttgtctgtcgaatgttctgtcttgtcctggatgttttgagatggaatagaaccctgcctgtaaaacctcaa

>TaeST2.46054.1

actacggccagaacgctttgagcggggagcgggccgaggacgcgacatatccaccaccaccaccaccatttagggttagaggatttgcgctctccagacacatcgccccaaagtaggtcgaatcgaagcgatggcgtctcgtctgcttgcggcggcctcctcctcctcctcctcctcgtcttcctcccccctcgctcgcctcatctccagccgccgtatggccggcgccgcagatcaccacggatcgacaaaggtgaacatgtggcaggagcccttgaaccccggcaactggaaggaagagcactttgtgctaaccagcttagcgatgtggggcgctattatatatggtggattgaaggcgtttggcgggaaaaaagaagtgaaaaccgaggcggggtcagcgccagcagcagcaccggcacattaaacttcatccgagctttattttcttaccacaatctgttttaagtatgttgttgacgataaattgagaccaagttggctggcaaggttaaggagctgcgctgcccagccaccatgaataatctgaacgtggtgtaacttttcatccaacaatttttctactacaggaagttggaaatatgctattttctgtgtgagcactcagtttcctataattcacacacagactaatgttcaggaaatcatcaactggtaactgcttggtcgatggtaactaggtgattaataattgtcaaattggcaagttaattgtctgataaatcagtaatcgttggcccattgagaaggaatttagtatcggtatacattttgatcaatgataaaccttgaaaatgaaatattccttttctttt

>TaeST2.46418.1

caaccgtcaacagacaacaacaagggactccatctagccagccagccagccaaagacgatgaggccaaaagccctgacgctgctcctgcctctcgccttcctcctcctcctcctcctcgtaccaccgctggcatcagctgaggcgaggtctgattgtacgagccaagtcatcgatccgatgggcaacaagtgcgatcgcccgggttgttacttcagcagccgcgtagcgtacgctcagatagggtgccataaacccgcggtcagttgttcttggaccgcctactgcagcagcgaggggtgcaacttcacctactgcatgtatagctaagataggctggcatgtccatctcatggaataacaaagattatatcgtggcaatgcaatatgtactaccttcgttcctaaatataagatgttttgccagtttaaactgaaccaacgagacctcttatgtatatttagaaacgatgtaccagagtcgtgaattattaacatgcctaccatgaatatgtctcttt

>TaeST2.46584.1

ggtaaaatcaacaaacaacattagaagaaaaggaatagctaactatctcccatggcacgtatgcagaacaacacacgggccctatgcttcatagctcttgtagtcatggccaccactgtcttctcaggttatgcagcaggtcgttacatagatactgacctgctgtgtacacctgtggagtattgttcgccgccagtccgagctagtggcaacattgcgtgcaaggctaactgtgttaaccgaggttatgccaaggacaagagcagttgccaggaaggcagtagtggcacatgctgctgcggaaaaaactaagaaccatgaaatgagtttatatatgtactagaaaatattgcatgaaggtgaatgcttatattgtttgtgtgaaacctgattcagttcatacaaaacaattaataataagctattgataattgttgtttaagt

>TaeST2.46649.1

aagttattcaatccaagcattcacaatggcctgcaagatagcagtagtcttgtctctatcggtgttagcattcttcctctgcctcggtactgctgaagctggcccgcagtgcatgtacaagactcatccttactatcatggtaggtgcgagacagattatgattatgacaacgacacttgtcgaagtgcttgtaaaaaatatgaggcaccaaagtacatgggtggtatgtgccatgataaacaatgcgtctgttatacctgttaatgatcatcggactcttccgccgctggggcagccatgaagatacatcacttgtgccttggtggacaccaccatggaaagatttgagctcctaggaagattgtctttttgttgtaacagaataaattggcaatgtaccgaactatgatttagcaagtatttgttttcgatgagttacgatgaagagtttttcaatcaatttataattttcgcctaatatcttgtaggcatcaatatatatggtgggtctatcttattaacaccca

>TaeST2.46827.1

ttctcacctaccacgaaagatgataccgtattgaatttattatttagaagtgaggaaaaatacacatggtgcaataacaatgattgttgctcctcatctttgatcgctacaaaggcttttgcgacctcagaaaacataaaggaaaaccaaatatagtatagctgagaaaaagggcagcccggtgcatgtagctcccgcttgcgcagggtcggggaagggtccgaccactttgggtctatagtacgcagcctttccctacatttctgtaagaggctgtttccaggacttgaacccgtgacctcatggtcacaaggcagcagctttaccactgcgccaaggctaactaaaacaaaggttaaacttttcctattttactttgctatgcacatcactttaaagggacttcagtgtataaagggacttttcctatagtaacagttacttgcctgaatcatgtcccttcactggacacctcacactactccccacggatcacaacactcagcgtcagttcacctcttgcccctgcaagaagctcttagtggcaaggtcttccacgtgtacccaaatgggcga

>TaeST2.47080.1

aaccaacaacacaaaaaagaaaaggaatagctaactatctcccgtgccacatatgaggaactacacatgggccctatgctttgtagctcttgtagccatggccaccactttcgtctcaggccatgcaacaaggagttatataggtacaactgacatgtgtgcagctggagctgacgaccactgttcgccaccagaacaagctactggtaacattttgtgcaaggcttgctgcgctgcccaaggttatgacaaggataagagcagttgttgtcaaggcagtagtagctcctgctgctgtgtaaaggactgagaagtgccatgaaatgagtttatatatttgctagaaatattgcctgaaggagaatgaataaatagtctgggtgaacctttatttagttcatacaaaacaatcaataaaccattgagaattgttgatttattatatag

>TaeST2.47085.1

cttcggcagcaaccaacaacacaaaaaagaaaaggaatagcctatctcccgtgccacatatgaggaactacacatggcccctatgctttgtagctcttgtagccatggccaccactttcgtctcaggccatgcaacaaggagttacataggtacaactgacatgtgtgcagctggagctgacgaccactgttcgccaccagaacgagctactggtaacattttgtgcaaggcttgctgcgctgcccaaggttatgacaaggataagagcagttgttgtcaaggcagtagtagctcctgctgctgtgtataggactgagaagtgccatgaaatgagtttatatatttgcgagaaatattgcctgaaggagaatgaataaatagtctgggtgaaccattatttagttcatacaaaacaatcaataaaccattgagaattgttgattta

>TaeST2.47143.1

attcttggatcaacaaacccgtattgtgcctccaaaagcactgattgtttgaagtaattcaagtccgtgtgcatggagaagtacagggaacatgcgcacctgccagtgccactctcgatacttctcctcgtttgctttgttgttcatgtgcaatgtgggagaattgaagacataggtaaccggaagatcaagctaccatatggattgtgtgcaccacgtaaacaaattggaaaatgcaaggaccattgttggtgttgtttagtgagtccaacccccttcacaaacatttgctatgagacgcctggagattgtgatgagtattgtcgagtttgagttacatgttgagactctcggattaataaaaataataaagtcattagtctttgcggcaccattctagtatatgcacc

>TaeST2.47148.1

caggttgcacagccaacatcagggtccatcccagtcccagtacgtgccatttgattcaggggtcacatacataattcaatcattccatggaacagccagctcctgcctgagcacacataattactcttcttatatcttcccttggtctagtctagcagggcaagcataagttaacggcattattattaaatcaccatcatcgtcatcgcggctaaaccaacatcggtatgtaacctagcactgtataaccaaaaaggaacactaatctgcaagcaggaagtttgcataataggcgccaacttccgacttgttcttcttcattcacaccgccaccagcagcagctgcagctgctgtgcctcggaagctcatatgggagcggcgcaggtggtcctgaagtggcccgtctggttgca

>TaeST2.47191.1

tagcatattcaacacatattaccaatagtcatgaacacatatatatccaacacatgttaccaatagtagcaagtttcatccgtacatatacatagtttcatcaatattacacagtttcatccataggtagcaagtttcacaaggtcaaaacaaaaactaaatacaagcactccatcaacccaagcttccgtgatcttaagcaacatcacctcatttcca

>TaeST2.47291.1

tagcaagacatatttcatctatatggcaacttgctgtcgacggaatcccgcactttcattacaaccacactcatcaagatagaagacaagttcaagttggaaggtaaattttcatcaagctactgattatattcagagttttcactacagaaagacaaggaaactacagattacattcagggttgttcactttgaatagcaaggaatatttcatctatatcgcaacttcttgtcaagggaatcctgcactttcattgtgaagcagagacgacaggtttgaatgtaaacctacgattgagtaatccagagcaatgagattaggtgcacactaaggttccttgtagctaagctgatccctcgagagtgttgatgcttagccagccagtgcagctgagcagccttctcttggcggggggagagtcgaagccaccttcagagggcaggagcaacttgtggacccgcttcgacatgcccttgtcactaagcttcgtggggaatttggagacca

>TaeST2.47611.1

gtgaattgatccttccaagcccaagaggatgcaggtactccgctggcagatccttcccatgctgaaggccatgttcatgctactcatctggatgagcatggttctaccccaagacaggatatataccatttgttgcactacagttggtatatagcaagatggataagaaatacccaagagcttcttccttttcaagattagaagatggatgatagtgatacgcttctgagaagaaatcgttcgaggtactgggcgtagtggtgatggagacatatagacttgtaagacccttgggtgggcgaacgcccttgagcacctccacttgaacttctggtgaatggaacattccaagcccaagggaatgcactccattaacagaatcttcccatgatggaagaccatcctcatgctactcatgtgcatgagcatagttctactccaacatttagaaattccagtagtataattagttgttaatattgtgaaatagggtgtaggtaggctttctttgtcaattgcttctcttggcaatttaggaatcaaccagtatgcgtgtttcatatttggtgacacctcctgttaagttaaggctccatgaatcatgacttggatatgaatatgtagatcttgtgaataacgagatttaaataaattggatgggtacattgtgacctaggaattggatgtgattttaaacagggttatatgatctttgtgtatgcatgaatgtgaaatgatttggcatgttgctgtgaatata

>TaeST2.47733.1

aggatgaccgattcgccttgagcccgacgacttatattgtgaggcaaaaggagcagaagtgtcacatgttatacaagttcgtcaaactagtcattcacttggaggttggtacgttttgatggaagcaccaaggtgaagcacaaagaggcattttcatagtcaactccgcctcgttcgcgcttgccaatagcagagctggatttttagttggttttattgttaatttttctatgcaaaataaaatgttaaatgaatgaaatatctatgtccttttatttggtaataactcctgttgtgtggcgtccgtccggccagctatccgtagacatgttacaacgtgccaccaaacattcgtccagttggtgatccaagttggaattgcccgcaccaccacgtctacggaccagccggctatccacagaaatgttcaaatgtggcagcaaacgttcgtagatttggtgatccacgttggagttgcccgcaccaccacgtccctcgtccatttggtgatccacgccttgaagttgaccacagcagggaaaccacctctgctcagtcatagtagccacaccaggcggcggcaacagctcttctacatcaatcagaaaccctaggcctaactaccatcattatatgaactgatatcaagagagagaaaatacagttagcacaaactcaaatcctgaaaagcctcctagattattgtgactactcacattatcgctcaaattacaatagataaatctaatacacaaaaattgggcaacaccaatgttccagctgtcctctggggtatctatgttcgaattggagaagttatcattgcagaagcttctgtacatgcctcctgcatataacctgatcaaggttcaaacaggtggcatatggaggcatgttcttttgacaggaccatacgtttttgcatgccttggttcggccttcagcaactcaagttccgttgttaccctgtgagacaacacccagttgcccaaatcaaatatttgaagcagacaaagtccgtgcataaaaatgaatgtagggtcactatcccaaccagccaacgattatatcaggtcaatcttgctccacgccctttgacaaggcattttcttcgtccatcttgtccttggccacattggcatttacttccactatgttgttctcaatagccacttcagcagtagtttccaccatcttgtccttagcctcatcagcactttcttcccccatcttgtcttctctagtcacattaccttcatccatcttgtttacttcctgcaccgtatcatcttttgactcaacagtatcttcctctatcttgtcattgttcacagcatcagttgcctcaccattttctttctctgtgccatccttcgcattatccaaaacaatatcaccattgccagtttcaggaggggctgcatcagcatttgcatggggtgcataatattgcatggattgcatctg

>TaeST2.47812.1

tttataccgaaatgagtatatggttttttatatgaaatgatggctcacatgtcacaggggtaccaataatcgatacttccgcacaaacaacatatcactcaaatagtcacttcataaatataatccaactgcatactttagaagtttattaaatctatgatcggtagtggcatagatgattgacattcacccttgtaagagaagagctcatgacaatacattatactatcaatttaatggccttccatccgcatagtggcagcaactgccagcatcggcacaatagcatctgtgggtattccagccaaggtcggaacatatcttcgtgcacccaacaaccgaacattttgtttccgtctttacacacgcaagaataggtccgaagtccattgtat

>TaeST2.48406.1

gcggcggcggcggcggaggcttcaacggcggccgcggcggcggcggcggaggcttcaacggcggccgcggcggcggcggcggaggcttcaaccacgccggcggccgcggcggcgccaaccatggcccgggtcgcggcggaggacatcagggtgccggtcgcagggcacgtggaggtcgcaattgagcacttgtacatgacggctgggtgaccgggggagacgttttgcgtggaggtttctggacgttttgcaaggaggtttcggaatcctcgtggtgtcttgtccgggttctgttttcatagatcgatctcgcagtgtaggaagatttgtgtcttgcgttgtctgctatctaggtttctgttcaaatgttatctgggtgcatcttctgttcaaatgttatctgggtgtaaggcttgtgtctgttaagaagtactggtcgaaatctgtattggcgtttgtctggatgtaacgcttgattcgatgataatggaagtggatgtcgggaatttctgtgcactccaat

>TaeST2.48614.1

tacaactgaacactcttcagacatatatatcccgcaatcttacttcacatctcaaccaagaaaaaacccagggttgccaaaacctaaagtcgctgtagaaatccgaaaccacaatctcaaaccttacatatgcaaatacgcttaaatgattttccgaggatatatatctgatctcttcgaccaactccctggttagctctattaactaacccatgccttttaatcatgtgaagttcaaaatttgcacctgatgcacctgcggttctcttctcaaccaattatgaattgctgtatgcgaaaaagattcctaggcggcgtcattcct

>TaeST2.48654.1

ggtgcaaggacagcaccccgccgtggctctgcaaggtcaagtgcgccactggctgccccttgagcggccccggccacaccttcaatgggatactcgtctgcttcgacgcctgcaactcggactccatctgtgacctgccggcgccattggccaatgctgaggtttgtaagcattggtgcttgggcgccaactaaccttgaagagccaagggccggtcggtcatgggatcacaaattcacaatcagtccataaaatggctctgatttctgcaccagtcaataaaatgtgtttctctttgtgcatcacttgtgagagaatttgttaatcttaaaataaagtgtgacaaatccctgtttgtttgactcggcta

>TaeST2.48692.1

caaggtgggatgtacattaacaagggggggaggagaaaggaagagggaggtgcggtggtggttggttcaccggtccggtggcactggattggcaccagcacagagctcctgcttacggaaacgaagatccgttcttggggatgcgaagatccagggaggcactctcccgttcagttctccttactatcggttcagggccatgccacagcaggtgaaagttaatccccttctgggccggcagacgtgacatggctggctttcacgggattggcattggtggcgcagtcaatactacctacagaagtgccaccaacttgcaccaagctattgcgtcaaggtgagatgatcaattcgctccttggcggccgccaccctgtttagtactcttattaatctaattcctcttatttctctcttgttgatgcaaacctgcaaacttcac

>TaeST2.48980.1

aacaaagccgccgcgttcttctctgcgacacgcaaccttttcccctccccctacagactagaccagatccttgatatagagaagtacacgcctcgtcctcccaaccacaacctcctctcgtcgacaaggtgagcccgtgagtctgtctaccgtgattttcccccgtcgaaacccccgtgtgctctctggttcgcaggttcctcgtcgatcgacgggctgggcttgcggcgatcttggagcgagatgcgaggcagctgcatcttccgatctgcccgtccggcgagtcagatctgtttcatatctgtcggcccgcggcggatctggggttctcggcggtgtcgagttgaccctgcggcgaggaatgcgtcttctccggcctgcacgcggggatcccgtcttctctgcatcgcgcgacgacggctcttgtattgataattatccgatgatatttcgataattattagatctaagagatcggaggtaagagcatgtaacaagcgaggtgatcgtgccgagagggaaaaagagtgaaaaaagagagatatgtgacatctagaggcctttgccatgttgggtgcgctcgcatggcaggccaccaccgtaataaagatggttcttgtgtcccggggggagatcgcacgggggttattctgttttagccccggggtctccccccacagtgtcctggtgtgctcctttgtctgcacctctcgtccgatccgatcttcctggcacaacgttctaccatcgcgaatcaaccatggagctaccgaccaaagaccaaaggtacaatcgataatctacagctgaagcatacataaacttacacaaagcaccgctctcgttgatcttcatatgtggaaataaatttgagtcgttctcagttcgtttacgcagaagaactggagcccatttaagttttcctcatagttttactgattaaatcatttgtctaattcgctgttgcatctactatcacatcacagctcaaatttgcacttcaataacaaccggcagtccagacagaccaaatagttgtggtatcagattcacta

>TaeST2.49222.1

aatacacaagaaaagagtaaattaaatctaacaacttgtcccaattactttagtcaaatatatataccttgagttttccacgttacattacatggtcaaatacgtatattcaacttgcccacattgcatattagtcagacacaaatttaaccaacatactaacatccaactaccacttgagataaaccaataacaattatacagcaacttaactcagacatcctttgcaagtttgtggaaatggggtaggttcgggataagagaccatgcgacggtgacttcaaacttacaacggttaacttcaagcatacgcatgctcctggaggctttcttggctctgaatgcttctgaatctctctcaacagtttgatcctccaagctacttgccatgacgcagactctcaactctccattagcttcaacagatacaacatgtcttgaaagctgtatcatgccatcagctgcaagaggcaacttatctccgaatgcaagcaactcgattttcttggaatcaatacttgccgtattggcagaaattagaccttgaaaaccttctggccattccccaccaacaactctcacactgatggtggcctccactgaattaacaatatcacccagcatcaatgccactgtgcaaagcttgctagtgctgacacgtttaatcgcgtatgattcactagagccagaatcatgataattaaaaactagacaactcaattctctatcctcggattcagttcggcccttaacccgcaagttaacctcaaagcacgcaggatccacaacaacaacagcacgggtaggacctgtcagtctcagacatggattctgcatgcaaaatttaaaacgtaagttatgtatcatttatctcgagtaatgactggagcaaatataaccaatctatgctgatgtcaagaaagaatgcatccactgacaagtaatccatttattacgctataaacttaataataagtgcttgaacacaatatatgttggtagaaagcagaggaaaggcagaattttccagaggtgagcccatgaatttgagttgg

>TaeST2.49486.1

aacaagaagcgtttaaatggtcacgcatggcaacaaaagcaagtcggcatatgaagcaatgttccagggagctaggaacaacgttcatacaacaggttataacagctaccatatgtagaattacattattctcatttcaattctcgggatgcgaaacagcatgggctggtccatgatctacacaagtctgatacaaactcaaagtgcaaagcagagtggaaagaccactcggttacagcttacagaggggttggcatcaagcaagacttgctctctcaccttgtaatctggctgcttggtgcttgctattgcctcctcagttttctg

>TaeST2.49522.1

tataccaaaatcttttaagggaccaagtttatactttttgagaatttgacagaccaaaaacatacttttctctaaattgaaatcaactaccgatggttcctttatgatcgtagcatatctttaactactaaaatataggatgatatgatcctccaaaaacactacgacaaacacttttgaatctctttgcggcacattgcaagcaagatagataagaatctcctctttattggaacatatagaaaaacacaaccaccgttgaagcacattttgtagggcatagcttatagcttagaggagaattaaaccacgtatcaaggcttgatggaggatgcagttccattggccatcacgcggccgaacccacaacaccaggggtccctagggcaacagggtggatcgtgtcccataagcctgccggctgcctgcgctatcggcatggccatcatgagtaaccagacttgtgcaatgagaaaaacaatgaagacttggaggatgatattatttgccattgcctaagttcacagagagcggtagagaacactatgcaattttcctttcac

>TaeST2.49678.1

ctattattgaagtgccttctggttccttgcgtctttcccctcctcatcctctgttgatccaggcaccacccacccccgtgccccttgcggcaggtggctgtcatcttcaaccccaactcctcgccgccgctagaggagaggaagcataacaaaagcagctggcagatcgatgcagcgaatcgaggaagacgacgcggccgatggcacatgaattttcagtacctgggagattgagcaagcaggaccaaccagcttaattacttggtccctgcttgtgtcacgtgtcgaaccaggcgcgtcgtcccggaaggctctactgtaatgtacaggttcttcatcacagtcaaatgagccatcgaccaatgtggagagaggcagagagaggtttgaggttatataaaatgtaggtgtgtgagatcagctgagtgagttggactgcgcaacgatttagctagggggcacgatctttggatgttggtacttgaatgagagttcggtgagtgagatttggt

>TaeST2.49696.1

ggacacaataacaataactcatgcatgcatgcaaattcagacacacactttcaaaccaggtcaggtactcttcattgcaagccaagcgagtgagtgaatcgacatccctgatacataccataccacacaacatcaatcaatcgcctaacaactaacattcatcccatctcatctttgcgacgaaccctgggtaggtggtctcagtactactgactcactgagtcggtcttgttgttaattaggcaggccagtcct

>TaeST2.49700.1

accagtcagttactaccgcggtgacggacggacggccgggcgaggtggcggcggcggcgtgcggcgggatgccgcgcgtcagtgacggggatggcgaggctgtgggcggggcggcggagcacggcagagatgggagggcgctggcactggcggacgatgatagggcaggctttattgccccccggcaggcctgctgccgctgcaaaagctttctgtgatttctgtttacacttttggtgattagtgactgccatcactatttgccaattttcaccccagggaa

>TaeST2.49768.1

catgaaagtataggatgatatgatcctccaaaaatactagagtaaacataatttcaaatctctttgcggcacagtgcaaacaacatagataacaatctcctcccactttattaaaaaaaatagcttacacataatatttcttgaaactagtagcacaaatcgcatatatgaaacaagacgaccaagttgaagcacattttatagaccctagcttatagctcggaggagagtcaaacaacatatcaagatttgatggaggatgcagttccattggacatcacaccaccgaacccacaacaaaagatgtccctaggacagcaaactggattgtatcccataagtctgccggctgcctgcgctaccggtgccgccatcatcatgagtaggcaaacttgcgcaatgagaaaaacaatgaagacttggaggatgctattatttgccatcgcctaagttcacagagagcagtagaaaacactatccaatactcttttcactagtatgtttg

>TaeST2.49782.1

ttgaacaactaaaacacaaatggccaaacatttcattagtcagaccatcaccgatacagatatggtaccaccacaaatttcagttctagatagatatcagtctgccaaatgcaacaagttcagctcttacaactgattacttagccaacgaagcattggcgataacccaggcaagactaccagtactacatgcagaaataagcagagtcaacagtacagatacagagaccaactacatgcgacggacgcatcagatctagatctatgaggaggtgaatttggtgacggccttggtgccctcggagacggcgtgcttggcgagctcgccggggaggacgaggcggacggaggtctggatctccc

>TaeST2.49799.1

tactttaccaagctgtgtttttacagccggaataaatggcgctcctggttcagcccacaactcacaaccggagtactggtagtacatgtgtaattagctggtatatgctatatgatcaaggtggatgtttagcggctactcatggttcatcccaaaattttggtgtgatatcacctgtaaattttccctcccgggattcgccgaggaagtgaggagtagtacttacccaggttta

>TaeST2.49854.1

ctattacaacaagacagaagagagcctgagaacactcagcttgcactgtgggttgccaaatatttttcaatatactatggacccctcgtgtttgctcgagtgtattgtacaccaaaatcatgagagctccacacataaattttgcccagacttaccatgttccttcaagtgtaagcatttgaagttcttgatgcatttgagacagtggaaacgagagaagctgggagcgtagcactgaccgtgagtgtgattgctacagctctaagagatagacggaggtctgaactggctgggaagatttgacaagagatgtggagatggggcacggggagaggagggcgagtcggtctagaggccccggttgtggcgcagcgcgacgaggcaggcgtgggactcgcggtggcgggcggcctcgcgctggagctggttctggcaggcggcctcggcctcatccacacggacttctcctggttgtcgaaccggttgagggtcatccaacttcttgtttatcaggctactgtttgataaaatgtaagatatttatgtttggaaaggtttcttgacgtccgtaccggtcatacttttgcaaggttagatgtgttcttattgatgcacttcattgcttcttgctacacgctctgtttgatttatggcgcgccttgtctttttctaatgttgtcagcctttgtctccttattagcctgttaaattatgtatatgggttcacgtgtaagcaagctgagtggattctgcaggctactgtgtaaaatgtgcatgcataaccgcccgtaaaggggtcctgtacatcatacaaagagcagcggcaaatgcttattatgttccattaaagcagattagaagattgtttagtttgggtaattttattaattgctttttttctcctaactgaccgaactggctttatccaaaaaatgaaacgagcaggcttgattaacttacagaagagaattttttaaaacgctgtggttatagagttgtgctcggtttgcttcttttggtcattcttgatataagcaattgggtagcgttgatgtttactcatataaggaatggattgttgggaatgagtttatcagagcctactagaagagtttatggaagtggttctatgaacatgctgatcgtgatgcactcatgctgaccacggtgtgtgcaactagacagatctggttgcttgtctttggagatcttcacctgtaggttgtgcatttgtctgcatttatattagcagctgtgtttttataattaggtgcctggatttagttgatatacgtctactgtatttaagtacatgggggaggcgcgcatggttgatgattatgttctgggttggccattgagagggaggtcgaagcaaactccgtcaggtttca

>TaeST2.49947.1

tagaataacaattgaccatataaatcacctgtttgcctcaccaaatggcagccttcaagaagaatattggccccttggttttggtggctctcatggtcctggctattgttgtctccccatgccatgcaaacaatgagatacacacaaatgcagtgcgggtacctgcaagttgctatcctatgaagtttcctaaatgcactaacaaggcatgctacaagttttgcataaaccctggaggccactgcacagatgttgattattgttgttgccctatcgactaaacaccattggattgtcgttttaaacctatgaattaagaaagcttgttattttctcttcattattgcaatggataggcttggtcctgaattgagagtataaactcccgcaattcaacctatgaaatagaagataaatgaatgcgccatgatcatgcacaattttgtaaaccttctcaaagatgaattttggaaaaacaaatattatccacacaaattcttaaaatatccaa

>TaeST2.49956.1

tggacccagcacatatgcaacaaagtcaaatgaacagaaatgatcggttacagctaggagtgtgtgcgccccaacaaatttgatagaaataaaaaacgaacatgtaagtttacaatatgtactcatatcatgtatgaacacaagaaaaaccaaataatgatcccaaataattgtgcatgacgcacgtcgtctaaaagcctcagaagatgtagccgaaaacgagtgcggtcaaggacgccatggcgacagccggggcggtcgccgacgcaccggaggctggcgcaggagctggtccgtcggctgcggagaccgtgctgaccatgaatgcggcagcggcagccacggcgaggatcctgagcttcagagtcggcgccatctgcttgctcctagaaggatgctgctgcaacgagctaccaaagattactctggtgctggaagagaaatgaagatgttgtcaggct

>TaeST2.49964.1

acaatgttttgcctccaaattccccaaatggcagttttgtaacaagatacatctgttggaacaatgtttttgtctccaaatgccccaaatggtagttttctgggcacagcagggcgaccgttttacatcatgtgttggagatgctcttattgacagctgcgtcttgccacacagtgacacagtaaatataaaagcctggcaacaaaagtcacgcagcctggccgaaatcgaagccatgcaagccacaaaatcattggtagaattcatggggtaatatacagttatacgttgacaaaggtatagctagccactccaggctattcaagttccattttcacttttgaggttttcaaccagcaaattcagtacgaaaccaaattagactcaaacgaagcgccttctacatagacgttctacatagacgacgccttctccaagcagccaaagtacacatcacaaaatgtgcacatttactgccaaccagctcaatggatcaaattacgtctgcacaagcacaagatgatgctatttctttccattgctggaaaagagcttctgcgctgcaccattcttccaaacgcttcgcacaggcaactcagtttgcaggacttgtgtctccctttcaggccgttcagggctcagatcaggatgactaaaatcaagagtagccaatgagttgtcacccaaccgagctctgagaaacttcgacgtcttcttcgattgtttgttgttactgctgctgctgcttcctccacccttgcccgcatacctactggtgccagacaactttgaaatggcaactggatcaccgtccagacccatatttgcaccagaggatgaccctccaactgacactgggaatttttccttggaagatcgatacccatccttcgaaagcactgcagcctctccttgagccttgttgctcgactgaagcttccttacagtgtccataaagctatcagctagcgactcacttgcatccttagcagtaactgtttct

>TaeST2.50130.1

gaggtggtggtagatggcggcgtcggcgtcgtcgtcgtcgtcgtcgtggggtgcgatagcagcggcggcttgatgttgaggttgctggtggaggagagggagaggcggaacagcgttgttgggtgcagaggagtagtctggatgggggtgggagaagagcgggggcgagtaggatgagtagcccagcggcgaaggggaaggggagtaggcggagaagctgaagccggcgtcggcaaacatcttcttcttcttcttcttctacttgttgtctgctgtgctggatctctctctgctctgctttgctttgcttgtacgtatgaaggaaggaaggaaggaaggaagattggatctccggtccgggatgggtgggtgtatggagggaggtccggcgaggaggggaggggctggggctggacctaggctagctagctgccggtctgctctggtcacttttttccccactctatctctctctctctctcttctctatgtgagtgatgagatgagtaggctggggagggtgaagtaatgggttgggagagattggaaaggatggaggcctccgcaggcgggatttataggaggaagaaagagcggcgggctgctgctcttggttgagaggaagaagaagaaagggacaaacgaaagaaaagaaagcatcacaaaaccaacgccaacgccaaacccaacgcggtgctgctgggctggactcctggctgaaaagagtctcaccgcttgctcttgcttttgctcttggttggctggttgctgcctactggtaaacaacaagcaatcaaggaagcaacccgtctgcttctcccgcatctccctccctccctctcactcgctctcctgcttctggactagttactgccagccagccagccagccagccagatgcttcgagcaactccaaagtggtgacccaaacggacgcgcatttggatcgatcaggcatccggtttttcattt

>TaeST2.50173.1

gctaggcaacctcatgaacaaacataatttagacgatttggggagaagactttattagtttcacaaagtttctcatacagagtgcttaacataaactattctgagtacatcctcatgaattacaaatctgatacaaaatctgttgtgagaccacacacaaaaaatgtaaaagccaatgcttatcccgcggctaaactcggatcactacgccaaaggagggaatgtgtaacctgca

>TaeST2.50240.1

agggaatagccaatagataacttgcggtgacagtacaatacatatgtacagggcaaagtctgggcattgtgtataactgaaaaaagagagctctaggaacacataaaagtaaatcagcatgttatcagaaactcatgagcatgaaagagaaatttggatcatgtgcgaacatgggtagagaggaagatcgaggagaccttgaagattaaaggcaatgaaagccagatgaaggccgtcgaagatcacaagcgcaacttgctattgtacaggacttcaacacataagatcacaaactcatgatgttggcaatggtaagggggcgaaatcacgaagggcacgctttgtgttgcaaggtgctcaaactctcagctagatatttgcagtctgacatacatctgcatatacaaaactcagagaagtcatttgattgaaccgtagatctgtacacgaagaccttacaaacaaagcctgggttaggtgggtgagcatggaaatcgaagctagcctggattttggcaccgtttccctcagtgtttcaggaagttgaaatatattgggttgatttggttcgatctgaaccggccggattgtttgagaccagttcaaatagttggttcaaatatcttgggttttttgactacatatatttgccttactgttgtggaccggatcagaccctgttaactgacagttgtatatgtttctaatagttggttctgcctctcatgtatagattaactgatagttgtatatgttccgccccagttatcatgtat

>TaeST2.50252.1

aaccggcagaaaagttcaagagtcgggataatcaaatccaccatttcgtgcatcacattcgatgacagactcacaccaaaaggcacagatcttagaacaggtagcaacactttcatttcagttactgatgacagcaagatagtgacatcagatcagacaaactacatcaaccatgacacaaacaaacactagcacgcacatttccaccgctgaacacatctggctaagacgacgacgaacagagcttaggccctctctccacggatgcggcgtgcgagctggatgtccttgggcatgatggtgacgcgcttggcgtggatggcgcagaggttggtgtcctcgaa

>TaeST2.50391.1

catgcaagatcatagatcaatcttaagacaccaaacttgacttcaataacttagtccatccaacagatagatagatctgtgtcctcatcacagagagatgacaaaataacttggatcttaacgaacacgccaccacaattcttctggaaccaagctctcatcgagcagcagggggaaacgctacggcgactaatactagtactgataggtataggtatgacttgctgcggaatcaggaggtaggtaaaggagcgaagggaaacggggcacccagatctgacgagctgcaccgcctagtgggccttgttggggcactcgcgggagaagt

>TaeST2.50485.1

gtggctttttttcatcacattacaaattagcctgcaaacaagttcgattcatcggggctcctggtaaattatgtccagactcaagcatcacaaaggaaacaacaatcgcaccaaaacctatgaacaaaatcagatttacaacaagctgacagagacaagtaagatatgcaaataactttgttccacgattcagttcgaactacagagaaagtaaacagcgaactgaacaagttggggttccatcaagctttcggaatctacagagcaagacagctgaagaacgcactggtaatatctgaaccgcgcacgaacaaacatccacagaccgacagattaggtacgaattcaagggtaaactgctgcaaattccattccgtacagaagtacatccagacaacaaa

>TaeST2.50491.1

gtaccttttcatatttctcaaattcatggcttctgtcatacccaccattgagtatgattacactatggactttgatctgggcagtacaagcacataaatcgcccattgaagaaaagctgaaactcccaagaaggcaagcaggtcattgtcgccgaataatttctgctcttcggaaggatctgtacattgtgtccgtgtgtgttggttcgtttggcaatgaactttcaattgtaaactctgatgctctgcaggaaaattgtgctgattggtcctcttatggtgcagtgccgcactgatatgacatttatgattcttggccaacaaatctgaaattctgaattatcttg

>TaeST2.50525.1

ttgacaggaataagtaaagcgtattccagcttgccgacggcagaaaattacagatcaatggaagacaacaagtaaagcgtatttagcaatcttcggacgcagaggcatgtagcaggcattcgcaagatcaaagaacagataacagatacaatgtcacccttgctcgacgaatctgacgggcactacagactacatacaggatctcatctttttagtccatgagaaatggatctaagcaacactggctcggctatgggggcggggcgcctactccttggcggcgaccttcttct

>TaeST2.50531.1

atgatttataataaggttcagagtatgatgctggtagagtttaaaattgtaattgaaggtaaacacaacacaacacgatacaaacccatctagcttacacaacatagggtccaaggctcaagagccaacacacacactcacacattcgcacacgatacaaaccccaagggggccctatgctcacaagccgaaacacacgcacaaacactaagccgaggaagaccacaacagcgaggaacatcagcgtcca

>TaeST2.50565.1

agcaggtttaggcaactcaatgagaatgcacaacaaactagtagcaacgaggattgaagtttaattcccacattcggtacatgatagagcaaacataataaactgaaacatccagcaaatcatcgaatcaacaacatcctaacttaaaacattataagctctcataaaccccagcttctcgactaagtaagactgatgggtaccactgactaagtaagcacacaacaactccccaattcaagctcaaactggctggcattgccatggggccgcatggcacggtggcggttgcttcaatttttcgcagcttgatgttccacttcgtcccagcggtatatgctgccatcctcgccgcaggcaaggatcgtgct

>TaeST2.50574.1

tttcaacagcaaacactcaataattctctcaattaaatgattaaattcagaattcattcatccaaatacacacaacagcacatacaagtctgcacatccaacatcaactacaacaagcaaaaacagatacacaagcacatacaagtgtgcttacaagcaaaaacagatacacaagcaccaaagcatggtgcaacatcaactaaagttgtggtgcacatctccatgcctgttctgatcttccatcacagcagcagcaacctaaacaacattagatcaccaaaaagaaacattattaaatgtttagaaagaatgccaccgacatgagccggtaatccaacttaacactttgtcaacatattttacatatgaaaataactaagcttatattgtataagtgtatgatccaaatgcaatgtgaaattgaattaccaaatatactatatgagcagtcacggtcagagaaggtatagctatagaagtataatatgaccagttaataatcaaagacatagcagtaaccattaagaaaagaaaatgaattatgtcgatgctgaaagaatatctctggagtagttttaattgaaaaaatctacagaatactctagtaattagagtacacggctaagatgttgtaaaaggttccatatttgtaagatattcactcaactaaaagaaggtcgagttttacttgcaaatgaaaacatgctagaaccttttacagtagaaccgctatgccacatggtttttctttaaataaaaaaatgagctgcaacgcaggctaacagaaaagctatgagcatctttgggcagaccatccaagatgatgaacaaatcagaatgaaaacatgattttatatttcagggagacagtgggtaaccaaacatttcaacttatcgagaaagaccttgcagaaatggcctcgtccacgccttttcttggccccaaactgaggccttccatcatccgctcgaattcgccattgttgttcaacgtacgaaagatgcctgccaggaggatgaaatcaaatgaataagaaccccacattaacaaagaggctggacaatgattctacaaagtattac

>TaeST2.50597.1

agtgaaccataaggacctggctcccagtccaagctcgaccgagctcccttctctggcacaaagccatgttttcgaatcatcgatctccgtggactatagcggtgtagaaggacacccaccccagatcaatgcacctaagttggtagagtcgcctccgtctgactaaagccttcgagaaaatacggcacaccatgtaaacgggaagaaagtgccccaataaatatccctaattgttattgtattcttattgcctttggcacaagtgtcttgactagcgattacagcggaccattttatatgatatatcgatagcacaatgcataatatacaagtgaatgtgtgtttttg

>TaeST2.50624.1

gggcggcctccgcggagaggaagccgttctcgtggcgcacctcggccagcttctcctccaggtcgcgcttggccgcgcggagcgcgtccatctcctccgggtcgtaggccgccttgtggcccccgccgccgccgttggtgtgcgccgggccgaagccgaagtcgaactcccccgccgccgccgtcggggacgcgaaggtctcgtcgccgttctccatctcgcccgccgaaaccctcgccgcttccctctctctctctctctctgtctgtctgtctctaggtcgcgccgcgtgtgccgtgcgctggatgcgagg

>TaeST2.50639.1

atgtttctgaaccgagatacacaattgttcagtggattttcttcatcacattacaaattagcttgcaaacaagtttgattcatccggggctcctggtaaactatggccaactcaagcatcacaaaggaaacaacaatccagagatgaagaagaccaaccagcaaacaaaatcagaattacgacatgctgacatagacaagtatcatctccaacaagacatgcaaataactttgttccacgattcagttcgaactacagagagagtaaatactgaacaagttggggttccatcaagctttcggaatttacagagcaacacaactgaagaacgcactggtaatatctgacccgcgcacgaacaaacatcaacagaccgacagattaggtccgaattcaagggtaaactgctgcaatttcgattccgtactactagtacagaagtacatccagacaacaaa

>TaeST2.50650.1

ctgatccaagaaactgaaagccgatcgatccatcgtaagatcagctaccggttaattaacacagttgattgctccgcttgatcataagagatggtcatcactcatcaagtctaacaacagtcttttgcattcagccctcgtactttacatcatgcatggttgtagtacaaacgaaacccagctgcatgcatgcatgcatgcatgcgcacacttgatcgcgtacgtacggtcgatttccagacactcgaccgtggctacaacacccagcagagcttacttgccgggcacgaagttggtg

>TaeST2.50705.1

gaaacaggggcggttggagccagtagagactcgtgtcaagaataacaagcgtggtttaggttccaaagaaccaaagccaaagccaaaggttgaggatgatgttgaaacggctcctagtaaaaggcccaagcagggcgcgccgacaaccaagaaggcaaagttggccgcaaagaggatacggaagatgcaggaggaggagaagcgagcacaagagagggaattcgaaattgctttcttcagggaattttggcctgataatgtgtaaaccacaaacctgacggcgttgcacctgttggcatgccattgtacagcttatcgatgcttctgcaatttgcacctgctgttcactgtttctgtcttgttctggtcgaagtgttgcattgatagacatatgttgtaagaaatgttctttgattgaagaaaatgccatttggcctccatgca

>TaeST2.50751.1

cgaggggctcgaattcgttagcaggggtcggagaagcagtgaaaacgcttggggacccgtagatcgccggtgtccgtgaagcagaggaagccggggaggaagacgactgcaacggccttggtccttctggctcctggccttgttggacaacgaacgagacgacgtcggcgagtcgatccggagcaaagacgacgacggaggagggatatggctgcggcagagccatggaagtgctccacgagctctccggcggaaggggcgacggctatggaggcgtggcttggagctacctcgcggcggcggcacagagaggagg

>TaeST2.50800.1

gttttgtttgcgaacagatacgcacgctatccgaatgcctagcgatatcgagcacacatgcttccaattgggccagcagttttttgcgttatggacggattgctcaatatcgggcccgaatgccacaactgaaagccaagaccaaccaaatggtggcattcgggatattaggcccagacaaatgccaatacgcaatacaaggcctgaatgcaaacatccaaacggagcgttagggt

>TaeST2.50811.1

atctcgccagagttcatcaaccaaacataccatagttcaccacacatagcataggggagttcttacaaaccataataattatagatagcataagggaaccgaaacatagcatatgggatctactagtcgccggagccgggggtgggcgggggtctacttcaccggggttctactcgtcggagtcagagtcgtcgctggagacgacctcggcggacagcggcttccctccgtgaatggtgccgtagatgcgttcgcggtagatccgggtttggagttggtactcctcccagagcgccggcagccgcgcagctacagccgcatctccttcttcggcgtcgatcctcgcctcaaaccacgcccactcggcctcaaactggcggtggatcgccgagtcggagagccggccgcagcg

>TaeST2.50821.1

tgaatccaaatggatccgttgaaaaccagcaccatccaatccaacaagaattttgtacttgaattgcattattattcataatgagtggacacaccagtactacagtgattgatgaaagtaattgcattatttataaaaaggccttggatcgatattgccgagacagagcggcatgcagacaggggagccacacatgaacacgtaagcagtacagtagtacactatacataatatagcgcgcacacatacaagacaggacacgaaacaacaacaacttattcatcgtctctctccagacttgcagaggcagaggcagaggcagaggcagagatcatctcacttaccaattctctcgggtactcctcgatcgtctccgggctgggcggtgggtcttagacttagatttagagatttagagtgact

>TaeST2.50829.1

tccataaatcacaaaatcctgattgaaaaccaaatccccaatctccaatcttctataaatcacaaaaaactgcttgaaaaccaaaccccagatcgtcccttccaaggcctgggggtgcccagtacgggaacttccaggaaaacaaacagggggggccacaaagtgaatggatcacaaaagggcgtcatataatttgcttagagatactgaaccttctaagatgattggaatatgatcctctactaaaataaggcagtaacaccagcaacaatgcatgcatatatgttgacatcacccgtccggtccgaacgaacagatatataaagacataacagcatgcgacgtcacgcaccatcagaaagatagcatagtgctaggtagccaaactgcagttgataattagagagtgaagaacaagctggggtgcgatccatgttgggctgtcaatttgcggcctccacttcagtggtggtttccgctgacgtcaactctccgaacttcatgaagctgagatcaacgaagg

>TaeST2.50839.1

cctgagctggcgatcgtacttggtcttgggctcggcgacggcggcggcggccatggctgcggaggcggtggtctctcttctgtggctgcggccgaggagcggaagtggaaccaggtggggaatcgcgacccttccctggcgtgaagctgcacgcgcgatgcggagatgggtgcagggcgttttgcaccagctcgtgcgtgcatccgattccaaggaacggacgg

>TaeST2.50873.1

attctagccaattgatggctttgttaattcaaagtcaggctaggcttgagccttttctcgggctcggtctgagccttccccttacaagaagtacatccgcaaataaacctaagcgacaaaattgagacagccaactttgattgaatagggaattgtttgacacgtacaatcaggctgtgctaaatgctaaagtggtaataccagttcttattttgcaacgtaaccccagtaaaattgagctgaaattctttgccagaggctgtgtgccgctatggagtcaggctgctcattttttcaacatgcttttaagcttttcggtagctgataccagttcagccgcaacgccaacttcattggccgagtgtcctgcatctggaaccacct

>TaeST2.50931.1

taccaaaacaaggcatacttattcatgttccttcttttgaaacaattgatgacacattagccaaaatgtctctcataaacttatgcgaataatagttcagtattcatcatccttccatcatcataaaataagaatacagttcatacagtcaaaacaaaattaaaagaactcttgacagaagtagccaacacttccaacaatgtt

>TaeST2.50948.1

agataaccaagagactacaggagccgtctgccgaggaatatgaggtccagtgcaggatggaggcgcttgttgccaagtgggataagttggcggagcttcacaaggaggagcttgagaaatcagcaccagatgcctttgagttgggtgtcaaggcttatggaaatgcggctaaatctgtggtccgtgtagctgccaagtcg

>TaeST2.50950.1

caggtcattgatctagcaacctctctagccaaagttgcggatgttgatagaaacctcggcaacgaggatgcggctgtcgagggttttgaggaagcaatcaagtgcctggagaagttgaagctagattctgagcaggctagcctagaacaacggcggcgctcggttctcgacttcctgcagaagcaattgcacaagtaagacagatggccccagtgtgtagtacccataacgccgttgttgtgttgcaatatggaaggagttgttgtggtgcataagggaggagggaggcgctctagaatggttgcgccctgtgtgtgtaattccaaccgcttgaattctgtcgcggcacaaacataaacaaggccgcgctgtacgtattctgggagagttctcttcaccgtcgccaatatacagtccaggctctagctagctccctgcggtcttcaccgtcagacaggggtaaggtgaaacttgtctgaagctccttgttgcatgtcggattggaccatgaccttgttgcatgtcgaattggaccatgaccccgtcgtatattaacaatagcacccaaaagatgcttcagtctgtctggcca

>TaeST2.50995.1

gcggcggcggcggcggcgcggcggtggcgcgaggtgcggttgcccattgtgtgtgaatgggtttgacgaagggagatcgaggcgatagggggctcggggtttgaagcagcagcagcgctgcgcaagcgtgggagttttgggggagagacgaccgcgtgatctgcgctgcggtgttggggtggggatcgatcgggtgcggaagggaagcgagttgccggggtgtggcggggaggttgtccgtccgt

>TaeST2.51002.1

ccaaaataagtgtcccgagtagtataagaacccaaaaccgacaccagattaacgtctcatgaaagcatttttcttccaacagggtgttacaatgggaccacgaacgaattacaccatcatatatacaccaatactctagtgcaaacttgtaagcgagtcgcgcggaggagtattcggatatcgtcaccacgcccataatgtcccgacaaactcatggctattgacgattacaatctagtagtggcagaaataagcatgtctacaaaagttttcatggctaatgacgattcggcggatatactagcagaaacaaacaagtctgcagaactctttttcctctgaaaacttgcatctgttgggatcactcacaaccatcagcagaattggtactgtagctccttgcaaaattctccggactccttctcttcttagcttcgtctgccaataa

>TaeST2.51108.1

aacatttcagattggacacgaaaagttccatgttccttggctttatgttattaatcgcaaaagctctgaagttccactgattgatttccatttaaaatacactggaaacgatttgcttggtgttacggctaaagtagtggacatgcctcaccattttgtggaacttcatcctgacataaagaagcatttctgggatccacagaattggccaaaacatgtacttgttagttatacatgggaggaacaatcagagatagatgttactgcaggattctatgtgttatttggatctggccttgttctttccttcatccttgcaatctatgtattgcagtcatctcaagaaaagttaacaaggtttgtgcgagaagcggtttctgatagcagtctacctgaaggaggggttgcaaaggttgagtgatgtttccccgaggggctgttggattatcgacaaatgttcactattggaaacaccgacaaagacattgtgaactcaagctaccaaatttcttgggagcgtggacaatagcatgcatctgaagaaccggtgaaccattttgtatgcatgcattgtacggaactggcgttacaacatggcgagttaaatgagagatgaattgtaggtttttcattggtataacagaacattttagcaaactggagtatgtgcagttgctagttcattacagttttgatatatgacctgtgtgatttcaatgttgcaagaattcttctt

>TaeST2.51127.1

ttgccgacggatattaaatcaaattttcttctaataactgcccacgtttcgaaatagacttgctcaacatgaaattatgtgtggtacaaaaccaatgtaatatttgaactaaaactaaccaagtactaactgcataagaatcaaatagcccataatttactgaatagtaccgagtacatcttaagcagtttctgagcaagtctgctgtgcctaagatggccgcaattgcaaatccacgtcactggtaagattcttctgctgagcatatcttccaatatcaaagtatgtgcttcatactggttcagctaatggcgacggaacaaaaatgatgtccacgtccatagctgtatttcatttactatccaatcccatcatacagctgcttaggctacattaggtaagagtttacaaactgaaacaggagacgacaaataacagggtatctcacagatcaaactgttaacatttgtacatctcaagatagcagacaaatatgaaaccaaatttggataaaataacagcctttgacatatataccatattctggttaaaaaataaaaatagggtctgtctatatcacatctagatgtgagatattatctcatacctaactcctcatccctaaaattcatgagactttaaaaaaaaatcaagcatgagagagttagatgtaagataatatctcacatctagatgtgaaatagcaaacctgataaaactatagtgaatctgataacacaacaatttggtctgtctggacagccggttgttattgaagtgcaaatttgagctgtgatgtgatagtagatgcaacagcgaattagacaaatgatttcactgcaaatttgagctgtgatgtgatagtagatgcaacagcgaattagacaaatgatttcactacagctacgaggaaaacttgaatgggttctagttcttctgtgtaaaagaactcagaacgactaaaatttatttccacatatgaagatcaacgagagagtgctttgtgtaagtttatgtatgcttcagctgtagattatcaattgtacctttggtcttgctcggcagccccaagatagattcgcgacggtagaacgttgtgccaggaagatcggatcggacgcgaggtgcagacaaaggagcacaccaggacacggtgggggagaccccggggctaaaacagaataacccccgtgcgatctccccccgggacacaagaaccatctttattacggtggcggcctgccatgcgagcgcacccaacatggcaaaggcctctagatgtcacatctctcttttttcactccttttttcctctcggcacgatcacctcgcttgttacatgctcttacctccgatctcttagatctaataatcatcgaaatatcatcggataattatca

>TaeST2.51147.1

cctggtgcgggcggcgatgggcgggtagtcgaagagcggcggcacgagcaccatgggcggcgggggctccggcttgcccaacaggctgctcagcagggagcccatcggggacgcgcgcgcggcggctgggagatccgagagccgaggcctcgtcggcggcggcgaatcgcgcgggggtttgttggtgggggaggagggaggagatcaacttgggggagaagaagaagagaaagaagagcccgttacggtgggacggcaagctgttg

>TaeST2.51151.1

agtctgtaccaaatccttacacaacggaggcacaagacggaaacttcccggcgtcggcggcggcggcggcctagagcctgagccaagctgggcgggctcgcacagcgaagatactgatccatctcctctccgatcgctcctaaccgtcccgtcgccgatggcgcagttcgtggcagattagatcggattcctcacctcttccccacttctcaggctcccagatcagctcgcggccgtcctcctccaccccccacccctctcgtgattcagcactagctcgacggtcgtggatcctcgcatccttcccggacggccgcttctggttccacaaacaggccaccacggttcttcgttcttcaggtatatcgaactgatcaagtgtataagagtccatggaggttgtcatctctctcccagcaggaagctcaattgcctggaagcactaacacgaggcaaaacaattgatattttcggtcttgtaccaacaagatgcttgttgagcgatgatattttctgtctttaccacttggtcttgccggccatatactaaaaatgatctgtatgcctcatgtactcaagtactcgaatgcttgctgagcactgataatttcagtccttaatttgctcgacttccagtttgacttgtaagtgtggttctacttgcctcttgtgacgtgttctcatgtactcaagtgctgaaatgcttgccgagcagctataatttcaatccttaaattgcta

>TaeST2.51160.1

tgtagactaaccatgtgaacacacaactagtgcaggttatactataccagcaaaatacaagttctgaatttgatcatcccatgaggccaagacactcggcaactagtcagtagatcagacagtttttcgatgatgacttttcgtccaaattcttggcaatattacaatcagacaatgtcaacaagacctagctggatcaagcaaaagagagacgatctaaaatctcgcatcaacatgtaggctcttccaaatttttgatttccatcttcgtggcagatgcggatgaaagaggggcacacaggagactgacgacaggggagagagggatcagagtcacccatcacccagacttgaatagcaagacagctttagaatcaatgtagaaatacacaaagtagttcgtctcgtgcgtcacgtaggaacc

>TaeST2.51309.1

ataagaaaaattagtctgctcttatggaggcgaaagttattcttctttgccttatggtgcttgtgcagctaggaaactccattgatgatcattgcgagatgcgaactattattctttcaagtccaaagtgcacaggctcaacttgccagacggcctgccaaaagatttggggacccgacgtcaagagagctgagtgtagggttgtcgaccatcacaagtattgtgactgcatcatctgttactagagttgcactagacggaaaatgttaggatccaataataaaatcttcatgtgacatgcatgcgagatcgtatattgatcaccaattttggatgttctattttattttgttgtgtccaatattttctcctcaatcatggcaataacctgaacgccagaagaaaaaaagttacatcctgatcc

>TaeST2.51320.1

ttaagtctaggggatcataacacatttttatatataaaccgactaaattttgtactaagaaatcaaacaaaaacaataccaagttatggccaaataatacttgtcacttccattggtgttttccatcgtctagatcccgctttaatgtgcgaaccatttcttccttacatagtcccatgtttgttttccattttagacctagccatttttttatcatctattcttctgatgcggatatgtctccatcagtttccttcactactttggatggcctccctcttggatgtcttatagttttggaggagtatgcttctttccctttagccttcatgtttctcttcctgatttcttcgttctcatctggtgaatctaagtctgtagaggtgttgctgtcctc

>TaeST2.51358.1

gattggttatttcaactctaacttgcttccatgtgaagtaccatagaacaacaatgcttccagcacaacagagttcaccataaacaacaatgaaagacaagtacataattttgtcaactaattattcaagggctgcacttgctcatcaccccaaagctgcacttggcaactgaacattccattagaagcagagcttaccataaaccacaatacaagacaagtaaataattttgccaattagttatttatttaagggccgcactacctcctggaccaccccaaagcggcacttgggacct

>TaeST2.51459.1

tagaaagaacacttttgcttaatttccagagacctacgtagacaacgttttcgttgagatggctgcaacttttcactctgtcatttgcaagatccttatcactgctgtactttgtgccgcgctccttttctcttcaggaacggcgcagtacatgtgcatgggcaaatgcgccgacatacccgactgcgacaactattgcaagacgaagggggtgtaccccaagggaggggtgtgcctgccccaacaccattactgctgctgctacatctagctgggtcggaggaccgccgcccctttcccgctttccccatgattttttgttatcggtcgtcgatggcacaatcgatgtcgtctaaataaaatggaaacacttttatgaaattttagggaactcgggacgcgcctgttttcttgtttcatgaaacgttttgtgaaatagctgtagctttcttggattgctgtttggattttacggtgccacaaacgggacggagttgtttgtg

>TaeST2.51550.1

tgcagcaacaacaacagaacggagagcaaagaaagaaagaaaaccagtagttagccccgaatttggatctcctgatggcaatcctcggaaggagcaggagcgccctgtgcttggcagccctagtgatcatggctacctgcaccgttttgtcgtcagctaatggaactcctggtacaatcagttgcatgccgccgactggcagctgcgacccggcgcactgcactaaattttgcgcgccgaactatcatggccgttgcgcggtgggcggcatagggggcgcatcttgttgctgctacaatccgaggagtgcgtcaattggggtggctcgtgatgctgctccctcacatgcatgaaggacgtttgcataaatcagcattcgatgtatggctcaaaattatcgaatggaataataataaacttctttatatacttttgtgagatatatcaaccagcaagtatttttattccaaggtgaagattgaatcatttagccaacatcaa

>TaeST2.51680.1

tacggtaattcggttgacaaattgacttgattaacttacttgattcataagaacctcttgtacgaggaaacgcaccgaaactacaaaatattgataaagcaacacgctcacaccctcacactcgcctgacacacacacgcacaaacggggcagccaatggcagaacaccaaacaatacaccccacacatgcaaatggatcataaatagcaacgacgtcgatcactcactga

>TaeST2.51914.1

ttcccagcgcaacttcctctccttctttcccatcccgaccggcggctagggttccgccggcgccgccgcagccaccggcgccatccctgcttgtccagcgttggccaccggagaaggtgggtgtcgacgccgtcgccgtcgccgcctccgacatcgacctcgaccgtgtctgcggcagcggcaacgacctccgcccgtaggtctccttctcgccaagcgagcgctcgggacgagcaggtgctgctccatccatcggttgcagctacagcacgagcccctctttagatagatggatggaattatgctggcagtcacctgcaggctaatgtgacgaggtgatgagatacacgaggggtgggagaaggactgcaggaggacatgccctgttttagatccacccgatcatttcaagtttttgcaaggagtttagattactctgaagacttcttttaggatcttgaattactctgagcagttcgcacaagagttgagagtatagtctgaacactggccatagtgtgccacgaccaccacttgcactacaggagcctcgcgtgtgagtctggtttcatggaaatgtgcactcaacaagtgtgattcggaggagactggatcgagctcacgagagggtcagtattgttcagg

>TaeST2.52215.1

tttttgtaacatgcatgacagttgcatgtattattatacaagaaagagaagtcttacagggtgaatacacgaccagaacagagtcctggtattattggatcaacataactgtgcaatctgtacgtgttcgtacatacatacatgcacctaccttcttattgaccacatatacgagtagttactacgtgcaggcaagtagcaacgcccgtatgtagcatctggcacttagttgcggacctccgcccgatacatgggctccgggatctcgttgggacttgcgcagcagcggcagccaccgcctccgcctcggccgcctccgtgcccacagcactggtaccggcagccgctgccgccgccgccgccgccgccgcccccgtggccagggtaaccgccgccacctccgccgccaccgtggccaggatacccgccgccgcctcctcctccgtggccagggtacccaccgcctccgccgccgccatggtagccaccgcctccgctgcctccgccgccgccatggtaaccctgaacaccggccttattagtctcttccttcttggcct

>TaeST2.52491.1

taaacccttgtaaggtaatctgtaatatactttattgccatcaggaagtatcatactggcatacattgtgaggcacagtacatttatcaatcaggtgaattctgatattctacatggtttgtgttctgaacattactcaaggaataagctctgctgttgattatggaagaacttgtgcattaagatttagaaggcacaaaaaaactgatctacattcttgcggtgaacatcattacaactttacaaggcttcggaacttccaatccttctttggaacagaatggatccaatcagtacagcaagtccggttattgcaaatgtgaggagcagttgcttgtgttttaaaccaaaagcggggcgtacttgcttgtgacgaccaccctcttctttttccagcatgga

>TaeST2.52530.1

agggtcaagcaaatgtctggaccagccccacacacgcaaatagatgccgatctcgctggtggaatgggaatgccggacaatggcttgaagctcatgtctacgtgaagctcgtgatagttgtttgatgtttttattgctgctgctatacatctgtaccaactttgcttgatttgtaacttgcaaatagccgctttggtccaagaggttaggattatacattgctgtatctgcctcatgcttgccagcttttatctaaattgagaacgagtcagctatctgcatctggaacaaattgccaatgatttagcagcagattatgtactatgaattggaactccgtagtggtattatcatcagcttgaatacaagccatctgtgtgcccttcatcgattacgttaa

>TaeST2.52652.1

tgaacgactgtgaacttgggactttgtgattagagtgacgaaataggacatcgatgatgcaaccagctgacatttgcatccaagaagacgatcacactaacatggtatttactagtattatgtaattgatgatacatgtagcacatcaaggaacgaagaatgcgagaacatgaaccggaaatagatacatacgacagttgatacagacgctcagtaccgaacatggtcatgagcaaaacgcccacttttttcttcccggcggtgctacctatagaatctaaagcgacaaggtgagctttagcttcttgtaagccagcgcatgagccagggatgatcatgggccaatagcagggatcccaggaatcgccaccagcgggccgttaatggtgctggttccggtagcggttccaatgagcgggagcttcacaggcaagtccaaagccttggactcgccgacgccaccacctgcaccggcaccgcgtgtcgctgcaaccttctcattgagctccctcccttcgcaggacgcgagcaccaccagtagcaggagcgcgacggcgagacactttgccatattgctaactgggaggctctgataggagatggtgttgtgtggatgtgtttgagtgagcttgggcttagcatcgtgtacgctgcttcttataggtgagtttcacgcgcgggg

>TaeST2.52680.1

tgttctttctgcgtgaccaaacatatcacttgttgcatgcttattcccatgaatctgttctttgagtagagattgcaggcagcgagcgcacgaattcaatgagggggagtgcgaggtctctagtggaaactgcaacccgaaaaatcaaacccggcaatggactcgcgaggttgatgacgattactactccccggaagcacgagaaggagatcaaccctactgagtctgtcctcccgaaggatgaaaaggtcgagcccctcgtcgcattcagccggccgcctcctttgccaccggttctaggccccttgatcgcgctctcgctttttcagactaattctggtgatgaagacagcaaatgagtcgccagggggcttgcttgtttgatcgctgagttggttacttctcagagcctggccgtgtatatactcggaggttgtagacatcatgttgctgtagtagtctggcaagtaggagtactacgaaatctgaataatgtactactagtagtcttgtgtgacgacctttcagactgtacaaatttcgaagtctgtcgctgtgcaatgttttattttcttgtgaattcatatgggctcgtttacaagttgcatcaaacgtgtcagaatagctacaacaacgc

>TaeST2.52799.1

gtgcgcacctgagacgccaatgtcgtagccgaagatgaggccgcaggaggcggccatgaggcaggtgaccaccacggagagcgtcacgcggccgccgccgtagccgcggacgcggtcgccgccggccgccgcggcgagacctcctctctccattttgtggctgctcgctgagtcttggatggtcgatgggttggggtttgcaaggcgagaagagggtcgccgctattatatatgggtttgacaagagtacaccgaa

>TaeST2.52849.1

aaagagatgcaaatttctaagaaataaaatagttccgaaaacaaaatttgtcagtggtcattcactgcagataagttctgtcagcgaccccaagagaaagaatgaggtagacatttgcttttgcacaatcaacagggatgtgaacttgaatagcgatgcttccgagaaggtaatcaatcagattcaagtaccataaaatggtctcccgtacaagtataacccaagtactgtgaaacatcagtttattacactaccgcagatcaaatgcggagccagtttctctgaccaaagcaaactgcagaaagcccggttgccgccaaacaaatagctaatcgtcgtcttcgtctgtag

>TaeST2.53075.1

tactccccttgtcccgtaatattacgggagggagtacttcacataagttcagaatttaatatttatcttggtgccatgttatcacacaacatctatttcctctacgaagagttgagaaaagaattcagtggatgccaaataatacacgaccaaaaagaaggatacaagtctgcagcagagcaggttcacagcgaagcatgcccaaatccagatacacactgtctgaataaataacgtaataactaaagcgagcgtgcaggggaatgaaatgcggaaatacggtacaaaagaatttactactgcacatagccactagtttccccagcaatgtttggttcccaaagtacacagagagagattattttgggtcttggatggccataatcttaggctgcacacagcctgaaacaaaaacctccgccactctataaacaggtgccattctggggctccagcaccagaacttattgtttccaccgctgcaagga

>TaeST2.53104.1

aatgcaggacaagctgagaaagattattggagtggaataaagggtggccaagggagattcaataacaataatagaagcaacaggggaggaagggactggaagaacaacagaggtggcggcggaaggcaccatggtggtgggaagcgaggtggtcgccactctgacaaccacgaaagggccaacaaagtccaaaagctcgattcgtctccttagatagcctggatcggcctataccatcttttagccctagtgaggaaaagaaagatatgtctccggtgcctgcctgctgctgcctccacagctttgggggcagcctttctgtgtctggtttaaattttacctgtgctttttaaagtctgaacggcgggtgtgatgagccatgttgacaatggatgaagagaaggaaaactttatatgcggctaaagtttgtgctcaacctgttacagggccgtggaaacggcgaccctgtgattttatctgtgttttttttgttgagaatcctgtgattttatctgtgtttgct

>TaeST2.53170.1

ggacttatacacaacaccaaaacaagttcttcattcgtaccagctactactagcttgcacttatgtaaaccagctccaagtgcacaactaagacgtcacatttaacaaaatataaggtagcactttaatccaagacgcaaaagcaatgtcccagtgcacagcttttacatcatgttgtcacgatcctaaccagtagtcatgccatcacgatggcatcgagagggtcaacaatcagccaggtcatgtcatcacgacggtagaacagcatcacgctcttcgtcttcgacttccagcccaccttatcaatgaagttcttccagcaacatattctcacacgtccatcatctgcagtgagcacattgtaggatcctacaacagtcacagctgggtggaatacaacagtaataggcccacttagcgccaaatcatctggaaccgcacacctag

>TaeST2.53204.1

acgtaaatccggtcgcagttcactttcattcacgataagaacgagttacgatgaacgggcatgaacacatcgacagagatagtactgtatacaatccaacaacagagtctgaacgaaggaaatctgaatatcgatcaatatatcaacaatggtcgaatagtatgtagaatcccaatataggccaattactagaaattgtatactgctaacaaagaaccaaaggaacagtaatcgaccgatcgatagtatgtgcaaagctaactagcagcagtagatgcgaaaatctctagcaagatctacatactaatcttcagaccacggcggcgacgcagagcagcagcatcccgcaaccaaccaaccaaccaatcgaatcgaaccagagagaagacatcacatcagatcccccaacgcaccaaggaatcgaatcgaatcgaatcgatggcggcggaatcagaaccagaccttgcactcggtgggcttgaatgcgggggcgccggcggtggcgccgggggcggggagggagagcgggcagctgatcctggcgcggggtccgggcaccttgaaggcccacaccttgacgcggagcttggc

>TaeST2.53277.1

aaaaactagccctccatccttgcatccacatgcaatcaactacatagtacagaagaaactaacagaaagtaacaatgcagcagcagcagcagcagcacgcaactgttaaacagcccctgcatgctatttgtcgccaaattaagcagatcgagcgagaaacacacacaccggccggagtttaccacaaacataatatatatgtgtgtgtgcggtcaacacatagctagagaaactctagatcgagtggtagtagagtagggagtttcattcatatagagaccatgcaatgcctgctgctaatccacacgtaccgtagcaataccatgtcttgcctgccgctaattaacctcattacaactacatatatacttagtttgcacgcatccagggtaagatctgg

>TaeST2.53342.1

ttgtaagcagaatctcatgtctgagatgaataaaaattagaacatctattaggcattaagtaaatcaccgcggaaatgagtaaatagaaccaacaacacgaaatcatcatttgccaatagaccagcggacataattcagttgttgcaggcggccgacgagccaacaaagcattggcaatgattcagcaatttgcatccaacagaagcactaatattactcgcagataaccagagtccacggacaagcactaagccaacagcaaggaactacatttgctactagatataggcgacgcaatctaagaggaggtgaacttggtgacggccttggtgccctcggagacggcgtgcttggcgagctcgccggggaggacgaggcggacggaggtct

>TaeST2.53353.1

caatgacccggccgtttggccaggtctgtaccctaccccattcctcaccagccgctagggacgctataatctccccagcacccgcgtccttcgccgactaacaaattccccaaatctcctccctcccggcatcgcatttcttgaccactagcaggtgatcggaagaggagtagggcgagatggggctcgcgttcgggaagctcttcagccggctcttcgccaagaaggagatgcgtatcctcatggtcggtcttgatgccgccgtgaaagaccaccatcctctacaagctgaagcttggagagatcgttaccactatcccaaccatcggt

>TaeST2.53435.1

ccggcgcagtcgaagtggcggtagcccgcgcggagggcggagtggatgaggccgcggacggcggcggggtccatccgccagacgccgagccccacggccggcatctcgtgcccgctgctcagcttcgtcgccgccgccatccctcgccggtaggtcgtcgatgagatgataagaagaccgacgacgagaaggaaacgcagtacggtacactcgagtcagcggccccttcgttgtgtagagggggaaggaagaaagattccgttggactctgctggtgctgggtgaactggtggcggaaaggtgaacacgtgggttttacggaaatcgc

>TaeST2.53449.1

tttcctccccgtgaaaaaaatattatttatttcccttcattcaattttttctgatggacagaaacatcaaagattggccttgattaatgacaacaatgagatctatgggcaaacatggaggcactcttcctgcgttggccagcaaggtagacccgacaatgttgcacagcagaattcgactttctcagggccgtgcctcctccctatgcacatttttgggaacggggtgtcgtctttgcctatgacatcgccatctgaaagacgaggaccacctgatctgccgatctcaccatctg

>TaeST2.53465.1

gcaggaagtggaatatggcaaggctgggaagccaaaggagcttactactactactaaagtgcagaaagtttagaagcaacttcattccaggttgtgtgtgtagtgaagcaaagttctatttgttagatcttgtcattggggcttcaagagtttttgcctctttatctgcctgatgaagtaggatctacatttgacacaagcggcacttagatcatggagtctgaagttattgttttttgttggctcctttgtgggtctgctgtaatctgaaacgcaaattgcgtatttgcagtggctaaatgttgcggttcaacatcttatcacattgtcttgcattgcatagattgcacggcataaatttccctcatcattttcacttta

>TaeST2.53479.1

gacccctatgacgtgctgctactcgaagaggatggtgaagaactgaagattcatgccccttaagagcagacaggaagaaggttctacctcatcaagggagctgcttgcagaagcgaggtagtggtgttcttggatgcgatggtgccgtggaggatggaggcgcggttggcagcgacggtgcgggcggcgctcctcggctggctcttcttctgcctcaccgtcgccagcgacgtcgcacgccaactcaggagcagcgatctctccgtctaagaccgaccgcgttcgacatggccggcggcgaagaaccgactctgcgtctacagggcaagaagattgatttgcagcagggtaaccagatgttattttgcggtgtgttggacccgcagttaggtgcaagtgctcgtctttagaagacggttgtccttaagactcagatgctccctgtaaattttcatctaaagcatgttgttcctttgcttat

>TaeST2.53636.1

gctctgcacaaaatcaggccaacaatttttcattcatgtctgaaagtgattacaatacgacttgatagtcgatacattcaggacttgcttggccaacaattttccattcatgtctgaaattgattacaatacgacttgatagtcgatacatccaggacttgcttagcccaaagcttcacccacaaacacacacacacaagagttcactaaccaatgttactcgtcggaaactgcaactttaacccaccaagacgcc

>TaeST2.53795.1

tcagcataatacagttagaaacaaggtctctcttctgatggcgcttctcaaaaccaagaacgcaatgcctttgtttatggtgaccatgatgatcatggctttgatcatgtcaccctgcttcggtcaaggaggcgacagtatatcatgtttagttgtttgcggaaagcttccaggatgcacccttgacaagtgcaaacattcatgtgttacgtcggggattaccaattttacagcaacttgtggcgatcttaaagagtttgaaagttgttgttgcaagtacaattgtaaacgggttcctcctgtttgaggatggttggatccgcaactttctttgcattatctaaaaataatgtacgaagataggaagtcagaccgatcatcattactgttgcactacatgttgtccgatcaattaaaacaatgtcaaaatacatctaatat

>TaeST2.53798.1

tgagacagatcaaagaagaccattgctatttcacgagataatgaaacagtctgttccaacacaaccagcacaaatcacaagaccaacatggtccgaacagatgacaattgcagagaaccatcgatccaccaggaagagaggaagcatgctatctaggaggaggtgaacttggtgaccgctttggtgccttcagagacggcgtgcttggcgagctcgccggggaggacgaggcggacggacgtctggatctccctggacgtgatggtgggcttcttgttgtac

>TaeST2.53804.1

agggaggttcattccagggtgctatactctggctgtctcagaggaacttccagaggagtatcaagggatgtgccaagacaacaacgtgcagtatttccctcccaagcgcccctgagcaggctcgaagttgatgcagtcatgatcctggagccgtactttacatatagttgcgcctgtaatgtctgttgattaaactgccgccagtgttatgcatccttgagcgatttgtcgaacgcttctgtataacctggagacattttaagctgctgtacaacctggagacattttaagctgaactagttcatcgcacatttctacttcgacgagacattgcaagctgtaagctgtgctagtttagttttatgtccgacacgtgtccaggttgctgttattatgcaccaaagctacgccaaaagctcctaaaattattgttattccga

>TaeST2.53808.1

tagagccaaaaagacgtttatatgcactttaagaaactttcacgtgttcatcatttctaggttggttaatgtgatttgatatcgattgatcatataactgaaattggttaaaatacatagaaccaacagtaatgttatctgaaaccccacatcgaccttgacactgtgcccttgtctggaaatgaactggaatatatcatagttgtagattggaaattcggactaagttaccagctcaccagcacagttttgctttcacttaagccaaaagccaaaaggaatagaaaagtacagtaacatccacgtgagatgtcactagttgatttttgaatggttgctcactgatgtagttaccctcagcttactggcgtcatcttgcaaatgctgcataattagttgctgctcccggtgatttcgttgtaagtgctcttgatgttgctgatcagagcttccctg

>TaeST2.53813.1

tgctgctgcgggggcggctgcgcgtagtggggcatcggcggcgggatcgcgccccagtgcggcggctgccctcccgcgccctgcgggggcatgggggcgcccatgcccggctgcggctgcgacggcggcggtggctgcatcatggcgaggggggaggggagacccgaaccctagggtgagagaggggagagggacgggagagaggagggatacgggggaggagatggggatttcagggctg

>TaeST2.53829.1

tttaccaccaacgtaggctgaaaccatcccgttttccaattaattgcatgaaatatgtacagaagcactagcatttgtatgtactacatccaccatccccaatttccaaaggtttacacatgtttgtcccccttccaaccaccaaacaacctcatctggaaacatcttgctgctaccaaatgcgctcaagcccatcaagatccactaacccaggtcgctctatccgtccccgcgagggccgccggtgcggcgtcgtgatcagctcgggccccagcagctgaggtacatggccttgcggagcgactcgtcggcgccggcctgcctccggccgtcgt

>TaeST2.53835.1

tgaaggacccaatgttgcattaatatcaaacatgaagtacacgtcattttacaaacagacccttttacaaacatacccaggactaactgcatataacacactggtccttatcttttacaaagaagacccaacaaatatataagaaaagcaatcgggtccagatcgctgggcggcgtcgagcttcttgcggcgcatccagatcgctggacggggcaggggccagccgaggcctgccggagcggggttggggacggcggaggcctgcagggttggggacgtcggaggcctgcggggcatagacgaaggggccggcggcggtgaggagggccgcagcggcgccaggggccggcggcggtgaggagggccggcggcgg

>TaeST2.53837.1

ttttcctttaaaacgaatgatatattggtaaagctgaaaaaaaaagaggccagatcgcaagggttgatatagccgcaacggtaccttgttaataattactctctcatgacacagcgtttatattcaggacgagatcataatctgtaaaaactgcacattctctatatcggtaagaaaagtggacaagtcctacaatgtcacttcatcacagcagtatcctatgccagcttaagattatagtcagagccaaggcaaacatatacttttgtttatggtgccatctaatccatggaattaatcttgagacctatcatcatctgagatatgtgacaatcaaaatgccgctctcatagccccccttgtcgcatcgcgcttcatctctgcggcctttccacttcctcggaagtacatgtacacttgctgcacgcatataatgccaaagcggctcagacatttcaattttca

>TaeST2.53841.1

ccggcgcgggcctggagcgcggcggcctgggcctcggtgtcggcgaaccgcgcgcgaggactcggacagcgcggggaggagggggcagcggcgaggaggtaggccgtggtgcggggaggatggcagcatgacgcggcggaggatggcgtggcggaggatcccgaccggcggcggcgaggcggggatccagatgggggaggagacgcggtcgctcgatttggg

>TaeST2.53926.1

ggccagatcggacagtggagctccaaggcgcccatggcgtcctctggtatatggttcctcaccggcgggtggtgctgccctggctctggctaccacggccacgcgctctccagggtcatcagcgccggggtggcggggcgacgccaagccagccagccagtcgtttgtgggtgcgctggattagataggaagaggaggatgtgggtgatgatgtgaggtggtggggatagagaacagagaagaggagcagaaccagcggctgggtgggtgatgacgataagaagca

>TaeST2.54052.1

aacgataacgagtttagtccatgatggatcaatatgcaaagcatttaagcaaggacacacaatgcaaatgcaagctctttccattacttagttgatgatgattcctagcttaactctcacgccgaagccgaaccgagctggagtatgtatgtatgtagatctaacagtggtaaaccgtggaatgtacacgaagagaagaggaaaatgtggagcaaaaaaaaaaacatgtgcattcacgacaaactctaatggccatcaggcacgtaagtgcagtgcacaagtaataagtaaccttgaggccagcagcaatgcagcatgcagctgacccttctcatggcttctcgagcaccagctgctgctcgtccgggccgacggagttgtgccgctcggagggggcggagggcgggatgagcctcgcgcgcggcagcccgtagccgcaggagcccatcgacggccgcctgttgccggccaagtcgacgatgcctgatcttgacccgtcgacggccaacaagacgccggcggccaagaggaagaagacgacgagggcggttgctcctgtgcaccgcatgcttcggcgcattgatttcaggggcgaggtttggtcaggatggatgta

>TaeST2.54158.1

gtgtgtcttgtagacggagcagaagctgattgccgtgtgccaggtggctgacgcgcaccagtgcctactgctcctactggctctcaagagtgcatccatatgtatgctcctactgccaacccgactagatgcctcccgcctcagacatacgtaacaagggaagacgagccgcgcatggagcctttggtatcccccttgtatgcctcaagtaaaacaaaggtgaagacttgacacgggatgcatgagtcatcgaaaaaatacaaggtggaggacagagccaccatatggttgcgtggtgattcatcttccactccgaagccttccttcaagtcgtgtgtttgttaaatattgccaatatgcccttccacacacaagattgcctttggcagacggagcagaagctgatcggtgtgtgccaggtggctgacccgcaccaatgcctactgctcctactggctctcaagagcgcatccatatgtacgctcctactgccgacatgactggatgcctcccgcctgaggcatacggcgagagatcattgtataaagaatagaaaagaaaatcctgaatgtcatggttgttaagccgagggagggagaattttggtatgctcaagggaagggttacctggagtcggttgtcaagctgaagagcctgggtctacacgttgtgatgatgagatcctaatttaattggtcacccagtgagtctttgctgcaatcttgtgcatgtgcggtacttgtgtattggaaacagatgtaacgtttacactttgttggcctccttgaagggcatcaatcatcgagtggttgcgctcgtctttttttttgttcgattgtccatgggatgcttttggagagttgttgttttccatttaattatatcgtcaatgaatctatgtataagatgttcagaattattggctgccatgttgctatgcctttgaaagatgcaatcaaaccgaatgtttcgttttttatgatctaaataaagaccttgtcttgttgat

>TaeST2.54167.1

aggtaccaacacctgggactggttgccagctacacgaacaacgtcaagcagatcacctccatcgccatcgagagttcaaggtgaccaactaccagtcgtcatcttaaagggagcaaaggacaagctactgcaggcaaaggaccctgtgaggatgcccaccaaggtgctcagcatcaccagcaggaagtccgtttgttgcaaagcacctcaagtgagcatggccatggggacggggactagaacaggatgtcgagcgacgacatgtctgccttcatcaacttgtgttcgtctttgtctttgatctgatggcacaccccaggaatccgttttggattgggtacaacagaggacaaggagttcattgtgccactgccaggaaaggtgcggtgctaggacttcatctctagcctcggtggcgtcttccgttggccccgctgtcgtgcggcgttggcgctgtttgcatctcatacaaaacagcccaag

>TaeST2.54375.1

aattcccctctctcgcctcctcacatcaaacatgaacagagggagagaaatccctaggtcgtcggcgtccgcggccggcgtgagcccgaccacggcgacgtttcaaggggcggagcgcggactggcgacgagctcatcgacaccaacaaccacgcggaggcgaagcaccctaggggcgaatcgagaggcgctcctgcaaccagaggtggggaaggaaaaggggaaggatgccggtgacgcgaaggacgacgagcggggtggcggcgagctgctacgactcctggggatagagaagaagcaaggtacaaggcaatagacatgttacaatttaagacaactgtttttcttctatccaacatccagtttgcagatgataggggggaggcaagaacagctgacaagtttttcaagtcaggttctgcaacagtttggtacagggggcaattttttcaagtagtagatcagggggtaagtacagagagggtaatcatctagttctagaactaaaagaagttcatcgtcaaaataattggaactgctttgtttacctttgttgttttcactgggagaaatttgcccagcactggcaatttttttgcccacaattcgtccagatctttgccgaacaaggtggaagagttgcagacattacgttttccagatttatttccagga

>TaeST2.54443.1

gttacatattatcttcacagttaagaggactgaagctatcagtatcagtagtgaagttaatcagccatttctatcctgactgcatgaactttatgttttcctcacaggtgattgacaaggttcaagaggaaacttctaagagtagatcagataggaagccaccaggtgttggccgcggaagaggaagaggagatataggcactaaacctgggggcagaggcatcggacgtggccaagatgatggcaaaggcggtggccgtggaaggggcggaattggaagtaaaggtggcaacaaaggcggacggggtcgtgggtgagtcggatcctgagatgcgagaatgccgctttttaaggcctgcagttgtggtgttgaatagattgcttaggacatgaaaaactttggagctggaatttctgggttttgtggtatggtatggcaaacggtttgtgtggtatgaagttaccaaggaagagttggctgttacctgtttgtgttgtggttcacttaatattttgggtatgggcattatgcgtactagtcaacatgcatttgcaaagttccagcccagattttacctatttgtttgttgttgagatatcgttatgtgatgtgaaagtgcagtttttatccatgacggtgctttaaaa

>TaeST2.54454.1

gacacatgcaactaaatctttacaaatcccatagaatcagcaaaaatacaaaaaagacgtcatggcaacttgccaaacgcaacacaagctatagccgagtcccctacgtcggagggggagatcatccacacactaatagaaaccaggcagcaaaccttgtagtacaagattttatccttcggaagcttcttggtgccattgtgcgttaatgactcaggaaataatatacctcaaacatttcgaatggcaaacttcacgtccggtaagttgcaccccctaccaggctacaaaggcatccaatactgtgaggaagcatccaactgcaccgtgccatcaatcacttgctacaataattcaggacacctttattcttgcttcatgtaggatttgcactctgcacacttgggagacccaagaagcggtctataaataccttggtccatccattccccagttcatcaaatacacagacagcttaagcttctgagccttccagtgccggaatagagcagcatataagaatggcatccctgaaggccgtgaagccaaccggacttgaagggcaggccaaggagcctaccaaggttagtgccaccaaggggcctgccaagctcagtgccacggcgaccaagccagcagccgctaagggcggcatcaagaaggctgagtcaaagccacgggagcctaagaagaggcaggtgaagagcaccaagccagcag

>TaeST2.54740.1

aattaaccaatcaattctgaacacatgcctttctctccgctgaacaccttaatccttgtgatgaatgaaaaccaattattgtctatacctgttacaaactgcatggcgtattcatcgcaagatgaaaaaaaaaaacaggctaatgtaggcgcgtttacagatcgaaaagaattaatccttttccatgcccttgtttgttggcgagtggaaatgggaatcttactgtactggatcgaccactcatgggatgggatcaaagggacgcgagtgtgctgatg

>TaeST2.54769.1

aatagtaagatcatgaaagctggaatttatagacaagcacaaccagatcactgcaaaatgtttttagccgaaatttacaagctgttttacacgatctcaacctacatttggcgaccaatctggacagctgaggtccgaaatctaaagcaccaagccaaccaatctcatcatctatgaaagaattgtgtgacattccctggaccaaatcctcgcaaagaaatactccgttccagcaccacccccctcagcgctcaagagcgcaccgaaatccaacaaagaatcttgaaatgagtagttgtagcaattgtggggagggggagacgtcaattacagggaaggggggcggaggcgcggcgcgtcagaggaagccgaggaggacggcggcgccgagcacgaagagccagacgatgtggacgagcaggagcgcctggccggagacgggggcggagccggcgccgacctcgcagtagccgcggcgcgccacgcgcgcgccggcgcaggcggcctcggggcagccgcaccagtaggtgacgccgtcggcgccgcagacggggtcggggcggaagcagcgcacggggcagag

>TaeST2.54923.1

ggctgatgaggctcccgaggtaggtctccggggacgccgccccggccggcggctgccgcgcgggggccgcggcggaggaggaggacggcggctgcgggggcgcttcggccgccatggatggccgccgcggcgggggtcgtcgctagggttagggtacggggcacgagcagggggagcaataggggaaaggagatggagagggagagg

>TaeST2.54969.1

agcagacacttttctattgacaccacatacaacttaggatgaggattccatctttaacgctactcctaaccctttcctttctcgtgctagcatcaaatgcggaaacagaaccaggttgtacggtcatatacttgaagaagtttggtgggtacttgcttggtggttgtgaggacgattgccacaagaagtataaggcgaccattcactgctctgatgacatggcgtgcggttggcatgccgatattgatggcagcaggtgtaagtgctcattgtgcatatactcctccaaggctccagcaaaggaaactatgatgtgaacttgcaaaataattgtcagtgcacatccgctaaaaacagatatgtcatagtatgtggtgtagcggcatgactatttcgccagagtttgtagaattatcaagtgatacatataggaggagttgaaattacgacatcgctttgtaataatttggcaatgtattaccaatataagttagagataacagccctcgacatgccaccattgggcttgcctttttt

>TaeST2.54984.1

gcacgcaagaaccatgcaaggaataaggcaaaacacaagtcacgcgcctcttctcttcatggtatctatcaaccagagcaacacaaggaggaccatggctatggctttcctggtccttataatgctgtctggttgtacactgccatcttgtcatgcacgtgttgttgttccgtattgcatggatgtgccccgaggatgcgctatcgctgattgcgtggacgaatgcaacaggatagagggacatgtgcgtgccgtattttgcaacgacgttggccagtgctgttgctacactctatagcatggccggagtacatagccgctttggttacttgtacgatgtagtccagatgaataagctcattgcatgtagtgcgcccgactatgagtcgcaaacataataagatgttccttgagaatgtcattagtgcagtggcatctgctgcggacttcatttgtttcacgcccaaacttttcgttcctaccgtgtagttttagagtctgaagaaccac

>TaeST2.54987.1

tataaagcaaccacaccaacatccatccacaagagaggtctcaggttcaaagttcaacaacaagaaggaaagaaaaacaacgtcatcccctgattgaataaatcaacgccgcacactagaagcaagaagcttattgatccggaggattacatcgcacaagtaaccaaaacggttgtgtccggtcaaactatgtaaaccaacaacagcactggccagggctggtgcaatacgtggaatgctgacgtccccgccccagcctgctgcacatatcgttgcaatcggaataattgcattgacctcctatcaaaaaggagcaatatcgggtaccacctggaggagatggaggaagatcatacgtatgcgctgcatggcaggacggtaaggtacaagcagacaccattacaaggaccaggacagcaatagccatggtcatcctcttgttgttcgggttgacgagggccatgaggagaagaggcgcgtgactattgtgtttgttcttctttctcgtgtgctcct

>TaeST2.55029.1

ataaggaaagccactgtagaaaaaaaaaataggacaccatttccgatggcatttatcaagaacaatacgagggccctatgcttgttagttcttctgatgtctgccactttttcatgtcatgcaactgggacaaataaaggtacgaatgcatggagggctttgtgtggagattttaagatatgcgagaacccaacagtgaacaatgttaatatttgcagacttaactgtgaatggtctggttacatcttcgatgacagctactgcgatcgtggtaagtgttgctgcgcattgacaaagccaaggaaatgatgggtactacgatgtgccaaaagtttgggcttgtaagatatgatgaatttcaataaagtatacaagttgatcttgatttgaatatgtaactccatgattgtaccatgaagtcgttattcaggttcgtttgatattgcaaacgggtttgctaaatcttaagtcacctgagaattccctagcagtcagttgatggctacaagattttattgcactggatgaacaaattagtacgagatgctaaa

>TaeST2.55182.1

ggatttcttatgcatatgaatctataattatccatcatcaaaattgaatgaatccagatacatacatcatctttacaatacaaaaactcgttaacatcctcccaacagcagataacttatatatcatgcccaaaatgaaagaacagaaataagccaccaacatctggtggtctgaaaataaatgctagtgtacaacacgccccttacaatctttgctgctgctgctgctgtctagaagcattggagatcaccagaatttgcaaattggatgtgtctactccagtatctggtgatatggcaataatttatagtatacaacacttccccctaggtaatttttataccacgatcaagaagcttaagatgaccagactttgcaaatgctgtgtctattcctatgcctgatttaagtcgaggtaagttctac

>TaeST2.55270.1

ttcagctgcaagtggtttttcccaatcagcttcgcgtgcccgcacccaatcacccgtgtctcaccccactcccaccccagccgccaaacaaaccctagccctctttctccttcgccttaatcccctccctcgatccccatgatcctctcctagcccgcagccaccggccttgtctctccactcgccttctcctttcctctaccatgtcatcgttgatcccaccgccgaccttctcctctgcttgctcctccggtcctcttccatgtcgccgtcgatccagatcggacgtaggtcatcattgtgggagaccagcggcggcggggtcttcgcggtgcgtgtgcggatgtaggggaggggtgggatgcggacggcagcgaggaaggcaagcaacaacggggaaggatcaggtgagcgatcctagcgtggggaaggggtagggtgaggcctcgcacacctgccggcggccgacgggtcgacttgacgcgtcaccgtattgtcgcctcctctgcgccatgagaggcgcttcccctacgcgatgagagattgtttaacattggagaatctgatttggtgttcagaagaagaaaaaaggcatttgctagtttcaacaaatgatgacctttactggatgaaggtgagatgatagttgtatttccacaagcacattatctagagctgatatttggattaaacaacaaacttgctaaagccagtcaagtgctcagtgacaattagcttgtgtactgattctttagtacctggttagatctctgtgttgatgaaggggaagattgttggtccagaaggacatttcattatttacgaaattgcatacaatgcattggataaatccatgagtttgaagctcaaagaatatgttttgaaggtataaggggagttgcgtccctggtagaggtgcagatcaaggaggtcggcaaatgggatgaggttgggtgatgctaaggaggaaaaaggtgcatgtccctgttttttggtgatggcagcggcatacttccttcagtgaaggaaggaatcccctgccggaagcttggcgcagattttgcctattctctcagatggatgatgatgagcggtgcaaccaaaaattcagagctggtctttacacacttcagtaggagtagcatgacaacaccaaggtagagacctgaatagaagtaaagggatggaccaaggaaaatagaaccgcactgtcgtgatgtgggtcgtgtcgtcttcgtccatctccatcgagggatatatacataggttaggtataagaaaggagacgtctccatggaactctgactgatgtgtaatgtcctcataggacctgaccgtttacagagggagtacttgaaaatagattctttatatattgtttggttccatcagccgtataacctaaattagcaattaggtatctaaacagtgtagtgtaaccattgtgcttgctgcagttttactttagtgttgaaatgtttttgttagtttgttctgta

>TaeST2.55288.1

ttgccacaaaaaaatagaattttttaagctttttaaaaatcaggttttactgttcacctgagctcatatgagctcgagtaatccgcgtcccctgcagtgctagtatcacaatccagtttgcagaacagtctgcaccgtaggaaacgaacagcagaagatgtcattcattgtccacttgcctgcaatacatgcaacatgctacatcttgaaaacaaatcacgagcattgccgacatcgaacatcggtacaacatatccacgttacacaacacatcggtcaccaaaccaaacatagcttgttttcagaacataaagctgggccatagtgccttccttcggggaaatccaaccatgcggctgctaccttcttcaaatttcaaaggctttcagggggtccttggggtcaatgtcatggaagagagggagatcggaccggaggctgtgctggctgacaccaacactttccagcgtcttcagcacctcgtcgaacaaaattttgttcccggacggggtgaaatgtagcccgtcactgcatgatgtaacaaagttatctgctgctttcggtcaggaagttacat

>TaeST2.55319.1

gtcagcagaaacagcgacacaaataaaagctcaaggagcggctgacgcgtacgtaagtagagtatacgtacattattgcgagtacatgcatacatacatactggatgttcatcacacagagggacagggcacgaacagcgcacacgctgtgctgattttgtgctaggtcgtgacgtgcagcgtctctctcagtcctcgatcacaaaggttacgccgtcctgcttcttgaagtagtgcgtggtgtgatcaacgac

>TaeST2.55343.1

tttcttttatctgatattttttcattcaaaacaatcatccatgatccaaaacctgtaaataacatccaggttcttcgcccatgcacatgattgcatatcaaaatattttctgtcaagcgatgatcagtgattttttttacataataggaattaataaacaatgaactaaacctgcttgaatactaagatatttctaacaccatcaggttagaaaaggataatgtaatcatttttaaacacatcatgatggctccatgtccagataaatctttgtggcagcaacaatacaatagaggcgacagaaaataaaataaaatgattgttcctccgttgatgttcgtcatggtgccgaggaacatttccatcctgagaaagacgtcacaaatgaacgtttggaacataaatgtattgaatacaggatgaggcttaggatgatcagttgactggaaaaaaattcatagtgtcattgtgtcactcatatatggaggaaacattgttcgtttattttggaagatgtatcgacaataaatagagaatagctcaagaagtctgagccgttgcctgaagtgctgtgtcaagaaaatgactaacggatactgcatatgttaaaagcaagagccaaaagacggtggtcaaaaattgaattagattatgatcaagtaaatgctacatgttccacatagcaaataggtccaattattaaaaaaattaacaaagcatacataatgtggcgaagaacctgaaacaattacaattttaaacttctatacataattggaggtgcttaactaaaccttggctggagattttgtgtcgagaaaacccttcaatttgttccagatcaacttgaacacactgcccacatacacaacaaaaatatgatgcaaggtctgcaattttaaattaaaattattactgcactacaccataatacttcttttaaactatcataattctaaacaaactattcagtatctacaactaagtaaaataaccgccgataatagtcgatctttatcttctgcatatgtacatatatgtcgtatatttgttcttacaatgcttgccaatgttggggatcaacactggtcacatccatgccaaggtgggcttcaggtgagaaagagatgcaagtggcaacgtcctctacctctactccggggcgaccctccttgtgcatcctcttgcttgatggtatcctttgactacaaatcatccaaatctctccacctctgggcttcatttactacagagaaatggagttaagagggggctcaattttagacaccaatcttactaagggataacatcattatggaagtaggaaggagccttggcgaataatagatatgtttgaaataaatagaccaaacatgctaattacaagaggaagaacatgttgatccatctacgactcatagtgacctaacgcctaatgttataacatcattatgaaagtaggcaggcgccctggtgaataatagatatgtttgaaattaaatagaccaaacatgctaattagcagcgggagaatatgttgattcatatatgaattctaatgaccttacatgatgctcagcgccatcgagcgaccatcaataaaaattatagttctccatattagtaatctaacaccaagaaaatgaataagcagtcataatcatttgtcaacattattttctcttctaattttgtagaaaaggatagaagttttgttccagaaccataaataggaaagagaaagaaatactaaaatatagggaacttaactatttgtcaaatcatgagttatgtacataaagaagtaggcacaaaattaaccttggaagaggattactcttcctgtttctgggcccaacaccttaatagtgtacagtgtaccataagagcatcagtaagtgacacaactactctgcctacatacatgataaagaatttcaatgccatatgtgtgtgctgaaaggaaaaaaaggaaataaaaaatactgcatccgaaccatattgactactaaaagggtaggcacaagctttgctaaatttaccacaaatgtgatatattgtgctataattaaatgggtgcaatctgacatttgccggcatcacagatcatccatgcttctagttacatctataagtggccaaaaggaatgacaaactttatttagcttgtaatatgtgattccaggaaacagaagttgaatatatatataagcaatcaacaaagaaatgagatgctatacgtgaataatatacaagtaagaagatgctatatcaatggaagggcatatgggcacatcaagggagagagttcctcagtcatataaatcagcaggtatgaggcataaacatgcatcttgtatgtatataaacaaaacaatatgtaaaaagagttttttctgggccatcagaaataatgcattctaagcatcagatgttacctagcaggaaaaaataatccattcgacaacaaacaatgcctagattgcatcaaaccgaacccatgcataaaaaggttaacagaaagcaagctgaaggcttgttacctcaaaatcatctcctacatagcttaatatctacagcccatctccaaatgattcctacaatattgcaaaccttgccaattgggcatcatccccacattcttaaatatctatatacgtgaattaaggtcagttggtttgagcacacaactgtaatatgatacagtgcatagcaagatagaacagaaaatgtcaaaaaggcagaataaacttgcaaagaggctaccaacataacaatagggattcttttttaccaaaagcaaaagcagcaagctgcaccttgctccatgaaaattagggctcat

>TaeST2.55355.1

gccatggcgccgaaagcaagaaaagctcgaccctttctcgcgactctctagcttcctcctcctcctctcctttttggtgtgctcggctcggggttgggttctctcctctcggtggggaatttgtgtgggttataatggtcggcctccctcggcgctttagcggttcggccttgtggagtggagcgtagtggtggaggccggtcgtcgtcgtcgtcgtcgtctcgtcttctttttcctctgctctcctctcagtgtgtgtgtgtattcaagtagcaggtagctacgtacctaagcggcagaatattgagctggtggctact

>TaeST2.55616.1

aaacataaatcagaacaacactcgaatagtaaagtgcatcggttagtctgctcatggaacacgagctgaacatcaaattatttcgtatagaagcgcctgcaacaggacagcccttgtaagatttctcatttatgttacttgctactccctccgtcccgaaatacttgtcatcaaaataaataaaaggggatgtatctagatgtattctagttctagatacacctctttttgtccattttgatgacaag

>TaeST2.55620.1

cagatggggaaggcttttaggtgaggtcagtataatgctcatttgtcaaggcttgatgacacgacttcagatttaggtcagaatgccaacatgtttagtttgtcggcgttgaagtcgtcgtgcgagcaatgtggtgatgccacaggacaatcttcatgttggcggcggcggcaacggcagcgaggtgtaagccttacgaacttgagatcagttctatattgaagagcatgcacctgtgttggcgaagagctcgtcggcgtggccgagactggcgcctcgggggctgcacctgtgttggcgaagagctcgtcgtcgtggccgtcgccgggtgactcagtggttgcatttgtgctggcgaagagctcgtcgtggcatccatggactggcaccttggcggatgcacctgtgttggcgaagagctcggctcgtggccgtcgtcgggtgactcggcggctgcgcctgtgttggcgaagagctcgtcgtcgcggccgtagatgggtgtctcgatggctgcgcctgtgttgtcaaagagctcgtcgtcacggccgtgggacgggtgactcggcggttgcacttgtgctagcgaaaagatcgtcgtcgcggccgtcgttgggtgccccggcgtctgcgcacgtcatgctggacgcctggaatgattccccctaatttagcagatttctttccatattgttgacccattccatgcgaattggacgggctgtttccttgtgcaagcatgggatccgtactacttgtacgggcgtgggaaaggtagagttttcgtccgccaggaaaaaaatgctaaatgcactctggacgtgggtttgtgtaatctcgacggttagatcaaatcaagttatctaatcacttggttgtaatttctctatgttgttctgtgtacattaggctgggtgtatttagagatgaatatgtttgcttgtttactagtagtagagattggtgttaaacctaatgtattcca

>TaeST2.55709.1

gaggcacaagcatctcgtttgtaccctagtctggacgtttataagtttgcaaccaaataatgaagaacgggacaatcttacttttcgctcttgcgctcctcgtgctatcttcagatatggcaagctcgctccaatgcaggtttgtctacaaagatggcccatgtcaaatcaatgaatgtaaagcttactgtgataagattcctagagtttcgcccttccctgaaaatccagatgtgtgcattcctcgaggatgcaagtgtgtagtatgtggaggagtgcaggcagggatatagtagttttcttcaaggctgacaaagtataataataatgatgtcacaactcacatgctttggcttgtgtatatcagggtcaatatttatgacagaatgatgaaaaactgcagtcatattacaccagactttgacttatgaacgtga

>TaeST2.55721.1

gtgcatctacaacagtttgtgaacaaagttatccttgaaatacagctggccatcagtgtttagattatttcatcaaatgagacagccttgatgaatcataaaagttatttgatcaaatacatacaagttctgaaggaatggcgtgcacaaatagcatcaatctgttctccctagctaagcctaataatggtatcaaaacatgttcaattaaacgctaaacgttgcagtttgcttgcttcgagcttctggttaaaggaaacaaccattgcacgacatgaccttgtagtcgacggcggtgatgggcttgtcggcggggccaaggatgacctagaagggccacaacttcatgtcctgcct

>TaeST2.55772.1

cctcagatccttgagctgtaaaggcggatggccgactcccacgtagccgtgttgttgatccagagagctacagcgccgattcgctacagagatgcaatcacagcgcgctgccgctggaactcataatttggatccgggcgttggtgtttcaaggctggcggccggagatggagatggagatggagagggatagggatagggacagccgacagggagttctcgaggaagaggagatggagatcgagggacagaggtgaatcgcccagtttttcgactaaccctagctcagcctctcgccctggttcattggtacaggtacagaaaagaacagaggaaag

>TaeST2.55778.1

tttgtctgaagatataaacagtttcgtcagttaactgaaacagtgcgctaaacagttggctgaaactgaaattcagaattgaaacagagagacatatctgcaagttcagggtcgttgccgcagagatttccagagaagagcgagcttgcaccacagatcaaatcgaatcgaaccaagacaaagtagcccaaacaagatcataacaagaactgaaataaccgctgttactatttctccagatcatgagaagtgatcccacacacacagcacaagttacacaacacggcagagcacgaccagctaataactacagagcaacagcatcgaccgacacatcaaaggagacacacgggggacgggcagatcaggcggaggtgaacttggtgacggccttggtgccctcggagacggcgtgcttggcgagctcgccggggaggacgaggcggacggaggtctggatctccc

>TaeST2.55790.1

aggcaggcaaatcagctccttacagaattcttctctgttgccgcacgagccatgaagagagctttctcttcttttgccatggcaccagccattgcaatcctagctatcttattcatatgctgctccctgccatgctcagccgctgtccaagctcatacggaaggaaaccgtcatggagtgagtcctccggcaccgtctgcagcaccgggcctaggtaatcaaggccgtggacgctccagcaccccaccgccagtgccgcaccgcaagaacttacagctacagcaggctgttgttcccgtgccaccgatgccatgaattatcttgcacaagtcttgtaaacttcatcgttttaatttttgtcagtgctctgtggtgccagtgtcactgatatgttcctagagtccagaaataagtatggtagtgatggatgtacccttttctacaattataaactttaattaaaaaaatacatgataattaatcctatggaaccgcctagc

>TaeST2.55817.1

cgaggtccgataggacaagaagacgaagccggaagcgcgacgacagaggtgcgaggaggtgatgatggcagaggtgatcgggtccgtggagattaggaagcagagcagacgacatcgaggaacttggcgttccgcggcgatgaagaggaagaagacggcaacgtggacgtggacgacggcgagctgctgatctccggcgtgccgcgaagacgacgaaggggaaggagctccgggtccgtgtggtcgcgcgcgtagccgtggaagacgtccgcaacgcttccatggacgaagaagacggctatgacgatgatcaagacgggaggagcttaatctctcctcggccatggcatcggcgggagcgtgggcggacttaccttgcggcggctagacgaggaggaggg

>TaeST2.55871.1

tggcgtccagtataaatgcatcagatcaaataacagaaaaagagcaaatgatgttgctgaatgaagacacaaaattgttcagtcactggcgttcttccacattacaaattagactgcaaacaagtacagtactacgactcatccaggccccctggtaaattatggcaaatcaactcatcgccaccccaatagcagagaccaaccagcgagacaaaatcagaaacacggtatcagcagagacaagtttcatttccaatcacatatgcgaataactttcgttcgccgattcagttcgaactacagaggaaacggcgaactacacaagtcggggtttcgtcaggcttctggaatctgcacgcggcgacgaagagtgacgaccgtcggccggccctagccgccgaagccgtagagggtgcggccctggcgcttgagggcgtagacgacgtccatggcggtgacggtcttgcggcgggcgtgct

>TaeST2.55944.1

ttttggggatatccagcagaaatttcaacctgtcgttgcacataagcctcagcagtacaggcattcattgaagtttgtactactgatgtagagcaaaaaatccagaaattttaacaagaccggctagaatgcctcaagttgaaacccctctattccagttaaactgcatcagcacaaacaaagatccatggtaaaaacaatttccagtagatattcacctggtctgtattcctaggaaacctagaggtgggcagattcccaacagataaattgataatccgaccttgtacagcaaataacgtacaaaggttaaatggcaacggacaatttgtttcacagttaacttgggccatctatttctattcctcttaaggacccacaagtttcctgtgcacgtgtgcatggagagaatgatcgtcaccatcatggtggtggaacacatatgtcagaatcccaacaaggcacagcactgagccaactaggaccttgtcatagcgttccacccagtgaaacttgatctggctagcaccataaaatgagagggccaccaaggacgtcatcaccgtgatggtactg

>TaeST2.56095.1

tgcaacccgtggttttggccttgtgtgccgcgatactgctggtgctggtcgtaacagcggtgagcgcgtgcgtgagaaagaagaggggcaccgggaatgcggcagcggcagcatcaggtcagcagacgaacgctgctgcaaactgagggagatggaagggagtcaccagtcaccaccgccacctttggagtcatatacgaccaccctgctcatctgtttcgccgcgtgcggatgagatcgatcgtcgatgttttgtgtgtgtgtgtttcagatccatgcttccgtagctagtagctgaactagtagtgtggccagggattggccactgcattgctctgtcgtcctgggtgataccaacatgaacccatgtactgattgtcaactttatgtcctagatcaatttcagtggctagccatgtgtgtgcctcttcggtccttgagctgcaggcgagatttagag

>TaeST2.56146.1

aagttctagtcaccgagtaacagacaacagagaagataacacgaccacaatcggtcgaacccgagattacatagctcacccacataatgtacgagtggctcgccggccaaatacatatttatattcagaagattcagacatgatcagtaggcggcagcagctccagcaaacttcctgaatagcctggccgcgtccacggtgtaattcgaaacagtgacatggtcgtcatcctcgttggctgcttcatccaggaagcagtgcgcgggcgcctgccactggtcgtcgtcttccagcaccacgcccacctcgaagctcatcacgaactgctcaatccccgtcac

>TaeST2.56200.1

ttgtttgaaagatatgactttttgaaaatttataaatccgaataaatgcacatgcacatccaacagacttggaattaaatgcgctgtgcccatcctattagtacttgccacgtcacgagtaaagacaaatttcaaagtaagaaactacatgcatgtttgtgtgagagccggctcgatttcaccatatagtgcgtacacactttttagatttcaggataattattaattgacgctggattagtataaagtagaaatatgggtcatagacagcaaggcagcctgaaagcgaactcatatatcaacatcccctccggagctgggtgaatcgccatcactgccgtcactaccactggtgctgctcccgctcccgctgccgctgccgctcccactgccgctgccactgctgcttccacttgaactagaggtggcctgctccacctgctgtccttctgcattgacttgagctcgcagggcctcagctgcattaagtccctcctcagcttcgtcatttacatcaacatcagcaacctcatcatccatgtcgagctgatttggtaagttgatgtcaaaaccacgataagacgtcccttgtgctgaggccatattattgggtgaaccattgtcatcatcaccgagtatgtctagattttcaaattggtcattgggatcaggcgaagaggaaaatgggtctgcggtgactggttgatatgtagagctcctgttactgtttcttggtcctg

>TaeST2.56234.1

gaccgaccaatgctactgcattaaagaagaaaaggaactaatgaacacccatacacataacagcattctggtataatcatttaggccaattgacatattattagggataacacagggagagcaaggtatgtgagccgaatgacttcagaacaataagagtgatgatgcccgaacctaacaaagagaaatcctacaaaagagtaccatcaagtgggccagtccaaaccatcaagtgggccagttattggttggacgttcatgagaatttatcagttatctgcggcatccaaattactccagccaataactgataactgataacacaactattcgatgccccaagcatgattcggcccaataacagcgcataaattctccaagtggacactttgaaaacaaatcaactttattgcactttcctggcttctgctgctggcttccaaagaccaaggcggtacttgtgggcaaaactcaaaatcaacctcctctgc

>TaeST2.56254.1

tgttatgcattcactttattttcatcatctctcacggttgttatgtgtagtaaaataactgggagttttttttgagcatcacctgggagaataacttttttttgggagaataattataagtacaatccatatactagaagtgtacatggtacaacgtacaatacagcctcgtgtctcctcaatgggattgaaagtaaaaacaaccatatagtacgatagtggccggccatgcagagctggctcagcgtacacactcctttctccctcacatggagatcagatcagagcgttcattcatatgtatttgtgaccttaacatactcagcaacgatggatggcg

>TaeST2.56378.1

tagcatgcggtaaatgtcagaaagaagcttcccaacattgcatttatttggacacaaacatgtacacatacagaaaactagacagcgtccattacccaagcatccaacgcagaaaaccagggaagaacacaacaggtacacatacattggggaagcccggccttacagaacaagagaggttcataaaaatacatctcatgcatgcacacagcaatagttcagtctacttgcagtcgacaacataaaacatcacacgacgcaaaccagcaaagaccccgccacctgagagaacccggcgctaaagcctgccggcaaagccagtacatgatatgcgcgcggtcgtgcggtggatgctggatacagagttgcttcactcagatacgcagggatcgcctaggtacacaagtctcgtggtttccttgcgctccggttggatgccatttgcttcagttttct

>TaeST2.56401.1

tttcgaaaactgaatcttttattactactagctgttgctcgatcgccacatctctacggcaccaatttactcgatgcaatgcttggtagtagtactagtacaaagatgatgatgatacaatagacaaccgaatacaattttgttgatactggtagtagtagtagtagtagtagaagtactgtctgctaagaactattgtattttcacacgcccggcacaaaaatgtcatgtttcctgtatttatttgccctcctaccacaaattaaggcaagaaatgaagagattttgcatcatcaaacatgtaaggattacattctcgggaacctgctacgccggattgagagccaaccggattgggcgtgctgttggtgttgttggatcggatcggatgagatgagcagctctatgactgggtctcggtcttgcgctcgccgtgcgagtcgcgctcctgcagcagcgtgcccgcctcctcgtccgcctgcgccacctt

>TaeST2.56449.1

gtataagaacaagaagaaacattttcatttccattgctaatggcaacaagaaggtaccatcagattaggcacaacacatccaccatcagattaggcacaacacatccaccatgacaggaacagcctaacagggatctaacacgacgcacattctgaaacactattttccaccgataaactgaggatgtaacaggacgcatggattgcagcagcctaggccctctcgccacggatgcggcgggcgagctggatgtccttgggcatgatggtgacgcgcttggcgtggatggcgcagaggttgg

>TaeST2.56457.1

taaagcttccagagaccattttcatttttgtttctttttgtgaatcagatgtgtatagacatgtttgttcactcatgtcaacccgaatatactccatattgaaatatgcaaaacatcttatatttgtgaacggggggattacctgaaccctaagttatagacaaaatttcgcacgagggatcaaaacagcttttgagatttcagaatggatcgaacaaatggagcttccgtcttatttctacaagacggccaaaaacagaaaatgacatacaacatcacagggcaactgacatttctttacatgaacatatataccctagtaatgtcttagatacttgtggtagcatcccacaaacgaagaagtccattccggccaccactgcatattcttttcctatccaggtccgatgctatacatctcacc

>TaeST2.56471.1

ccgaaaatggtttattctgcttccgttaacattccggtcgtctcttgcgcgacatatttggttgaaatcacctagagccaaccacttggccctcactggttgctttccccaagagttttttggtgccaaccgtaacaagagtgagatcaaaagtagcatgtgcaagtatgaaactagtttaacggaagttttaaccttagaccacaagaaagtgttgtcatcctattcatgcttctactgaaggttggcatgtttaagagctgccggtgtacaatctctgaaacattgcaacgcccatgatcattcactcatgactagccgaaggtgcagagggttcctccgacttcggcttggcgaacgccttctgtttctccagtgtggctgtagagggttcctctggcttcggtttgatgaatgatttctgcctctcaagtttaggaatggatgccaccgtgttcttctggagagcatcagcacttgccttattgtctggctgatcccgagggctcactagctgcttctgaaggacatcaaactttgctgtcgcccagggagcggtgcctacattcccaccagaacttggagtatcgaccgtcagcgatgagatggcatctcttgacgaaaaccatgtgtaaccacaggaaatacattcaagctgaaaaaaggaggcaaattctatgagacagttgtggcaaattcaggcttattac

>TaeST2.56526.1

cgagcgggggtgtcagcttgcagcatcttgtaaaactcgtccgcacaaaggagagaggctgaaaataggatacactgaaagaggaaccgatacatcctgagagcgagcggcgtctttgccaatgggggagacggcggcgctgcacatgcgatggagatccacgacctaaaagccccacgccatctgggtgcgccgtcgccttccaggcacagatgccggcgacctcttcttcaccatcactgtctgcccatcccaggctccgacctacccaccatctcaagcaggtcaagatccgccacggctcccgcagcaacgcgtcggcagaccaagcaggcgcgcagttgcatgaggtgtggttcctcttccactctcatggtcacaattccgcttccttccctccctggctcagcagcggcagcat

>TaeST2.56630.1

tttttgttgccaaatttcctccaagtctgtctccccttcctccccgagggtcccgactcctgacctacatctctaccgccggccgtacccccgcgtcaccgccgacgccctcgatcgtcccccctcccaaccccctccgccgtctccgttcccgttcctcctcgtcgcgtccctgctgtctccgccccgttcgcgattttgggcattggggggttttcagtgatttcagctctaccataatcatgagtctagcctgtctcgtatgccatggcatgagcagcccttcacagtctttcagaagctattcagtctcaagctcagaggaggaaaacaggtgtggagctgctgttgcctgcctatcgcggaaaattttggctgcaggacctgctaaccgtgtgggaacgtcaaaggtgacacctgtaatggccactggacaaggcggcatcgagggttctcctcgccttcaacggagccgtgctgtttcaagggatcttgtcagggactggaacttcgatgagatcgttattgggaactagagtagtaggttgccagcagttatccccaggctggacattggactattatctggcaggagttgatgtacattttctagtatacatctggatatctatgtactatattagtatgcgctcaatcaaagagcaaattatttaagagtactgcctatagagaccgctcctggcttgttatataatttgccaatatgtgtagtaagcatctatttttctgttcccgcgtctggatgataaatcgaatcta

>TaeST2.56749.1

agaactatgaaagcctccgtctttatttttatttggcttttctttctttctttctttctttcttaattcaagaccccttgaccctaatataagatcttttgcagtcaacaccaatgtgttgtattaacatagcatacatgaccccaagattttgcattcaacaccctaaagcattactttccttacaaagaaggccaaaaatccgacaagtcgtgggaatttcgggtataaattccggcagcataacgcgttgggtacaaatttgagaagcgtcgaaacaatggtattatatatcacattgttgcgagtagaaatacaacagtgtgctttgcctcgtcctattccatccatcaatacaatctgagagctaccacctacctactccagtgcatccatcctaccaacatactcaaaacaactaactaatctcgacaaacggcaaccaacggctccatccatcatcatggcctcgggcttcaccaccatgacctccttccatccttcctgagtcacaccgccaacggcatagcgacggaggcaaaca

>TaeST2.56770.1

tacgcacacaaggtaggagagatctaaagctagccggaaaagagaggagcccagcctgccggcggcgagatgtataatcccccgccgccacagggcatgtcctactatgagcacgcatagaagcgccaccaggagaagggctgcctctacgcatgcgtgttcacggcgctgtgctgcttctgctgctacgagacctgcgagtgttgcactgttgcctcgactgcatctgctgctgctgcaactaaaccagccatggtcaccatggacagacggacggagaaaggtgcagcgcgagatcgaccggcctccactactgttaattatcccaaaaacaatttagaatttcgtaataatctagctagcagtttagctcgatgagaactgtaccaggtacttctataatattatctgcagacaagagtatcattgtaatcaccaaccatcatggtttgtgttgcatcttattttaattagcatcatattctggtcctcgcctgcccactgcgctttttcgttgacactttgctttgtctccctgaaattatctcaagactgaaatgtacatttaattttttc

>TaeST2.56773.1

ttcagcagcagcacaaacttgtagtataacggagacagcatggacaaacgaattcaaatgaaacttcaacgaaaacatctataacattcatcagtctacaaccacgctgccataacagatgcgtaagtcgaacatcagcacgtctcggaggagaacactctaatgtcgaggaggagacaaacaagccccaaatccataaaacaaggtgcttcagcagacttcacaggatttcgcacggcatcaatcaggcctcggccatctcttgctcgacgggagctgcctggactgggtagtagtggtagtgcagctctcccacatcatctccagacagcttcttccagccgtcagggccgacatagta

>TaeST2.56779.1

ttcccctttcttccgcagacccagaaaaaaaagggcaaaggaaaacaatcgagcggagaagagggatcaggatgcggctctccttccccctcgtcgccggcgccgtcgtggctggagtgatttctgggaatgtgatattcgggccgccgctccagaagtactgggcagagaagcagcagcaagatcaggcggcaaaagaaggccaaaccgggtcaacttaacggagatatcttcatgtttcgctgagccaatgtcaacatacttccctcgaccagtcgcagacaaacaattctgaaactaataatacaatgatgtgaaatgtgaaatgttatttaatagcctcggtttttgttagctttgcttgaattagctgcgtagctttggtactgaaaggtatcatttgtaccatgtacagaatgtgttcgaatccaaaccaccattttggtcaaactttgtttgaattcagtttaatttctttggttcaaa

>TaeST2.56825.1

agctgttgagagggaggagaagagatgacggatcctgctgcgaagattccgcaagaccaagggcaggagagttgggacgaaaggtgcggatctggaactgtcaacacactagatctgctaatgtgctatcttggtcaaacaacttaggtattaagagttaaatttgggttgctgagacaccgccatggctagttggcgtggggaagttcttctattttgctgagatgcagccatgcctagttgatgctcaaattgtcttgctgtgtagtctgtagtttcttctattatgtatgccgtgggatgtgatgtggttgatgctgagattg

>TaeST2.56825.3

gaaatctaactgtgatggtgtttttgaatgggtgctgctgcagaaaaccattccacttgagggtatggttccaacgagaatggattctgtacttttcgtcgggtatgatgaggacaatgtgattgttctcactactaaccggcaacttcacactccaactcgactcaatgcagattaaacatattgtcaaaagaaacaatatatgtcatgacaccttttatccctacaggaatttctatactccaggcaggagagttgggacgaaaggtgcggatctggaactgtcaacacactagatctgctaatgtgctatcttggtcaaacaacttaggtattaagagttaaatttgggttgctgagacaccgccatggctagttggcgtggggaagttcttctattttgctgagatgcagccatgcctagttgatgctcaaattgtcttgctgtgtagtctgtagtttcttctattatgtatgccgtgggatgtgatgtggttgatgctgagattg

>TaeST2.56933.1

ctccagttgctaaaataatgccacaaattctggagagtgagagggaaagaagggcatatcggcatatcttgcggaaaggaaacacaagtgtgaggcatatttgaaggtaagggatgcaaggattgcagaacgcaaggctgcagagcagaaacctcttagagacatacaaagcatagctcgcagccttttataccctactgcctctaatgcgcaaccaatacaaaatgcacgaggtgccggtgatctcagtgtctcggagcatgaaccagatgttgaagaggaggcccattttgcggagaggctttgagagatgaagaggaagctgctcttactatgtccaaagaaatggtgcccacttctatttacatgtgcctacgaagatggttggcaaagaaaatagtaatcgaaacttgcaaaatattcttttacagagctctagtcctaataacactccaggttcaaggcaaactgttattgcttcaaatgcactcatacaagctgcagcaaggaattcttgaatgtttaaagcaatggatccaaataactagctggactggactctagatcatgaacaatttagtcttcgcagttaattaggatggctaaattacatttgagtttcatttgatggatatttgctatgagaattatgaattttatgaatctacatactatacgtatggttttgaatattgtaattgaatgacagtaatttttttacggttgcaataggctattacccaacacacttttggttgccacatgttagcaccactaaaaatagtgttggctcatatctcaatcgctacgcctacttgttttgttgcactaggatgttgcaacggagcattttatattgtcgcaatagcttggcaccacaatt

>TaeST2.57012.1

attcagaagattaattgagccccaaatgtttcaaaagcaagggccggcaatcaaacattcacaagattcaaagggttgcattttggtcattcctgacatactaaatcatgggtaaaatacagtccttgctggcttgttcattgaactaatgaaagagccactacagaccaacttttacatacatgatccaatcaaccaaaagtctgctcatcttggcatcagctagcctaggcggcggcggtcttcttcttgggggact

>TaeST2.57277.1

gattacttatggggatccaagttaccgattgaatcttctgaaggacctccaattttggacagtaaagcttcacgtcatagataggtgggagagaagttccaaaaagacatgggacctgaccgagccactgcctttacatgatgatagtacatcgattatgaaccttcttggcccaaaacctgatgttcactatatttatgtctgtataaaggacaacaacagtgcaagagtggacttgaaggctgaggtcaatgaagcagacaaatgatcgcatattataacctttttggatcatcgtgtataaccagtgtagcaaataatgtagggaatacattttgtaactcttaactgatgtcgttgttggcaacagtttagcaaaattacagctaggatgttgcttcttcgatcatggaatttgtaatagaaacggatgtgctgtttttcc

>TaeST2.57304.1

tgtaaagacaatgtcaaatatattcacgcaagcataaatgctgaatatgttcactgataccactgacgcccactccagtacagttacaaccaactaaaacacaaagagtgcagctaaaaggttcacatggccaatactaagcgttcactcaacgcaacctagatgctcctctactgaaatgcccattggttctccctgcggatcgcctcctcctcctcgggcgactagtccttcatgatgctgaggaaactacggagctgctccagtctatggcaactaatcatgttgatagtctggcaagtcatcaggtccaacaaccctttgatgttgaggtaacttgcagcctgtgaggggtaag

>TaeST2.57399.1

taaccagaagctgacagtcatcggtttaaacagaaacaaggtacaagatgttaataaccagaagctgacagtctatgaagcaaatcaaacattcacaagatagattgcagccatggatacacaagagaagatggttgaaaggattgcatctttagccattccggacataaattagagatggaatacagctcttgttggcttgttcatcgaacgaatgaaactgccaccgaagactaagcaattacacacaagatccaacaaaccaatactcatcatcctcactgatacggacctaagcgatgcacgatagtagtctatgggggcagctactcctttgcggcgaccttcttcttgggggacttggtggtggtcttcttcttgggcgacttggtggcctccttctcggcggcggcggcggacttcttggggagcagcacggagt

>TaeST2.57398.1

aaacttgttgacataatctcgcaaaaaataaataaaatacttgctgacatgaataaatggcatgaagaaaagtctgaatctgaagcgacaggcaagcacacacgaacattgattgattcaaacagcagcatcactatttctttcaaggccgcaagcagcacaattcttacagagattgtaacaccgtgctactgccaactacatacacgatgcctgccaccatcacctcggcatgattcgaggatcaagcccaagcctaggaggaggtgaacttggtgacggccttggtgccctcggagacggcgtgcttggcgagctcgccggggaggacgaggcggacgga

>TaeST2.57412.1

tcagaagaaggacatagcatagggaagaaaaaaaggacatcatggagatatttaagaagaacacaagtgtccagtgcttgttggctcttgtgctcatggctacggtcatttcgtcttgtgatgcgtttggcccagaaggagggtggtgcttcatgcctttcgtgagatgcgatctggacttgtgcaagcgtagctgcaaaggtctatttccctattgcgaacagatacacggagtttcttcccaatgttgttgctctcccaagaaacttagtgcatctattgataagaatagtcgtccttgagatgaattcctaaatagtaattgatatgtcactgtaactaatctttcatggatccaattctgctcgactgaataaaatttaccaccattgaaaaaaaaacgctcccgagatatttatgttgaaatatattgccca

>TaeST2.57420.1

ctatgcaggctataagaactggttggatccatccgaatatcaatctagacaatccagagaaacatgtggatgtcagcttgctggtgggatcacaaaaggagagatgtgatgtgaaagttgcattgtcgaattcatttggattcggtgggcacaattcatcaattttatttgcgcccttttgagctctattggtgtgctagcttatggtacgattgtgtcatggaactggaattttgacaaagctcagcaagacagcaacatttcacgagccacaaatttgtaatgggaatccaactcagtagaatatattgctacataggtgagcaaagaaagcaattaattttcttttgtattgcaaaagcaaatatatcccatgcaagggtgtccattcagaagttaatcaagacgttgcacatgcactttggtctatcctatttttgtaccagatgttattggattcgtcgtatgggaact

>TaeST2.57629.1

aggttccctggcctgagcctgcccatgctccacggcatcatggacgccgtgtaccagtgatcgggcaaagacggttgacctccggtgcgtgaggcggcgtagatgtcttgaggcatcggccgccatggagaaggaggccagtcaggtgatagttcaagggaaccgcataatggagataccatatatataggaggcatatcaccctccctgcctggaggatccggggacgcctgtgagataaataaagagcgcccgaccatatagtttgatccgttccggcatgtttgaattgaattctgccagtgtttgcccatctttactgtcatctggggaatcatctgctgttgtaagtcgtaaccggttggttgtttgttgtcgcaagtctgtatgtttgtgctttggcatctcagatttgatggtgtacatgggaaatctggttcagcaaaaagttcatgttagtcccttgaattgcggaatcctttactacctctttggtttgtaggaagtatttttttacagtttgttgctgagtgttaggtaattaacaaatattgaggagataacaacaatgtgtcttaatcaatcttatacaaa

>TaeST2.57726.1

tttggaaatagtgtgagtactcatcaagaataatgacataatgtttataattagggaaagtacaatgggaaaatctcaaaaaatgtattttttcgaaagcagcagtagtaaaaaaagggaagaattaatacgcaggtagggccgccgtccaaattggcaagaatcacaaacaagattttgctgatttttttattacatgatggaagtaattttgtagctactatagaaggagcatcttgccctgagtgcctcagacttcaatgccagtggtcaccagaactggttgcagagcaaacaatggtgacacctttattcatcgaacgagagttgtagaggtctgccgagttatttgatctcggggggattctcattgttgctaggtcctt

>TaeST2.57794.1

ttactgaaagagtcatgagcgacgttcttgcagaggaattttagaacatacaggatcaagcaaggaggtacagtcatatcgagagatgcagtacaactttccattggcaagaaccaacaatcccattacattcagatccatcgccttcccctaactacacagactgatggaacagcaacgcacgcactcattcagaaacaccggaacagcagcagctagcagagactggacggagcagcgggcgcaggtcagccgccgaatccgtagagggtgcgtccctggcgcttgagcgcgtagacgacgtccatggcggtgacggtcttgcggcgggcgtgct

>TaeST2.57810.1

tgtttccatcagccagagactatcatttttgtagtactaaataagttgcattttatttttctttaatacaagtaaggaaaacaccgagagaaaagttgaagtcgaagaaatgtagtactgaaaaccctggaagatgttttattcaacatagcatgcatggcaaaaaaactagttataacgcgggtggaggaggacaatgccttattgggtcacgacccgcacacgaggtaccagcacctgacggtgctacagtagagttcttcggagtcagcgcatagcgcacccatccttgactggtttcttcgattggagtagatgccactgcttgctccaggttcatgactgaggggttcatcatcaaaccaagaatcatcactacagccacaatttttgacattgtccttcacaatattgtgaacaaagtatctagatctagcttgatgtaaccg

>TaeST2.57812.1

tgaagatccaaacgaaaattaccacaattattaactctctgctgagaaattttaagtggcataataagttggtggtgaccagattcaaatagaaagaaccagcaatcccattgcattcatatccatcgccttccactgaggaactgaatacagagtccgatacaacaacacacgcgctctgacgaactgactgcattcagatccatcgccttccactaactacctacacagaccgacggaacaacaacgcaagcacgcactcagaaacgaacgaaaccatagcaccggaacagcagcatccagcaaagtcgactcgatggaggttggagcagcgggcgcaggtcagccgccgaagccgtagagggtgcggccctgtcgcttgagcgcgtagaccacgtccatggcggtgacggtctt

>TaeST2.57931.1

tccgatttctacctttagtttatatgtgggctagctactacacaatttaattactctgtatattttgtattaccagaacatggataaattgcatgtaaaattttggtgatgagctcggaagaatatgaggaagcgaggccgccagaggcagaggagaagaggcaagcgagtgctccttgcctgaaagcgaggccgccaaagccagaggagaagaggaagcagcagggtagggcgtggtgatggcgggatctccgacatgctcaccattggggagtggcggcggtcagagttatctaggcgctgcaagtatccatgggaggttcacggttcagatttcggaccattcacaagtcgtgcaggaagcaaacaaaaggacaacactcactaggcacgacgaagccagcgatccgtgtcaattggatgtttttgctttcattttgtacaaatgatctcacatacttacatcttttagctgtcc

>TaeST2.58014.1

tgagaaacaatttgatatatatagataattggaaggccgtctcacgggggcagacaccgctttaaccaagaaacgaaggacatgatcgaattgaaattgcgactaaatgattagtcgatagattgtctgcaagacgaaggaggaaactagataactacgacatgcttgggcagtttcaagcgaccttgcagcagatgaacatcactgaaaactgaataagtaacagaagagcgatgatgcagcagtggaacttgcggtcttcgcgagatgggacagtgaacagccttggccacttggtgcgtatgacattgatgtagttgtcctcccatttgcagaagttgcatttaactaaacagaacaacaaaaaacaagaggaatgaggaaggaaatcatatgatatacaatgaaaacaagggaaacagatttggacatagacactcgtgttcgcaacagtaaaagtgcttccctggtttgattttggtgcgagaaacccaccactgaaccatcccattgttgcaaaacgggcataccatgagggggaggggaggctcctcatcgagtggaagaaggcgggacgaagagcttgacatgggctatgttgagcttgacctggttatcagaaccatctatgtatgaggtgaatg

>TaeST2.58026.1

gatatataataatctcctccatttttattatgtaagtaactagtacaataactttttggaagtagcatcacaagtattcaagattaggtaacacatgaagcacacatatcatttatatgtcgagcttactacttggggaagaataaaaccacatatcaagatacaatggatggtgcgggtgaattggccaatccgctaccgaatccgcaacaatagatgttcctagggcagcacactggattgtaccccaccggttctagcagctgcatgtgctaccgggttgctgccatcatcatgagcaagcagacttgcgccatgagaaaaattatgatcacttggaagatgccattgttcgccattgcctaagaacacagaacaacagaaaatatatagttatgcaatagtctatgaacca

>TaeST2.58038.1

tgcttggaatatgaccatgaaacatttcataactgtattatcataaagcatcgtccaattacaaaaacatataacttgaccacatccagggtccatccatggtatataactagactgaaaagttcaacagggaggagcagaagctactcgtttggagcgagatacgagcgaaaggctaagcatatggaaatagtagaaaaaacaagctggctggcatttggaatgtgcgagcggcactctagtttgatgggcttgctggatcactagaggatccactctgagaatcgcccggttcagtagcacctggagttccactggccacatcctgtttcatctcctctttttcatcagctccaaaccttccactggccacatcctgtttcatctccccttcttcaccagctgcaaaccttcctatgatcacctgcgcatcagcacaacaaaatgcaaaccattaagagaaagcacaaggttctcaattgcgacatatgtgtgttttcgtgaaacgatgttatttcccaaacaagctatgtaacataaaaagaagcacatcatgattttcagtaggagtattaatttgtatacaatgtaactcatttggcagcagacattttgttttcgaaacgtaggaagcacatacagacttaaata

>TaeST2.58127.1

aaagcttgcgcgccgttcaagcaaaggacccgtcgtcgttgcatttgtttatacgatctgacgaggacggcattgaaaatttgagacttgactcgaaacatgttgacgagagttcgcaaaatgcgcccgttaacatggcatggagcaacttcgctccaaggtttcactcaccggtcgccggatcgaccagaagaaaatcaatgtccggtgagctcaagatgccatcaagataaacctctcatgcctggtcttgttgtgctcttgtagtagcacgagcagatgccaagtagtaacaaacagagaagaacatctaaagagaagaagaaaaagcagaggaaaatcgagcacacaactatgtcttccgcggaagaagcactacacgagcacgccgtcacacctcgatcgatcacgccaatggatcaaacaccagggaggaaaataaaaataccctaagccagcgtggaggccacggccacggccgccaacgccgcggcgagccaccggacgcgcggcgcgccggcggccgcgctcttcttggggtccttcttctggtccgctggggcggtgtcgtcggcctcggggccgggcgcgtccgcgacggcggggggcgagcggt

>TaeST2.58137.1

cagattattcaattcaagctttcacaatggcttccaagatagcactagtagtcttgtctctatcggtgttagcattcttcctctgccttggcactgccgaagctgacccacggtgcctgtacaaggataatccttactatcatggtagctgcgagaacccttatgacgagaattgtcgaattgcttgtattagatttgagccaccaaagtacaggggtggtatgtgctctattggaaaatgcgtctgctatacatgttaatgattatggtagtggtactattcaatcgctaggggagccatgaagatacatcaccaccatggaaagattagagctcctaggaacattgctttttgttgttgttgtatcagaataaattggcaatgtatgtgactgtgctttagcaagtatctgt

>TaeST2.58231.1

ttacaaagaaaaaaaaagacttcgttttctgaagacattcatggttacatgatcttgcagttctaatgatgtaaacaaagtacgttaattcatgatttacttgtatcttgcatgcatgtcctaagaaacacatgacaaacaatctttctcattgtatttgttttttcatttcatcttctggaaatgcatacattttcatctcaattttattacataagcaactggcaacaaagttgtcaaagccaaaagacttccggcaacaaacagagtgccgtat

>TaeST2.58252.1

agaagtttgtggtgaaggtggtttcccttgctattgcacgtgatggtgctagtggaggggttgttcgcaccgttactataaacgaggagggcgttaagaggagcttccaccccggcgacaagctgccgctgtggcacgaggagataaagccccagaactcgctccttgacattctcgcggccggtacctctgatgctatggtccagtgagccgtgcttcctactgtgcccgctgatgacttgtgctgaatttgagcttaatgttgttaagaaatcgtgccgaattgttccacaccagcttattttcctccttgttattacgaattaataccagtacattccttgttcca

>TaeST2.58265.1

taaccactcaaatatttatatttatatttatatttatattcatacacctcaaactatacagctgtacaagtgtattacagatgtttggagaaaaatatagggagaccatactatgctaatctatacagcaatgccacaccaagcataaaccaacacaaaaaatatatgtgggtcaaccaggacagcaagacacatttgaccactaccatcaagtgcattacatatatatatagtcaattgaccaacagttggtctaggcatgggttgcaacttctagccaatagaatgact

>TaeST2.58323.1

ttcaccagcagatcaacaacactacatcaaggtatatacaaggacatatgacacaaaattttagtaccggaacacatggttcaacatcacatcacacgagtttcagcagcttcccagaaaagaccacaagttcaaaggaccacaagttcacgcaaagtaatgaaaatagcaacatcactaaaagaccacaagttcatagtaacagtgtgtacatagcatcaatgtcaaaattgagcgaccaaatcgttcgagcagcctcagcgggctagccgttgctgggttgccgctgcctggagctccggccaacgaccctctctctctctgcagatgagatgttgccactgaactaagacgccaccgcaggcgaccaagtcgcgtacatatgctctgtagatgagaagttgcgaccaaaccaagaagccgccaccggcgaccaaggcgcatacatatgcatcggtgttacgcagtggtgcaccaacagtcgtccgctacatcg

>TaeST2.58350.1

caaggtcaatcgcatacggtgccaaacggggttcatctcatacaactcaaacattcattcacgggtcatcatctcggcggttataccttcgaagtgtgcatctcggggtggttcgggtttgtcgagggaagtagtcgttcacgggtcgtagtggaagtagttgtacgcgacgatagtggaggtagtggtacacggcgactgcaaaggtactctcgatattggcgacggtaatggtacacgttgttttgttaaatccacaacgacggtagttgtacactcgacggccttcggcgatggtagtcgtacactgtcttggacgaacttgatggatccgagggctccgggcgtcgtggtacttggggtcttcagtcttgccgatctcggttcttccgatggtagtggtacacggtcgtcggtgttggcgggtcgtcgtggtacttggcgtccaagtgggctcccggggattcggcgtatccgcgtagtcatacaagggtcgaccagagggccttggagatccaagatgattcaaggcgagatgcacgactgttgtagttttcggacttgggtcttcatgtgcacgtcttgacgagtctgggggtctcggtggtgaccatggcaggggtttggcgatgcagatttgcggaggtgggaggccggcaacggacggccacagcgaggtgcaggagaggtggccatgacaggttgtcgaagagaagttagatggtgtcgggcgcgcagctgcagctcgagcgcaagcgctcgcgcgtgggggatgcagccacatgggagaaggtggcggacagagcaggtccgtcgaggaagctgagggacggcggttgcaggggcggcggtgcaaacagggaagggcggcgaggatggcactcggggcggcgtgatgcataggacgcgggggaggctgcttggcgcgagaattcgcgaggtggaggaggcgcagggtaggcggctggctcggagctgagggtcgcgcggaggatgtggcggagcagaggagaaggtgaggaagggagactgggaagggtggaggaagcagtcaaggggaaagacgaggggatcggttgggggtatgttgcgtgcgagtg

>TaeST2.58373.1

taacaaagctgaccaacttcagatttactcaaatggcgcctaaatatctaaacaacttgtagaggttaatgaaattgagatgttgcacctgcagacttacgaactacaataccagattcagactgcacaaaatgagaactagcaagacgtacagaagaaccttccaaatgaaactgaataatacaagcacacattgaacaacaattcagacagcaacattactatttcttcaaaccaacaagcagcagaattcttacagagattgtaacatcgtaccactactgttggtgggcttcttgttgttagaacaccaccatcatcaattcaagaatcaaggaagcctgcctaggaggatgtaaacttggtgacggccttggtgccctcggagacggcgtgcttggcgagctcgccggggaggacgaggcgcacggaggtctggatctcccgggaggt

>TaeST2.58382.1

gggacacagcatatcttctaattccatcactgcaacaacttggtacatgtgacaagaccaccattgcagaatcacggagcaatttacaagcaaaattaaattcattgaagcatcaagacgataactgaactttccattgccattgacatatcattcgagcatcacgatacatgagaccagacgacacatcatatcaacaccgacaaacagaagcaccggacacacacccggaccactggacaagaacagctactacccagaattgccggcgaccacgacgaacctaggcgcgctccccgcggatgcggcgggcgagctggatgtccttgggcatgatggtgacgcgcttggcgtggatggcgca

>TaeST2.58394.1

ttattgattcacatgttgttgggtcagatataatttagggcctcatagactggcggttccttgaatgacaacatacatgaatcaggaatgagtatatccttgaataaatataataaccaccataacataaccatgactttgaaacatcaccacttatttggtattatcaaaaagtagagcaatctctgcaactaatgtagctaactctactctcgtaaactcacggactacatacacatagcgtaaatcaaaacttacaatcatcaattacatggaagaaatcttacacggtgcggactggagttcatctctaagccgtgccgttgctggactttggaggtaagaaggcctcccaccatccagactcgtatttggggtatggagccgtccatttctcaggcttatcaaatttcaactctgcaggattagaagacaatgcttcttcaagtgtctctgcatcgaattgctccgctaagacccggtccagccttagtttcatgaatctga

>TaeST2.58522.1

cctgcacatatgcacgatatgctccatggccgcacacttgagcattaacctgcaccacccagcggctccgcggccaccagcactggcgacttcaatctggagcagcagaggaagagcagccccaacaagaacataacctgcaagtcaaatctgagaggggattagaaagtttggtgctgaatccttgggaaacaaatttggggaattggatcaggggagagagagagagcggaagatccgacagcggcggagcagcatcttcccgttgctggcggcggaatcgatctgggcaaggagtagaggaaacagagaagggcatggcgtgtactactggtgttctggttggactagcactagtgtgttttggttcggttcagaattgatatactagtactaggagaga

>TaeST2.58527.1

tttcaccaccaaatgagttcatcatatgccaaacttaaggagatgaacaaggtttcatgaccaatttcagcaccaaatgagttcaccacatgccaaactggagcaaatcaacagaatttcatgaccaatttcaaccccgaagcaagttcatcatatgccgaaccgaactagaggaaattaacagagtccgcgaccaacttcaccaccagaccagtacctactttactggatcgaagacgatcaacgcagcaaaacaaggaagatagacaccaaacatgaccacatcaacaaggctctcgcgccggccatcctccatgagcagagcaccacctactcgacgttgacgtggaagacgtccttgcgctc

>TaeST2.58539.1

attagtgctggagatccttgtggtgttggtggcactggccctggcctcggcgggcgtcagcgttggcaacaccttgccatgcgcggccgtcgggtgaaaaaaattaatcttgaggaggtccaagcgccggatcgaatacatgtgcctgaggccgtcatacaacccaaggttgaggatccgccgcatgccgcccttcatcctctcctctcctgggagggcagtagatctacagaaaatcgaagcggcggcggcgaacctggagcagatcaagcggcggcggcgctgaaccctggagctgagcgg

>TaeST2.58554.1

gacctcaaggcaggcacccattcgtcactcaaaggggaggcaccaacatttcataagatgataaaaagaaccagcaacacagaacataatcaactccccagccaccaactaaacaactaactaggtatagtgtttacgcacagcctatgggcaatccatgacacaaagatatatagagttgacgggcacataagtcgatagagaagacaacaagtccattcacacggtgttcgtccaaagcacaccacaacacagccacttcagagatacatacgcaccatcatgccaggaatctcacgaggttgtgctcttttccctttgattacagctacataataaacaaggatgcgatctgctgctccagctgcat

>TaeST2.58555.1

actcattaaaccagagcaataaaagtcactatgtaaacaatgagtcctcccatgcaaaactaaaacagctcactgtataaatgaaaatagaaatccactacacaaaagtcacaaatgacaaccattcactatataaagaaacacgagtccactctcccaaacaacaatagttcacaagttttctaaacaagcacataagcatattacggaaaccacatcggttcactatgtaaacaaacaaaattccacccacccaaactatatcgattctacgcgcgacaatgtaatatagttcactatgacatcaatgctgaacatgatccaaggatagatgcttcagttctgcaagggaggatgtgcagaatcgtttacatatgtcttggccttccttggtgagcgtctttgatccaatgtagcttattaattactgatgagatgcaagagcttcattgaacatacgagtgatcgtccatgttggagagctggttgtgtgccgagtccat

>TaeST2.58586.1

aggaatactgggaaactggaagacacaacccatattcaaccaactactactaacgttctacgaacaaggatgaggggtagccacatgatcgtcgtttgtcttgttcttctggtgttagcctcaggtacggcgacggcatcaagaatgggcggatatctaactggaacaaattgcttagtcaaaacccctatgccttgtggtgaaatcaagagatgcatcgatacctgcaactccttgtctccggtgcacggcaaacctgaaattagttgcgttgacaaagagtgtaaatgcacattttgttgaagttcgacatgaatctacaagcaaggcatatattttcttattatcggagtagcagtaatagtagtattcattgagtgtgtactggcaatatttgataccaatggtgttctcaaaacaataaatggcatgaaaaaaaattcaattgcatatgaaacatcatgtataacca

>TaeST2.58587.1

tgacatagagaacacacatcaacttttgcacgttcctattgaaagcatggcatagaccacaaatcaacttttaacgttcctatcgaggcagatgaggatacataccctatgccttattaggagaattaatctcaagccctgacaatcacagcaacaggtccaaaaatgtcaatccctcaaggatagtaactacatagtgatttcacactgcaatgtagactgtacataccatccaggctgctaacctaatggcataactatacatac

>TaeST2.58600.1

atgcaaaattaaagtagttttctccttagacctggccatggcagtcatcaagagcagtcgcacacatgccatgggttgttcaatattgattgttgttatgatcatgtcttccactttgatcttaccaacctattgctatgaacttgatcttcatgaagagtgcttgcctattcaagtctgcaatacaaatgtctgcaaaaacttatgcagacacaacagcaacagttggctcggggcctactgcaggactggaccaggcgatacgaaaatatgttgttgcgcttattcctgaatctctagtgatgcttgttgttgtttggggcactaatattgtcgttccctgtatccacccgcttggaatacattctcaataaaataatattctagcgactcaatcaaaattttaattattatgaccaacatcatatgtgtatgatcgatatcgtgtgcacatatctagttagtggctcgtcttgacgtcttaaaccagcgttgaaaagattattgtatcgact

>TaeST2.58604.1

aaaaagatatataataatctcctccagttttattatgtacgtaacttggacaataactttttgaaagtaccatcataaatattcaggataaggtaacacatgaaccacatatataatttatatgtcgagcttactacttgggggagaataaaaccacacatcaagatacaatggatggtgctggtgaattggccaatccgctaccaaatccgcaacaatagatgttcctagggcagcacactggattgtaccccaccggtctagcagctgcatgtgctaccggtgctgccatcatcatgagcaagca

>TaeST2.58617.1

gccgcgcccacgcgcgccggcgccgcggccaccagcgccgccgctaccatcagcgcaagaacccgcatgacggacatggccgctgggcagcgagtacgggggatctcttctccggtgccgaggagttaatgggggttcaggtgggaggagaccggtgggtggtgcggtgtggggaaagaggagggctgcgatatctttcgggttgagagacgggagtccggag

>TaeST2.58662.1

actgtccaattccatccctgcaacaccatgctacatgtgacaaaacaccattgcggaacaacagagcaatgaagacaaaattagcatcagatgataactgaactttccattgcaatttcattcgagcatcacgatccatacgaacagacgacacatcatatcaacaccaacaaacaggagcacccgacaggccgacacacatccagatcacaagacaagaacagctaccacccagtatcgccggcgacccccaaacctaggcgcgctccccgcggatgcggcgggcgagctggatgtccttgggcatgatggtgacgcgcttggcgtggatggcgc

>TaeST2.58692.1

tgagccgaaaactatcatttttgtagtagctgaataagttgcactttatttttatttaatacaagtaaggaaaccaccgagagaaaagttgaagtcgaagaaatgtagtattgaaaaccctggaatatgttttattcagcatagcatgcatggaaaaaaactagtcataacgcgggcggaggaggacaatgccttattgggtcacgacccgcacacgaggtaccagcacctgacggttctatagtagagttctttggagtaggcgcataacgcacccatccttgactggtttcttcgattggagtagctgccactgcttgctcctggttcatgactgaggggatcatcatcaaactaagaatcatcactactgccacaacttttgacattgtccttcacaatattgtgaacaaactatctagatctagcttgatgtaacc

>TaeST2.58780.1

actagttgcaaactctgcatcaaatgtctgacgcacaagaatcgtctgaaagcacagccaggaaaactccagcccaaacttctgccccaagtcgatatcattcaccgaatttaaacatccaatcaaattaaaatactatgggcaaatcaacatgagctcagaattaaaatgaattacatcacccccattgcaattttcgagcgggtaatcaacatcacgagactgacaggacggaacaaatcctaataacaactcgaagcacgactagactagacctaagagggcagaacaaattcatcggcgggatcaagcaaggaaccatggcggcgggcgggacgcagagcaagaggcctacgcgcgctccccgcggatgcggcgtgcgagctggatgtccttgggcatgatggtgacgcgcttggcgtggatggcgcagaggttgg

>TaeST2.58810.1

atttggatcacaataaaactttgatgtttactttttattagaaaaaaattagccccaatgtaaaataaattttacaaacaactattgtgccatatatacttaggcatgaagaaaatgccaactaatagacagccaatcattttacacttgtcactagtacaacttgcaagaatgacaaatggagacttgcgtgcgcatggaaccatgcatgcatgccacctcctcctgctccccaaaccgacctagctagtacccagctataaataggaggccatccctccactgtgtatttcacacaccacacacacacatcactccctggagcagctcactttctcccatcaacttttcacatatccattgttcttctttggttgcatgctgagttgccatcaggtactattcaagtaccttattttttttgcagctttgtttgcttgatgattgatgtgtcgtcacctttgcttagattagaatatgcatctaataatctggactctaggaccctg

>TaeST2.58813.1

tttaatactgaacataatttaatactagcttgtgactcaaattgtatagatgtgcacttgtgacaagatactttaatcacattattttattcaaatatgaaaattcaacacatttcctagcaatgcacaaccttattttccaggaggaaaatgtgggcatttactctggcaatccaatctggaataaaaacaaggtgctcccggtgcattgaggcaacaaaagcagtgatttggacccggttcacctttggtctcgcaatcccgtgcgacacaccattgcaaggtaagttt

>TaeST2.58814.1

agaaacattaaccacacgtttaaatagaaatgacaaagcaccaaaccctaccgcaaccctccgcaaacttggtactattaaataccagtcattctctaccatcagcatcagcaaaacacattcactaaagctagcaagtcatttgcagagacaagctttacagataaaacagcgtacataacataacagaatctacattcagcattggcaatctccaccacaggaagaagttcctggcgcctcccgtgcttaaacattttgccacgcatagcagagttaataaaaggaataagaaatccacacacaaaattcccagtcatgtttcctgctccgatctttccagaatggcaggacagatctgcatcagaccaagaaaaaacaacagcatgcaggttgacattttcaacacgttgttcggcgcaccttcataacaagttttggggggggtgtgctccatcacaataactcatttcgtaccggaggggtgtgcctaaataatattaatctttatcttcctaggcggtgttccaggttttgagttgccggttccagctttccctttcttatcagaaggtttaaggatctggaatgagcatgttagtgaatcatctatgctcatcatggcaccagcattgtcgaactcgccacaatagttgggtgcagagaatatggttacgagctgacgctttgcaaa

>TaeST2.58950.1

tcctcccccggacgagccaggtgccgcatttatatagccgcgtattcactcacaccgcctccccctccccctccccacccatccaattccctcactccaccgcccgctccttctcggcccttcctcgcattgcagggaggaggagactgaatcaagatgccgaccatcatcatcaaggtggacctggattgcgcccgctgccaccgcaagatcgagagggtgctcgacaggatcagagagaagggcgagttcgtcatcgacgacatcgagta

>TaeST2.58951.1

cccgatctttacatacacagaataataatactctacgagaaagcatggctgcataatcactctccctcctggggacagacggagccatcggagcaaagtaaagctgctcagcataatgaagcccagcacaccatcagatcagagcgcaacatacgtacatgaatacataatttaaagtgagaagatgtcacagcagcaccgaatactaataatttgataaaagtggggcgctgacaatgacactgatgatcgatctcagatcctccttagtttctactactagtagtagtatacgtactaccacatgaatgcatttgggtgtgtattttattctggcttctaatcacaggggaaataataaacagtgggggtatagggagtgcgtgtatatgtacgcagaagagttgatcgtatctgcacggtgcttatacgggacggccggattaatggcggcaacgtagcaacgcgacggcggtggtggtgggaaggaacggagtgtgctacgacgtactggctgatcaatcgatcgatcacatgatggcgcaggcgtaggaggggtcctcctcgcacacgatcttgtagccgccgcacgggtacgccggctgctgcgggtacggcgggtactgcgggtagatgttgtactgtggcggcgggggcgcgggcgcgggcgccggcgccggcgccggctcgtccttgcaggagcagccgccgtggccgtggccgtggcagcagcagcagtccgacggccacgccgggtacgggtaggggtacggccacgggtacggcacctccacgatcttgggcggcgggggcgcgggctcctccttcttgggcggcggcgcgggttcctccttgggcggctccggttcacacttgggcggctccggctcgggctcagggcatttgggcacctcgacgaccgctggcggcggcggcaccacgacctccggctccggcggcgggggcgcgggctcctccttcttcggcggctccggcggcgggggcggcggcggctccacgatctcgatctccttgatgatcttgcaggccttgcagcagagcttgtcggagagcctgtccgcgtcgaagggccccttgacgatcaccttgttgttcttctcatcgtactcgatgtcgtcgatgacgaactcgcccttctctg

>TaeST2.59217.1

cccgtctctggtcctagcaaacgaaacccaaaactatcaatccatcctcgaggtttataaacaaactgtcacataccatggacgccaggcatgccaccctgtgtttcctcctatttctcgtgttgcacggaaatcctactgcagtggccgaggattgcagatacaggaacgataagctgccgctatgcgaggacgttttgtgcaagacgcagtgctggctggagggggcggtggtgaacgccgaagtaaaagagtcgaggtgcgtgggatcggggccagactccacctgctactgcctcttctgcaaaaaagattgatgaaccttctcaaggttcctgccgagcgtgagggcatcgctagagacgaaatcctaccgagttgaattgaagacgccaaagcatggatgctgttaaaaattcttgtagtagccagtagtgttgtccttttctggaaaaccagtgaatcctttttttcttctatttttgtggggaagggaatttttttccatcaaacagtgaatcaaaaaaaggccgcccccttggatatctatatccttctcttgcatac

>TaeST2.59252.1

tgcaaagcattccccccctccactgtttcttgaaaaagttatggtgcatgtaggtccacaagcattgctatatattccatgacacctttggcagaaaccaaaaataaataaaatgagaccatccaggactagcccttgcaggcatattttcatagaaaaaactctaagcaccacgggccactccgtgatttgaacccaggtgggctagataccttcgtgctatcctagtcgacaggggtgacaacctctagtaaaaaccaaacagcgcctacgaccctgatttgctcacttgtgcacccgagcggtagcgactttttggcctaattaagaaactcgaagtattccaacttggtcggttttcacagatcttttttttcttgcaaggtttctcaggcgattttgattgtttacaaagcataacgcaacagagcagaaattcccctaaaaaaacacagcacaaattttatcgcggttgagggtcccgcacgcacggaagctatgccaccggcgacacagcggttggcggagatcttccattgtagcagcctcccagatcgcagtcgtacagaaaaataacggtgcaaaaatgtcttacatcgtgggacggagggagcttgaccggcacccaactcagacgatttgatcaaagttgcaacacaaatttcatcggctgcagccaggaagcatcctggccgccgtagatcatcacccaa

>TaeST2.59329.1

agaggtcgccagatacacccgccccgctccaacccacttctcctcacgcacgccacctcctttctcctccccctctctcagacccactcgcggcggagactggaggtagaacatcgcacgcgccggccgacgaggaggagctgcgctgccaccggcgcaccgactgcatctactacctcgcctccccgttgacctacaagaaggtagccaacggcttgatcatggtgctcggcaatctggaagtgaacctgagaaggcttctcgaggaggttcctcggtagcagaatcaggggatgcttgatattggggactaataagacatgtttcagctatgagccgtgtaactaaatgtctcttgtatgtgtcctaaaatgaaagatcttgaattatctctttctgaaatcatctgctcatctttactctcctattatttgctatttaatgatttgtcccactacttcagcgatgagcatggattgtatgcactagatcttggtgtaattcttgacagagcttcaggtcattgcctatgagttggagaaccaccaatggtttggtatcctgtatttgccaaaagtgaacaaattgagtatgtcttctggaaagcatttgccaaagaaattgaaaggcaacactcgtatcagctgtcatttatcgtttctttcgataaa

>TaeST2.59397.1

aaacaactgcatgaatgcagtgacaatctcactaacatcaattacacggaagctggataaataagcatataaatgatgtctgatcatactacagaagaaaggtggaatactactgcatcaaagacgggcacaattcagggtactgcatcaaatacgtactaactaggattgccgagttgtcgcagctagcatactaattatttatcagtcactcatcagtacctcacgagctgatccatggcgtgcttctcattcctactcctcacctccctggaggagggcccagaagctgat

>TaeST2.59518.1

gcccttaaatggccgtttccgcaaaaacgccatcccccgtaacacatcgaagagtgggtctctgtctctctctcgcggcggcgcaggcgtctctctccatctcctccggcggcggcaaggcattttggcatctgagcccaacagctagcagatggcaggcggcagagttgcacatgcgaccctcaaggggccgagcgtggtgaaggagatcttcattggactcacccttgggctggttgctggtggtatgtggaagatgcatcactggaacgagcagaggaagacccgatccttctacgacatgctcgagaaggggcagatcagcgtcgttgtcgaggagtagttctgacgtgctgaaccacaagttctacatctcgaagtcttttgtcattttggttttgcaagtaatttctcaaactccctggagagcatgacagagtgattgctgtggagattgtcatgaaaataagcaccaccatttgagaacatcgaactgttttgaatttgccttttgtcgtatcaaatatatctttgtcgcatatctgttgcaaacaaattgagggattggaattggatggcttccctcttgacatgaatatgatttagatttcactg

>TaeST2.59618.1

attgtgacagatggttcataattgtttgtccttaatgtccaaatagatgttggtttatcaaggttccatcaagtagacacatcactaggatcaatttatttctcctatcaccaattcattcatcaagagctagcacaccatggaaacttagtacaaagtacaaatacaaagacagacatgtgcgccagatctattaagcagt

>TaeST2.59619.1

gagatacaagcccccaagattgtaatacaagatcagataaaaaacaactttgtaatacaagatgagggctacacatgttctactcctagtccttgccctcttcatgctatccccagatctggcgaaggctaagctgaactgcacggatttcgtcatagccaaccagtgcgacgtggacgagatcacatgccaacattactgctacaaccagttcaatggaaccggcaagtgcatccccaacaaaggatgccagtgtcatttctgctatgcgactccaccgcctgctagcagctcctagtcatagttttctctttaattttttgtttcttcaaggtcaatgtacaacagttatgaacagttatcaacaactatggcataattaattacatgtactgtggcctatactgcgaagctatatgttaatggatactccttgttttgctttatatataaatgtttgttatagtcaaggagcgagaatacatgccataataatcattgtttgttataaatgttttggtttgtgtaaatgtttgttat

>TaeST2.59811.1

gtaattaacaaaacgcaaactgagggatacagtaacggtaattatcatcatcaatctgtaaatacttctctgctaaaatatcgacatcatcggagcgaagcaacaaaggggattaattcagcaggaaatcaatgtaaatctaccggccccggaatctctccttcgcagacggcatgacgctgtgcagtgcacgggatcgatccccacggcgcggcgggacgccgggggcggaggggatcggccccatgccgtgctgctggcggagtagatagatcccgggggagggtctggggtctagtagtaggagggcggcggcgcgaggttgaggtggccgtaggcgcggtaggaggcgccgtcctggaggtggatgctggaggagagcttgtggaagccgcgggtgtagacgccgaagccgaagacgaggccgacggcgacgaggacgaggatgatgagcatgcagatgcagcacgccttgctcgggcagcagcacatcttgattgatcttggcctggctgccgcctccggcttctgctcttggctccgaggtaggggagggcaaggaggaagaaaaggtagcggaaaaaggcgaggcgcttttgctggggtccgtgggggtcggggtggggtgg

>TaeST2.59857.1

agacagtgcttctgaaaaaaagactacagacagtggagttgtgaaggcggcgggatgatctaccggagctggtcgctgctctcgtcgacggtggcgatctggggcggtgccgccgccgcagggctcgccgtcgtctccctctctggcggcaaggaaaagtttcaggactacctgtgccgcgaaggcgataggctgaggcgcgaggacagggccaccatggccagcgccaactaatggaccatgaacgaacgcgccacggagacgaagaaacgagcatttcatcatatatccgcatccgtgacggggtcgtcttggtccaataatcctcatttgccgtgctttaaacatgatgcggtctggtgtcctcattgaatccaataatcgttgctggacatgatgtggtacttggtacggcctgatgagcttcgctttccggcttgccaagtggctcgaaccgtcatcttagcaacaatttcgtccttgtcggcggacaatctttgtcccattttggatttgatcactgtttcggatatgtgaataatggacaccgtgggttctagttgaaatccaattggctatttggatactgttggatatgtttttttttttgcgagtaggatatgtttttctctctagagagcttcagtattattatttttttaaacttgaactcttggcataattacacaca

>TaeST2.60061.1

catactatagctttattcttccttaggtagaaccactcatcagcacgacatatatactcgctggctacatcagagattacgaactctggaaccgtacgtatcatgccacaacatatgagcaacacgattacattcgcggacataggacacaggatacttggcctcccaaaagatagtaccgtccaccctccaccgtgctgtgagagatcataagcattgcaacacaggccatccttcaaatttgtagcatcagtttggaagattacccgcctcattccggcatcgcgggcacactgaattatctgcaa

>TaeST2.60071.1

caactgagcaaagttgctatgcataactgagcagcagcatagtatgaaagcaatatactaaaccttcggttacaaagacagatgaatcgatgagatctactaaaccttcaactatagatacagatgaatcgatcgaatgcactaaaccttcagttacatatacggatgaatccactaaaccttcaattactactgatcaagtaaacatgcgacgaggatactttggccgccaagtcaggcttcatataacaaattttcagcccgcaatactgcaacactgaactttgtagcttcatagaacatgtccttggacaaccttttcttcttttgtcatga

>TaeST2.60101.1

ttgagttttcttgttgggttttacccaaaaagggttgcccgagctttatattataaagcaaacatcactacatacatccgagcgaaacgatacaaacacatccatcacggcacatagcacacacccaaggcaaaatacaaaggtgaggggcaccgacacaccactcggacgacaaccacgcaaactagagatgaaccaagaactacttggagccaccgaggaggtgtgagacggacaatgacggagcaaggactctaggacggtgcctcccagaagggtacggccacggatagccgtcaccgtccgatccgaagatcaagttttcacccgaagccacatggaggagtgggagcaccgcaacggagccttcaagaaggatacgatgtccgtacgaggacttggcaagtgatggacctcggcgttcacacccctccgacagccccgctccgcccaccacccgcagccaagcccccacgaagccatg

>TaeST2.60133.1

aaacttacggttgtagatacgatacctgcacaatctccgcgtaattactccctcactgaacactcgcctgtatcccgtctccccttctcacctcgcctctcaggagtcaggaacctcccatcgtcgccgccaactgcaccacctcggtctccccgatctcggcgttcgcctgttgcctccttctgacctcgccgtcaagctgcagatcgacctcatcgggctctctagatccgttcccctactgcctgctgcctcccagatctgttcatctgctcctcccgaccccatggtgctctccaattatgcgatctcgagaaacatcgcatgcagatgtgccgtcccggcctcgattcggtgcttccccatgtctacacctcggagtcaggccaacgccaggagaacctgcagcggcttccaacattctgtaaatttctcacaaaattgtagctgtgcttcatcgttttgggcacaagtgcatgataaggatattgtaatgatgtctagagcactaagactgggatgaggcatgactctacgtccaacggattccttattggcgaaaagctggcagtatatgtggagctcttccgtgacattgctaagatgctgctagatgagagtgaaaataatgtcttatgctttggtcttgttttttgttccaaaaataagtccataacaatttgttctttgcgggcaactccataatgtctttgtagcatatgtaattggggaagagaaactatttttctcttttgatgcaaataaattgctatttggttgaaggcaattatctggtcgatggaaacaagacatcattgcttctgaatta

>TaeST2.60136.1

catacactcatcaaccccacttacaagagtaccatccattaataacctccggtcctcggtgatgatgaccaagcacatgaggaacaatgtgcaaggacttctgatactttcttttcttcttgtggtgtgtttgacttgtcctagatgcacatatggtgaaaccataaagacgagcaacatgatggccaaagtaagccatgtaaccagacctccttgttataaagatgagcaagtccttggacaattctgctgtaaaatagataagttgtgttggccaaatctcggagaatgcttcatcaactgtccctgcaagattaattgcaaatgagcagaagcaacaacacagtagttttgttatctctcctttttgggtggataaatatcaaccttacggttcttgaaataatgatgtgttgatgagatcacaaatttgctatgattttgaaatcatgtgtgttattttaatatcaagaaaacttgtcggttgtctgcgtaa

>TaeST2.60445.1

gccgccgcgcgaagaaaaaaaccctaactaaccagatagaactcgcgggaggagatccgaagagcggacatggcggcggcgaaggcggcgttggagaagatgagggcgttctgggactcgcagtgcaacaacgaggagaactgggcggtcaactataaggtgctaaaggctgctggtatatttgctggatctgtcttcctgatgcacaactttggccataatatggtcatctaaacaggactagatagggcaacaatggacattcaatggcgcggtgttgcgttgcctttttttattttctcatcaacatggtcatcacacatgttcttagtgtggattaaggagtgaaaccgttgttcttagacattggttggtaataatggcagccaaagtagtatgtgtattactctatcaggtgttctcatgctttgtgaattatcaggcaagtcgtcgcaaacttgttggggatccctttttgttaggtcttcttgaactttgtcctccggttcatgcagtgaattaagccggtggtactatattgctgcgaatatttacatcttcctga

>TaeST2.60499.3

accaccaccaccaccgcctccacccgctcctccgcctgaaccctagccatggcgtggcgcggcgctgcgtctcgcaccgtccttgccgcggtccgccgcccggcaccctccgccgcgctcggcggcctccgcgcccctccccccttcgcggccccgcgacgccggatcccttccccctccacctcccctctcggggccgcacggcccctggcggccatgatggggtcgccgctgacggcggcggtggtgctagggcggatgacggcgcacccgtcggccagcgcccgggcctgctgcgagctctctcaggggaatggaaaagatgggtgatgcgggagaggcaagggtagagcatagctaaggtacttgaatttgctatgttgattcaccatcctttttaaagaggatacaaatcatggattcgtcttttggaaagctattctggggccgtccttttaattcttgagttattagaagaatgtctccaacaaaatagcttctctccaaattactagcctagactgaaaataatcttcgtgtcatatgacatggtttatgtcatggatttagcctcctacttttgtagcaaaatatctttgtgttgcctgctaagaggatttggtggcacattttttatatgatttcttagattggaaggtatctctgttccacgagcaacaactgtggtcacactgtcatttcagtttccagtttagcaatgatgcttgagatgtgtgtgtctggtttccaat

>TaeST2.60642.1

catttgacagattttttgaacaggacatacaaaatagattttccacatttaaacagtcttatgatatagtatgattagaagctagctagtctgctagtcggtggtaagaagcaagctgattgatccctcggcggccatcttattttccttgatgcttgtcttggtacaaagaaatcaaggaatctccaaccgttttgccaataaaaaggcaccccgcaactttcaagcagtcatgaatgaatgcccatcgccgatacgagcccctacaggtgagataatagatacattgtccgcggggaaagatggctgagcagctggacatttttcaggcctgagctgccatggagagtgtaccttgcttcccacccagcaatcttatcgtagagcttctctttcttgacttgaatctcagccaactcggcgacaacaacctttttctcatcgcccgtgaggcg

>TaeST2.60649.1

ctgccaacaatcgttgcagccgttctcatctatctatacatggagaagtgcacgatgcgcgtccaggcaatgctgttccttctcgtatgctttgctatctattcacaatgcagaggaatagaggaccatccactcccccacaggtgcatcggggctggacgatgctatagctgtacaaattccattgatatctgctatcagactttggaaatttgcaaagcacactgcaaacaagccgcagccgaaatcacctcctctgcagttccccattagcttgaagaatgtacttggtttgggtgtgcgttttctgttgacaaaagaactatgaatgaataagagtactatgttttta

>TaeST2.60762.1

atcgtcccatccaaccccacagcaccaccggcgtagcaggaaggcatccatccatggtcgtctccaccttcagtggccctgggattgggctcggcttcggcgtcggctgcggattcggagtcggatgggggttcggaggaatgcctctgaacgtattcggcttgggcatcggtgggggatgcggagttggtcttgggttaggatggggctttggaaatgcttatggttgtcaatatcgatcttcaagggtccagtttcagggcattgagtttcagaaaaagtctgaaggagatgaagcaccaaaggttgcttcgtcagggcttgttgaaaagtctcgtccttatggctagtatagctgcgatcatacaattggcacaggcaatgttatcattgtccccttgattgtagtcaattgtctacctggaaagttgtttgaaagcattttgatgttttgatgaacttggatgtttccatagtaacagaatgaggataatatggtattgttttcgtttcggataatgctcttcatatttggctaatattgtccattaacattctcttggttgggtgcaccctgcaagtgtttgttgttactcttgttaagtacttgcaa

>TaeST2.60937.1

atcacaatatgttattcttgattgctcatcgttccaacataaactatgtgtacggacggaaataaaaggtgcgtgcgcggtggcaaagatgtttctcattaattacccgaatacatagtcgacagattgtataatattatgtgcatgtgcatatacatagccatcgatagcattcacatacgagcatgctttctggagccatgttggtacatgaacatggatatacacatattcttaggaacaaacaagaaagataaatggagaaggagaatccaaagcaatgtcagagttgacaaaactgcaatggcactttatatgtggcccgtggcgagtgagctgtttaggccttgccggactcctcgca

>TaeST2.61058.1

atggcttaaataatagcgtcatcatttcgatgagcactcataatacacattcaaggattcacaagagacgacacaaccaaaaccaacatgacaattcttaataacacaatatttgttcgcaaaataacaaataatccatcatctcatagagaaacaaaataaggtccctcatgctagaacataagagtattattaagacggaggatgaacaatcaccaatgctcaaggaaaaatactaatccatgttcatccgtcttcttgccacttggaaatctaagactttgattcatccttttgagtgtcctcacggaatggaatccctg

>TaeST2.61079.1

cttgttgcacttcaattggatacacgtcagtccggtcactcgcaaattttactttatccaaaggaaacagaaacagaacaaggaaattactggacatatactctactaatgcatgatggtttattattacagaacaacgacatttttaccggcataccagtaggccagcattatgcattatcaacacatagtcggatcatgcaccacgcgcttcagatcatgatttacacgcatataatatatggtagtgatccgcgacatagcacaacatgatagggatgcatgaggttcaggcatatcgtgtgtagacgccatgggcggcgttggatgcatggagggtggtgggtcgatcatgggcacttgggcctcgtgcgcgcgccgtggccggtggtcttgtcgcggtagca

>TaeST2.61093.1

ctcgatgaaccgctgcagctcgcccgagtcgtccatggctggtggctccgaggggggcgggcgaggaccgctaaaaccctagttcaggcggaggaggagggggacaagaataggagagcggagaagagtgtagggagggagacagggcagaagcaggggtttatgtcacggccgttggcccgctccgcgagacttgggtttg

>TaeST2.61127.1

ggggagagcggcggcggcgactcgccgacggagccgacgctgttggcgccggagtccatgccgtccgccgccagttggcgtcaacggcggccctgggagccggatgggatcagggagctgggaggggagcgcgctggggctgtcgggagcgcatggcggggctgtgaggatcgggaattcccgggattcgatctcggagatccaaccgaggagtggaagtggggaggaagcaggggaggggagtaaagaaaacaggggaggggaatggagttttccttatcgt

>TaeST2.61145.1

tatcagtgacctacatttttatacacatttactgcagattatcttctttacagtttttcaaacaactaatagactgtctttgcctcacaaaagaagaactaatagactgtctttatgacattccttccatccatttatatatgtatatattgactcaaatatattcttctcacatgtgtagccatggttcttcacatgaacatccttttctttgagaaattataacagcgccagttttttcacatgatgcagtgacctagaagtgaacacacacacacacacactaataaagtcatggctgcaatgcaaatatatgctctactaactaatgggcatgcttcaagttattttacagaatccatgcaagtattgtctctgcagcgatatagtacggcagaaaaacacatgtgtgtgaagccactcaaaaagaaaattaaaaaacctgacaaacacaagcaaacactatctggtatagcctttacttttttatgtccatgtactgatgtaccccatactatctgcagcctaataattttgtatattgtgttgatactccttttttaggggtcaatactactttctgagatgacttacagtagaaatttccatccatcgactgctattcaatgagaaaatagaacaacgccacaccactaattattgccatgtcccagacctagaaccacccaactatatcacgcaaggcatgccatcagatttataaactattcataataaaatatcaactgcacatagcaacatacgagcagaaagggaaggaaacaacctgcatcttacttgttgagatctggaggcagggccaggataagggtcgccagttgaggaagaagaacgacaggctcctcctcaacagtagtcggtgcggtggatcctccggcgtatggtgctgttgttcccggcgcacggcggatccttggcggcggacggcgctgcggttcctcggcggcgaacggtgcacctccgtcacagaaggcactgctgctactac

>TaeST2.61150.1

ctatccagcaaatcaaatttgcatcaacagattgtatatatgtttgtacaggacgacgaaaggaccaggagcgacgccattaaactaactaatcttatctctcgaggatcgttcatcattagttaatgatggccagggcgggcgttggatcggttcggttcagttcaccggagcagttcatacatgcctgcgtacacggggtgagcgaggtcaacgggatcgatccaactgatggatagtactcaagaacacatcgaatttagcttagacacttacacatgctcttgcgattgcttacttatatataattaaggagtaagtagt

>TaeST2.61154.1

ccgtggtgggcgtagatgtaggcggttctccacgagtagatgtgatccccggcggcgagggcctcccggcccacccgattcgacagcacccccgccatcgacgaatccagcactggggtcgaaatcgaaatcgagttccccaatctgaaacggcagagatcgccggggctagagcattatactcatcctcgtcgcgcaaaccctcgccgcctcggccccgaggtgtcgcttgcttagcggcaaaggatgccctggatggatgaacgcggacgtagacggcaatcggttagggctggttgcttggttaggttaggaagcaagggcacgaggaaagcggccgttgctcccgcgaggccgcgaccacgacgaccacgactctctttctct

>TaeST2.61192.1

taagactgacaatggcaatcatcatactgctgatatcaaggtgaaagaaatcattcatgtttggattaccatcatgccaccagtatccatgcaagcacggaattgggttcctcgccagaaaaggggaaagatagcggcagggggaggccacggcaacagagtacagctcaaaaaacatttggtttccgatcacaaaagcatactgtaggtcagaggatggcgacgacaaaacaaaaaaccgcgcggaaaaggctcggagatccagctaataagcgtttgtgttccacgcgcctccatggtgccaccaccttcgttgccttgtaacttgcatcttctctccttctgggttttgctgtgctgaatggatcatggcttggtaggctggtacaccgccgaatct

>TaeST2.61222.1

ttgctcttataaatctaaattaggataaacaaggttttatatttgccatgcaaagagaaaatcgatgaccgtagaaataaggaatcatgtattgtcgagtgacactgcgtcgctcgctcgctcgctgctgaccgacactgggactgggcgtcgacccgacaacacttttatttggcccacgtcacgcgcgaccccgcgcaaagcaccccacacctagtacagtagtaaaaagtactgtagcactccattccactggctgactgagcatcacgccgtgccacgtaggagcacacgaccccgccgccgtggacgctgacacagccagctccctctccgcgcgcgcgcgcctcacacgctgacgctggccactgcggcggccggctcctctgccgcggccgcgggtgcctccacgggtgtttcctcagcagcagcgggagcggccgcctcgggctcggcagcaggcgcggcctcctccttcacctcctcggccaccgcggcgaccggctcctctgccacggcctccttggtctctgtctcgaccacggctggcgcctctgccgctttgggcgcctcggcctccggctcggccgcagctgctggctcgggctccttggtctcctccgtttcggcggctgccggtgcctcctccttggtctcctcgacctcgacgggggcaggcgcctcgacctcggcctcggccggcgcgggggcctcctccgtcttcgcgacctcctcggcggcgaccggtgtctccacggcgggcgcctcctc

>TaeST2.61260.1

gtgttcctggtatagattcatctaggtgcaaagaaaaggagctgcgtcacacggcaaaaagatacaagaactgacaacaaatgctaggccaaaaggcacaattcagagcacttcaaagataataggtgcatactcccataaaattcaagtcacaaaacacataatcctcgcattacataaaactcagtcctggtacatagccagactagtattaaacagctaaatttgaccaaacacaggcaaagcaacttccaccgccacaaacaccaaacaaatcaggaatcactaggacaatgccatcatccctcaagagtttcgctgcctggatgtgacatgatgcttcccctccctccaaggcgcctacttggatcgctgcctcatctttcggcgcttcctcttgagcctcctcatcctcttcttcttccactgca

>TaeST2.61314.1

acgcctcgcaccctctaatcagatcgtcagttcgtcaccacaggctcacagtagcactagctagcttcagctagccagcctcagccactccgggcattttcttgcatccacgaccacgacggcaatggccaccgctgatgtccagaacccgacggccgcgctgacggaggaggcgcccgccgtggagacacccgtcgccgccgaggaggtcgcgaagac

>TaeST2.61352.1

tgcttcagagtcaaacggagcttctccaaaacaagcaatgtcacttttatgaaagtaccaaattaatgttcaaaacagatacaaacagtttcaaaaagtaatctgagtcacagataaatgttcaaaacagcaacaagaaataatccaacgaactgaacttgtaaacgtctgaaattgtttgcgacctgatgcctatagacagacacacatggtcctctaggatcatcacgataaaccgcaaattgtggaattgagaacctgggacgacgccatggcctatccacagcccaccaatgcagctaggttacgcaggcctcgatcgtaggtggccatggcagctggtacgcgtactccctggcatggcgcttgtcccggagcagggcgtactgtgcgtcgcccagcacgataccagagaagagctcggcgttctccttgtgcagagcaaggataacgttgtctccgaccgtccagtaggcggcccgcttgctgctccctgcgaaatagatgggggccaactccc

>TaeST2.61389.1

ccaagccccagccccacccggactacggccccgtgcccaagcccgagcccaagccgcagccccacccggactaccaccccgtgcctcccacgcccacctacggcggtgggggcggcggcggtggataccacggacaccactgactcagagtctcagaccagccaactgagtgatcgacgttgctatttatgatccgtttgaatgcgtgggtgtattgtttggtgttctgccattcgctgccccgttttagtatttgcaccacagaattgagagttcagaataacagtcacgtactacgggtgtatcaggcgagtgctgtttgctttatcaagattttgtaatttcagtgtgtgtttttggtataagaagttattatgaatcaagtaattccaacaagtcgatttgttcgaggcgatggacaagataaggacagt

>TaeST2.61422.1

caacacaaagcatagataaaggccaaacatctatagataaatataagagactgcaattgccataattatgtagcatccattgatgataaaagagagtagcatagtaccacaatccaaaacttgacatcaaacagagacatcctgaagcagcacgattcaaagtcttaactataataaagcaaagagcattagacagctccgagctagcgaaaaccttttacgccaacatcctaagcttcaaccttctcgactggtgcatcgagcatcagggccttggtaggtctcgccaagatgtcggtgaactcctggtacggcgccttggtgaaggtggtctccctccagaagtcaggggtgaggaatccataggtcttcatcaggcagtcaaaggtagcctggaaacaaaagaat

>TaeST2.61478.1

gccatggttcatgtaaattcgcaggaagcacgggaaccaatggcagcagcagtggtgcggcttctggacccgcgagccaagatagttctgcttcacaatctcaatctttctggttgacttggctaatcgccatgctgctgcccgttctacttctcatgtaaaacattgtaaccgaacgttgtattttggatgagatgcgtgagatttgttgcgcctcttgtatttaggctatttgtctcgctctattgaggtagatgccatgtacgtatctctcaattttagttgtcctagaaatgaacgggcaaggtgcactaattttgaatgtcaagct

>TaeST2.61487.1

tttttttttttgagacgatccatgcctagttaaggaccgagatctcggcacaggcactgggcctttgctcacgcatgcgctgggtccactgagtgaacaaagtccggccacaatacgaacagtctgttaagtcggttacaatacatcagcataacattcactcagtacgagaagtctcgctgaagctgggtccgtaccttctactgaacctgttaagagcgagcaattcaaccagcaggcgtatcaaagtgccaacacatgtttaacagccgtgtaatcatacacaatccgcacatgaagaggttctacgagttcagaggatcttcgacagtccttgtctttttttttctaccgcggttcagacatcagatataactagtagcaagggaggtttcacatcagcatccaacacccttggcctagtagccctgcttcatcctttcctcgaatcgggtcttctcatcctggaaa

>TaeST2.61565.1

aggtgctcctgttccttcagtttcacagactacgtctggtacattgacagttcctgctaaaatatatggtgctcctgttgctgcagtctcacaggcagtgaacttgacaggccatgctgaaaacgctggtatctcggcagtgaaaaatgcaggcacaaataaggttttgagttcggaaaattccaatctcagaaagcatccaaggacagaacaagcaacttttgagcaaggtcctcgtccaaaaacacaacggtcaagtggggcttttggtgaagcatgtctggcgtcagtgactaaagctggagcgtgctcgcaggctggtattgtcctggaagcgcctaagcatgaagttgagcaatcctaagcatgaagatcctaagcgtggtggcccttctggacttccgagtggagaaacagtcgaggaaacaatatgctgttgaaacgaaataaaacaataattctgctgcggcttgtactagcactctatttatgtgacctacttgaggcttgcaagtctcctggagtctatgttatgttttgacgcaactatgttcctgagtttctaatatgaacttaatttaggtgtaggagagtgaagtattgcataatcttttttattctcttctccaacggccta

>TaeST2.61578.1

ggtcggatcaaaaatctctcaaattcattaagcatagcacaaacaggatgtcaaatataactactcttgatatgattcatcacaaattcggagcagcagatattgttccattaatattcagatcgaccaggcaaaaggcagcaccagagaaacatcctcttacaaccaaccacgactaccacactgaccatcatccttccaacgacttccatcaagcacgactactaatactactatatatatctagataggcaggcagatccaccacggggatcaacggcggtcaccgggagggggggcgggcgaacctaggaggaggtgaacttggtgacggccttggtgccctcggagacggcgtgcttggcgagctcgccggggaggacgaggcggacgga

>TaeST2.61594.1

tctacatggtcaagcatagattagggggtacaaatgtaattggggaaatctccccttgcccgaacggtcgtgcggttgctacagcaaccacctctcttctagaaccgacgcatccctctggatcgtcgcgtctttccagcacatatataggttgtactctcaattgatcaatgacagagatttcactttcaatctcgaatagttcgcgaacagatacgcacactatccgaatgcctagcgatgtcgagcacacaggcttcgaattgggccagcagttttttgcgtta

>TaeST2.61622.1

gaatttgcatgcccgagtttctcccccacctgctgaagaaagtgcatcctcagagcctgcttatcacccggtggattggaagcacagggatgaacctccagttcctccaaaaaatgatggatttgatgtgggtgcttacccagtaaaatgggatggcaaggatgaatctccatcttctcaagataatggttttgctgttgatgctcacccagtaaaatgggatggcaagaatgaaccgaaagcttcttctc

>TaeST2.61623.1

caggggaacaacattccagccatctccatctactcccgagcctagcttggaggggttgcttggtccaccattggccatggctggagccatgcgcacgatggtggtagctctcgtcctctggtgcgcgatggagccgagcggctctgtgacggcgcgcgcgacatcagacgccactggcaccctggcagaaaacgcagatccatcctttactgcaaatcagtaccccgtgaatttg

>TaeST2.61672.1

agttctcaccttggcgaagtcggtgggggcgacgccggggaggtagaaggcggcggcgggcgcggcggcgaggagcgcgaggagggcgagcccggcggcgagcatcgccggcgcccgcatcggggggtcggcgcggtggcgtggatctgggaggggcgggtggatcggcgaggaggaggaggaggaggcgacgaccaaaccgcgcgctggctgggaaggcgggtggggtggggatggaagtagagaagcttcgggtgcgcggatctctgcgccgggggttttattggataaccggctctcgcgcccaggcccccgaagaaatg

>TaeST2.61682.1

tttaccagattgcaagcaatgatatttatgtcatactccatccgttccaaaataagtgtcttgactttagtactttagtacaaagttgaaacacttattttgggacagagggagtataaaagaggctctactacatcaacgcagctacataagcagagatccatgcaattgcattcaaaacttcaaggtgtcatttcactcatcgagtatagacatctagttccttattccatcatcaaacttaaaatagctggtaggtgctcacaccaacgggagaaaagggaagggggagcaaggcacgggttaattcaaacagaacagatgttttcttcatacaagaacaacattcatgatccttgcaagcactctagtcaccaacccatcctg

>TaeST2.61737.1

ttaagcgttggagagaagatactgctgattatgaaaagtataattgagtgttgaatgggaggatcactcgattcccccattacaacacacagagaattatctattatatatcatagaatgtgttatggggtattacaatggccgttagacgacggattgtgaggagaatctattatttttcctttttctagggatgagagcatgttattcttctttcatgcgtgtaattcaaccgtccattctggcgggcacttgggatcgcagttagggcatttagacttgcactcattaaacgtcttgaaacacggtgtacccttctgactgtcacagcagtagcagatgccaaagttacaaccatatttgatgcaaaattggacggtgaccttggcatcatctaatggtgatactgagttgggcacagtagcattgttatcaccgctgtctaggtagctccttccggcctcatgacgac

>TaeST2.61753.1

cactcttcaagatccgatctgccggagcaagagctactactagaacaaatatcatgccacgagccaccacaacgaagctgcaagggcctagcacacagtgtagaagtgaccaagaaagaataataatgatacatatggtgagatgaattaaccgggggattgtacaatggaatggatacggatccaacgacgacgacgaccaaacaatcagatctgtccatgcacgcccaacaagctactggtaaccgaatcgaatgccgaatgtaatcatcatcaaagcaacggaaaaaaaaaagagaagataaacagctccaagaacaccaccacaaaaggagagggagcagggccctcggataaattaaatacgctggcagccgatctgctgcgggggcggatctctcagtagaaggcggcggcggcggccacggcggcggcagcggcccacgcggcggcggggtggacggtgccggcggcgcc

>TaeST2.61922.1

accacaaaaaaataacactagctcacagatttcaaattcagatgaaacacataatatttaaaggtttttccaatcaccagctccatgccgtgcatacatattaaccgatgctagaggagtacatacatacgtcaagcatactatctcatcactgatatactactatatgttgattccgatccatcacagcagcacacaaattaaccataattcctgccacactaatcatgcagtagccaatagatagatagatagatagagtactgatcgctagactaggcatggatcatggctagaaacaagagactaccacgggcggtggaggacgagtacactgatgccgaggcggaaaaagacccagagcggaattaccagcaggatgtagtcggcgatgaa

>TaeST2.61951.1

ttttttttttttgccttcttttccttttgcatcttgatttttgatctctcttctcccggtggatgtaatatagtagtacgtactaccaccgggaaaaaaaagcactggaccggcttgcgcttcgttgttgttccccggactgatgaactcctcaaatcatcatgtaatattatatatatcacactgctattgtacaatatcttgcgtgca

>TaeST2.61979.1

tgcctcgccccgcctctctccccgccacacgctccgacgaggcgacggcgcggccacgccaccaccccctcctcctcctctccaacccccgccgccaccatctccccgctcccgccctcaagcacgttcccctccatccgcgacgctgggtttccaggccgagggcggcatccgcgttgctctgagcagatgagggggcatggctttgtggcgttatcgttggccgccctgttgtgcctggttgatggcgtctactctggccgcacgagctcctatgtgcgcacggagttcccgtctaccgatatgcctctggacagcgagtggtttgcgcctcccaagggctacaatgctccgcagcaggt

>TaeST2.62023.1

taactgctaagcatagtctgacagactccatatatattcatcaacaaatttacatacatgcatacatatacgtactgcacacgctagtaatataagtactgatcgactgtactaacactaacaaccacccctcagcttaataacttattcattaggagctcaccctcaggtttacgccgcgcccacgcagcctaaacaagggaattaagcgaaagaacagcaccaaaataaccagcccgccatgcatggatcaggcggcggctgaggagggggccggcgtcatgggcacgacctcggactggtagcggtggaagttggcgatgtagatgccggccttgttgtccacgtcccgctcgcccacgtagtagatgccgtcctggtcgctcggcctcacccgctgcggccgccggcacccggcctcctcgtacgccccggacgaagagaagcaaccgcccattgccttcttctcttctctctttttttttgaaacgaggccttcttctcttctctcgtggctcagctagcttggatgatgtatgttgggctctcgatcggcgctgtgttagcctgggaggaagagg

>TaeST2.62049.1

gacgcccaatcataaacaagaaaaagaagcttgcattgatcttatggagcggaaaactattcttctttgcctttttgtgcttttgctgctaggaaattctactcatgctgaaatgtgtgaagtgcaccttccttattctggcattatttgcgtagaatcgggttgccagactgcctgccgagagtcctggggagatcacaccaagaaagcttattgtgtacctgtcaacgcttccctttggacttgtcattgcatagtatgcaatgactagaatttgaccagtccatcatgttgaacaatgtacaatgatgtgatccactaataaaatatttgtgcaagatgcatgtgagaatccatattgagcatacaattttgggacacttgtgatttgatttcatttggtcgtctgttatcttaggtttgatgattttgtgtttgcatgacttgtgttcgtaagttgtgaaataaccatttgtggcaca

>TaeST2.62050.1

acagaaacaagaagtttgcctctgatggactgcaaaaatattcttctttgccttatggtgcttctgctgctgggaaattccactcatgccgagtgtgaagtgcacgttgcttttacaggtcctgcatgcatagcattaacttgccaatatgcatgccaaagttcctggggagatcacaccaagcaagcttattgtgagtctgttaatgttgccacttggaaatgtagttgcaacgtatgtaagtggagttgaactagtccatcatgttaaacaatggacaataatgtgcctgtgagaatgtctattgagcaccaattttgggtgtacctgtgatttgatttgattttgtcatatataatctctggtttagggattgtgtgtctgtatgg

>TaeST2.62052.1

aaccagggctaaatgttttctagctacaaatctataagataacatcatgtatgcatctcgatgcccaaacagaaactagaatcagaagtttgagcctgccttatggaggggaaaactattcttctttgccttacggttcttctcctgctaggagattcaactcatgctcggtgctatgaaaagcgtgcttactggatacctttgtattgcctagacgaaaatttctgccagaacaaatgcgtggagaagtttggaaagatcgtgaaaggttcttattgtaaaatgactggatttttcattgtctcttgtttatgttcggtctgtgatttgtgactgtatcgagttggacttttccatcatgtcaaataatgaaaaatgatgtccagtaataaaacattatagtgacatgcatgtgatatt

>TaeST2.62180.1

gtacagaggcggatttgactagaccagatctcgtagcggggtctccggccgcctctccccccgggccgagcacgtcctgccggccgccggcggcctgctcggtcgtcggtgagtgcgtgcagcggcctggagaggcgccggatctcgcggggcaattgcgccggcgacctgaggtaccttggtgcagaatgaagtgaagattgcagagagtatcttggtctcattaatcaatgcatcatattatgtgcgcaataagattttgctatgctttcaccgtagatgcccactggttggatcatgcttctgtttatgagctcattgaagtaactctctcgtagctcaaattgggccaatttctcgtttgcgtggcacacaaaaccttcagctatccatctccatatcagttggtctctcttaattttgtaatcttctggaaatatgcttaggtataataaatgatgcgaaggtagatcatacaatatgaacaatattcttaatctaaagctagatggtgtaggcaagtgccagtgatttaacacaaatagaaggcacacatgcaccaatactatgaaaagtagcaaagaaaatgagtaaaagaggcacttgacttgtagattatacaagtgtgatgatcttaaagtcaaccctcttttaccagtagtttgatctaccttctcatctataggattagttattatgtctaagcatgtttttagaagattagaagattaagcaagctcattcgatatgtaaggtggatagtttgaggttttgtttatcatgaaaaacaagggattagcaagtttgagctcggtggtagaattgcttagtgagatcgtgaataggagcatgattcaaccaattgacatcaatgttgataaatgcatggaaaaatcttatgtatacatgatatggtgattgatttcatatgctgaagaaaagcacttgcctggcactgatgaaagcggttcatccaaaggcaagatttgaagattatccatcagaatgagcatgacaggtagcagtgatgtgacaattgaggtcctcttgcaattgttgatttggtgtagcttatgttgaatgttccaatttctacatgcgctggattagaatattgtgatgcatgtctccattcttgcaatcacaatgaatcatgaatattgtgtttgccttggtgtttcggtttgtcttattcagtaacatgaatctaccattgtatttgtacccactgatgctgaattatagtcatgaatctcttgttgtatttgtacccactcatgctgaattatattgtcacttaggcaagaaccatcaaagtttctgtgttgtcctgtagttgtctcgtgatcaacttgttcttccattcctttattttgatgctggcttgtaattctgtcttttgattatcccatggtgtctctctttataataggagaagcagctttaaaagtatttttaccttacgctctttgaatgaaatttaacgtcattaagttggtgcactgttctgaaactttgattttctttattgacctgtatatgtgacatttgttcaaatttgactgaacaatggttgcagtgctactagcctgaactgatacaacgatttttgcaggtaaaat

>TaeST2.62187.1

acgtcaaaacttatatatcacacaaagatcaacaactcgctccttcaccacaaacgacgagttgaaatgaggaatacccatgtgttgcttatgtcattggtcttactcgtgctagcatcagatgttctagtggaggcatcaagatattctagcattaaagaattcgtatgcgtggagaaaattttcccacctcgaacatgcgagaccaatgcttgtgaacagtcatgctacaaacatacaaggcaatacaaatctggcgagtgtgttgctgaggggtgcaagtgcaagttatgcatatatcagcctccggccatccatgataatggaaaaatgggttctaactagctaagataggattgttttttagcaaataatgtggcacaatcacaaatggtgtagaggcaaggtttttcactcatatgagccatatgaagagtgatgaacttcgtcttgcttcttgaattatctggaaataagaaatggtttataaaatctgaagaagtggtctataaattttgggtgcttgactttgcatgtgtatttagttgaataaactgtttttttatgaagaaaatgatatgtttgttta

>TaeST2.62241.1

gccatacaccacaatccctaaccctatcctcagccccttccgtctgcggcgacagccgccgccaggccctcccccctctgcgcgcagccggcgtgatcccaaccggattagatccgccaaccgccgcctttcctcgtccctctgcgcgcagggtggacggcgaggggcggcaagggaagttgccggcgcgacctcggtccgatccaaccgagatcggccgcgcggggaggagccggcagcgctgagccgcgacggacggtaggccttgatgcccgaaggaggggaggcgcaaagagcccgtgaatcagtccgagtcaaacagatctggcgccgctgacaaaagctgacgggataggtctggtttcgccagatccggctttgcttttgcctgacatctttatctgggaaaagaaagagatgacgagctgcgtcatacagggaccactaatccaagacggcggatccgcacacccctcccctcaacgctgccgtctgtgtcgatagtttcggtcttcttgtatttttaagatgtaatcttcaatgatgtgttgtgcgggtaatatggcatgatctttaggtcgttttttttgttttctttttcttttggttttctatttgaatctagtaacaacaaaatatgtaagtacgttaacttgtggtaagagattaagattgttggttcttcaataatatattttttgttagtcttctaaattttgggtgtacatatctccaatttttttttattatgacctacaaagtgctatttacagtaacatagcattttgatttttt

>TaeST2.62567.1

tggagtagaccggcttcttcttgaggatcttcttgggcgcgggcgacgcggctgcggcagctggcttggcctcgccggccggctcggcggcggcggcggccttgacacgcctggtcgatgacggcgtccatcttgctggcgtggccagcgccgtcgtcggagtggcggtgccggcgaagagagcccgcggcgccgaggcgagagcttccatggctgtggctctagagctagctgggaaaaatccacgagaagctgcaccgcgcgcggcggataaggcggccacggcggtgaggtgaggctgg

>TaeST2.62583.1

aggagtaagaagtttgtgtcttctcttatggaagggaaagctattcttctttgccttatggtgcttgtgcacctaggaaattccattcatattgataaatgcaaggatctagttcacccgcaaacaaaaacatgcagggatctaactgcttctatcgcccgtgagtgcgttttgtcaaaatgccaggtgacctgcacaaatttgtatggagcggaactcatattctcatggtgttacaaccccgtcccatactattggcattgtcggtgccgtgtctgttggtagagttgcagtgaacaatcatgtcgaaaattgtgatccaataataaaatattactgtgacatgcatctaagaatacataatgattgtcaaa

>TaeST2.62916.1

aaggccgcaggtcaaaacgaagacacatcctatcgttcttggatcagatcaaatattgcagtcgtggtcgatcggctgcaacatgggaaattgcactaagcaagttcacgcgctgttgctacttcttctcgcatgcttcgctattcatttggagtttgaagtcgtcggtgtcgatggcgagcgtattcctccgtggtgcacaaagagtccaactataccaatctgcaacccagataaatatagatgctattgttgtgtaggcaattggcaatgctacaaaacaatggaagagtgcagagccaagtgtgcatatttacctccctcttcttcctctgtacttccatgaaaccgggttcttatcatgagctggaagaaaaatgaataagatccagacaataaacaatttgctatgaatttattggcaattattgtttgcagttt

>TaeST2.63020.1

ttaagatctagagaaaataagtactaccaagcaagtcttgatcatggcacttatccagagcaagacacattttatctattttgttgctcttctgttgatgtgtaccactttcttgtgtcaccctgctatagcacggagggatataggtaccaaggctatacttggacaactatgttttgctttctcggcgtgcgcaggagcatatgactttgctttggattactgcaaggaagagtgcgtactacgtggctatgacaggcaaaaaggctactgcgttaaggcgactggaggagtgtgctgttgtcttcctcccttcctaaagtgacagtaatgttacaccatcatgttagaaaattgttccatgtaatcgctagacttattaccaacttatgtggatcgaaccagaatttcaatgaagcatactagtagttgctttcgaag

>TaeST2.63164.1

agcagccaacaacacaaaaagaaaaggaacggctaactatctcacatgccacatatgaggaactacacatgggccctatgctttgtagctcttgtagccatggccaccactttcttctcaggtcatgcaacggggagttacataggtacaactaacatgtgtactggatctgacaaccactgttcgccaccagaacgagctactggtaacattctatgcaaggcttgttgcgctgcccaaggttatgacaaggataagagcagttgttgtccaggcagtagtagctcttgttgttgtgtatgaaatgagtttatatatttgctaaaaatattgcctgaaggagaatgaatgaatagtttcg

>TaeST2.63189.1

accgccgacacggcggcggtcgcctcgccgccggcgctcgcccccgcggcgactccgcggcggcgcaggggccggaaggaggcgcgcgcggcgcataaggggaggcggtcgcgggccgctggtggcgacgggcggaggtggtgggcggtggcgacggacatggatggtggtcgagggcgaagttggtgggtggctatcgggaagtgctggacgggag

>TaeST2.63212.1

ttgaggaaatcacgagtgagggaaaagcatcatcaaaactagaattaaatggagagcgaacatatatcagcagatcaaaccggataaatagcactggtgagaagtatctacaagcacacgggtacaatctctaccaaggacgtaataataatcatcatcgtcgtcatcgagtgacgggcaggagcaggcggatctggccaaacggacaggaacaaccaacaaatcgatcgctagcggtaacagccagcagcggcgttaaacggatctacgcggcggtactactatgtagatgcagaggaggagccatcgacggacggacgggggcggccgcttcagcggcggacggcggcgaagaggaaggcggcgccggatgcgacgagcgcggcggcgagggacggcgccg

>TaeST2.63238.1

caatatcgctaatggcgccgcccctactcctgtctaggacgtgtccacgtctcctctatttaatatgttttcttcgtgattctagttcaatttcttgttctcacaattggactagatatgacatggtcaaattcactactacatatgttattttctcactctctgatagactacatgactagtttaatcattaatattatggtcttgttttatctactatttttatggattaatcatatgataaactgctcatatttccaacacgccccatgtacttgtatgggtgtaacaaacacatcctatgtggtcagtcttccatcatcaaaatggtccatggcaatccaatgagattgatgcctctaatatctatgtttatcagaaaatataattttatttaattattggcatcagtatacatcattgcattgcataagctgagaaactttgtggctctcattttcatatagaaccgaactgtctacagcagcactgaagatgatggttgttggtttcgcattccaattgataaggtgggagaaagcctcttgctaagcagtcactctggcacgactcatgagtgcatcccgctaaatccctgcatgagcgtcgttgaactacaatgcagtacatcaaataagaaaacaaattagtaatacagtccataaaactaaaatattt

>TaeST2.63241.1

taggacatatcaaacccatcaattaggcaaccatgagttcgatttcccatcatcaaaccaaaccatgatagcaaaacaaggaatgcacttcttacagatacacattgccaagggcacaagttaaaaagagattgcgctcccttatcatcatcagaccaaaccaaggatcgctcactcactccctcacaggccacaactaattcaaatggctttctttcttgcccacctttttgggcaaaccggaagaagtgcctacaaaattaaatccatgaagttactgacggcgacgatcgattatgtgtgcacgtcacagcgaagaagccgttgttggagtcatgcccagcgccttgaggatgaatgcgtaggtgaaggcgtcttctcgcaacttctcaaacct

>TaeST2.63337.1

gagctttacaccggtaccgccgcccattccatgtgcgtcatttatttgcgttcccttccagcttaacttcctccatatcgctagcctctgccctcagcccttgcagcaccattcggccgttgccttcttccccacatcatcatcgtcacccgtccacagacgcggagagaccgactcggcgatggatcccccgcctgctcagtccatgtctgcgaccgacgacctccacgacaagagcctcgacggatgctgttcttgctgctacgactgctgctccagcatcctggacttcctgtgctgcgcttgatgcccctcgaggcgttccccggcttccggcggaagcgcgcggagatgcctgagttatttcggagtactatagtcattgggggatatattttatatagtctgtaaggagatttaaggatggatcaactgctgtaatgtgttgacttatcttccgttaaagtagtcgttcaaagaaattcacagaaattctgttttgcgta

>TaeST2.63348.1
[truncated: 85,759 more chars]
