# Supplementary material for: Nanopore RNA Sequencing Revealed Long Non-Coding and LTR Retrotransposon-Related RNAs Expressed at Early Stages of Triticale SEED Development
Source: Plants (Basel). 2020 Dec 17;9(12):1794. doi: 10.3390/plants9121794 (PMC7765848; doi:10.3390/plants9121794)
Supplement: Supplementary file 1 [file plants-09-01794-s001.zip › Supplementary file S3.docx]

**Supplementary file S3.** Sequence of RTE7B LTR retrotransposon that is expressed and produce eccDNA in triticale developing seed at 10 dpa.

>7B 7B:312336869..312341902 (+ strand) length=5034

ATTACTAATTGTAGAATCAGGGGAGAGAGGGGTGGCGGCTGGGATAGAAAGATGGCCTGGTATTGTATTCTATTGGAGTTAGGGTTTCCTGTACAAGGGGGCAGCACATATATAGGCTGCAGGGTTACATGGGCCACATGGGCCACATGGGCCATAAGCGCGCGCGCGCACACACACACACTGAAATACAGATAAGACACAGCCCAACACTCCCCCTCAAGAGGGATGATAGATATCTATCATGCCCATCTTGTCACAAGCAAGATTGTTCTCCCTGGTTCCCAAACCTTTTGTCAAACAATCGGCGACTTGTTGTCCAGAGCTCACATGAGTTATCTTGATGATTCCTGCATCCAGTTTCTCTTTGATAAAGAATCTGTCTATCTCCACATGTTTTGTTCTGTCATGTTGCACTGGATTATTAGCAATGCTGATTGCAGATTTGTTGTCACATCACACATTCAGAGGATCTTGTCTGAGCACTTTCAGCTCAAGCAGAAGGCCTCTTACCCATAACATCTCACTCAGCCCTTGAGACATTGCCCTATACTCAGCTTCTGCAGTTGATTTTGACACCACCGGTTGTTTTTTGCTTCTCCATGATACCAAATTTCCACCAACAAACACACAATAGCCAGAGGTTGATCGTCTATCATCAAGACAGCTAGCCCAATCAGCATCGCTATAACCGTCAACATTCAAATGGCCATTACTCTTGAACCAGAGTCCCATACCAGGGCTGCCCTTTAGATACCTCATGATTCTATAGACTGCATCAAGGTGTCCACTTCTGGGGTCATGCATATATCTACTTACTACACTTACTGCATATGTAATATCTGGTCTAGTGTGACACAAGTACAGAAGTTTGCCAACCAATTGTTGATATTTTGCTTTGTTAACTTGTTCACCCATCTGTGCTGTCAATTTGTGATTTTGTTCAATGGGAGTTGGGGCTGCTCTACATCCAAGCATTCCCATATCACTTAGGAGCTCTAAGGTATATTTTCGCTGAGACAGGGCTATTCCCCTCTTTGATCTTGCAACTTCGATACCCAGGAAATATTTTAGCTGACCAAGATCCTTAACTTCAAATGCTTTGCCTAGGCATTTCTTTAGTCTTCCAATCTCCTCTTCATTGTCTCCCGTTATTATGATGTCATCCACATATACTGCAAGAATAGTAATCTGTTGCTTGGAATGTTTATAGAAAACAGTGTGATCTCCATTGCATTGTTTATACTCCATACTGCAAACTGCTTGCCTGAACCGGTCAAACCAAGCCCTTGGCGATTGCTTCAAACCATAGAGAGATTTTCTCAATCTACAAACCTTCCCAACAGTCCCAGGTGTAGAAAGTCCAGGAGGAATTTCCATGTAGACTTCCTCTTGTAGATCTCCATGTAAAAAGGCATTTTTTACATCAAGTTGGTGTAGTGGCCATCCAAAGTTAGCTGCACATGAGATTAGAATCCTCACTGTATTCATCTTTGCAACTGGGGCGAATGTTTCGTCATAATCAATACCATACGTTTGGCTGTACCCTCTGGCTACCAGTCTTGCCTTGTAACGCTCAACCTTACCCTCAGGATTCTGCTTCACTGTGAAGATCCACTTACAACTGACTGCCTTCTTCCCATATGGAAGATTTGCTAGCTCCCATGTTTTGTTCTTTCTCAGGGCCTCCAACTCTTCCCTCATGGCTTCACACCACTTTGGATCTTGTCTTGCTTCTTTCCAGTCTTTAGGAACAATCACGGCTTGAAGTGATGCGACAAATGCTCTAAATGAAGGTGATAGGGCTTCATAGGTGACATAATTACTGATGTCATGCTCAAGATTGCCCTTGCTCAAAGTTTTCCTTGCTACTTCCTTCTTGAGAGCAATTGGCAGGTTAGGTGATGCTTCTTCAGTACTTGAATTTTCATATGACTCCACTTCAGATGAGCTTCCTGTCGCTTGAGTTTCTTCTGACTGCTCCCTCTGTTCCATAGTTTCTTTTGACTGCTCCTCATGCACTTGTTCCTCCACACCTCTCTTCCTTCTTGTATACACCTGAGGATTCCTTTCCTCATTTGGTCTCTGCCACCTCTGCTGTTCAGCCCCATCAACTACAACTGGAATGAAGGATCCCACTATTTTCGGGGTTGGTATTGGCTGCTCCTTGTCGCCATTGGTGCTGCACTCACCACTCAAGTTGCACTCCCCCTCTTGACCACCCTGCATAGATTGTGAGTGGTCAAGTTCTTCGAATAAAGTGCTGAGATCTGTCTTCTCACCATAGAAAGGCTCAGATTCTCGAAAGGTGACATCCATATTTACAAATGTGCGCCTTTCAGCAGGACTCCAACACTTATAGCCTCGCTGCCCTGCAGGATATCCAACAAATATGCATTTTACAGCTCGTGGATCCAACTTTCCCACTGCTGGTCTGTGATCTCTAACGAAGCAGGTGCACCCAAATAACTTCGGAGGAACTGAGAATTTGTTCTCCCCAAATAGCATCTCACATGGGGACTTCATACCAAGCATTTTTGAAGGCATCCGGTTGATCAGGAACGTTGCAGTCATGACAGCTTCACTCCACAAGAATTTTGGCACATTCATGGTGAACATAAGTGATCGAGCCACTTCTAAAATGTGTCGGTTTTTCCTTTCTGCAACACCATTCTGAGCAGGGGTATCTGGACATGATGTTTGGTGCATTATGCCTTGTGCTGACAGGAAACCTCCAAATTGCTTGTTTACATATTCAGTTCCATTGTCAGATCTGATCACCTGCACCTGCACATTGAATTGGTTTCGCACATAGGCATAAAACTCCTGAAAACATTGGAATACCTCATCTTTATGTCGCATGAGGTACACCCAAGTCATGCGAGTGTGGCAGTCTATAGATGTCACAAAATATTTCATTCCACTAACAGAAACAACAGGACATGTCCACACATCAGAGTGAATCAGCATAAACGGGGATATACTCCTTAGCCCCTTACTCAAATATGAGATTCTTGTGTGCTTTGCATATTCACATGCATCACACACAAGCTTACTCTTGTCTACTCCACGCATTACATCAGGAAACATCTTACTCATCTTATCAAAAGCCATATGCCCCATGCGACAGTGATGGATCATCGCCATTGTCTCACTTACTCCAACTAGTACAGCCAAAATAGGACTAGCACTAGACCTAGACACATCACGATCCATATACCACAATCCACTACGCCTGGTTCCTGTCCCAAGTTTCCTTCCAGTCATCCTCTCCTGAATCAAACATATTTTCTTATCAAGAGTTATTCGGCAATCTATCTGATCAATCAAAGCACTTAGAGAAAGCAAGTTCACAGGAAAAGCTGGAACATGTAATACATCAGATAATTTAATAGTGGGTGTACATTGAACTGATCCAACTCCTTTAATCGGTTGTGAGGTGCCATCGGCAGTTTGTATTGTTTCACAATGTGTAATTGGGTACTGAGTATATGATTCAAACTCAGTTGAGGTACCCGCAACATGCTTGGATGCTCCAGAATCTAATATCCAATCTGGAAGGAAATCATGTGTGGATATAGATGCATTTGCAAAGTTACCTTCCCCTGTGTAGACAAAGTGGGCAAAGTTCCCGAAGGTGTTGTCCTCCTGGCTTCTACTCTTCGACTCCCCTTGAGAGGTTGTTGACGTCCCTTCTTCTTGCGTTGCCAAGTTAGCCAAGTTTGCTTTGAAGCCACTGGAATAATTCGAACCCCTCGAGTTACCTCTGCCTCTAACTCCCCGGTATCCACCTCGGCTGTAACCTCTTCCCCTACCACGTATTTCTCTGCGTTGTGCTGTGCAATTGATGCTTATGTGACCTTTCTCTCCACAGTTGAAGCACTCTCTTGTCTCCTTCCATTCAGACACAGTAAATGCTGAGCGAGATGCATTCGCTTCTCCAGTCCTGGTCAACTTCAACCTGACCTCCTCCTGTGCCATTGCTGCAACTGCCTCCTCAAGCGAGGGGAGCTTTGGCTGATGAAACAACCCAGCACGCCTTCCTTCAAATTCTGAGCTAAGTCCCTTCAAGAATTTCATCACTCTCCTACGCTCTACCCACTTCTTAGTAGCAACTATACATTCTGAATGTGGTAACTCCAGTGGATCATAGTGGTCTAAGTCAGCCCACAAATGCTGCAATTCTGCTACATACTCCATCACCGACTTATCCCCTTGTTTTAGCTCATTTACTCTATCCTCAATCTGCGCCATAAGCATTATGTTCCCTTTCCCGGAGTACAATGTTTCCAGGGTTTTCCATACCTCTGCTGCATGGGGAAGAGTCTCCACGGCTGCTGCAATGGATGGGGTCAAGGAGTTCAGCAGCCATGCCACCACCAGAGAGTCAATGACACTCCACTTCTTCCACTCTTCACTTGCCCTGTTCGCCGGTTCATCTACTTCCCCTCTGACATAGCCTTCCAGCCCCTTTGTCCTCAATATTAGCAATGCCCTTCTGGACCAGCTTAGGTAGTTTGTGACCCCTTCCATCTTGATTTCATTTGGCATCAGTTCTATTTTCTGTATCACCTCAGGCTGAGGAACAAGCGCACCTGGGGAAGCCACACCCTCTGCCTTGACAGTGATGAGCTCAGCTAGCTTCTCCAACGCCTTTACCAACCCCTAGTTGTCCCCCATCTTTCCAATCTTGAATCACACCACTAGCAACCTCCAGCAACCACTGCTCCTCACTTTCACCTCTCTACTACCTCCTCCCCTCAGATCTGGGATTCCACCTCTCCTTCTTCCACACAAGCCTTCTCCTCTCAGACAATCGCCAGCAAGCACAACCAGCCAACCACCATCACCACTGCTGGGATTGGGAACCCAAGCTCTGACACTATGTAGAATCAGGGGAGAGAGGGGTGGCGGCTGGGATAGAAAGATGGCCTGGTATTGTATTCTATTGGAGTTAGGGTTTCCTGTACAAGGGGGCAGCACATATATAGGCTGCAGGGTTACATGGGCCACATGGGCCACATGGGCCACATGGGC
